# Diverse synthesis of C2-linked functionalized molecules via molecular glue strategy with acetylene

**DOI:** 10.1038/s41467-022-29556-2

**Published:** 2022-04-06

**Authors:** Bo Yang, Shaodong Lu, Yongdong Wang, Shifa Zhu

**Affiliations:** 1 Key Laboratory of Functional Molecular Engineering of Guangdong Province, School of Chemistry and Chemical Engineering, South China University of Technology, Guangzhou, 510640 China; 2 Singfar Laboratories, Guangzhou, 510670 China

**Keywords:** Photocatalysis, Synthetic chemistry methodology

## Abstract

As the simplest alkyne and an abundant chemical feedstock, acetylene is an ideal two-carbon building block. However, in contrast to substituted alkynes, catalytic methods to incorporate acetylene into fine chemicals are quite limited. Herein, we developed a photoredox-catalyzed synthetic protocol for diverse C2-linked molecules via a molecular glue strategy using gaseous acetylene under mild conditions. Initiated by addition of an acyl radical to acetylene, two cascade transformations follow. One involves a double addition for the formation of 1,4-diketones and the other where the intermediate vinyl ketone is intercepted by a radical formed from a heterocycle. In addition to making two new C-C bonds, two C-H bonds are also created in two mechanistically distinct ways: one via a C-H abstraction and the other via protonation. This system offers a reliable and safe way to incorporate gaseous acetylene into fine chemicals and expands the utility of acetylene in organic synthesis.

## Abstract (web summary)

Although acetylene is an ideal two-carbon building block, very few catalytic methods can be applied to incorporate acetylene into fine chemicals. Here, the authors show photoredox-catalyzed syntheses of C2- linked molecules with gaseous acetylene under mild conditions.

## Introduction

**Fig. 1:**
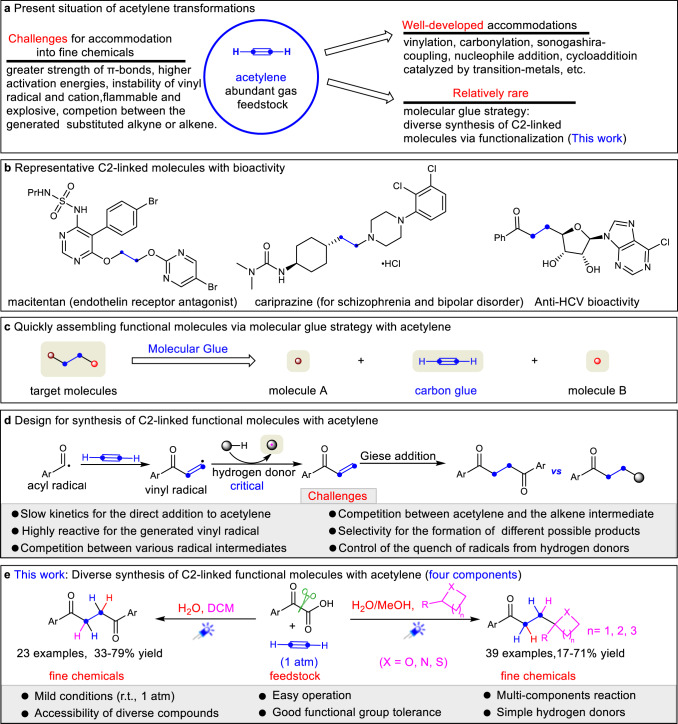
Synthesis of C2-linked molecules with acetylene via molecular glue strategy. **a** Present situation of acetylene transformations. **b** Representative C2-linked molecules with bioactivity. **c** Quickly assembling functional molecules via a molecular glue strategy with acetylene. **d** Design for synthesis of C2-linked molecules with acetylene. **e** Diverse synthesis of C2-linked molecules with acetylene. DCM, Dichloromethane.

As the simplest alkyne, acetylene has traditionally been viewed more as a fuel than as an economical chemical feedstock, though it is widely used in chemical industry, with an estimated annual goal production of over one million tons^1^. The high-volume industrial use of acetylene includes the production of vinyl-containing monomers, such as vinyl amine, vinyl chloride, acrylic acid and its derivatives, used for polymeric materials, in other chemical commodities and as feedstocks^2–4^. The spatial orthogonality of the two independent π-systems in alkynes can be used for the discovery, design and control of the new cascade transformations^5^. However, in contrast to other substituted alkynes, a very limited number of catalytic protocols directly incorporate acetylene into fine chemicals. This is probably due to the greater inherent strength of the π-bonds^5^, higher activation energies for reactions^5^ and, especially, apprehensions about handling an explosive^6^ and flammable gaseous reagent. In fact, reactions exploiting acetylene at atmospheric pressure (1 atm) are uncommon^1–4,7–14^. Moreover, the instability of its possible intermediates, the terminal vinyl radical^15,16^ and, especially, the cation^17^ has traditionally restricted its conversion. Circumvention of these obstacles would undoubtedly boost the use of acetylene as a reagent in modern organic synthesis involving complex small molecules. Currently, the catalytic transformations of acetylene into fine chemicals are mainly focused on simple monofunctionalizations, such as vinylation^2,3,7–10,13,14,18,19^, the Sonogashira coupling reaction^20,21^, and cyclization^2,22^. In addition, construction of other molecules with different levels of molecular complexity^11,12,21–23^ is of interest (Fig. 1a). To effectively introduce acetylene in synthesis, competing reactions, such as the generation of more reactive substituted alkynes or alkenes from acetylene, need to be avoided. Despite elegant synthetic developments, effective catalytic strategies to incorporate acetylene gas into fine chemicals remain scarce.

A variety of structurally complex and biologically active molecules or their precursors can be regarded as two components linked with a saturated two-carbon unit^24–26^ (Fig. 1b). Ideally, it would be desirable to bridge two or more simple molecules simply through their exposure to a C2-synthon in the presence of a catalyst. This would provide an efficient platform to quickly construct the target molecules and offer molecular diversity via the molecular glue strategy^27^. As the simplest and abundant unsaturated two-carbon molecular building units, ethylene^12,28,29^ and acetylene^2,3,7–12,18–23^ have been employed as C2 building blocks for the formation of fine chemicals. In fact, due to the presence of two addressable π-systems, acetylene can be used as a carbon glue^30^, a type of molecular glue, that connects molecules through sequential transformations of the two π-bonds, which mutually complements the ethylene transformation field^12,28,29,31,32^ (Fig. 1c). Given 1,4-diketones have great importance as versatile intermediates for the synthesis of some bioactive molecules^33^, natural products and related compounds^34,35^, along with the ubiquity of bioactive molecules containing a carbonyl group linked to a heterocycle moiety^26^ by a CH_2_-CH_2_ bridge, developing a general methodology to quickly access these compounds via a molecular glue strategy from readily available molecules and abundant feedstock would be highly desirable, especially in drug discovery chemistry^36^.

Photocatalysis has recently emerged as a powerful platform for the direct functionalization and activation of organic compounds via open-shell pathways under mild conditions^37–42^. As one of the most widely exploited transformations within the realm of open-shell chemistry, the direct addition of carbon radicals to carbon-carbon π-bonds has been broadly leveraged to effect carbon-carbon bond formation with alkenes^43,44^. Encouraged by these developments, we wondered if it is feasible to utilize the highly reactive terminal vinyl radical, generated through the acyl radical addition to acetylene, to accomplish the C-H abstraction from suitable hydrogen donors, followed by a Giese radical addition^45–49^, resulting in the formation of 1,4-diketones or connecting the carbonyl group to heterocycles via a two-carbon unit (Fig. 1d). However, the direct addition of most common radicals to unactivated alkynes, especially acetylene, to obtain the terminal vinyl radical, is a really big challenge due to (i) the diminished rate of C-C bond formation owing to increased activation barriers^15,50,51^ and (ii) the in situ generation of high-energy vinyl radical intermediates that are highly unstable, have a short lifetime, and readily participate in various undesirable open-shell pathways. To the best of our knowledge, direct addition to acetylene producing a terminal vinyl radical followed by functionalization is rarely reported^52^. In addition, the various radicals formed can react with the acetylene or the newly formed alkene generating undesired products. Importantly, how to selectively generate the desired product is also a big problem. Using the polarity matching effect, a subtle yet important element in radical addition process^53^, the generated product may be controlled by varying the electronic properties of the hydrogen donors. In addition, side reactions may be limited by quenching the radical intermediate formed by the C-H abstraction from the hydrogen donor.

In this work, we developed a photoredox-catalyzed synthetic protocol for diverse C2-linked molecules via a molecular glue strategy that employed gaseous acetylene under mild conditions (Fig. 1e). Formally, aryl ketones were linked to aryl ketones, or linked with heterocycles by CH_2_-CH_2_ bridges, resulting in the formation of two C-C bonds and two C-H bonds. Mechanistic experiments demonstrate that the two C-H bonds are created in two mechanistically distinct ways, one via a C-H abstraction and the other via protonation.

## Results and discussion

### Reaction development

To start our investigation, the synthetic method for 1,4-diketones was explored with the commercially available α-oxocarboxylic acid **1a** as the model substrate, acetylene gas as the C2-linker reagent, K_2_HPO_4_ as the base, H_2_O as the hydrogen source and Ir[dF(CF_3_)ppy]_2_(dtbpy)PF_6_ as the visible-light photocatalyst at room temperature under the irradiation of blue LEDs. From a mechanistic perspective, the highly reactive vinyl radical should abstract a hydrogen atom from a solvent C*sp*^*3*^-H bond because of the C*sp*^*2*^-H’s higher bond dissociation energy (BDE)^54,55^. To our delight, the reaction in a solution of DCM/H_2_O (*v/v*) (3/2) afforded the desired 1,4-diketone **2a** in 10% yield (Table 1, entry 1). Examination of a range of photocatalysts revealed that Ir[dF(CF_3_)ppy]_2_(phen)PF_6_ was superior with respect to reaction efficiency, yielding **2a** in 16% yield (entries 2-4). Various solvents, such as acetone, *N*,*N*-dimethylformamide, acetonitrile, and tetrahydrofuran, were used instead of dichloromethane (entries 5-8) and the results indicated dichloromethane was more suitable for this transformation. The concentration of **1a** and the amount of water may affect the reaction efficiency. When **1a** was decreased to 0.05 M, 38% isolated yield of **2a** was obtained (H_2_O (5 equiv.), 24 h) (entry 9). Prolonging the reaction time (36 h) did not improve the yield of **2a** (entry 10). Further reducing **1a** to 0.025 M improved the yield to 51% (entry 11). A slight decrease in the yield was observed when the concentration was further reduced (entry 12). Using other inorganic bases did not positively affect the reaction efficiency (entries 13-15). Gratifyingly, the yield of **2a** could be greatly improved to 79% by increasing H_2_O (20 equiv.) (entry 16). Further increasing the amount of water decreased the yield (entry 17), which means the amount of water has an important effect on the yield.

**Table 1:** Optimization of the Reaction Conditions.


Entry	PC	Base	Solvent	Yield (%)^*a*^
1	Ir[dF(CF_3_)ppy]_2_(dtbbpy)PF_6_	K_2_HPO_4_	DCM/H_2_O (3/2) (0.1 M)	10%
2	4CzIPN	K_2_HPO_4_	DCM/H_2_O (3/2) (0.1 M)	0
3	Eosin Y	K_2_HPO_4_	DCM/H_2_O (3/2) (0.1 M)	0
4	Ir[dF(CF_3_)ppy]_2_(phen)PF_6_	K_2_HPO_4_	DCM/H_2_O (3/2) (0.1 M)	16
5	Ir[dF(CF_3_)ppy]_2_(phen)PF_6_	K_2_HPO_4_	Acetone/H_2_O (3/2) (0.1 M)	0
6	Ir[dF(CF_3_)ppy]_2_(phen)PF_6_	K_2_HPO_4_	DMF/H_2_O (3/2) (0.1 M)	0
7	Ir[dF(CF_3_)ppy]_2_(phen)PF_6_	K_2_HPO_4_	MeCN/H_2_O (3/2) (0.1 M)	<10
8	Ir[dF(CF_3_)ppy]_2_(phen)PF_6_	K_2_HPO_4_	THF/H_2_O (3/2) (0.1 M)	<5
9^*b*^	Ir[dF(CF_3_)ppy]_2_(phen)PF_6_	K_2_HPO_4_	DCM (0.05 M)	38
10^*c*^	Ir[dF(CF_3_)ppy]_2_(phen)PF_6_	K_2_HPO_4_	DCM (0.05 M)	34
11^*b*^	Ir[dF(CF_3_)ppy]_2_(phen)PF_6_	K_2_HPO_4_	DCM (0.025 M)	51
12^b^	Ir[dF(CF_3_)ppy]_2_(phen)PF_6_	K_2_HPO_4_	DCM (0.017 M)	44
13^*b*^	Ir[dF(CF_3_)ppy]_2_(phen)PF_6_	K_2_CO_3_	DCM (0.025 M)	26
14^*b*^	Ir[dF(CF_3_)ppy]_2_(phen)PF_6_	KF	DCM (0.025 M)	49
15^*b*^	Ir[dF(CF_3_)ppy]_2_(phen)PF_6_	K_3_PO_4_	DCM (0.025 M)	19
16^*d*^	Ir[dF(CF_3_)ppy]_2_(phen)PF_6_	K_2_HPO_4_	DCM (0.025 M)	79
17^*e*^	Ir[dF(CF_3_)ppy]_2_(phen)PF_6_	K_2_HPO_4_	DCM (0.025 M)	49


^a^**1a** (0.3 mmol). Yields of **2a** were determined by ^1^H NMR with mesitylene as an internal standard. ^b^H_2_O (5 equiv.), 24 h, isolated yield. ^c^H_2_O (5 equiv.), 36 h, isolated yield. ^d^H_2_O (20 equiv.), 24 h, isolated yield. ^e^H_2_O (40 equiv.), 24 h, isolated yield. dF(CF_3_)ppy, 3,5-difluoro-2-[5-(trifluoromethyl)-2-pyridinyl]phenyl; phen, *o*-Phenanthroline; DCM, Dichloromethane; equiv., equivalent; PC, photocatalyst.

### Reaction scope investigation

**Fig. 2:**
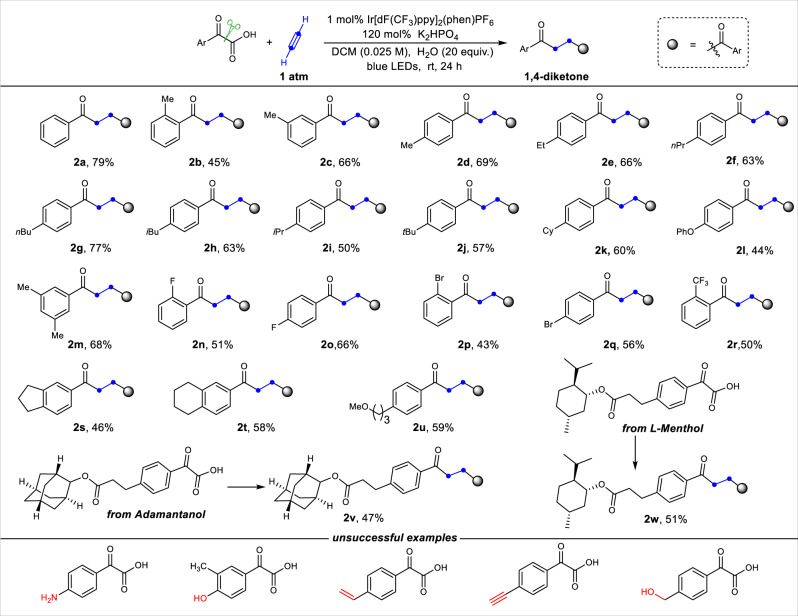
Substrate scope for 1,4-diketones synthesis. Standard conditions: α-Oxocarboxylic Acid (0.3 mmol), Ir[dF(CF_3_)ppy]_2_(Phen)PF_6_ (0.01 equiv.), H_2_O (20 equiv.) in a solution of DCM (0.025 M) under the irradiation of blue LEDs under acetylene gas for 24 h at room temperature. Isolated yield of products. dF(CF_3_)ppy, 3,5-difluoro-2-[5-(trifluoromethyl)-2-pyridinyl]phenyl; phen, *o*-Phenanthroline; DCM, Dichloromethane; equiv., equivalent.

With the optimal reaction conditions in hand (Table 1, entry 16), we next investigated the scope of a variety of α-oxocarboxylic acids summarized in Fig. 2. The *o*-, *m*- and *p*-methyl groups on phenyl α-oxocarboxylic acids were tolerated (**2b-d**). A number of phenyl α-oxocarboxylic acids bearing electron-donating groups, including not only 1°-alkyl groups but also more hindered 2°- and 3°-alkyl groups, were converted into the corresponding 1,4-diketones in good yields (**2e-l**). The 3,5-dimethyl substituted substrates was transformed into the desired compound in 68% yield (**2m**). Halogenated phenyl substrates were incorporated into the corresponding compounds with slightly lower yields (43-66%) (**2n-q**). The phenyl α-oxocarboxylic acid bearing a strong electron-withdrawing group underwent smoothly to afford **2r** in 50% yield. Notably, 2-(2,3-dihydro-1*H*-inden-5-yl)-2-oxoacetic acid **1s** and 2-oxo-2-(5,6,7,8- tetrahydronaphthalen-2-yl) acetic acid **1t** provided the corresponding **2s** and **2t** in moderate yields. Importantly, the methoxy-substituted long chain substrate **1u** was also suitable for this transformation giving **2u** in moderate yield. Moreover, the 2-adamantanol-derived α-oxocarboxylic acid **1v** and L-menthol-derived α-oxocarboxylic acid **1w** reacted smoothly under the standard conditions to furnish the corresponding desired compounds **2v** and **2w** in moderate yields. These results show a great potential for the structural modification of an array of complex biological molecules in medicinal chemistry. Notably, various experiments with substrates containing redox non-innocent substituents, such as amino-, phenolic hydroxyl-, vinyl-, alkynyl- and hydroxyl-substituted, were also performed under the standard conditions. Unfortunately, these functional groups were not compatible with our system.

**Fig. 3:**
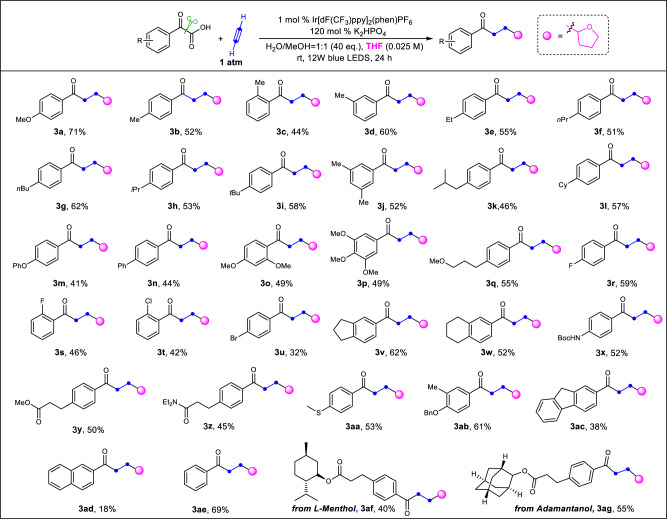
Substrate scope for synthesis of tetrahydrofuran-containing molecules with acetylene. Standard conditions: α-Oxocarboxylic Acid (0.3 mmol), Ir[dF(CF_3_)ppy]_2_(Phen)PF_6_ (0.01 equiv.), H_2_O (20 equiv.), MeOH (20 equiv.) in a solution of THF (0.025 M) under the irradiation of blue LEDs under acetylene gas balloon for 24 h at room temperature. Isolated yield of products. dF(CF_3_)ppy, 3,5-difluoro-2-[5-(trifluoromethyl)-2-pyridinyl]phenyl; phen, o-Phenanthroline; THF, tetrahydrofuran.

Considering these results, we turned our attention to test other hydrogen donors, specifically nucleophilic hydrogen donors. These donors would generate radical intermediates from C-H abstractions by the highly reactive vinyl radicals. The resulting C-H abstracted radical intermediate reacted well with the electron-deficient alkenes^44–48^ to furnish the desired compounds containing the carbonyl group and nucleophilic component linked via a CH_2_-CH_2_ bridge. In 2016, Knowles^56^ reported an elegant intermolecular C-H functionalization to construct similar molecules in moderate yields using stoichiometric *N*-ethyl-4-methoxybenzamide as the abstractor. This abstractor has the potential to serve as a structurally modular catalyst for radical C-H functionalization. Though powerful, the preparation of the vinyl ketone and additional reagents to activate the substrate were required for the reported method. If the vinyl radical generated in our system could be used as the abstractor, it would be highly desirable. Due to the prevalence of furan-containing compounds^57,58^, THF was subsequently explored as a hydrogen donor and furan source, as well as the solvent. In fact, 13% yield of the tetrahydrofuran linked compound was isolated during the optimization (Table 1, entry 8). A considerable increase in yield was obtained with a slight variation of the reaction conditions (Fig. 3). With respect to the 2-aryl-2-oxocarboxylic acid partner, we observed moderate to good yields of the desired products, which represent an important skeleton in a variety of bioactive molecules^11^. 2-Oxo-2-phenyl-acetic acids bearing both electron-donating and electron-withdrawing substituents on the phenyl ring are suitable substrates (**3a-3ab**). Relatively lower yields were observed when an electron-withdrawing group was attached to the phenyl ring (**3r-u**), which could be ascribed to the reduced reductive quenching ability toward the photoexcited photocatalyst. Notably, 2-aryl-2-oxocarboxylic acids with synthetic handles, such as halides, were readily incorporated into the products (**3t-u**), which highlights the potential for the incorporation of these scaffolds into more complex targets. Evaluation of substrates containing reactive groups, such as the chemically and biologically abundant amide and ester, provided the corresponding products in moderate yield (**3x-3z**). The starting material, bearing an easily-oxidized thioether, is also tolerated in this system, furnishing the desired product **3aa** in moderate yield. In addition, 2-naphthyl- and 1-fluorene-substituted glyoxylic acids are both suitable substrates, albeit in slightly lower yield (**3ac-3ad**). The reactive benzylic C-H bonds in the starting material, or in the corresponding product **3ad**, remain intact under the reaction conditions. The simple benzoylformic acid was transformed to the corresponding product **3ae** in 69% yield. Moreover, the menthol-derived α-oxocarboxylic acid and 2-adamantanol-derived α-oxocarboxylic acid reacted smoothly under the standard conditions to furnish the desired compounds **3af** and **3ag** in moderate yield. These results show the potential for the structural modification of an array of bioactive molecules.

**Fig. 4:**
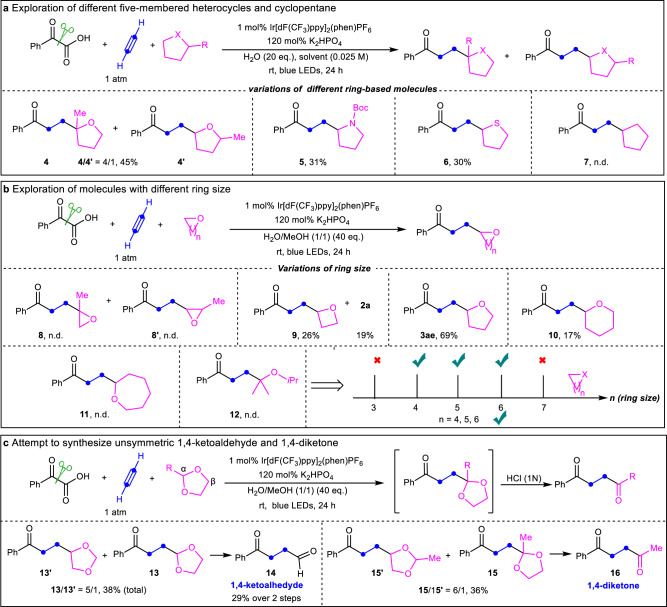
Exploration of different cyclic molecules with acetylene. **a** Exploration of different five-membered heterocycles and cyclopentane. 2-Me-THF was used as solvent for **4** and **4’**. *N*-Boc pyrrolidine (5 equivalent) and 1,2-dichloroethane (solvent) were used for **5**. Tetrahydrothiophene (5 equivalent) and acetonitrile (solvent) were used for **6**. Cyclopentane was used as solvent for **7**. dF(CF_3_)ppy, 3,5-difluoro-2-[5-(trifluoromethyl)-2-pyridinyl]phenyl; phen, o-Phenanthroline; DCE, 1,2-Dichloroethane; eq, equivalent; 2-Me THF, 2-Methyltetrahydrofuran. **b** Exploration of molecules with different ring size. All the heterocycles were used as solvent or cosolvent, see supplementary information for details. **c** Attempt to synthesize unsymmetric 1,4-ketoaldehyde and 1,4-diketone. The heterocycles were used as solvent.

Considering the experimental results above and the requirements for construction of other heterocycle-containing compounds as well as the extension of this approach, some hydrocarbons, especially, heterocycles instead of tetrahydrofuran were subsequently tested (Fig. 4). It was found that this strategy could also be adapted for use with other five-membered heterocycle-based hydrogen donors (Fig. 4a). When substituted tetrahydrofuran was examined, the regioisomers **4** and **4**′ were provided in moderate yield with a 4/1 ratio. *N*-Boc pyrrolidine reacted smoothly to furnish the corresponding compound **5** in 31% yield. Moreover, tetrahydrothiophene provided the desired compound **6** under similar conditions in 30% yield. These results suggest that the weak C-H bonds adjacent to heteroatoms in the five-membered heterocycles could be directly functionalized with acetylene using our system. However, the less reactive cyclopentane (a five-membered carbocycle) could not serve as the hydrogen donor to provide the desired product **7**. In addition to five-membered heterocycles, we also examined the feasibility of other heterocycles with different ring sizes (Fig. 4b). The experiments showed that the three-membered cyclic ether, 1,2-epoxypropane, could not be glued with acetylene to the carbonyl group for the formation of **8** or **8**′, which might be attributed to the epoxides easily opened ring. To our delight, the four-membered cyclic ether, oxetane, was successfully connected with acetylene, furnishing the desired ketone **9** in 26% yield, accompanied by 19% yield of **2a**. As shown in Fig. 3, THF, a five-membered cyclic ether, gave a good result with 69% yield of **3ae**. When the ring size was further increased from five to six, the corresponding ketone **10** tethering with tetrahydropyran (six-membered cyclic ether) could be isolated as well, but with a lower yield (17%). However, no desired product **11** could be detected when the seven-membered cyclic ether, oxepane, was used as the substrate. It seems that the larger membered heterocycles (over six members) are not suitable partners. Linear ether (isopropyl ether) failed to provide the desired product **12**, which is in line with the trend demonstrated in Fig. 4b. Interestingly, connecting 1,3-dioxolane with the carbonyl group was possible, giving regioisomers **13** and **13**′ in a reasonable yield with a 5/1 ratio (Fig. 4c). The observed regioselectivity of H-abstraction in 1,3-dioxolane may be attributed to the anomeric lowering of the C-H BDE^59^ (BDE_α(C-H)_ = 86.8 Kcal/mol *vs* BDE_β(C-H)_ = 88.2 Kcal/mol^60^). After deprotection, 1,4-ketoaldehyde **14** was obtained efficiently from the crude regioisomers. Similar regioisomers **15** and **15**′, which are an ideal precursor of unsymmetric 1,4-diketones, were also obtained by switching from 1,3-dioxolane to 2-methyl-1,3-dioxolane. These outcomes illustrate that unsymmetric 1,4-dicarbonyl compounds could be accessed by using acetylene as a carbon glue, which greatly expands the synthetic scope of 1,4-dicarbonyl compounds. Compared to Knowles’ strategy^56^, our protocol utilized the vinyl radical generated in situ, to activate the weak C*sp*^*3*^-H bond of heterocycles avoiding the need for the additional abstractor and the preparation of the corresponding vinyl ketones as substrates. Our strategy provides alternative access to this type of compound from abundant feedstock under mild conditions.

**Fig. 5:**
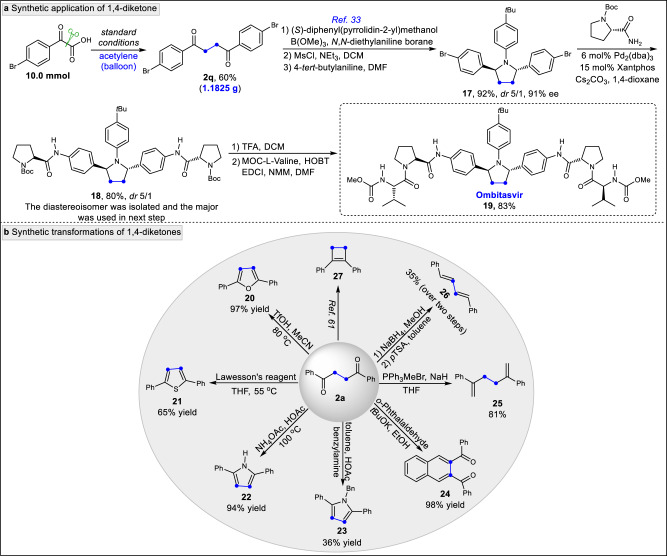
Application potentials in the syntheses of bioactive molecules and transformations. **a** The 1,4-diketone **2q** could be prepared in gram-scale with no decrease in isolated yield. The approved drug Ombitasvir could be efficiently constructed with our strategy. DCM, Dichloromethane. MsCl, Methanesulfonyl chloride. DMF, *N,N*-Dimethylformanide. TFA, Trifluoroacetic acid. HOBT, 1-Hydroxybenzotriazole hydrate. EDCI, *N*-(3-dimethylaminopropyl)-*N*’-ethylcarbodiimide hydrochloride. NMM, *N*-methylmorpholine. **b** diverse high-valued compounds could be prepared from 1,4-diketone **2a**.

To illustrate the synthetic utility of our strategy, a series of experiments were conducted (Fig. 5). On a preparative scale, 1,4-diketone **2q** was isolated in 60% yield, which was slightly higher than that of the 0.3 mmol scale, suggesting that large-scale production might be feasible. Synthesis of enantiomerically enriched Ombitasvir **19**^33^, an orally bioavailable and potent inhibitor, was achieved using chiral **18** which was prepared in 4 steps from **2q** via sequential asymmetric reduction, methanesulfonation, nucleophilic cyclization^33^ and coupling steps (73% yield for 4 steps, *dr* 5/1) (Fig. 5a). Moreover, simple transformations of the 1,4-dicarbonyl moiety afforded a range of synthetically useful scaffolds (Fig. 5b). For instance, heterocyclic molecules, such as substituted furan **20**, substituted thiophene **21** and substituted pyrroles **22**-**23** were obtained via the cyclization of **2a** under acidic condition or in the presence of Lawesson’s reagent, ammonium acetate or benzylamine. The 1,4-dicarbonyl compound was easily transformed to naphthalene-2,3-diylbis(phenylmethanone) **24** in 98% yield under basic conditions with *o*-phthalaldehyde. Additionally, alkene **25** was accessed through a Wittig reaction with methyltriphenylphosphonium bromide. The derived 1,3-butadiene **26** was also obtained through a sequential reduction and elimination processes. Furthermore, the 1,4-dicarbonyl compound **2a** was transformed to cyclobutene **27** in good yield according to the known literature^61^.

### Mechanistic studies

**Fig. 6:**
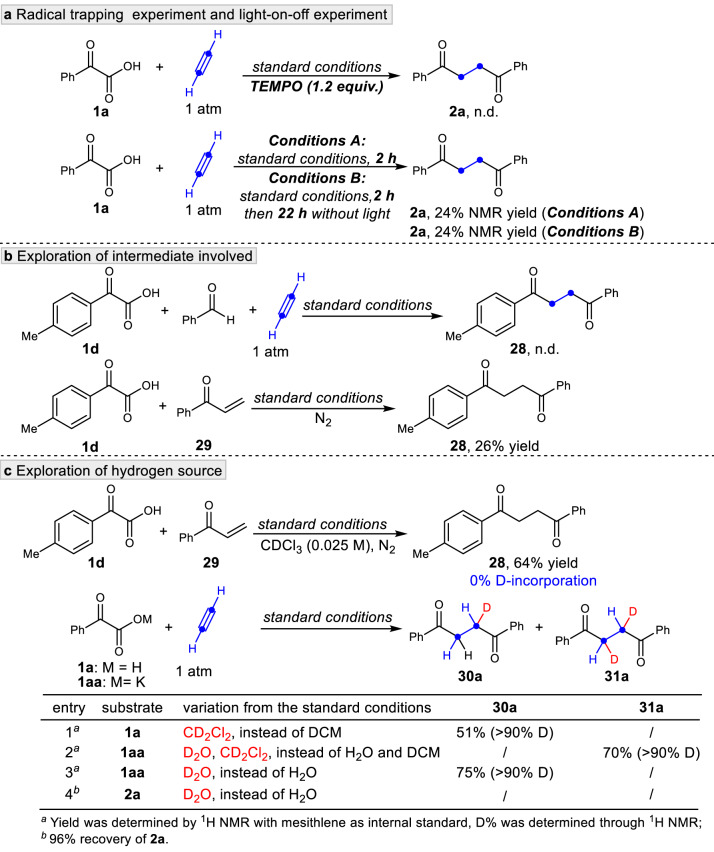
Mechanistic studies. **a** Radical trapping and light-on-off experiment were performed, suggesting this transformation might proceed via a radical pathway. TEMPO, 2,2,6,6-tetramethylpiperidinooxy. **b** Exploration of intermediate involved. n.d., not detected. **c** Exploration of hydrogen source. Both the solvent and water are the hydrogen source of this transformation. DCM, Dichloromethane.

To further gain mechanistic insights, control experiments were performed (Fig. 6). In the presence of the radical trap TEMPO, the reaction completely shut down (Fig. 6a, top), indicating that a radical intermediate might be involved in this transformation. The reaction of **1a** was performed for 2 h under standard conditions, affording **2a** in 24% yield (Fig. 6a, bottom). Additionally, no change in the yield of **2a** was observed when the same reaction was conducted for 2 h and then for an additional 22 h without light (Fig. 6a, bottom). These experiments indicate that the reaction is a visible-light photocatalysis process. Considering benzaldehyde was detected as a by-product during the reaction condition optimizations, we explored the reaction of **1d** and benzaldehyde under the standard conditions (Fig. 6b, top) to verify whether benzaldehyde was the reaction intermediate. No cross-over product **28** was observed, strongly suggesting that benzaldehyde is not likely an intermediate of this transformation. According to our initial assumption and the inherent reactivity of the vinyl radical, direct abstraction of a hydrogen atom from hydrogen donors to produce the vinyl phenyl ketone is reasonable. As a result, the reaction of stoichiometric vinyl phenyl ketone **29** and 2-(4-methylphenyl)-2-oxoacetic acid **1d** was performed under the standard conditions without acetylene, affording the expected product **28** in 26% yield (Fig. 6b, bottom). The result showed that the corresponding vinyl phenyl ketone is the key intermediate in our system. To get further insight into the hydrogen source, chloroform-*d* was used as the solvent instead of dichloromethane in the reaction of vinyl phenyl ketone **29** and 2-(4-methylphenyl)-2-oxoacetic acid **1d** (Fig. 6c, top) and furnished **28** in 64% yield with 0% D-incorporation in the α-position of the carbonyl group according to ^1^H NMR analysis. In sharp contrast to this phenomenon, > 90% D was incorporated into the molecule **30a** when dichloromethane-*d2* was used in the model reaction (Fig. 6c, entry 1), indicating one of the hydrogens next to the carbonyl group comes from the solvent. When the corresponding potassium salt of benzoylformic acid **1aa** was tested under the standard conditions using dichloromethane-*d2* as the solvent and D_2_O as an additive, the corresponding product **31a** bearing > 90% D-incorporation in each methylene site was isolated without **30a** being detected (Fig. 6c, entry 2). Additionally, > 90% D-incorporation was verified in one of the methylene sites using D_2_O as the additive (Fig. 6c, entry 3), meaning another hydrogen adjacent to the carbonyl group originates from water. To further exclude that the deuterated product was generated from **2a** under standard conditions through H/D exchange with D_2_O, **2a** was subjected to reaction conditions with D_2_O instead of H_2_O (Figure 6c, entry 4). Interestingly, no deuterated products **30a** or **31a** were found with the 96% recovery of **2a**. This experiment suggested that the CH_2_ of **2a** cannot be deuterated through H/D exchange with D_2_O under standard conditions. Taken together, these results demonstrated the water and solvent are both hydrogen sources but used in the different stages of the reaction process. This is further supported by the mechanistic experiments for **3ae** (see Supplementary Fig. 6 and Supplementary Fig. 7).

### Mechanistic proposal

**Fig. 7:**
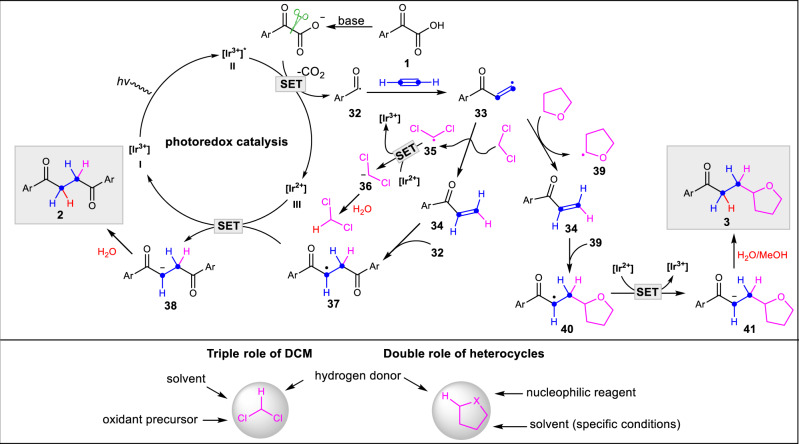
A plausible mechanism. The catalytic cycle begins with oxidative single electron transfer (SET), generating the acyl radical **32**. The intermediate then undergoes addition to acetylene to generate vinyl radical **33**. Intermolecular hydrogen atom transfer (HAT) then occurs to afford aryl vinyl ketone **34** accompanied by the generation of **35** or **39**. At last, the Giese radical reaction between aryl vinyl ketone and another acyl radical **32** or **39** occur to furnish the final product.

Based on the previously reported literature^54,55,62–66^ and the aforementioned mechanistic studies, a plausible mechanism for this transformation is proposed in Fig. 7. Upon irradiation with visible light, the photocatalyst Ir[dF(CF_3_)ppy]_2_(phen)PF_6_ **I** is known to access the highly oxidizing excited state (ES) species **II (**[Ir^3+^]*), Which could be reductively quenched by the anion of α-oxocarboxylic acid **1** to generate an acyl radical **32**^62,65^ through a decarboxylative pathway, and the reduced species **III**. The generated **32** could be captured by acetylene gas to afford vinyl radical species **33** which is highly unstable and quickly abstracts a hydrogen from dichloromethane to provide the electron-deficient vinyl ketone **34** accompanied by the generation of **35**. This proposed process is in line with the fact that the BDE of the C*sp*^*2*^-H bond (~110 kcal/mol)^54,55^ exceeds that of C*sp*^*3*^-H bond of dichloromethane (~95 Kcal/mol)^66^. The reduced species **III** could be oxidized to regenerate the photocatalyst **I** by **35**, furnishing the carbon anion **36** which could be protonated to regenerate dichloromethane. Subsequently a Giese radical addition reaction occurs with another molecule of acyl radical **32**, which adds quickly to alkene **34** to produce a new carbon radical **37**. Then a single-electron reduction of radical **37** by **III** affords the carbon anion **38** and regenerates the photocatalyst **I**. The desired product is obtained after protonation of **38**. The phenomenon that nearly no electron-deficient alkene **34** was observed could be attributed to the fact that **34** is more highly reactive than the parent acetylene. Similarly, vinyl radical species **33** could also abstract hydrogen from heterocycles bearing weaker C-H bonds, affording alkene **34** and nucleophilic radical intermediate **39** which is rapidly captured by **34** to produce **40**. Then the sequential single electron transfer (SET)/protonation process occurs to provide the final product **3**. It is noteworthy that dichloromethane functions as solvent, as hydrogen donor, and as oxidant precursor to facilitate the regeneration of the reduced photocatalyst. In addition to serving as hydrogen donors and solvents (special conditions), heterocycles also work as the nucleophilic components due to the higher nucleophilicity of their corresponding radicals over that of **35**.

In summary, a diverse synthesis protocol for C2-linked functionalized molecules with gaseous acetylene was developed, which provided an efficient method to quickly access a variety of compounds through connecting two components together via a molecular glue strategy with readily available substrates and abundant acetylene gas under mild conditions. A series of 1,4-diketones, which are important precursors of an array of heterocycles, were quickly constructed. Importantly, the reaction system was expanded to construct heterocycle-containing compounds. These compounds form when the intermediate vinyl ketone is intercepted by a radical intermediate formed from a heterocycle C-H abstraction. In both of these transformations, acetylene is incorporated in the final product as a CH_2_-CH_2_ bridge. Additionally, mechanistic studies demonstrated that one of the formed C-H bonds is created via a C-H abstraction from the hydrogen donor, such as dichloromethane or a heterocycle, and the other via protonation from water or methanol. Moreover, this approach provides ready access to interesting, functionalized molecules and expands the utility of acetylene in organic synthesis, which will inspire new perspectives for value-added chemical synthesis using acetylene and promote the renaissance of catalytic transformations for acetylene.

## Methods

### Materials

Unless otherwise noted, all the materials were obtained commercially and used without further purification. All the solvents were treated according to general methods. Flash column chromatography was performed over silica gel (300-400 mesh). See Supplementary Methods for experimental details.

### General procedure A for the synthesis of 1,4-dicarbonyl compounds

To an oven-dried 25 mL flask, Ir[dF(CF_3_)ppy]_2_(phen)PF_6_ (0.003 mmol), 2-aryl-2-oxocarboxylic acid (0.3 mmol), K_2_HPO_4_ (0.36 mmol), H_2_O (20 equiv.) and DCM (12 mL) were added sequentially under N_2_. The flask was degassed through three freeze-pump-thaw cycles under acetylene and then an acetylene gas balloon was attached through a long syringe needle. The reaction mixture was irradiated by 12 W blue LEDs at a distance of 5 cm for 24 h at room temperature. The reaction mixture was filtered through a short pad of silica using ethyl acetate. The filtrate was concentrated in *vacuo* before it was purified by flash chromatography on silica gel to afford the desired product.

### General procedure B for the synthesis of heterocycle-containing compounds

To an oven-dried 25 mL flask, Ir[dF(CF_3_)ppy]_2_(phen)PF_6_ (0.003 mmol), 2-aryl-2-oxocarboxylic acid (0.3 mmol), K_2_HPO_4_ (0.36 mmol), H_2_O/MeOH (1/1, 40 equiv.) and THF (12 mL) were added sequentially under N_2_. The flask was degassed through three freeze-pump-thaw cycles under acetylene and then an acetylene gas balloon was attached through a long syringe needle. The reaction mixture was irradiated by 12 W blue LEDs at a distance of 5 cm for 24 h at room temperature. The reaction mixture was filtered through a short pad of silica using ethyl acetate. The filtrate was concentrated in *vacuo* before it was purified by flash chromatography on silica gel to afford the desired product.

## Supplementary information

Supplementary Information

## Data Availability

The authors declare that data relating to the characterization of materials and products, general methods, optimization studies, experimental procedures, mechanistic studies, HRMS data and NMR spectra are available within the article and the Supplementary Information.
