# Supplementary material for: Diverse synthesis of C2-linked functionalized molecules via molecular glue strategy with acetylene
Source: Nat Commun. 2022 Apr 6;13:1858. doi: 10.1038/s41467-022-29556-2 (PMC8986794; doi:10.1038/s41467-022-29556-2)
Supplement: Supplementary file 1 — Supplementary Information [file 41467_2022_29556_MOESM1_ESM.pdf]

SUPPLEMENTARY INFORMATION

Diverse Synthesis of C2-Linked Functionalized Molecules via Molecular Glue Strategy with Acetylene

Bo Yang,[1,2] Shaodong Lu,[2] Yongdong Wang,[2] Shifa Zhu[1,*]

[1]Key Laboratory of Functional Molecular Engineering of Guangdong Province, School of Chemistry and Chemical Engineering, South China University of Technology, Guangzhou 510640, China.

[2]Singfar Laboratories, Guangzhou, 510670, China.

*email: zhusf@scut.edu.cn

Table of Contents

I. Supplementary Methods 2

II. Supplementary Discussion 46

III. Supplementary NMR Spectra and HPLC Spectra 57

IV. Supplementary References 141

I. Supplementary Methods

General Information

THF and toluene were distilled from sodium benzophenone ketyl prior to use. DCM was distilled from calcium hydride. Unless otherwise noted, all the corresponding ketones from suppliers were used directly without further purification. The $Ir[dF(CF_3)ppy]_2(phen)PF_6$ was prepared according to the literature.[1] NMR spectra were recorded on a Bruker-500 instrument. ^1H NMR chemical shifts were referenced to the tetramethylsilane (0 ppm); ^{13}C NMR chemical shifts were referenced to the solvent resonance (77.00 ppm, $CDCl_3$). The following abbreviations (or combinations thereof) were used to explain multiplicities: s = singlet, d = doublet, t = triplet, m = multiplet, q = quadruplet. IR spectra were recorded on a Nicolet 210 spectrophotometer and were recorded in potassium bromide (KBr) pellet. High-resolution mass spectra (HRMS) were recorded on ESI-TOF (electrospray ionization-time of flight). Melting points were determined using a hot stage apparatus. All manipulations were conducted under Schlenk tubes. All reagents were used as received from commercial sources, unless specified otherwise, or prepared as described in the literature.

Optimization of Reaction Conditions

Supplementary Table 1. Optimization of Photocatalysts[a]

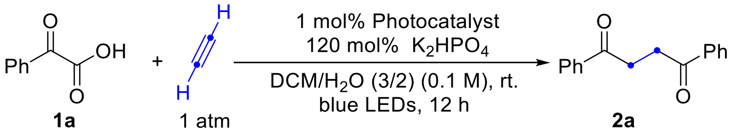

Entry	Photocatalyst	Yield (%)[b]
1	$Ir[dF(CF_3)ppy]_2(dtbbpy)PF_6$	<10
2	4CzIPN	0
3	$Ir(ppy)_2(dtbbpy)PF_6$	0
4	Eosin Y	0
5	$Ir[dF(CF_3)ppy]_2(phen)PF_6$	13

[a] Standard conditions: 0.3 mmol of α-Oxocarboxylic Acid, 1 mol% of photocatalyst, K_2HPO_4 (120 mol%), DCM/ H_2O (3/2) (0.1 M), under the irradiation of 12 W blue LEDs under acetylene gas atmosphere for 12 h at room temperature.

[b] Yields were determined by ^1H NMR with mesitylene as an internal standard.

Supplementary Table 2. Optimization of Solvents[a]

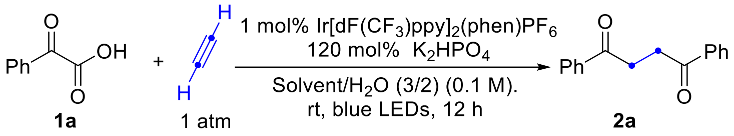

Entry	Solvent	Yield (%)[b]
1	MeCN	10
2	DCM	16
3	DMF	0
4	Acetone	0
5	THF	<5
6	Toluene	6
7	EA	8

[a] Standard conditions: 0.3 mmol of α-Oxocarboxylic Acid, 1 mol% of Ir[dF(CF_3)ppy]$_2$(Phen)PF_6, K_2HPO_4 (120 mol%), solvent/ H_2O (3/2) (0.1 M), under the irradiation of 12 W blue LEDs under acetylene gas atmosphere for 12 h at room temperature.

[b] Yields were determined by ^{1}H NMR with mesitylene as an internal standard.

Supplementary Table 3. Optimization of Reaction time[a]

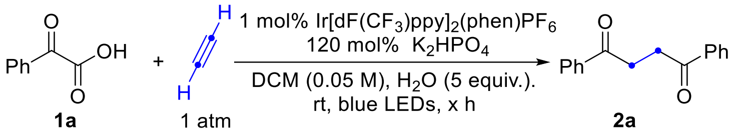

Entry	X/ h	Yield (%)[b]
1	24	38
2	36	34

[a] Standard conditions: 0.3 mmol of α-Oxocarboxylic Acid, 1 mol% of Ir[dF(CF_3)ppy]$_2$(Phen)PF_6, K_2HPO_4 (120 mol%), H_2O (5 equiv.), DCM (6 mL) under the irradiation of 12 W blue LEDs under acetylene gas atmosphere at room temperature.

[b] Isolated yields.

Supplementary Table 4. Optimization of Reaction concentration[a]

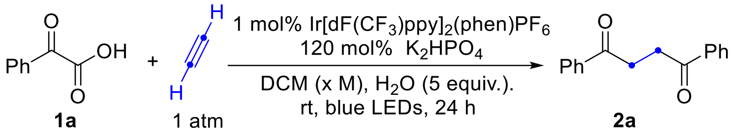

**Entry**	**x**	**Yield (%)[b]**
**1**	0.05	38
**2**	0.025	51
**3**	0.017	44

[a] Standard conditions: 0.3 mmol of α-Oxocarboxylic Acid, 1 mol% of Ir[dF(CF_3)ppy]$_2$(Phen)PF_6, K_2HPO_4 (120 mol%), H_2O (5 equiv.), DCM (X M) under the irradiation of 12 W blue LEDs for 24 h under acetylene gas atmosphere at room temperature.

[b] Isolated yields.

Supplementary Table 5. Optimization of various bases[a]

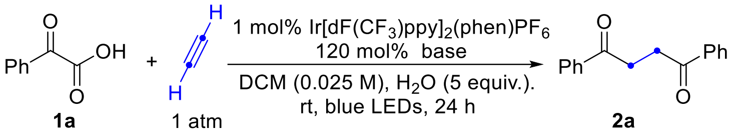

**Entry**	**base**	**Yield[b]**
**1**	K_2CO_3	26
**2**	Cs_2CO_3	26
**3**	KF	49
**4**	K_3PO_4	19
**5**	$KHCO_3$	22
**6**	$NaHCO_3$	22
**7**	K_2HPO_4	51
**8[c]**	K_2HPO_4	31
**9[d]**	K_2HPO_4	27

[a]Standard conditions: 0.3 mmol of α-Oxocarboxylic Acid, 1 mol% of Ir[dF(CF_3)ppy]$_2$(Phen)PF_6, H_2O (5 equiv.), DCM (0.025 M) and 120 mol% base under the irradiation of 12 W blue LEDs for 24 h under acetylene gas atmosphere at room temperature. [b]Isolated yields. [c]50 mol% K_2HPO_4 [d]200 mol% K_2HPO_4

Supplementary Table 6. Optimization of the amount of the water[a]

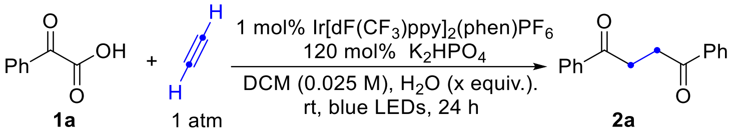

**Entry**	**X**	**Yield (%)[b]**
**1**	5	51
**2**	10	48
**3**	20	79
**4**	40	49
**5**	60	49

[a]Standard conditions: 0.3 mmol of α-Oxocarboxylic Acid, 1 mol% of Ir[dF(CF_3)ppy]$_2$(Phen)PF_6, H_2O (X equiv.), K_2HPO_4 (120 mol%), DCM (0.025 M) and 120 mol% K_2HPO_4 under the irradiation of 12 W blue LEDs for 24 h under acetylene gas atmosphere at room temperature. [b]Isolated yields.

Preparation of Substrates

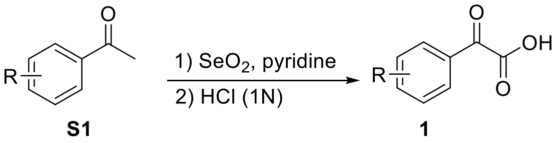

All the α-oxocarboxylic acids were prepared from oxidation of corresponding methyl ketones by SeO_2 according to the reported procedure unless noted. (Reference: Kuldeep, W.; Yang, C.; West, P. R.; Deming, K. C.; Chemburkar, S.; Reddy, R. R. E. *Synth Commun*. **2008**, *38*, 4434.).

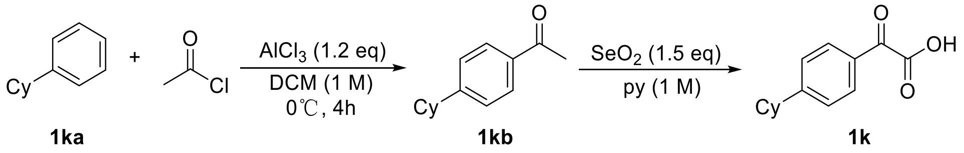

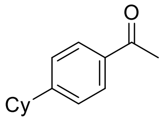

1kb

1-(4-Cyclohexylphenyl)ethan-1-one (1kb).[2] To an oven-dried flask cooled under N_2, **1ka** (2.4 g, 15.0 mmol), $AlCl_3$ (2.4 g, 18 mmol) and CCl_4 (15 mL) were added. Acetyl chloride (1.28 mL, 18.0 mmol) was added slowly at 0 °C. The reaction mixture was stirred at 0 °C to rt overnight. The reaction was quenched by HCl (1N) at 0 °C, extracted by DCM and dried over anhydrous Na_2SO_4. After filtration, the filtrate was condensed and purified by column chromatography (PE/EA = 20:1) through silica gel to obtain **1kb** (2.9 g, 14.3 mol, 95% yield) as a white solid. ^{1}H NMR (500 MHz, $CDCl_3$) δ 7.88 (d, J = 8.5 Hz, 2H), 7.28 (d, J = 8.5 Hz, 2H), 2.62-2.50 (m, 4H), 1.94-1.70 (m, 5H), 1.50-1.19 (m, 5H).

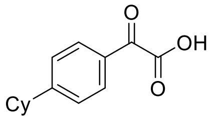

1k

2-(4-Cyclohexylphenyl)-2-oxoacetic acid (1k).[3] Then **1kb** was oxidized by SeO_2 according to the general procedure to afford **1k.** ^{1}H NMR (500 MHz, $CDCl_3$) δ 9.59 (s, 1H), 8.18 (d, J = 8.0 Hz, 2H), 7.35 (d, J = 8.5 Hz, 2H), 2.68-2.50 (m, 1H), 1.99-1.69 (m, 5H), 1.55 -1.18 (m, 5H).

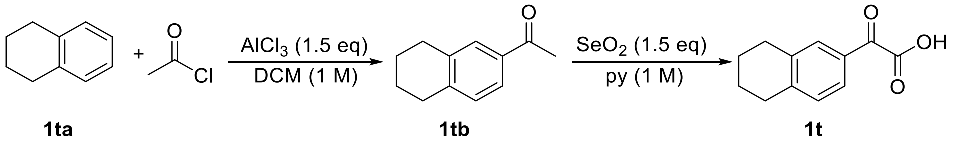

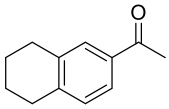

1tb

1-(5,6,7,8-Tetrahydronaphthalen-2-yl)ethan-1-one (1tb).[4] To an oven-dried flask cooled under N_2, tetralin (2.64 g, 20.0 mmol), $AlCl_3$ (4.0 g, 30 mmol) and DCM (20 mL) were added. Acetyl chloride (1.7 mL, 24.0 mmol) was added slowly at 0 °C. The reaction mixture was stirred at 0 °C to rt overnight. The reaction was quenched by HCl (1N) at 0 °C, extracted by DCM and dried over anhydrous Na_2SO_4. After filtration, the filtrate was condensed and purified by column chromatography (PE/EA = 20:1) through silica gel to obtain **1tb** (1.8 g, 10.1 mmol, 51% yield) as a yellow oil. ^{1}H NMR (500 MHz, $CDCl_3$) δ 7.67-7.62 (m, 2H), 7.10 (d, J = 8.5 Hz, 1H), 2.84-2.74 (m, 4H), 2.54 (s, 3H), 1.83-1.75 (m, 4H).

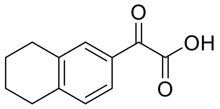

1t

2-Oxo-2-(5,6,7,8-tetrahydronaphthalen-2-yl)acetic acid (1t). Prepared according to the general procedure affording **1tb** (1.9000 g, 9.31 mmol, 93% yield) as a yellow solid. mp: 63-65 °C. IR ν 3456, 1640, 1400 cm^{-1}; ^{1}H NMR

(500 MHz, $CDCl_3$) δ 8.79 (s, 1H), 7.93-7.85 (m, 2H), 7.16 (d, J = 8.5 Hz, 1H), 2.86-2.74 (m, 4H), 1.86-1.73 (m, 4H); ^{13}C NMR (126 MHz, $CDCl_3$) δ 185.3, 164.4, 146.4, 138.0, 131.7, 129.7, 129.2, 127.7, 29.9, 29.1, 22.7, 22.5; HRMS (ESI-TOF) Calcd for $C_{12}H_{12}O_3Na$ $[M+Na]^+$: 227.0684; found 227.0682.

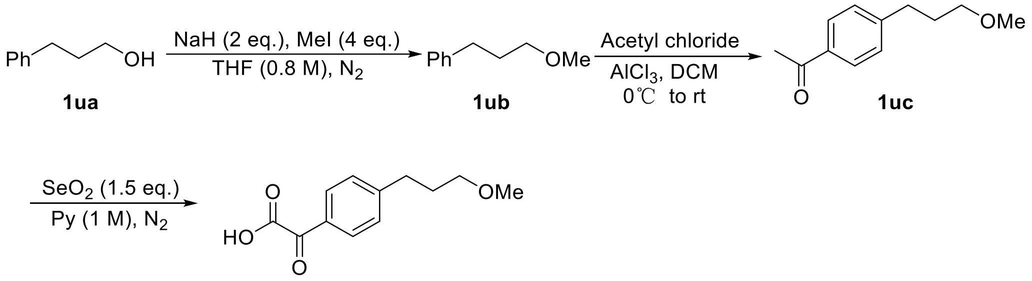

1u

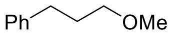

1ub

(3-Methoxypropyl)benzene (1ub).[5] To an oven-dried flask cooled under N_2, **1ua** (2.72 g, 20.0 mmol) and THF (25 mL) were added. NaH (1.60 g, 40.0 mmol, 60% in mineral oil) was added slowly at 0 °C. The reaction mixture was stirred at rt for 1 h. MeI (11.36 g, 80 mmol) was added slowly at 0 °C and stirred at rt overnight. The reaction was quenched by Sat. NH_4Cl at 0 °C, extracted by DCM and dried over anhydrous Na_2SO_4. After filtration, the filtrate was condensed and purified by column chromatography (PE/EA = 30:1) through silica gel to obtain **1ub** (2.30 g, 15.3 mmol, 77% yield) as a yellow oil. ^1H NMR (500 MHz, $CDCl_3$) δ 7.30-7.24 (m, 2H), 7.20-7.14 (m, 3H), 3.37 (t, J = 6.0 Hz, 2H), 3.33 (s, 3H), 2.72- 2.64 (m, 2H), 1.92-1.84 (m, 1H).

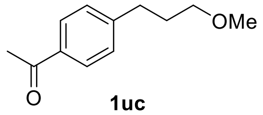

1uc

1-(4-(3-Methoxypropyl)phenyl)ethan-1-one (1uc).[6] To an oven-dried flask cooled under N_2, **1ub** (1.80 g, 12.0 mmol), $AlCl_3$ (2.23 g, 16.8 mmol) and DCM (20 mL) were added. Acetyl chloride (1.13 g, 14.4 mmol) was added slowly at 0 °C. The reaction mixture was stirred at 0 °C to rt overnight. The reaction was quenched by HCl (1N) at 0 °C, extracted by DCM and dried over anhydrous Na_2SO_4. After filtration, the filtrate was condensed and purified by column chromatography (PE/EA = 10:1) through silica gel to obtain **1uc** (747.0 mg, 3.9 mmol, 33% yield) as a yellow oil. ^1H NMR (500 MHz, $CDCl_3$) δ 7.88 (d, J = 8.0 Hz, 2H), 7.28 (d, J = 8.0 Hz, 2H), 3.38 (t, J = 6.5 Hz, 2H), 3.34 (s, 3H), 2.75 (t, J = 7.5 Hz, 2H), 2.58 (s, 3H), 1.94-1.86 (m, 2H).

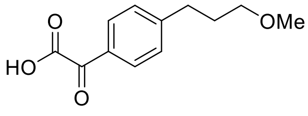

1u

2-(4-(3-Methoxypropyl)phenyl)-2-oxoacetic acid (1u). Then **1uc** was oxidized by SeO_2 according to the general procedure to afford **1u** as a yellow oil. IR *v* 3453, 2919, 2851, 1688, 1607, 737 cm^{-1}; ^1H NMR (500 MHz, $CDCl_3$) δ 9.24 (s, 1H), 8.08 (d, *J* = 7.0 Hz, 2H), 7.28 (d, *J* = 7.0 Hz, 2H), 3.42 (t, *J* = 6.5 Hz, 2H), 3.37 (s, 3H), 2.75 (t, *J* = 7.5 Hz, 2H), 1.97-1.84 (m, 2H); ^{13}C NMR (126 MHz, $CDCl_3$) δ 187.3, 165.4, 150.0, 148.4, 131.0, 130.3, 128.9, 128.6, 71.6, 58.4, 32.4, 30.4; HRMS (ESI-TOF) Calcd. for $C_{12}H_{14}O_4Na$ $[M+Na]^+$: 245.0790; Found 245.0789.

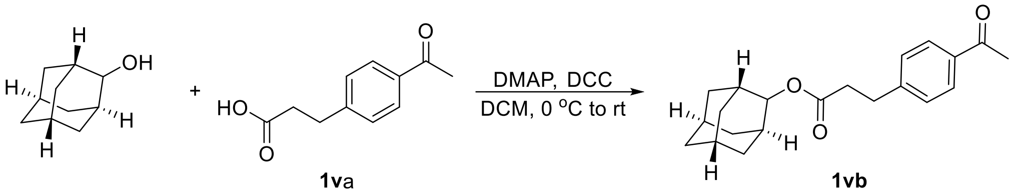

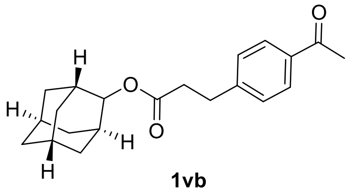

1vb

(*1r,3r,5r,7r*)-adamantan-2-yl 3-(4-acetylphenyl)propanoate (**1vb**). To an oven-dried 50 mL flask, 3-(4-acetylphenyl)propanoic acid **1va** (686.4 mg, 3.57 mmol), Adamantanol (468.7 mg, 3.08 mmol), DMAP (36.9 mg, 0.30 mmol) and DCM (10 mL) were added sequentially under N_2 at 0 °C. Then dicyclohexylcarbodiimide (798.2 mg, 3.87 mmol) was added and stirred for 10 min at this temperature and stirred at rt overnight. The reaction mixture filtered through a short pad of silica gel and washed by DCM. The filtrate was concentrated in vacuo before it was purified by flash chromatography on silica gel to afford the desired compound **1vb** (882.1 mg, 2.96 mmol, 83%) as a colorless oil. IR *v* 2908, 2854, 1728, 1683, 1266 cm^{-1}; ^1H NMR (500 MHz, $CDCl_3$) δ 7.88 (d, *J* = 8.0 Hz, 2H), 7.31 (d, *J* = 8.0 Hz, 2H), 4.95-4.90 (m, 1H), 3.03 (t, *J* = 7.5 Hz, 2H), 2.69 (t, *J* = 7.5 Hz, 2H), 2.57 (s, 3H), 1.97-1.88 (m, 4H), 1.87-1.78 (m, 4H), 1.78-1.69 (m, 4H), 1.56-1.48 (m, 2H); ^{13}C NMR (126 MHz, $CDCl_3$) δ 197.5, 171.7, 146.2, 135.2, 128.5, 128.4, 77.1, 37.2, 36.1, 35.5, 31.7, 31.6, 30.9, 27.0, 26.8, 26.4; HRMS (ESI-TOF) Calcd for $C_{21}H_{26}O_3Na$ $[M+Na]^+$: 349.1780; found 349.1778.

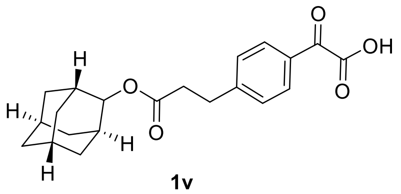

1v

2-(4-(3-(((*1r,3r,5r,7r*)-adamantan-2-yl)oxy)-3-oxopropyl)phenyl)-2-oxoacetic acid (1v). Then **1vb** was oxidized by SeO_2 according to the general procedure to afford **1v** as a yellow oil**.** IR *v* 2908, 2854, 1729, 1636, 1400, 1242

cm^{-1}; ^{1}H NMR (500 MHz, $CDCl_3$) δ 8.16 (d, *J* = 8.5 Hz, 2H), 7.83 (s, 1H), 7.38 (d, *J* = 8.0 Hz, 2H), 4.96-4.91 (m, 1H), 3.07 (t, *J* = 7.5 Hz, 2H), 2.73 (t, *J* = 7.5 Hz, 2H), 1.99-1.67 (m, 12H), 1.52 (d, *J* = 12.0 Hz, 2H); ^{13}C NMR (126 MHz, $CDCl_3$) δ 184.9, 172.2, 163.3, 149.0, 131.2, 130.2, 128.9, 77.7, 37.2, 36.2, 35.4, 31.8, 31.6, 31.2, 27.1, 26.8; HRMS (ESI-TOF) Calcd for $C_{22}H_{27}O_5$ $[M+H]^+$: 371.1858; Found 371.1853.

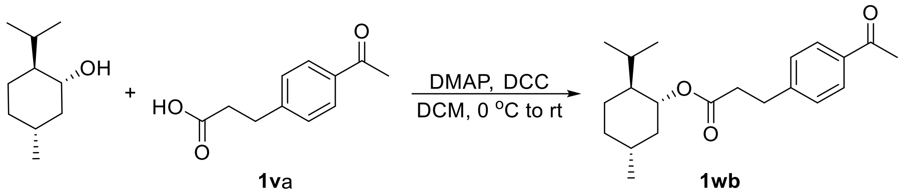

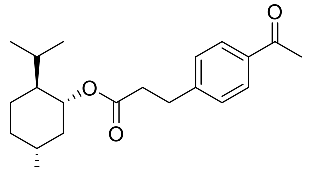

(1*R*,2*S*,5*R*)-2-isopropyl-5-methylcyclohexyl 3-(4-acetylphenyl)propanoate (1wb). To an oven-dried 50 mL flask, 3-(4-acetylphenyl)propanoic acid **1va** (695.6 mg, 3.6 mmol), L-menthol (466.8 mg, 3.0 mmol), DMAP (46.4 mg, 0.38 mmol) and DCM (10 mL) were added sequentially under N_2 at 0 °C. Then DCC (759.8 mg, 3.68 mmol) was added and stirred for 10 min at this temperature and stirred at rt overnight. The reaction mixture filtered through a short pad of silica gel and washed by DCM. The filtrate was concentrated in vacuo before it was purified by flash chromatography on silica gel to afford the desired compound **1wb** (793.0 mg, 2.40 mmol, 80%) as a colorless oil. IR *v* 3440, 2956, 1728, 16841641, 1266 cm^{-1}; ^{1}H NMR (500 MHz, $CDCl_3$) δ 7.87 (d, *J* = 8.5 Hz, 2H), 7.29 (d, *J* = 8.0 Hz, 2H), 4.66 (td, *J* = 11.0, 4.5 Hz, 1H), 3.00 (t, *J* = 7.5 Hz, 2H), 2.63 (t, *J* = 7.5 Hz, 2H), 2.56 (s, 3H), 1.96-1.86 (m, 1H), 1.72-1.59 (m, 3H), 1.50-1.39 (m, 1H), 1.36-1.25 (m, 1H), 1.07-0.96 (m, 1H), 0.90-0.84 (m, 4H), 0.82 (d, *J* = 7.0 Hz, 3H), 0.68 (d, *J* = 7.0 Hz, 3H); ^{13}C NMR (126 MHz, $CDCl_3$) δ 197.6, 171.9, 146.2, 135.3, 128.51, 128.48, 74.3, 46.9, 40.8, 35.5, 34.1, 31.3, 30.9, 26.5, 26.1, 23.3, 21.9, 20.7, 16.2. HRMS (ESI-TOF) Calcd for $C_{21}H_{30}O_3Na$ $[M+Na]^+$: 353.2093; Found 353.2097.

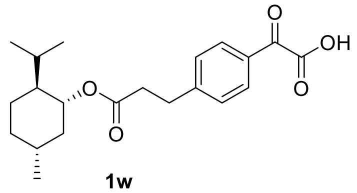

2-(4-(3-(((1*R*,2*S*,5*R*)-2-isopropyl-5-methylcyclohexyl)oxy)-3-oxopropyl)phenyl)-2-oxoacetic acid (1w). Then **1wb** was oxidized by SeO_2 according to the general procedure to afford **1w** as yellow oil**.** IR 3456, 1637, 1400 cm^{-1}; ^{1}H NMR (500 MHz,

CDCl3) δ 8.24-8.03 (m, 3H), 7.35-7.27 (m, 2H), 4.67 (td, J = 11.0, 4.5 Hz, 1H), 3.00 (t, J = 7.0 Hz, 2H), 2.63 (t, J = 7.0 Hz, 2H), 1.96-1.87 (m, 1H), 1.75-1.58 (m, 3H), 1.52-1.41 (m, 1H), 1.38-1.28 (m, 2H), 1.07-0.90 (m, 2H), 0.88 (d, J = 6.5 Hz, 3H), 0.83 (d, J = 6.5 Hz, 3H), 0.68 (d, J = 7.0 Hz, 3H); ^{13}C NMR (126 MHz, $CDCl_3$) δ 185.1, 172.4, 163.4, 148.9, 131.2, 130.2, 128.9, 74.8, 46.9, 40.8, 35.3, 34.1, 31.3, 31.1, 26.1, 23.3, 21.9, 20.7, 16.2: HRMS (ESI-TOF) Calcd for $C_{21}H_{29}O_5$ $[M+H]^+$: 361.2015; Found 361.2014.

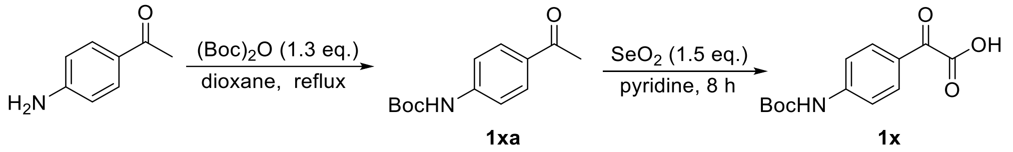

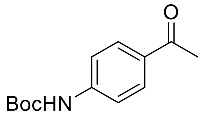

1xa

***Tert*-butyl (4-acetylphenyl)carbamate (1xa)**.[7] To an oven-dried 50 mL flask, 4-aminoacetophenone (1.3510 g, 10.0 mmol), di-*tert*-butyl dicarbonate (2.8373 g, 13.0 mmol) and 1,4-dioxane (20 mL) were added sequentially at rt under N_2. The reaction mixture was refluxed for 8 hours. The reaction mixture was condensed to provide a crude product which was purified through column chromatography (PE/EA = 5/1) on silica gel to afford **1xa** (1.7050 g, 7.30 mmol, 73% yield) as a white solid. ^{1}H NMR (500 MHz, $CDCl_3$) δ 7.91 (d, J = 8.5 Hz, 2H), 7.51 (d, J = 8.5 Hz, 2H), 7.29 (s, 1H), 2.57 (s, 3H), 1.51 (s, 9H).

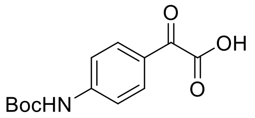

1x

2-(4-((Tert-butoxycarbonyl)amino)phenyl)-2-oxoacetic acid (1x).[8] To a solution of ketone (705.8 mg, 3.0 mmol) in pyridine (3 mL) was added SeO_2 (499.1 mg, 4.5 mmol.), the reaction mixture was heated for 2 h at 110 °C and then 90 °C for 9 hours. After cooled to room temperature, the reaction mixture was filtered through a short pad of celite. The most of the solvent was evaporated and the mixture was acidified by HCl (2N). The mixture was extracted with EA and the organic layers were dried over anhydrous Na_2SO_4 and filtered to afford the crude product which was purified by chromatography through silica gel to obtain the corresponding a-oxocarboxylic acids **1x** (1.8100 g, 7.8 mmol, 78% yield) as a yellow solid. ^{1}H NMR (500 MHz, $CDCl_3$) δ 9.95 (s, 1H), 7.86 (d, J = 9.0 Hz, 2H), 7.70 (d, J = 9.0 Hz, 2H), 1.49 (s, 9H).

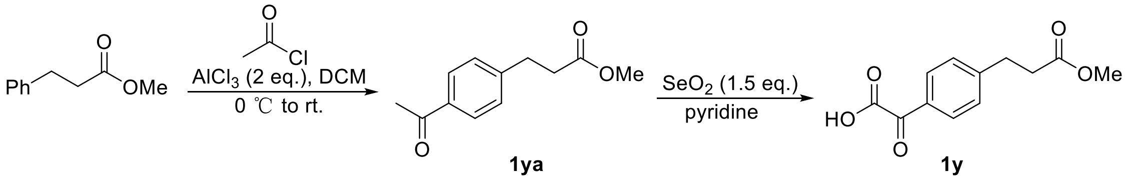

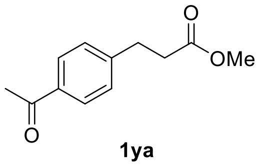

1ya

Methyl 3-(4-acetylphenyl)propanoate (1ya).[9] To an oven-dried 50 mL flask, benyl propionic methyl ester (17.8228 g, 100.0 mmol) and DCM (200 mL) were added sequentially at 0 °C under N_2. The aluminum chloride (26.6680 g, 200.0 mmol) was added slowly. The reaction mixture was stirred overnight at 0 °C to rt. The reaction mixture was quenched by water and extracted by DCM. The combined organic layers were dried over anhydrous Na_2SO_4 and filtrated. The filtration was condensed to provide a crude product which was purified through column chromatography (PE/EA = 5/1) on silica gel to afford **1ya** (16.4150 g, 80.0 mmol, 80% yield) as a yellow oil. ^{1}H NMR (500 MHz, $CDCl_3$) δ 7.88 (d, *J* = 8.5 Hz, 2H), 7.29 (d, *J* = 8.5 Hz, 2H), 3.65 (s, 3H), 3.00 (t, *J* = 7.5 Hz, 2H), 2.65 (t, *J* = 7.5 Hz, 2H), 2.56 (s, 3H).

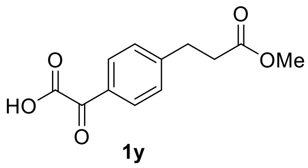

1y

2-(4-(3-Methoxy-3-oxopropyl)phenyl)-2-oxoacetic acid (1y). To a solution of ketone (1.0310 g, 5.0 mmol) in pyridine (5 mL) was added SeO_2 (832.2 mg, 7.5 mmol.), the reaction mixture was heated for 2 h at 110 °C and then 90 °C for 9 hours. After cooled to room temperature, the reaction mixture was filtered through a short pad of celite. The most of the solvent was evaporated and the mixture was acidified by HCl (2N). The mixture was extracted with EA and the organic layers were dried over anhydrous Na_2SO_4 and filtered to afford the crude product which was purified by chromatography through silica gel to obtain the corresponding a-oxocarboxylic acids **1y** (822.0 m g, 3.48 mmol, 70% yield) as a yellow oil. IR ν 2955, 2566, 1729, 1605, 1440, 1367 cm^{-1}; ^{1}H NMR (500 MHz, $CDCl_3$) δ 10.04 (s, 1H), 8.12 (d, *J* = 8.0 Hz, 2H), 7.36 (d, *J* = 8.0 Hz, 2H), 3.69 (s, 3H), 3.04 (t, *J* = 7.5 Hz, 2H), 2.71 (t, *J* = 7.5 Hz, 2H); ^{13}C NMR (125 MHz, $CDCl_3$) δ 185.1, 173.6, 163.7, 148.6, 130.9, 130.1, 128.8, 51.9, 34.7, 30.7; HRMS (ESI-TOF) Calcd for $C_{12}H_{13}O_5$ $[M+H]^+$: 237.0685; Found 237.0764.

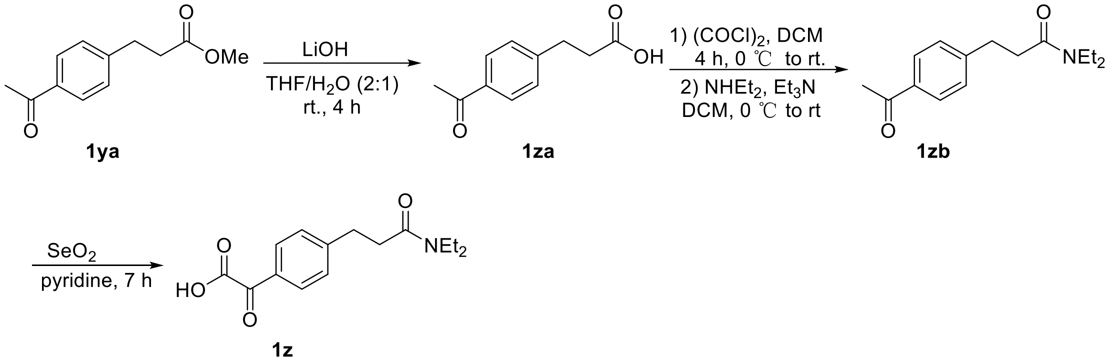

To a 50 mL flask, **1ya** (2.0620 g, 10.0 mmol), THF (20 mL), H20 (10 mL) and LiOH (1.6760 g, 70.0 mmol) were added sequentially at rt. The reaction mixture was stirred at room temperature and monitored by thin layer chromatography (TLC). When the starting material was consumed by TLC, HCl (1 N) was added to acidify the reaction mixture and the reaction mixture was extracted with ethyl acetate. The combined organic layers were dried by Na_2SO_4. After filtration, the filtrate was condensed to afford a crude product **1za** which was used directly without further purification.

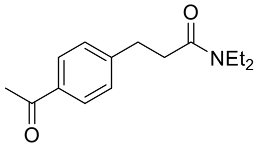

1zb

***N,N*-diethyl-3-(4-(2-oxopropanoyl)phenyl)propanamide (1zb)**. To an oven-dried 50 mL flask, the crude acid (961.1 mg, 5.0 mmol) and DCM (12 mL) were added sequentially at 0 °C under N_2. The oxalyl chloride (2.53 mL, 30.0 mmol) was added slowly. The reaction mixture was allowed to be warmed to rt and stirred for 4 h at rt. Then diethylamine (365.0 mg, 5.0 mmol), triethylamine (1.0120 g, 10.0 mmol) were added slowly at 0 °C. The mixture was stirred overnight at 0 °C to rt. The reaction mixture was quenched by water and extracted by DCM. The combined organic layers were dried over anhydrous Na_2SO_4 and filtrated. The filtration was condensed to provide a crude product which was purified through column chromatograpy to afford **1zb** (1.2050 g, 4.35 mmol, 87% yield) as a yellow oil. IR ν 297.3, 1680, 1638, 1431, 1268 cm^{-1}; ^1H NMR (500 MHz, $CDCl_3$) δ 7.88 (d, J = 8.0 Hz, 2H), 7.33 (d, J = 9.5 Hz, 2H), 3.38 (q, J = 7.0 Hz, 2H), 3.25 (q, J = 7.0 Hz, 2H), 3.05 (t, J = 7.5 Hz, 2H), 2.63 (t, J = 7.5 Hz, 2H), 2.58 (s, 3H), 1.15-1.07 (m, 6H); ^{13}C NMR (126 MHz, $CDCl_3$) δ 197.5, 170.4, 147.2, 135.0, 128.5, 128.3, 41.7, 40.0, 34.1, 31.2, 26.3, 14.0, 12.8; HRMS (ESI-TOF) Calcd for $C_{15}H_{22}NO_2$ $[M+H]^+$: 248.1651; Found 248.1648.

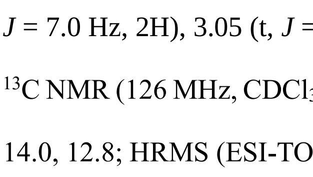

1z

2-(4-(3-(Diethylamino)-3-oxopropyl)phenyl)-2-oxoacetic acid (1z). Prepared according to the general procedure affording **1z** (1.2050 g, 4.35 mmol, 87% yield) as a yellow oil. IR ν 2934, 1729, 1681, 1567, 981 cm^{-1}; ^1H NMR (500 MHz, DMSO-d_6) δ 7.84 (d, J = 8.5 Hz, 2H),

7.48 (d, J = 8.0 Hz, 2H), 3.24 (q, J = 7.0 Hz, 4H), 2.93 (t, J = 7.5 Hz, 2H), 2.63 (t, J = 7.5 Hz, 2H), 1.03 (t, J = 7.0 Hz, 3H), 0.98 (t, J = 7.0 Hz, 3H); ^{13}C NMR (126 MHz, DMSO) δ 188.5, 169.9, 166.4, 149.9, 129.9, 129.6, 129.4, 41.3, 33.2, 31.0, 14.2, 13.1; HRMS (ESI-TOF) Calcd for $C_{15}H_{20}NO_4$ $[M+H]^+$: 278.1392; Found 278.1389.

General Procedure A for Synthesis of 1,4-Diketones with Acetylene

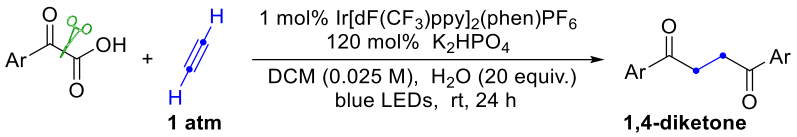

To a 25 mL flask, α-Oxocarboxylic Acid **1** (0.3 mmol), Ir[dF(CF_3)ppy]$_2$(phen)PF_6 (0.003 mmol), K_2HPO_4 (0.36 mmol, 1.2 equiv.), H_2O (6.0 mmol, 20 equiv.) and DCM (12 mL) were added sequentially under N_2 atmosphere. The mixture was degassed through three freeze-pump-thaw cycles under acetylene gas and then an acetylene gas balloon was attached through a long syringe needle. The reaction mixture was irradiated by 12 W blue LEDs at a distance of 5 cm for 24 h with a cooling fan. The reaction mixture was then diluted with EtOAc and filtered through a short pad of silica using EtOAc. The filtrate was concentrated in *vacuo* before it was purified by flash chromatography on silica gel to afford **2**.

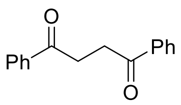

2a

1,4-diphenylbutane-1,4-dione (2a). [10] Prepared according to the general procedure A employing Ir[dF(CF_3)ppy]$_2$(phen)PF_6 (3.3 mg, 0.003 mmol), **1a** (45.0 mg, 0.3 mmol), K_2HPO_4 (0.36 mmol, 66.8 mg), H_2O (6.0 mmol, 108 μL) and DCM (12 mL). After 24 h, the reaction was diluted with EtOAc and passed through a short pad of silica using EtOAc. The filtrate was concentrated in vacuo before it was purified by flash chromatography (PE/EA = 20:1) on silica gel to afford **2a** (28.4 mg, 0.12 mmol, 79% yield) as a white solid. ^{1}H NMR (500 MHz, $CDCl_3$) δ 8.07-8.01 (m, 4H), 7.61-7.55 (m, 2H), 7.51-7.45 (m, 4H), 3.47 (s, 4H); ^{13}C NMR (126 MHz, $CDCl_3$) δ 198.6, 136.8, 133.1, 128.6, 128.1, 32.6.

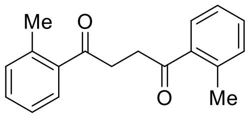

2b

1,4-di-*o*-tolylbutane-1,4-dione (2b).[11] Prepared according to the general procedure A employing Ir[dF(CF_3)ppy]$_2$(phen)PF_6 (3.2 mg, 0.003 mmol), **1b** (49.5 mg, 0.30 mmol), K_2HPO_4 (0.36 mmol, 62.9 mg), H_2O (6.0 mmol, 108 μL) and DCM (12 mL). After 24 h, the reaction was diluted with EtOAc and passed through a short pad of silica using EtOAc. The filtrate was concentrated in *vacuo* before it was purified by flash chromatography (PE/EA = 20:1) on silica gel to afford **2b** (18.0 mg, 0.068 mmol, 45% yield)

as a white solid. ^{1}H NMR (500 MHz, $CDCl_3$) δ 7.80 -7.77 (m, 2H), 7.40-7.35 (m, 2H), 7.28 (t, J = 7.5 Hz, 2H), 7.25 (d, J = 6.5 Hz, 2H), 3.33 (s, 4H), 2.50 (s, 6H); ^{13}C NMR (126 MHz, $CDCl_3$) δ 202.6, 138.0, 137.9, 131.8, 131.2, 128.5, 125.7, 35.6, 21.2.

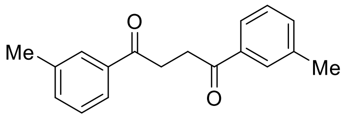

2c

1,4-*di*-*m*-tolylbutane-1,4-dione (2c).[12] Prepared according to the general procedure A employing Ir[dF(CF_3)ppy]$_2$(phen)PF_6 (3.1 mg, 0.003 mmol), **1c** (50.0 mg, 0.30 mmol), K_2HPO_4 (63.0 mmol, 0.36 mg), H_2O (6 mmol, 108 μL) and DCM (12 mL). After 24 h, the reaction was diluted with EtOAc and passed through a short pad of silica using EtOAc. The filtrate was concentrated in *vacuo* before it was purified by flash chromatography (PE/EA = 20:1) on silica gel to afford **2c** (26.37 mg, 0.099 mmol, 66% yield) as a white solid. ^{1}H NMR (500 MHz, $CDCl_3$) δ 7.87-7.81 (m, 4H), 7.43-7.33 (m, 4H), 3.44 (s, 4H), 2.42 (s, 6H); ^{13}C NMR (126 MHz, $CDCl_3$) δ 198.9, 138.3, 136.8, 133.8, 128.6, 128.4, 125.3, 32.7, 21.3.

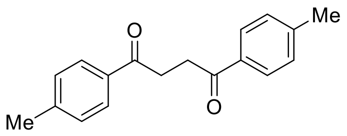

2d

1,4-di-*p*-tolylbutane-1,4-dione (2d).[13] Prepared according to the general procedure A employing Ir[dF(CF_3)ppy]$_2$(phen)PF_6 (3.3 mg, 0.003 mmol), **1b** (49.0 mg, 0.3 mmol), K_2HPO_4 (0.36 mmol, 63.0 mg), H_2O (6.0 mmol, 108 μL) and DCM (12 mL). After 24 h, the reaction was diluted with EtOAc and passed through a short pad of silica using EtOAc. The filtrate was concentrated in *vacuo* before it was purified by flash chromatography (PE/EA = 20:1) on silica gel to afford **2d** (27.57 mg, 0.103 mmol, 69% yield) as a white solid. ^{1}H NMR (500 MHz, $CDCl_3$) δ 7.94 (d, J = 8.0 Hz, 4H), 7.27 (d, J = 9.0 Hz, 4H), 3.42 (s, 4H), 2.42 (s, 6H); ^{13}C NMR (126 MHz, $CDCl_3$) δ 198.4, 143.8, 134.4, 129.2, 128.2, 32.5 , 21.6.

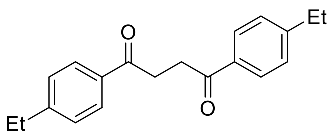

2e

1,4-Bis(4-ethylphenyl)butane-1,4-dione (2e).[13] Prepared according to the general procedure A employing Ir[dF(CF_3)ppy]$_2$(phen)PF_6 (3.4 mg, 0.003 mmol), **1e** (53.9 mg, 0.3 mmol), K_2HPO_4 (0.36 mmol, 62.7 mg), H_2O (6.0 mmol, 108 μL) and DCM (12 mL). After 24 h, the reaction was diluted with EtOAc and passed through a short pad of silica using EtOAc. The filtrate was concentrated in *vacuo* before it was purified by flash chromatography (PE/EA = 20:1) on silica gel to afford **2e** (29.1 mg, 0.099 mmol, 66% yield) as a white solid. ^{1}H NMR (500 MHz, $CDCl_3$) δ 7.96 (d, J = 8.0 Hz, 4H), 7.29 (d, J = 8.0 Hz, 4H), 3.43 (s, 4H), 2.71 (q, J = 7.5 Hz, 4H), 1.26 (t, J = 7.5 Hz, 6H); ^{13}C NMR (126 MHz, $CDCl_3$) δ 198.4, 150.0, 134.6, 128.3, 128.0, 32.5, 28.9, 15.2.

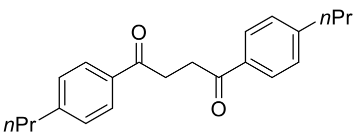

2f

1,4-Bis(4-propylphenyl)butane-1,4-dione (2f). [14] Prepared according to the general procedure A employing $Ir[dF(CF_3)ppy]_2(phen)PF_6$ (3.2 mg, 0.003 mmol), **1f** (57.1 mg, 0.3 mmol), K_2HPO_4 (0.36 mmol, 62.7 mg), H_2O (6.0 mmol, 108 μL) and DCM (12 mL). After 24 h, the reaction was diluted with EtOAc and passed through a short pad of silica using EtOAc. The filtrate was concentrated in *vacuo* before it was purified by flash chromatography (PE/EA = 20:1) on silica gel to afford **2f** (30.6 mg, 0.095 mmol, 63% yield) as a white solid. ^{1}H NMR (500 MHz, $CDCl_3$) δ 7.96 (d, J = 8.0 Hz, 4H), 7.27 (d, J = 8.0 Hz, 4H), 3.43 (s, 4H), 2.65 (t, J = 7.5 Hz, 4H), 1.72-1.62 (m, 4H), 0.95 (t, J = 7.5 Hz, 6H); ^{13}C NMR (126 MHz, $CDCl_3$) δ 198.4, 148.5, 134.6 , 128.6, 128.2, 38.0, 32.5, 24.2, 13.7.

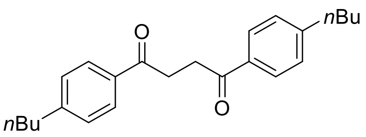

2g

1,4-Bis(4-butylphenyl)butane-1,4-dione (2g).[14] Prepared according to the general procedure A employing $Ir[dF(CF_3)ppy]_2(phen)PF_6$ (3.3 mg, 0.003 mmol), **1g** (61.9 mg, 0.30 mmol), K_2HPO_4 (62.9 mg, 0.36 mmol), H_2O (6.0 mmol, 108 μL) and DCM (12 mL). After 24 h, the reaction was diluted with EtOAc and passed through a short pad of silica using EtOAc. The filtrate was concentrated in *vacuo* before it was purified by flash chromatography (PE/EA = 40:1) on silica gel to afford **2g** (40.7 mg, 0.116 mmol, 77% yield) as a white solid. ^{1}H NMR (500 MHz, $CDCl_3$) δ 7.95 (d, J = 8.0 Hz, 4H), 7.27 (d, J = 8.0 Hz, 4H), 3.43 (s, 4H), 2.67 (t, J = 7.5 Hz, 4H), 1.68-1.57 (m, 4H), 1.41-1.31 (m, 4H), 0.93 (t, J = 7.5 Hz, 6H); ^{13}C NMR (126 MHz, $CDCl_3$) δ 198.4, 148.8, 134.5, 128.6, 128.2, 35.7, 33.2, 32.5, 22.3, 13.9.

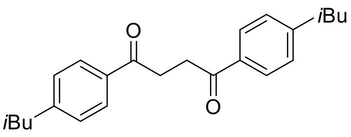

2h

1,4-Bis(4-isobutylphenyl)butane-1,4-dione (2h). Prepared according to the general procedure A employing $Ir[dF(CF_3)ppy]_2(phen)PF_6$ (3.2 mg, 0.003 mmol), **1h** (68.6 mg, 0.33 mmol), K_2HPO_4 (0.36 mmol, 62.5 mg), H_2O (6.0 mmol, 108 μL) and DCM (12 mL). After 24 h, the reaction was diluted with EtOAc and passed through a short pad of silica using EtOAc. The filtrate was concentrated in *vacuo* before it was purified by flash chromatography (PE/EA = 30:1) on silica gel to afford **2h** (33.1 mg, 0.094 mmol, 63% yield) as a white solid. mp = 129-130 °C; IR *v* 2954, 1669, 1638, 1402 cm^{-1}; ^{1}H NMR (500 MHz, $CDCl_3$) δ 7.95 (d, J = 8.5 Hz, 4H), 7.24 (d, J = 8.0 Hz, 4H), 3.43 (s, 4H), 2.54 (d, J = 7.0 Hz, 4H), 1.97-1.84 (m, 2H), 0.91 (d, J = 6.5 Hz, 6H); ^{13}C NMR (126 MHz, $CDCl_3$) δ 198.4, 147.5, 134.6, 129.3, 128.1, 45.4, 32.5, 30.1, 22.3; HRMS

(ESI-TOF) Calcd for $C_{24}H_{31}O_2$ $[M+H]^+$: 351.2324; found 351.2318.

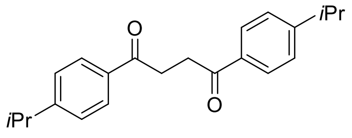

2i

1,4-Bis(4-isopropylphenyl)butane-1,4-dione (2i). Prepared according to the general procedure A employing Ir[dF(CF_3)ppy]$_2$(phen)PF_6 (3.1 mg, 0.003 mmol), **1i** (57.7 mg, 0.30 mmol), K_2HPO_4 (0.36 mmol, 62.7 mg), H_2O (6.0 mmol, 108 μL) and DCM (12 mL). After 24 h, the reaction was diluted with EtOAc and passed through a short pad of silica using EtOAc. The filtrate was concentrated in *vacuo* before it was purified by flash chromatography (PE/EA = 40:1) on silica gel to afford **2i** (24.2 mg, 0.075 mmol, 50% yield) as a white solid. mp = 96-97 °C; IR ν 2957, 1678, 1605, 1264, 1229, 739 cm-1; ^{1}H NMR (500 MHz, $CDCl_3$) δ 7.97 (d, J = 8.5 Hz, 4H), 7.32 (d, J = 8.5 Hz, 4H), 3.43 (s, 4H), 3.02-2.92 (m, 2H), 1.27 (d, J = 7.0 Hz, 12H); ^{13}C NMR (126 MHz, $CDCl_3$) δ 198.4, 154.6, 134.7, 128.4, 126.6, 34.2, 32.5, 23.6; HRMS (ESI-TOF) Calcd for $C_{22}H_{26}O_2Na$ $[M+Na]^+$: 345.1830; found 345.1824.

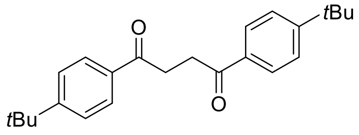

2j

1,4-Bis(4-(*tert*-butyl)phenyl)butane-1,4-dione (2j).[11] Prepared according to the general procedure A employing Ir[dF(CF_3)ppy]$_2$(phen)PF_6 (3.3 mg, 0.003 mmol), **1j** (61.8 mg, 0.30 mmol), K_2HPO_4 (62.6 mg, 0.36 mmol), H_2O (10.0 mmol, 180 μL) and DCM (12 mL). After 24 h, the reaction was diluted with EtOAc and passed through a short pad of silica using EtOAc. The filtrate was concentrated in *vacuo* before it was purified by flash chromatography (PE/EA = 20:1) on silica gel to afford **2j** (30.0 mg, 0.086 mmol, 57% yield) as a white solid. ^{1}H NMR (500 MHz, $CDCl_3$) δ 7.98 (d, J = 8.5 Hz, 4H), 7.49 (d, J = 8.5 Hz, 4H), 3.43 (s, 4H), 1.35 (s, 18H); ^{13}C NMR (126 MHz, $CDCl_3$) δ 198.4, 156.8, 134.3, 128.1, 125.5, 35.1, 32.6 , 31.1.

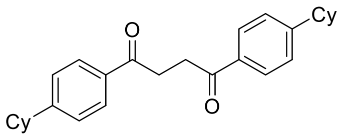

2k

1,4-Bis(4-cyclohexylphenyl)butane-1,4-dione (2k). Prepared according to the general procedure A employing Ir[dF(CF_3)ppy]$_2$(phen)PF_6 (3.3 mg, 0.003 mmol), **1k** (69.6 mg, 0.30 mmol), K_2HPO_4 (0.36 mmol, 62.9 mg), H_2O (6.0 mmol, 180 μL) and DCM (12 mL). After 24 h, the reaction was diluted with EtOAc and passed through a short pad of silica using EtOAc. The filtrate was concentrated in *vacuo* before it was purified by flash chromatography (PE/EA = 30:1) on silica gel to afford **2k** (36.2 mg, 0.090 mmol, 60% yield) as a white solid. mp = 164-165 °C. IR ν 3456, 1637, 1400 cm-1; ^{1}H NMR (500 MHz, $CDCl_3$) δ 7.96 (d, J = 8.0 Hz, 4H), 7.30 (d, J = 8.0 Hz, 4H), 3.43 (s, 4H), 2.62-2.53 (m, 2H), 1.93-1.82 (m, 9H), 1.81-1.71 (m, 2H), 1.49-1.35 (m, 9H);

^{13}C NMR (126 MHz, $CDCl_3$) δ 198.5, 153.7, 134.7, 128.3, 127.0, 44.7, 34.1, 32.5, 26.7, 26.0; HRMS (ESI-TOF) Calcd for $C_{28}H_{35}O_2$ $[M+H]^+$: 403.2637; found 403.2639.

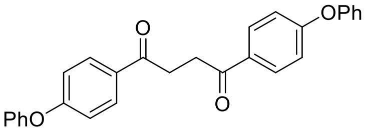

2l

1,4-Bis(4-phenoxyphenyl)butane-1,4-dione (2l). Prepared according to the general procedure A employing Ir[dF(CF_3)ppy]$_2$(phen)PF_6 (3.3 mg, 0.003 mmol), **1l** (72.8 mg, 0.30 mmol), K_2HPO_4 (62.9 mg, 0.36 mmol), H_2O (6.0 mmol, 108 μL) and DCM (12 mL). After 24 h, the reaction was diluted with EtOAc and passed through a short pad of silica using EtOAc. The filtrate was concentrated in *vacuo* before it was purified by flash chromatography (PE/EA = 10:1) on silica gel to afford **2l** (27.9 mg, 0.066 mmol, 44% yield) as a white solid. mp = 133-134 °C. IR ν 2921, 1674, 1592, 1266, 754 cm^{-1}; ^1H NMR (500 MHz, $CDCl_3$) δ 8.02 (d, *J* = 8.5 Hz, 4H), 7.40 (t, *J* = 8.0 Hz, 4H), 7.20 (t, *J* = 7.5 Hz, 2H), 7.08 (d, *J* = 8.0 Hz, 4H), 7.02 (d, *J* = 9.0 Hz, 4H), 3.41 (s, 4H); ^{13}C NMR (126 MHz, $CDCl_3$) δ 197.3, 162.0, 155.5, 131.5, 130.4, 130.0, 124.6, 120.1, 117.4, 32.4 ; HRMS (ESI-TOF) Calcd for $C_{28}H_{23}O_4$ $[M+H]^+$: 423.1596; found 423.1598.

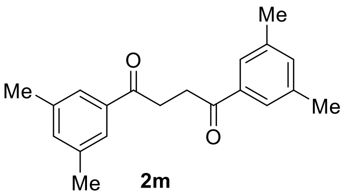

2m

1,4-Bis(3,5-dimethylphenyl)butane-1,4-dione (2m). [15]Prepared according to the general procedure A employing Ir[dF(CF_3)ppy]$_2$(phen)PF_6 (3.3 mg, 0.003 mmol), **1m** (53.9 mg, 0.3 mmol), K_2HPO_4 (0.36 mmol, 63.0 mg), H_2O (6.0 mmol, 108 μL) and DCM (12 mL). After 24 h, the reaction was diluted with EtOAc and passed through a short pad of silica using EtOAc. The filtrate was concentrated in *vacuo* before it was purified by flash chromatography (PE/EA = 20:1) on silica gel to afford **2m** (30.0 mg, 0.102 mmol, 68% yield) as a white solid. ^1H NMR (500 MHz, $CDCl_3$) δ 7.65 (s, 4H), 7.21 (s, 2H), 3.42 (s, 4H), 2.38 (s, 12H); ^{13}C NMR (126 MHz, $CDCl_3$) δ 199.20, 138.2, 136.9, 134.7, 125.9, 32.8, 21.2.

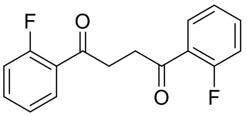

2n

1,4-Bis(2-fluorophenyl)butane-1,4-dione (2n).[16] Prepared according to the general procedure A employing Ir[dF(CF_3)ppy]$_2$(phen)PF_6 (3.3 mg, 0.003 mmol), **1n** (50.4 mg, 0.3 mmol), K_2HPO_4 (0.36 mmol, 63.0 mg), H_2O (6.0 mmol, 108 μL) and DCM (12 mL). After 24 h, the reaction was diluted with EtOAc and passed through a short pad of silica using EtOAc. The filtrate was concentrated in *vacuo* before it was purified by flash chromatography (PE/EA = 20:1) on silica gel to afford **2n** (18.9 mg, 0.069 mmol, 51% yield) as a white solid. ^1H NMR (500 MHz, $CDCl_3$) δ 7.90 (td, *J* = 2.0, 8.0 Hz, 2H), 7.56-7.49 (m, 2H), 7.26-7.21 (m, 2H), 7.19-7.12 (m, 2H), 3.47-3.41 (m, 4H); ^{13}C NMR (126 MHz, $CDCl_3$) δ

196.7 (d, J = 4.0 Hz), 162.1 (d, J = 255.1 Hz), 134.5 (d, J = 9.0 Hz), 130.7 (d, J = 2.6 Hz), 125.5 (d, J = 13.1 Hz), 124.4 (d, J = 3.3 Hz), 116.6 (d, J = 23.8 Hz), 37.42 (dd, J = 8.2, 2.0 Hz); ^{19}F NMR (471 MHz, $CDCl_3$) δ -109.0.

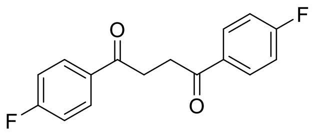

2o

1,4-Bis(4-fluorophenyl)butane-1,4-dione (2o).[16] Prepared according to the general procedure A employing Ir[dF(CF_3)ppy]$_2$(phen)PF_6 (3.3 mg, 0.003 mmol), **1o** (50.3 mg, 0.3 mmol), K_2HPO_4 (63.5 mg, 0.36 mmol), H_2O (6.0 mmol, 108 μL) and DCM (12 mL). After 24 h, the reaction was diluted with EtOAc and passed through a short pad of silica using EtOAc. The filtrate was concentrated in *vacuo* before it was purified by flash chromatography (PE/EA = 20:1) on silica gel to afford **2o** (27.2 mg, 0.099 mmol, 66% yield) as a white solid. ^{1}H NMR (500 MHz, $CDCl_3$) δ 8.10- 8.03 (m, 4H), 7.19-7.11 (m, 4H), 3.43 (s, 4H); ^{19}F NMR (471 MHz, $CDCl_3$) δ -105.11; ^{13}C NMR (126 MHz, $CDCl_3$) δ 197.0, 165.8 (d, J = 255.8 Hz), 133.2 (d, J = 2.5 Hz), 130.7 (d, J = 8.8 Hz), 115.7 (d, J = 22.7 Hz), 32.4.

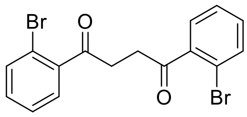

2p

1,4-Bis(2-bromophenyl)butane-1,4-dione (2p).[17] Prepared according to the general procedure A employing Ir[dF(CF_3)ppy]$_2$(phen)PF_6 (3.1 mg, 0.003 mmol), **1p** (72.4 mg, 0.31 mmol), K_2HPO_4 (63.7 mg, 0.36 mmol), H_2O (6.0 mmol, 108 μL) and DCM (12 mL). After 24 h, the reaction was diluted with EtOAc and passed through a short pad of silica using EtOAc. The filtrate was concentrated in *vacuo* before it was purified by flash chromatography (PE/EA = 20:1) on silica gel to afford **2p** (25.7 mg, 0.065 mmol, 43% yield) as a yellow oil. ^{1}H NMR (500 MHz, $CDCl_3$) δ 7.62 (d, J = 8.0 Hz, 2H), 7.57 (dd, J = 7.5, 1.5 Hz, 2H), 7.40 (dd, J = 7.5, 7.5 Hz, 2H), 7.33-7.28 (m, 2H), 3.38 (s, 4H); ^{13}C NMR (126 MHz, $CDCl_3$) δ 202.2, 141.3, 133.7, 131.6, 128.8, 127.5, 118.6, 36.9.

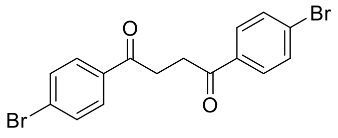

2q

1,4-Bis(4-bromophenyl)butane-1,4-dione (2q).[16] Prepared according to the general procedure A employing Ir[dF(CF_3)ppy]$_2$(phen)PF_6 (3.1 mg, 0.003 mmol), **1q** (68.9 mg, 0.30 mmol), K_2HPO_4 (63.0 mg, 0.36 mmol), H_2O (6.0 mmol, 108 μL) and DCM (12 mL). After 24 h, the reaction was diluted with EtOAc and passed through a short pad of silica using EtOAc. The filtrate was concentrated in *vacuo* before it was purified by flash chromatography (PE/EA = 20:1) on silica gel to afford **2q** (33.3 mg, 0.084 mmol, 56% yield) as a white solid. ^{1}H NMR (500 MHz, $CDCl_3$) δ 7.89 (d, J = 8.0 Hz, 4H), 7.62 (d, J = 8.0 Hz, 4H), 3.41 (s, 4H); ^{13}C NMR (126 MHz, $CDCl_3$) δ

197.5, 135.4, 131.9, 129.6, 128.4, 32.4.

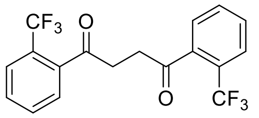

2r

1,4-Bis(2-(trifluoromethyl)phenyl)butane-1,4-dione (2r). Prepared according to the general procedure A employing Ir[dF(CF_3)ppy]$_2$(phen)PF_6 (3.2 mg, 0.003 mmol), **1r** (67.6 mg, 0.31 mmol), K_2HPO_4 (63.0 mg, 0.36 mmol), H_2O (6.0 mmol, 108 μL) and DCM (12 mL). After 48 h, the reaction was diluted with EtOAc and passed through a short pad of silica using EtOAc. The filtrate was concentrated in *vacuo* before it was purified by flash chromatography (PE/EA = 20:1) on silica gel to afford **2r** (28.1 mg, 0.075 mmol, 50% yield) as a yellow oil. IR ν 2920, 1702, 1637, 1399, 1314 cm^{-1}; ^{1}H NMR (500 MHz, $CDCl_3$) δ 7.75- 7.69 (m, 4H), 7.68-7.63 (m, 2H), 7.61-7.55 (m, 2H), 3.30 (s, 4H); ^{19}F NMR (471 MHz, $CDCl_3$) δ -58.2; ^{13}C NMR (126 MHz, $CDCl_3$) δ 202.6, 140.0, 131.9, 130.2, 127.5, 127.0 (dd, *J* = 49.0, 32.8 Hz), 126.6 (q, *J* = 5.0 Hz), 122.5 (t, *J* = 274.5 Hz), 37.0; HRMS (ESI-TOF) Calcd for $C_{18}H_{12}F_6O_2Na$ $[M+Na]^+$: 397.0639; found 397.0637.

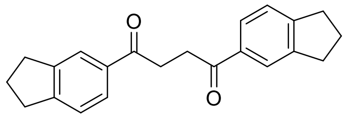

2s

1,4-Bis(2,3-dihydro-1*H*-inden-5-yl)butane-1,4-dione (2s). Prepared according to the general procedure A employing Ir[dF(CF_3)ppy]$_2$(phen)PF_6 (3.2 mg, 0.003 mmol), **1s** (54.7 mg, 0.28 mmol), K_2HPO_4 (63.3 mg, 0.36 mmol), H_2O (6.0 mmol, 108 μL) and DCM (12 mL). After 24 h, the reaction was diluted with EtOAc and passed through a short pad of silica using EtOAc. The filtrate was concentrated in *vacuo* before it was purified by flash chromatography (PE/EA = 20:1) on silica gel to afford **2s** (22.0 mg, 0.069 mmol, 46% yield) as a white solid. mp = 125-126 °C. IR ν 1638, 1401, 671 cm^{-1}; ^{1}H NMR (500 MHz, $CDCl_3$) δ 7.89 (s, 2H), 7.86-7.82 (m, 2H), 7.30 (d, *J* = 7.5 Hz, 2H), 3.43 (s, 4H), 2.96 (t, *J* = 7.5 Hz, 8H), 2.16-2.08 (m, 4H); ^{13}C NMR (126 MHz, $CDCl_3$) δ 198.8, 150.2, 144.7, 135.3, 126.6, 124.3, 124.1, 33.0, 32.8, 32.6, 25.4; HRMS (ESI-TOF) Calcd for $C_{22}H_{22}O_2Na$ $[M+Na]^+$: 341.1517; found 341.1511.

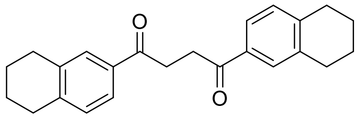

2t

1,4-Bis(5,6,7,8-tetrahydronaphthalen-2-yl)butane-1,4-dione (2t). Prepared according to the general procedure A employing Ir[dF(CF_3)ppy]$_2$(phen)PF_6 (3.3 mg, 0.003 mmol), **1t** (61.6 mg, 0.30 mmol), K_2HPO_4 (63.0 mg, 0.36 mmol), H_2O (6.0 mmol, 108 μL) and DCM (12 mL). After 24 h, the reaction was diluted with EtOAc and passed through a short pad of silica using EtOAc. The filtrate was concentrated in *vacuo* before it was purified by flash chromatography (PE/EA = 30:1) on silica gel to afford **2t** (30.1 mg, 0.087 mmol, 58% yield) as a white solid. mp = 124-125 °C. IR

ν 2918, 1677 cm^{-1}; ^{1}H NMR (500 MHz, $CDCl_3$) δ 7.77-7.72 (m, 4H), 7.17-7.12 (m, 2H), 3.40 (s, 4H), 2.88-2.76 (m, 8H), 1.88-1.74 (m, 8H); ^{13}C NMR (126 MHz, $CDCl_3$) δ 198.8, 143.1, 137.4, 134.3, 129.3, 129.0, 125.1, 32.6, 29.6, 29.3, 23.0, 22.82; HRMS (ESI-TOF) Calcd for $C_{24}H_{27}O_2$ $[M+H]^+$: 347.2011; found 347.2013.

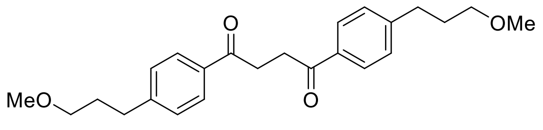

2u

1,4-Bis(4-(3-methoxypropyl)phenyl)butane-1,4-dione (2u). Prepared according to the general procedure A employing Ir[dF(CF_3)ppy]$_2$(phen)PF_6 (3.2 mg, 0.003 mmol), **1u** (66.0 mg, 0.30 mmol), K_2HPO_4 (63.1 mg 0.36 mmol), H_2O (6.0 mmol, 108 μL) and DCM (12 mL). After 24 h, the reaction was diluted with EtOAc and passed through a short pad of silica using EtOAc. The filtrate was concentrated in *vacuo* before it was purified by flash chromatography (PE/EA = 10:1) on silica gel to afford **2u** (30.0 mg, 0.078 mmol, 53% yield) as a colorless oil. IR ν 2921, 2866, 2828, 1675, 1605, 1569, 1176, 1117, 737 cm^{-1}; ^{1}H NMR (500 MHz, $CDCl_3$) δ 7.96 (d, J = 8.5 Hz, 4H), 7.29 (d, J = 8.5 Hz, 4H), 3.43 (s, 4H), 3.38 (t, J = 6.5 Hz, 4H), 3.34 (s, 6H), 2.76 (t, J = 7.5 Hz, 4H), 1.95-1.86 (m, 4H); ^{13}C NMR (126 MHz, $CDCl_3$) δ 198.3, 147.8, 134.8, 128.6, 128.3, 71.6, 58.5, 32.5, 32.3, 30.9; HRMS (ESI-TOF) Calcd for $C_{24}H_{30}O_4Na$ $[M+Na]^+$: 405.2042; found 405.2042.

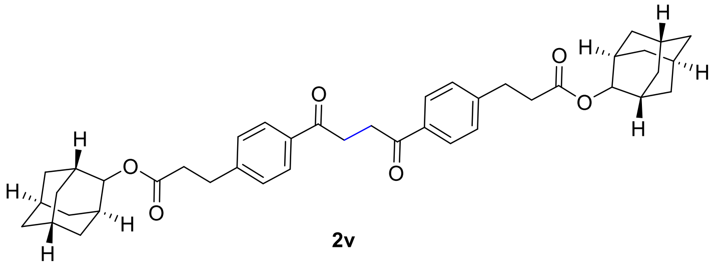
2v

(1*r*,3*r*,5*r*,7*r*)-adamantan-2-yl 4-(4-(4-(4-(3-(((1*r*,3*r*,5*r*,7*r*)-adamantan-2-yl)oxy)-3-oxopropyl)phenyl)-4-oxobutanoyl)phenyl)butanoate (2v). Prepared according to the general procedure A employing Ir[dF(CF_3)ppy]$_2$(phen)PF_6 (3.2 mg, 0.003 mmol), **1v** (111.9 mg, 0.30 mmol), K_2HPO_4 (62.5 mg, 0.36 mmol), H_2O (6.0 mmol, 108 μL) and DCM (12 mL). After 24 h, the reaction was diluted with EtOAc and passed through a short pad of silica using EtOAc. The filtrate was concentrated in *vacuo* before it was purified by flash chromatography (PE/EA = 5:1) on silica gel to afford **2v** (77.9 mg, 0.070 mmol, 47% yield) as a white solid. mp = 114-115 °C. IR ν 3437, 2908, 2854, 1728, 1680, 1608 cm^{-1}; ^{1}H NMR (500 MHz, $CDCl_3$) δ 7.96 (d, J = 8.0 Hz, 4H), 7.32 (d, J = 8.0 Hz, 4H), 4.96-4.90 (m, 2H), 3.42 (s, 4H), 3.04 (t, J = 7.5 Hz, 4H), 2.69 (t, J = 7.5 Hz, 4H), 1.98-1.88 (m, 9H), 1.87-1.78 (m, 9H), 1.78-1.75 (m, 2H), 1.75-1.68 (m, 4H), 1.56-1.49 (m, 4H); ^{13}C NMR (126 MHz, $CDCl_3$) δ 198.2, 171.8, 146.3,

135.0, 128.4, 128.3, 37.3, 36.2, 35.6, 32.5, 31.8, 31.7, 31.0, 27.1, 26.9; HRMS (ESI-TOF) Calcd for $C_{42}H_{59}O_6$ $[M+H]^+$: 659.4312; found 659.4312.

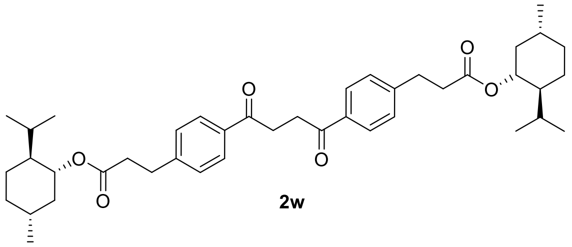

Bis((1*R*,2*S*,5*R*)-2-isopropyl-5-methylcyclohexyl) 3,3'-(succinylbis(4,1-phenylene))dipropionate (2w). Prepared according to the general procedure A employing $Ir[dF(CF_3)ppy]_2(phen)PF_6$ (3.3 mg, 0.003 mmol), **1w** (110.1 mg, 0.30 mmol), K_2HPO_4 (62.7 mg, 0.36 mmol), H_2O (6.0 mmol, 108 μL) and DCM (12 mL). After 24 h, the reaction was diluted with EtOAc and passed through a short pad of silica using EtOAc. The filtrate was concentrated in *vacuo* before it was purified by flash chromatography (PE/EA = 5:1) on silica gel to afford **2w** (45.0 mg, 0.068 mmol, 46% yield) as a yellow oil. IR *v* 3459, 2955, 1734, 1242 cm^{-1}; ^1H NMR (500 MHz, $CDCl_3$) δ 7.96 (d, *J* = 8.5 Hz, 4H), 7.31 (d, *J* = 8.0 Hz, 4H), 4.68 (td, *J* = 11.0, 4.5 Hz, 2H), 3.42 (s, 4H), 3.01 (t, *J* = 7.0 Hz, 4H), 2.64 (t, *J* = 7.5 Hz, 4H), 1.98-1.89 (m, 2H), 1.77-1.60 (m, 8H), 1.52-1.40 (m, 2H), 1.38-1.24 (m, 2H), 1.10-0.97 (m, 2H), 0.89 (d, *J* = 6.5 Hz, 6H), 0.84 (d, *J* = 7.0 Hz, 6H), 0.70 (d, *J* = 7.0 Hz, 6H); ^{13}C NMR (126 MHz, $CDCl_3$) δ 198.2, 172.0, 146.3, 135.1, 128.5, 128.3, 74.4, 47.0, 40.9, 35.6, 34.2, 32.5, 31.3, 31.0 , 26.2, 23.4, 21.9, 20.7, 16.3; HRMS (ESI-TOF) Calcd for $C_{44}H_{55}O_6$ $[M+H]^+$: 679.3999; found 679.4001.

General Procedure B for Synthesis of Tetrahydrofuran-containing Molecules with Acetylene

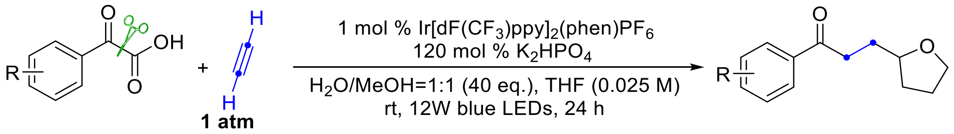

To a 25 mL flask, α-Oxocarboxylic Acid **1** (0.3 mmol), $Ir[dF(CF_3)ppy]_2(phen)PF_6$ (0.003 mmol), K_2HPO_4 (0.36 mmol, 1.2 equiv.), H_2O (6.0 mmol, 20 equiv.), MeOH (6.0 mmol, 20 equiv.) and THF (12 mL) were added sequentially under N_2 atmosphere. The mixture was degassed through three freeze-pump-thaw cycles under acetylene gas and then an acetylene gas balloon was attached through a long syringe needle. The reaction mixture was irradiated by 12 W blue LEDs at a distance of 5 cm for 24 h with a cooling fan. The reaction mixture was then diluted with EtOAc and filtered through a short pad of silica using EtOAc. The filtrate was concentrated in *vacuo* before it was

purified by flash chromatography on silica gel to afford **3**.

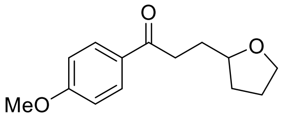

3a

1-(4-Methoxyphenyl)-3-(tetrahydrofuran-2-yl)propan-1-one (**3a**).[18] Prepared according to the general procedure B employing $Ir[dF(CF_3)ppy]_2(phen)PF_6$ (3.2 mg, 0.003 mmol), 2-(4-methoxyphenyl)-2-oxoacetic acid (54.1 mg, 0.30 mmol), K_2HPO_4 (0.36 mmol, 62.7 mg), H_2O (6.0 mmol, 108 μL), MeOH (6.0 mmol, 243 μL) and THF (12 mL). After 24 h, the reaction was diluted with EtOAc and passed through a short pad of silica using EtOAc. The filtrate was concentrated in *vacuo* before it was purified by flash chromatography (PE/EA = 5:1) on silica gel to afford **3a** (49.9 mg, 0.213 mmol, 71% yield) as a yellow oil. ^1H NMR (500 MHz, $CDCl_3$) δ 7.96 (d, J = 9.0 Hz, 2H), 6.92 (d, J = 9.0 Hz, 2H), 3.95-3.81 (m, 5H), 3.76-3.69 (m, 1H), 3.15-3.07 (m, 1H), 3.04-2.96 (m, 1H), 2.08-1.95 (m, 2H), 1.94-1.81 (m, 3H), 1.57-1.46 (m, 1H); ^{13}C NMR (126 MHz, $CDCl_3$) δ 198.5, 163.3, 130.2, 130.0, 113.6, 78.5, 67.6, 55.3, 35.0, 31.3, 30.1, 25.6.

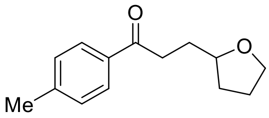

3b

3-(Tetrahydrofuran-2-yl)-1-(p-tolyl)propan-1-one (3b). Prepared according to the general procedure B employing $Ir[dF(CF_3)ppy]_2(phen)PF_6$ (3.1 mg, 0.003 mmol), 2-oxo-2-(*p*-tolyl)acetic acid (49.2 mg, 0.30 mmol), K_2HPO_4 (0.36 mmol, 62.7 mg), H_2O (6.0 mmol, 108 μL), MeOH (6.0 mmol, 243 μL) and THF (12 mL). After 24 h, the reaction was diluted with EtOAc and passed through a short pad of silica using EtOAc. The filtrate was concentrated in *vacuo* before it was purified by flash chromatography (PE/EA = 10:1) on silica gel to afford **3b** (34.05 mg, 0.156 mmol, 52% yield) as a yellow oil. IR 3031, 2925, 1680, 1573 cm^{-1}; ^1H NMR (500 MHz, $CDCl_3$) δ 7.88 (d, J = 8.0 Hz, 2H), 7.24 (d, J = 8.0 Hz, 2H), 3.95-3.82 (m, 2H), 3.77-3.69 (m, 1H), 3.19-3.09 (m, 1H), 3.08-2.97 (m, 1H), 2.40 (s, 3H), 2.10-1.95 (m, 2H), 1.95-1.82 (m, 3H), 1.57-1.47 (m, 1H); ^{13}C NMR (126 MHz, $CDCl_3$) δ 199.7, 143.6, 134.5, 129.2, 128.1, 78.5, 67.6, 35.3, 31.4, 30.0, 25.7, 21.5; HRMS (ESI-TOF) Calcd for $C_{14}H_{18}O_2Na$ $[M+Na]^+$: 241.1204; Found 241.1199.

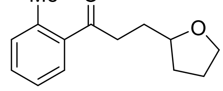

3c

3-(Tetrahydrofuran-2-yl)-1-(*o*-tolyl)propan-1-one (3c). Prepared according to the general procedure B employing $Ir[dF(CF_3)ppy]_2(phen)PF_6$ (3.1 mg, 0.003 mmol), 2-oxo-2-(*o*-tolyl)acetic acid (49.1 mg, 0.30 mmol), K_2HPO_4 (0.36 mmol, 62.5 mg), H_2O (6.0 mmol, 108 μL), MeOH (6.0 mmol, 243 μL) and THF (12 mL). After 24 h, the reaction was diluted with EtOAc and passed through a short pad of silica using

EtOAc. The filtrate was concentrated in *vacuo* before it was purified by flash chromatography (PE/EA = 10:1) on silica gel to afford **3c** (28.8 mg, 0.132 mmol, 44% yield) as a yellow oil. IR *v* 2958, 2925, 2852, 1683, 1638, 1401, 1069 cm^{-1}; ^1H NMR (500 MHz, $CDCl_3$) δ 7.66 (d, *J* = 8.0 Hz, 1H), 7.37-7.32 (m, 1H), 7.26 -7.21 (m, 2H), 3.92-3.82 (m, 2H), 3.76-3.67 (m, 1H), 3.11-3.02 (m, 1H),3.01-2.93 (m, 1H), 2.49 (s, 3H), 2.06-1.93 (m, 2H), 1.93-1.81 (m, 3H), 1.56-1.47 (m, 1H); ^{13}C NMR (126 MHz, $CDCl_3$) δ 204.2, 138.1, 137.9, 131.8, 131.0, 128.4, 125.6, 78.4, 67.6, 38.3, 31.4, 30.1, 25.7, 21.2; HRMS (ESI-TOF) Calcd for $C_{14}H_{18}O_2Na$ $[M+Na]^+$: 241.1204; Found 241.1201.

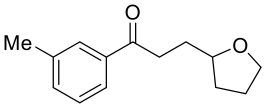

3d

3-(Tetrahydrofuran-2-yl)-1-(*m*-tolyl)propan-1-one (3d). Prepared according to the general procedure B employing Ir[dF(CF_3)ppy]$_2$(phen)PF_6 (3.2 mg, 0.003 mmol), 2-oxo-2-(*m*-tolyl)acetic acid (49.2 mg, 0.30 mmol), K_2HPO_4 (0.36 mmol, 62.6 mg), H_2O (6.0 mmol, 108 μL), MeOH (6.0 mmol, 243 μL) and THF (12 mL). After 24 h, the reaction was diluted with EtOAc and passed through a short pad of silica using EtOAc. The filtrate was concentrated in *vacuo* before it was purified by flash chromatography (PE/EA = 10:1) on silica gel to afford **3d** (39.29 mg, 0.180 mmol, 60% yield) as a pale yellow oil. IR *v* 2955, 2924, 2867, 1684, 1457, 1266, 1068, 690 cm^{-1}; ^1H NMR (500 MHz, $CDCl_3$) δ 7.82-7.75 (m, 2H), 7.38-7.31 (m, 2H), 3.94-3.83 (m, 2H), 3.77-3.69 (m, 1H), 3.21-3.11 (m, 1H), 3.09-2.99 (m, 1H), 2.40 (s, 3H), 2.07-1.96 (m, 2H), 1.95-1.82 (m, 3H), 1.57-1.48 (m, 1H); ^{13}C NMR (126 MHz, $CDCl_3$) δ 200.2, 138.2, 137.0, 133.6, 128.6, 128.4, 125.2, 78.5, 67.6, 35.5, 31.4, 29.9, 25.7, 21.3; HRMS (ESI-TOF) Calcd for $C_{14}H_{18}O_2Na$ $[M+Na]^+$: 241.1204; Found 241.1198.

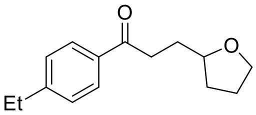

3e

1-(4-Ethylphenyl)-3-(tetrahydrofuran-2-yl)propan-1-one (3e). Prepared according to the general procedure B employing Ir[dF(CF_3)ppy]$_2$(phen)PF_6 (3.3 mg, 0.003 mmol), 2-(4-ethylphenyl)-2-oxoacetic acid (53.5 mg, 0.30 mmol), K_2HPO_4 (0.36 mmol, 62.5 mg), H_2O (6.0 mmol, 108 μL), MeOH (6.0 mmol, 243 μL) and THF (12 mL). After 24 h, the reaction was diluted with EtOAc and passed through a short pad of silica using EtOAc. The filtrate was concentrated in *vacuo* before it was purified by flash chromatography (PE/EA = 10:1) on silica gel to afford **3e** (38.3 mg, 0.165 mmol, 55% yield) as a yellow oil. IR *v* 2966, 2931, 2871, 1682, 1607, 1412, 1180, 1068, 833 cm^{-1}; ^1H NMR (500 MHz, $CDCl_3$) δ 7.91 (d, *J* = 8.5 Hz, 2H), 7.27 (d, *J* = 8.5 Hz, 2H), 3.94-3.83 (m, 2H), 3.77-3.69 (m, 1H), 3.19-3.10 (m, 1H), 3.08-2.98 (m, 1H), 2.70 (q, *J* = 7.50 Hz, 2H), 2.08-1.95 (m,

2H), 1.95-1.82 (m, 3H), 1.57-1.47 (m, 1H), 1.25 (t, *J* = 7.5 Hz, 3H); ^{13}C NMR (126 MHz, $CDCl_3$) δ 199.7, 149.8, 134.7, 128.2, 128.0, 78.5, 67.6, 35.3, 31.4, 30.0, 28.9, 25.7, 15.1; HRMS (ESI-TOF) Calcd for $C_{15}H_{20}O_2Na$ $[M+Na]^+$: 255.1361; Found 255.1356.

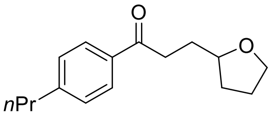
3f

1-(4-Propylphenyl)-3-(tetrahydrofuran-2-yl)propan-1-one (3f). Prepared according to the general procedure B employing Ir[dF(CF_3)ppy]$_2$(phen)PF_6 (3.2 mg, 0.003 mmol), 2-oxo-2-(4-propylphenyl)acetic acid (57.5 mg, 0.30 mmol), K_2HPO_4 (0.36 mmol, 62.7 mg), H_2O (6.0 mmol, 108 μL), MeOH (6.0 mmol, 243 μL) and THF (12 mL). After 24 h, the reaction was diluted with EtOAc and passed through a short pad of silica using EtOAc. The filtrate was concentrated in vacuo before it was purified by flash chromatography (PE/EA = 10:1) on silica gel to afford **3f** (37.68 mg, 0.153 mmol, 51% yield) as a yellow oil. IR *v* 3454, 3030, 2960, 1682, 1570 cm^{-1} ; ^{1}H NMR (500 MHz, $CDCl_3$) δ 7.90 (d, *J* = 8.0 Hz, 2H), 7.25 (d, *J* = 8.0 Hz, 2H), 3.94-3.82 (m, 2H), 3.76-3.68 (m, 1H), 3.19-3.10 (m, 1H), 3.08-2.98 (m, 1H), 2.63 (t, *J* = 7.5 Hz, 3H), 2.07-1.96 (m, 2H), 1.95-1.82 (m, 3H), 1.71-1.61 (m, 2H), 1.57-1.48 (m, 1H), 0.94 (t, *J* = 7.0 Hz, 3H); ^{13}C NMR (126 MHz, $CDCl_3$) δ 199.7, 148.3, 134.7, 128.6, 128.1, 78.5, 67.6, 38.0, 35.3, 31.4, 30.0, 25.7, 24.2, 13.7; HRMS (ESI-TOF) Calcd for $C_{16}H_{22}O_2Na$ $[M+Na]^+$: 269.1517; Found 269.1513.

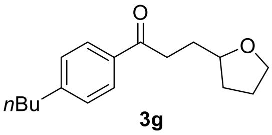
3g

1-(4-Butylphenyl)-3-(tetrahydrofuran-2-yl)propan-1-one (3g). Prepared according to the general procedure B employing Ir[dF(CF_3)ppy]$_2$(phen)PF_6 (3.3 mg, 0.003 mmol), 2-(4-butylphenyl)-2-oxoacetic acid (61.6 mg, 0.30 mmol), K_2HPO_4 (0.36 mmol, 62.6 mg), H_2O (6.0 mmol, 108 μL), MeOH (6.0 mmol, 243 μL) and THF (12 mL). After 24 h, the reaction was diluted with EtOAc and passed through a short pad of silica using EtOAc. The filtrate was concentrated in *vacuo* before it was purified by flash chromatography (PE/EA = 10:1) on silica gel to afford **3g** (48.43 mg, 0.186 mmol, 62% yield) as a yellow oil. IR *v* 2957, 2929, 2862, 1682, 1606, 1069 cm^{-1}; ^{1}H NMR (500 MHz, $CDCl_3$) δ 7.90 (d, *J* = 8.0 Hz, 2H), 7.25 (d, *J* = 8.0 Hz, 2H), 3.94-3.83 (m, 2H), 3.76-3.69 (m, 1H), 3.19-3.09 (m, 1H), 3.07-2.98 (m, 1H), 2.65 (t, *J* = 7.5 Hz, 3H), 2.07-1.95 (m, 2H), 1.95-1.83 (m, 3H), 1.65-1.57 (m, 2H), 1.56-1.48 (m, 1H), 1.40-1.31 (m, 2H), 0.93 (t, *J* = 7.5 Hz, 3H); ^{13}C NMR (126 MHz, $CDCl_3$) δ 199.7, 148.5, 134.7, 128.5, 128.2, 78.5, 67.6, 35.6, 35.3, 33.2, 31.4, 30.0, 25.7, 22.3, 13.8; HRMS (ESI-TOF) Calcd for $C_{17}H_{24}O_2Na$ $[M+Na]^+$: 283.1674; Found 283.1669.

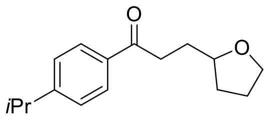

3h

1-(4-Isopropylphenyl)-3-(tetrahydrofuran-2-yl)propan-1-one (3 h). Prepared according to the general procedure B employing Ir[dF(CF_3)ppy]$_2$(phen)PF_6 (3.2 mg, 0.003 mmol), 2-(4-isopropylphenyl)-2-oxoacetic acid (57.7 mg, 0.30 mmol), K_2HPO_4 (0.36 mmol, 62.6 mg), H_2O (6.0 mmol, 108 μL), MeOH (6.0 mmol, 243 μL) and THF (12 mL). After 24 h, the reaction was diluted with EtOAc and passed through a short pad of silica using EtOAc. The filtrate was concentrated in *vacuo* before it was purified by flash chromatography (PE/EA = 5:1) on silica gel to afford **3h** (39.17 mg, 0.159 mmol, 53% yield) as a yellow oil. IR *v* 2961, 2870, 1681, 1606, 1067, 832 cm^{-1}; ^1H NMR (500 MHz, $CDCl_3$) δ 7.92 (d, *J* = 8.0 Hz, 2H), 7.30 (d, *J* = 8.5 Hz, 2H), 3.94-3.83 (m, 2H), 3.77-3.68 (m, 1H), 3.20-3.09 (m, 1H), 3.09-2.91 (m, 2H), 2.07-1.96 (m, 2H), 1.95-1.83 (m, 3H), 1.57-1.48 (m, 1H), 1.27 (d, *J* = 7.0 Hz, 6H); ^{13}C NMR (126 MHz, $CDCl_3$) δ 199.7, 154.3, 134.8, 128.3, 126.6, 78.5, 67.6, 35.3, 34.2, 31.4, 30.0, 25.7, 23.6; HRMS (ESI-TOF) Calcd for $C_{16}H_{22}O_2Na$ $[M+Na]^+$: 269.1517; Found 269.1515.

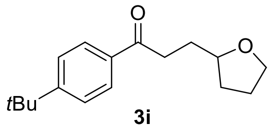

3i

1-(4-(*Tert*-butyl)phenyl)-3-(tetrahydrofuran-2-yl)propan-1-one (3i). Prepared according to the general procedure B employing Ir[dF(CF_3)ppy]$_2$(phen)PF_6 (3.3 mg, 0.003 mmol), 2-(4-(tert-butyl)phenyl)-2-oxoacetic acid (61.5 mg, 0.30 mmol), K_2HPO_4 (0.36 mmol, 62.5 mg), H_2O (6.0 mmol, 108 μL), MeOH (6.0 mmol, 243 μL) and THF (12 mL). After 24 h, the reaction was diluted with EtOAc and passed through a short pad of silica using EtOAc. The filtrate was concentrated in *vacuo* before it was purified by flash chromatography (PE/EA = 10:1) on silica gel to afford **3i** (45.31 mg, 0.174 mmol, 58% yield) as a yellow oil. IR *v* 2962, 2868, 1682, 1607, 1403, 1069 cm^{-1}; ^1H NMR (500 MHz, $CDCl_3$) δ 7.92 (d, *J* = 8.5 Hz, 2H), 7.46 (d, *J* = 8.5 Hz, 2H), 3.95-3.83 (m, 2H), 3.77-3.69 (m, 1H), 3.19-3.10 (m, 1H), 3.08-2.98 (m, 1H), 2.07-1.96 (m, 2H), 1.94-1.83 (m, 3H), 1.57-1.48 (m, 1H), 1.34 (s, 9H); ^{13}C NMR (126 MHz, $CDCl_3$) δ 199.6, 156.5, 134.4, 127.9, 125.4, 78.4, 67.6, 35.3, 35.0, 31.3, 31.0, 30.0, 25.6; HRMS (ESI-TOF) Calcd for $C_{17}H_{24}O_2Na$ $[M+Na]^+$: 283.1674; Found 283.1672.

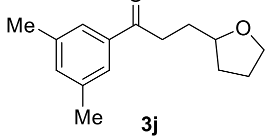

3j

1-(3,5-Dimethylphenyl)-3-(tetrahydrofuran-2-yl)propan-1-one (3j). Prepared according to the general procedure B employing Ir[dF(CF_3)ppy]$_2$(phen)PF_6 (3.3 mg, 0.003 mmol), 2-(3,5-dimethylphenyl)-2-oxoacetic acid (53.46 mg, 0.30 mmol), K_2HPO_4 (0.36 mmol, 62.7 mg), H_2O (6.0

mmol, 108 μL), MeOH (6.0 mmol, 243 μL) and THF (12 mL). After 24 h, the reaction was diluted with EtOAc and passed through a short pad of silica using EtOAc. The filtrate was concentrated in *vacuo* before it was purified by flash chromatography (PE/EA = 10:1) on silica gel to afford **3j** (36.24 mg, 0.156 mmol, 52% yield) as a yellow oil. IR *v* 1637, 1400, 1097 cm^{-1}; ^{1}H NMR (500 MHz, $CDCl_3$) δ 7.59 (s, 2H), 7.18 (s, 1H), 3.94-3.83 (m, 2H), 3.77-3.69 (m, 1H), 3.20-3.11 (m, 1H), 3.06-2.97 (m, 1H), 2.36 (s, 6H), 2.07-1.96 (m, 2H), 1.95-1.82 (m, 3H), 1.58-1.49 (m, 1H); ^{13}C NMR (126 MHz, $CDCl_3$) δ 200.4, 138.1, 137.1, 134.5, 125.8, 78.5, 67.6, 35.5, 31.4, 29.9, 25.7, 21.2; HRMS (ESI-TOF) Calcd for $C_{15}H_{20}O_2Na$ $[M+Na]^+$: 255.1361; Found 255.1360.

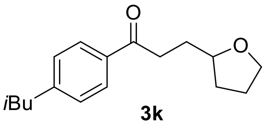

3k

1-(4-Isobutylphenyl)-3-(tetrahydrofuran-2-yl)propan-1-one (3k). Prepared according to the general procedure B employing Ir[dF(CF_3)ppy]$_2$(phen)PF_6 (3.3 mg, 0.003 mmol), 2-(4-isobutylphenyl)-2-oxoacetic acid (61.5 mg, 0.30 mmol), K_2HPO_4 (0.36 mmol, 62.7 mg), H_2O (6.0 mmol, 108 μL), MeOH (6.0 mmol, 243 μL) and THF (12 mL). After 24 h, the reaction was diluted with EtOAc and passed through a short pad of silica using EtOAc. The filtrate was concentrated in *vacuo* before it was purified by flash chromatography (PE/EA = 10:1) on silica gel to afford **3k** (35.93 mg, 0.138 mmol, 46% yield) as a yellow oil. IR *v* 2956, 2926, 2869, 1718, 1682, 1606, 1068 cm^{-1}; ^{1}H NMR (500 MHz, $CDCl_3$) δ 7.90 (d, *J* = 8.0 Hz, 2H), 7.22 (d, *J* = 8.0 Hz, 2H), 3.95-3.82 (m, 2H), 3.77-3.68 (m, 1H), 3.21-3.10 (m, 1H), 3.09-2.98 (m, 1H), 2.52 (d, *J* = 7.5 Hz, 2H), 2.08-1.96 (m, 2H), 1.95-1.82 (m, 4H), 1.57-1.47 (m, 1H), 0.91 (d, *J* = 6.5 Hz, 6H); ^{13}C NMR (126 MHz, $CDCl_3$) δ 199.7, 147.3, 134.7, 129.2, 128.0, 78.5, 67.6, 45.3, 35.3, 31.4, 30.04, 29.97, 25.7, 22.3; HRMS (ESI-TOF) Calcd for $C_{17}H_{24}O_2Na$ $[M+Na]^+$: 283.1674; Found 283.1672.

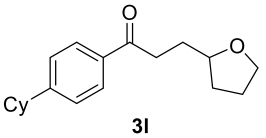

3l

1-(4-Cyclohexylphenyl)-3-(tetrahydrofuran-2-yl)propan-1-one (3l). Prepared according to the general procedure B employing Ir[dF(CF_3)ppy]$_2$(phen)PF_6 (3.3 mg, 0.003 mmol), 2-(4-cyclohexylphenyl)-2-oxoacetic acid (69.68 mg, 0.30 mmol), K_2HPO_4 (0.36 mmol, 62.7 mg), H_2O (6.0 mmol, 108 μL), MeOH (6.0 mmol, 243 μL) and THF (12 mL). After 24 h, the reaction was diluted with EtOAc and passed through a short pad of silica using EtOAc. The filtrate was concentrated in *vacuo* before it was purified by flash chromatography (PE/EA = 10:1) on silica gel to afford **3l** (48.98 mg, 0.171 mmol, 57% yield) as a yellow oil. IR *v* 2924, 2852, 1683, 1606 cm^{-1}; ^{1}H NMR (500 MHz, $CDCl_3$) δ 7.91 (d, *J* = 8.0 Hz, 2H), 7.28 (d, *J* = 8.0 Hz, 2H), 3.94-3.83 (m, 2H),

3.77-3.68 (m, 1H), 3.19-3.09 (m, 1H), 3.07-2.98 (m, 1H), 2.61-2.50 (m, 1H), 2.08-1.96 (m, 2H), 1.95-1.80 (m, 7H), 1.79-1.72 (m, 1H), 1.58-1.48 (m, 1H), 1.48-1.33 (m, 4H), 1.33-1.23 (m, 1H); ^{13}C NMR (126 MHz, $CDCl_3$) δ 199.7, 153.5, 134.8, 128.2, 127.0, 78.5, 67.6, 44.6, 35.3, 34.1, 31.4, 30.0, 26.7, 26.0, 25.7; HRMS (ESI-TOF) Calcd for $C_{19}H_{26}O_2Na$ $[M+Na]^+$: 309.1830; Found 309.1829.

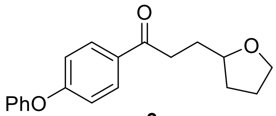
3m

1-(4-Phenoxyphenyl)-3-(tetrahydrofuran-2-yl)propan-1-one (3m). Prepared according to the general procedure B employing Ir[dF(CF_3)ppy]$_2$(phen)PF_6 (3.2 mg, 0.003 mmol), 2-oxo-2-(4-phenoxyphenyl)acetic acid (72.67 mg, 0.30 mmol), K_2HPO_4 (0.36 mmol, 62.7 mg), H_2O (6.0 mmol, 108 μL), MeOH (6.0 mmol, 243 μL) and THF (12 mL). After 24 h, the reaction was diluted with EtOAc and passed through a short pad of silica using EtOAc. The filtrate was concentrated in *vacuo* before it was purified by flash chromatography (PE/EA = 7:1) on silica gel to afford **3m** (36.45 mg, 0.123 mmol, 41% yield) as a yellow oil. IR *v* 2954, 1680, 1585, 1488, 1243 cm^{-1}; ^{1}H NMR (500 MHz, $CDCl_3$) δ 7.95 (d, *J* = 8.5 Hz, 2H), 7.36 (dd, *J* = 8.5, 7.5 Hz, 2H), 7.20-7.14 (m, 1H), 7.04 (dd, *J* = 9.0, 1.5 Hz, 2H), 6.97 (d, *J* = 9.0 Hz, 2H), 3.92-3.81 (m, 2H), 3.74-3.67 (m, 1H), 3.16-3.06 (m, 1H), 3.05-2.96 (m, 1H), 2.06-1.95 (m, 2H), 1.93-1.80 (m, 3H), 1.56-1.46 (m, 1H); ^{13}C NMR (126 MHz, $CDCl_3$) δ 198.5, 161.7, 155.5, 131.6, 130.2, 129.9, 124.4, 120.0, 117.2, 78.4, 67.6, 35.1, 31.3, 30.0, 25.6; HRMS (ESI-TOF) Calcd for $C_{19}H_{20}O_2Na$ $[M+Na]^+$: 319.1310; Found 319.1308.

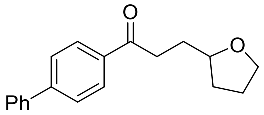
3n

1-([1,1'-Biphenyl]-4-yl)-3-(tetrahydrofuran-2-yl)propan-1-one (3n). Prepared according to the general procedure B employing Ir[dF(CF_3)ppy]$_2$(phen)PF_6 (3.3 mg, 0.003 mmol), 2-([1,1'-biphenyl]-4-yl)-2-oxoacetic acid (67.8 mg, 0.30 mmol), K_2HPO_4 (0.36 mmol, 62.5 mg), H_2O (6.0 mmol, 108 μL), MeOH (6.0 mmol, 243 μL) and THF (12 mL). After 24 h, the reaction was diluted with EtOAc and passed through a short pad of silica using EtOAc. The filtrate was concentrated in *vacuo* before it was purified by flash chromatography (PE/EA = 8:1) on silica gel to afford **3n** (37.01 mg, 0.132 mmol, 44% yield) as a white solid. IR *v* 3449, 2923, 1681, 1603 cm^{-1}; ^{1}H NMR (500 MHz, $CDCl_3$) δ 8.05 (d, *J* = 8.5 Hz, 2H), 7.67 (d, *J* = 8.5 Hz, 2H), 7.62 (d, *J* = 7.5 Hz,, 2H), 7.46 (t, *J* = 7.5 Hz, 2H), 7.42-7.36 (m, 1H), 3.96-3.83 (m, 2H), 3.78-3.69 (m, 1H), 3.24-3.15 (m, 1H), 3.13-3.03 (m, 1H), 2.08-1.98 (m, 2H), 1.97-1.82 (m, 3H), 1.59-1.49 (m, 1H); ^{13}C NMR (126 MHz, $CDCl_3$) δ 199.6,

145.5, 139.9, 135.6, 128.9, 128.6, 128.1, 127.2, 127.1, 78.4, 67.6, 35.4, 31.4, 30.0, 25.7; HRMS (ESI-TOF) Calcd for $C_{19}H_{20}O_2Na$ $[M+Na]^+$: 303.1361; Found 303.1358.

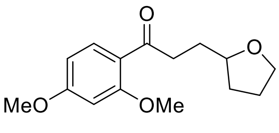

3o

1-(2,4-Dimethoxyphenyl)-3-(tetrahydrofuran-2-yl)propan-1-one (3o). Prepared according to the general procedure B employing Ir[dF(CF_3)ppy]$_2$(phen)PF_6 (3.3 mg, 0.003 mmol), 2-(2,4-dimethoxyphenyl)-2-oxoacetic acid (63.0 mg, 0.30 mmol), K_2HPO_4 (0.36 mmol, 62.6 mg), H_2O (6.0 mmol, 108 μL), MeOH (6.0 mmol, 243 μL) and THF (12 mL). After 24 h, the reaction was diluted with EtOAc and passed through a short pad of silica using EtOAc. The filtrate was concentrated in *vacuo* before it was purified by flash chromatography (PE/EA = 4:1) on silica gel to afford **3o** (38.86 mg, 0.147 mmol, 49% yield) as a yellow oil. IR *v* 2952, 2845, 1662, 1601, 1212 cm^{-1}; ^1H NMR (500 MHz, $CDCl_3$) δ 7.79 (d, *J* = 8.0 Hz, 1H), 6.51 (dd, *J* = 9.0, 2.0 Hz, 1H), 6.45 (d, *J* = 2.0 Hz, 1H), 3.92-3.78 (m, 8H), 3.76-3.67 (m, 1H), 3.15-2.95 (m, 2H), 2.06-1.96 (m, 1H), 1.94-178 (m, 4H), 1.56-1.45 (m, 1H); ^{13}C NMR (126 MHz, $CDCl_3$) δ 200.0, 164.2, 160.6, 132.5, 121.1, 104.9, 98.2, 78.7, 67.5, 55.40, 55.37, 40.4, 31.3, 30.2, 25.7; HRMS (ESI-TOF) Calcd for $C_{15}H_{20}O_4Na$ $[M+Na]^+$: 287.1259; Found 287.1255.

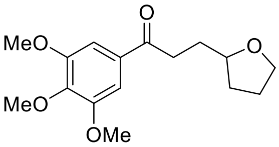

3p

3-(Tetrahydrofuran-2-yl)-1-(3,4,5-trimethoxyphenyl)propan-1-one (3p). Prepared according to the general procedure B employing Ir[dF(CF_3)ppy]$_2$(phen)PF_6 (3.3 mg, 0.003 mmol), 2-oxo-2-(3,4,5-trimethoxyphenyl)acetic acid (72.1 mg, 0.30 mmol), K_2HPO_4 (0.36 mmol, 62.7 mg), H_2O (6.0 mmol, 108 μL), MeOH (6.0 mmol, 243 μL) and THF (12 mL). After 24 h, the reaction was diluted with EtOAc and passed through a short pad of silica using EtOAc. The filtrate was concentrated in *vacuo* before it was purified by flash chromatography (PE/EA = 3:1) on silica gel to afford **3p** (43.3 mg, 0.147 mmol, 49% yield) as a yellow oil. IR *v* 2941, 1679, 1584, 1413, 1127 cm^{-1}; ^1H NMR (500 MHz, $CDCl_3$) δ 7.26 (s, 2H), 3.95-3.84 (m, 11H), 3.77-3.71 (m, 1H), 3.18-3.10 (m, 1H), 3.08-2.99 (m, 1H), 2.08-1.98 (m, 2H), 1.96-1.82 (m, 3H), 1.58-1.49 (m, 1H); ^{13}C NMR (126 MHz, $CDCl_3$) δ 198.8, 152.9, 142.4, 132.2, 105.5, 78.4, 67.6, 60.8, 56.2, 35.2, 31.4, 30.1, 25.7; HRMS (ESI-TOF) Calcd for $C_{16}H_{22}O_5Na$ $[M+Na]^+$: 317.1365; Found 317.1363.

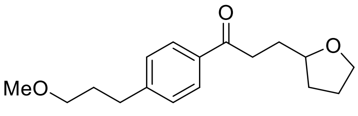

3q

1-(4-(3-Methoxypropyl)phenyl)-3-(tetrahydrofuran-2-yl)propan-1-one (3q). Prepared according to the general procedure B employing Ir[dF(CF_3)ppy]$_2$(phen)PF_6 (3.3 mg,

0.003 mmol), 2-(4-(3-methoxypropyl)phenyl)-2-oxoacetic acid (66.7 mg, 0.30 mmol), K_2HPO_4 (0.36 mmol, 62.7 mg), H_2O (6.0 mmol, 108 μL), MeOH (6.0 mmol, 243 μL) and THF (12 mL). After 24 h, the reaction was diluted with EtOAc and passed through a short pad of silica using EtOAc. The filtrate was concentrated in *vacuo* before it was purified by flash chromatography (PE/EA = 5:1) on silica gel to afford **3q** (45.6 mg, 0.165 mmol, 55% yield) as a yellow oil. IR *v* 2925, 2865, 1707, 1682, 1606, 739 cm^{-1}; ^1H NMR (500 MHz, $CDCl_3$) δ 7.91 (d, *J* = 8.0 Hz, 2H), 7.27 (d, *J* = 8.0 Hz, 2H), 3.94-3.82 (m, 2H), 3.77-3.69 (m, 1H), 3.38 (t, *J* = 6.5 Hz, 2H), 3.34 (s, 3H), 3.19-3.10 (m, 1H), 3.08-2.98 (m, 1H), 2.74 (t, *J* = 7.5 Hz, 2H), 2.07-1.95 (m, 2H), 1.95-1.81 (m, 5H), 1.58-1.47 (m, 1H); ^{13}C NMR (126 MHz, $CDCl_3$) δ 199.7, 147.6, 134.9, 128.6, 128.2, 78.5, 71.6, 67.6, 58.5, 35.3, 32.3, 31.4, 30.8, 30.0, 25.7; HRMS (ESI-TOF) Calcd for $C_{17}H_{24}O_3Na$ $[M+Na]^+$: 299.1623; Found 299.1622.

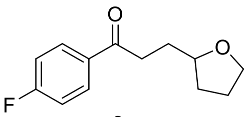

3r

1-(4-Fluorophenyl)-3-(tetrahydrofuran-2-yl)propan-1-one (3r). Prepared according to the general procedure B employing $Ir[dF(CF_3)ppy]_2(phen)PF_6$ (3.3 mg, 0.003 mmol), 2-(4-fluorophenyl)-2-oxoacetic acid (50.4 mg, 0.30 mmol), K_2HPO_4 (0.36 mmol, 62.6 mg), H_2O (6.0 mmol, 108 μL), MeOH (6.0 mmol, 243 μL) and THF (12 mL). After 24 h, the reaction was diluted with EtOAc and passed through a short pad of silica using EtOAc. The filtrate was concentrated in *vacuo* before it was purified by flash chromatography (PE/EA = 10:1) on silica gel to afford **3r** (39.3 mg, 0.177 mmol, 59% yield) as a yellow oil. IR *v* 3000, 1637, 1401, 1071cm^{-1}; ^1H NMR (500 MHz, $CDCl_3$) δ 8.04-7.97 (m, 2H), 7.15-7.07 (m, 2H), 3.94-3.82 (m, 2H), 3.76-3.68 (m, 1H), 3.19-3.09 (m, 1H), 3.08-2.98 (m, 1H), 2.08-1.96 (m, 2H), 1.96-1.80 (m, 3H), 1.57-1.48 (m, 1H); ^{13}C NMR (126 MHz, $CDCl_3$) δ 198.3, 165.6 (d, *J* = 254.8 Hz), 133.4 (d, *J* = 3.0 Hz), 130.6 (d, *J* = 9.2 Hz), 115.5 (d, *J* = 21.8 Hz). 78.3, 67.6, 35.3, 31.4, 29.9, 25.7; ^{19}F NMR (471 MHz, $CDCl_3$) δ -105.7; HRMS (ESI-TOF) Calcd for $C_{13}H_{15}FO_2Na$ $[M+Na]^+$: 245.0954; Found 245.0949.

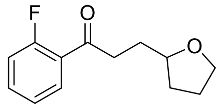

3s

1-(2-Fluorophenyl)-3-(tetrahydrofuran-2-yl)propan-1-one (3s). Prepared according to the general procedure B employing $Ir[dF(CF_3)ppy]_2(phen)PF_6$ (3.3 mg, 0.003 mmol), 2-(2-fluorophenyl)-2-oxoacetic acid (50.5 mg, 0.30 mmol), K_2HPO_4 (0.36 mmol, 62.7 mg), H_2O (6.0 mmol, 108 μL), MeOH (6.0 mmol, 243 μL) and THF (12 mL). After 24 h, the reaction was diluted with EtOAc and passed through a short pad of silica using EtOAc. The filtrate was concentrated in *vacuo* before it was purified by flash

chromatography (PE/EA = 10:1) on silica gel to afford **3s** (30.7 mg, 0.138 mmol, 46% yield) as a yellow oil. IR *v* 2954, 2925, 2867, 1687, 1608, 1406, 1068, 763 cm^{-1}; ^{1}H NMR (500 MHz, $CDCl_3$) δ 7.85 (dd, *J* = 7.5, 6.5 Hz, 1H), 7.54-7.45 (m, 1H), 7.21 (dd, *J* = 7.5, 7.5 Hz, 1H), 7.16-7.07 (m, 1H), 3.96-3.80 (m, 2H), 3.77-3.66 (m, 1H), 3.22-2.98 (m, 2H), 2.09-1.82 (m, 5H), 1.58-1.45 (m, 1H); ^{13}C NMR (126 MHz, $CDCl_3$) δ 198.3 (d, *J* = 4.0 Hz), 161.7 (d, *J* = 255.0 Hz), 134.2 (d, *J* = 8.9 Hz), 130.5 (d, *J* = 2.6 Hz), 125.8 (d, *J* = 13.1 Hz), 124.3 (d, *J* = 3.4 Hz), 116.5 (d, *J* = 23.8 Hz), 78.3, 67.6, 40.2 (d, *J* = 7.0 Hz), 31.3, 29.6 (d, *J* = 1.6 Hz), 25.6; ^{19}F NMR (471 MHz, $CDCl_3$) δ -109.5; HRMS (ESI-TOF) Calcd for $C_{13}H_{15}FO_2Na$ $[M+Na]^+$: 245.0954; Found 245.0951.

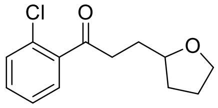
3t

1-(2-Chlorophenyl)-3-(tetrahydrofuran-2-yl)propan-1-one (**3t**). Prepared according to the general procedure B employing Ir[dF(CF_3)ppy]$_2$(phen)PF_6 (3.3 mg, 0.003 mmol), 2-(2-chlorophenyl)-2-oxoacetic acid (55.4 mg, 0.30 mmol), K_2HPO_4 (0.36 mmol, 62.7 mg), H_2O (6.0 mmol, 108 μL), MeOH (6.0 mmol, 243 μL) and THF (12 mL). After 24 h, the reaction was diluted with EtOAc and passed through a short pad of silica using EtOAc. The filtrate was concentrated in *vacuo* before it was purified by flash chromatography (PE/EA = 10:1) on silica gel to afford **3t** (30.1 mg, 0.126 mmol, 42% yield) as a yellow oil. IR *v* 2955, 2922, 2867, 1700, 1589, 1066, 755 cm^{-1}; ^{1}H NMR (500 MHz, $CDCl_3$) δ 7.47 (d, *J* = 7.5 Hz, 1H), 7.42-7.34 (m, 2H), 7.33-7.28 (m, 1H), 3.93-3.80 (m, 2H), 3.75-3.67 (m, 1H), 3.14-2.96 (m, 2H), 2.06-1.94 (m, 2H), 1.93-1.82 (m, 3H), 1.56-1.46 (m, 1H); ^{13}C NMR (126 MHz, $CDCl_3$) δ 203.2, 139.5, 131.5, 130.7, 130.4, 128.8, 126.8, 78.2, 67.6, 39.7, 31.3, 29.9, 25.7; HRMS (ESI-TOF) Calcd for $C_{13}H_{15}ClO_2Na$ $[M+Na]^+$: 261.0658; Found 261.0655.

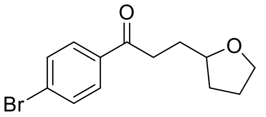
3u

1-(4-Bromophenyl)-3-(tetrahydrofuran-2-yl)propan-1-one (3u). Prepared according to the general procedure B employing Ir[dF(CF_3)ppy]$_2$(phen)PF_6 (3.3 mg, 0.003 mmol), 2-(4-bromophenyl)-2-oxoacetic acid (68.7 mg, 0.30 mmol), K_2HPO_4 (0.36 mmol, 62.6 mg), H_2O (6.0 mmol, 108 μL), MeOH (6.0 mmol, 243 μL) and THF (12 mL). After 24 h, the reaction was diluted with EtOAc and passed through a short pad of silica using EtOAc. The filtrate was concentrated in *vacuo* before it was purified by flash chromatography (PE/EA = 10:1) on silica gel to afford **3u** (27.2 mg, 0.096 mmol, 32% yield) as a yellow oil. IR *v* 2954, 1637, 1400, 558 cm^{-1}; ^{1}H NMR (500 MHz, $CDCl_3$) δ 7.84 (d, *J* = 8.5 Hz, 2H), 7.59 (d, *J* = 8.5 Hz, 2H), 3.95-3.80 (m, 2H), 3.77-3.65 (m, 1H), 3.19-3.08 (m, 1H), 3.07-2.95 (m, 1H), 2.08-1.96 (m, 2H), 1.95-1.79 (m, 3H), 1.57-1.46 (m, 1H); ^{13}C NMR

(126 MHz, $CDCl_3$) δ 198.9, 135.7, 131.8, 129.6, 128.0, 78.5, 67.6, 35.4, 31.4, 29.9, 25.7; HRMS (ESI-TOF) Calcd for $C_{13}H_{15}BrO_2Na$ $[M+Na]^+$: 305.0153; Found 305.0147.

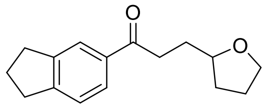

3v

1-(2,3-Dihydro-1*H*-inden-5-yl)-3-(tetrahydrofuran-2-yl)propan-1-one. Prepared according to the general procedure B employing Ir[dF(CF_3)ppy]$_2$(phen)PF_6 (3.3 mg, 0.003 mmol), 2-(2,3-dihydro-1*H*-inden-5-yl)-2-oxoacetic acid (57.2 mg, 0.30 mmol), K_2HPO_4 (0.36 mmol, 62.8 mg), H_2O (6.0 mmol, 108 μL), MeOH (6.0 mmol, 243 μL) and THF (12 mL). After 24 h, the reaction was diluted with EtOAc and passed through a short pad of silica using EtOAc. The filtrate was concentrated in *vacuo* before it was purified by flash chromatography (PE/EA = 8:1) on silica gel to afford **3v** (45.4 mg, 0.186 mmol, 62% yield) as a yellow oil. IR *v* 1637, 1400, 1069 cm^{-1}; ^1H NMR (500 MHz, $CDCl_3$) δ 7.83 (s, 1H), 7.77 (d, J = 8.0 Hz, 1H), 7.27 (d, J = 7.5 Hz, 1H), 3.94-3.82 (m, 2H), 3.76-3.68 (m, 1H), 3.19-3.09 (m, 1H), 3.06-2.98 (m, 1H), 2.93 (t, J = 7.5 Hz, 4H), 2.14-2.06 (m, 2H), 2.05-1.95 (m, 2H), 1.94-1.82 (m, 3H), 1.57-1.47 (m, 1H); ^{13}C NMR (126 MHz, $CDCl_3$) δ 199.9, 149.9, 144.6, 135.4, 126.5, 124.2, 123.9, 78.5, 67.6, 35.4, 32.9, 32.5, 31.3, 30.0, 25.6, 25.3; HRMS (ESI-TOF) Calcd for $C_{16}H_{20}O_2Na$ $[M+Na]^+$: 267.1361; Found 267.1356.

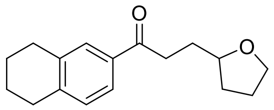

3w

3-(Tetrahydrofuran-2-yl)-1-(5,6,7,8-tetrahydronaphthalen-2-yl)propan-1-one (3w). **Lsd-02-151:** Prepared according to the general procedure B employing Ir[dF(CF_3)ppy]$_2$(phen)PF_6 (3.3 mg, 0.003 mmol), 2-oxo-2-(5,6,7,8-tetrahydronaphthalen-2-yl)acetic acid (61.3 mg, 0.30 mmol), K_2HPO_4 (0.36 mmol, 62.6 mg), H_2O (6.0 mmol, 108 μL), MeOH (6.0 mmol, 243 μL) and THF (12 mL). After 24 h, the reaction was diluted with EtOAc and passed through a short pad of silica using EtOAc. The filtrate was concentrated in *vacuo* before it was purified by flash chromatography (PE/EA = 10:1) on silica gel to afford **3w** (40.3 mg, 0.156 mmol, 52% yield) as a yellow oil. IR *v* 2928, 2860, 1680, 1605, 1068, 830 cm^{-1}; ^1H NMR (500 MHz, $CDCl_3$) δ 7.72-7.65 (m, 2H), 7.12 (d, J = 8.5 Hz, 1H), 3.93-3.82 (m, 2H), 3.76-3.69 (m, 1H), 3.18-3.09 (m, 1H), 3.05-2.95 (m, 1H), 2.86-2.74 (m, 4H), 2.06-1.95 (m, 2H), 1.94-1.84 (m, 3H), 1.83-1.76 (m, 4H), 1.56-1.47 (m, 1H); ^{13}C NMR (125 MHz, $CDCl_3$) δ 199.9, 142.9, 137.3, 134.5, 129.2, 128.9, 125.1, 78.5, 67.6, 35.3, 31.4, 30.0, 29.5, 29.3, 25.7, 22.9, 22.8; HRMS (ESI-TOF) Calcd for $C_{17}H_{22}O_2Na$ $[M+Na]^+$: 281.1517; Found 281.1515.

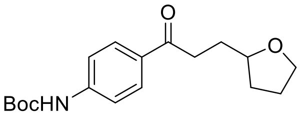

3x

Tert-butyl (4-(3-(tetrahydrofuran-2-yl)propanoyl)phenyl)carbamate (3x). Prepared according to the general procedure B employing Ir[dF(CF_3)ppy]$_2$(phen)PF_6 (3.4 mg, 0.003 mmol), 2-(4-((*tert*-butoxycarbonyl)amino)phenyl)-2-oxoacetic acid (79.5 mg, 0.30 mmol), K_2HPO_4 (0.36 mmol, 62.7 mg), H_2O (6.0 mmol, 108 μL), MeOH (6.0 mmol, 243 μL) and THF (12 mL). After 24 h, the reaction was diluted with EtOAc and passed through a short pad of silica using EtOAc. The filtrate was concentrated in *vacuo* before it was purified by flash chromatography (PE/EA = 3/2) on silica gel to afford **3x** (49.8 mg, 0.156 mmol, 52% yield) as a yellow oil. IR *v* 2930, 1730, 1675, 1590, 1529, 1156 cm^{-1}; ^{1}H NMR (500 MHz, CDCl3) δ 7.91 (d, *J* = 8.5 Hz, 2H), 7.45 (d, *J* = 8.5 Hz, 2H), 7.13 (s, 1H), 3.96-3.81 (m, 2H), 3.78-3.69 (m, 1H), 3.16-3.06 (m, 1H), 3.05-2.95 (m, 1H), 2.07-1.95 (m, 2H), 1.94-1.82 (m, 3H), 1.57-1.48 (m, 10H). ^{13}C NMR (125 MHz, $CDCl_3$) δ 198.7, 152.2, 142.9, 131.4, 129.4, 117.4, 81.0, 78.5, 67.6, 35.0, 31.4, 30.0, 28.2, 25.6; HRMS (ESI-TOF) Calcd for $C_{18}H_{25}NO_4Na$ [M+Na]$^+$: 342.1681; Found 342.1682.

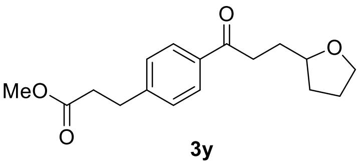

3y

Methyl 3-(4-(3-(tetrahydrofuran-2-yl)propanoyl)phenyl)propanoate (3y). Prepared according to the general procedure B employing Ir[dF(CF_3)ppy]$_2$(phen)PF_6 (3.4 mg, 0.003 mmol), 2-(4-(3-methoxy-3-oxopropyl)phenyl)-2-oxoacetic acid (70.9 mg, 0.30 mmol), K_2HPO_4 (0.36 mmol, 62.5 mg), H_2O (6.0 mmol, 108 μL), MeOH (6.0 mmol, 243 μL) and THF (12 mL). After 24 h, the reaction was diluted with EtOAc and passed through a short pad of silica using EtOAc. The filtrate was concentrated in *vacuo* before it was purified by flash chromatography (PE/EA = 4:1) on silica gel to afford **3y** (43.6 mg, 0.150 mmol, 50% yield) as a yellow oil. IR *v* 3458, 2954, 1736, 1678, 1609 cm^{-1}; ^{1}H NMR (500 MHz, $CDCl_3$) δ 7.91 (d, *J* = 8.5 Hz, 2H), 7.28 (d, *J* = 8.5 Hz, 2H), 3.93-3.82 (m, 2H), 3.76-3.69 (m, 1H), 3.67 (s, 3H), 3.18-3.10 (m, 1H), 3.07-2.96 (m, 3H), 2.65 (t, *J* = 8.0 Hz, 2H), 2.07-1.95 (m, 2H), 1.94-1.83 (m, 3H), 1.56-1.48 (m, 1H); ^{13}C NMR (125 MHz, $CDCl_3$) δ 199.5, 172.8, 145.9, 135.2, 128.4, 128.3, 78.4, 67.6, 51.6, 35.3, 35.0, 31.3, 30.7, 29.9, 25.6; HRMS (ESI-TOF) Calcd for $C_{17}H_{22}O_4Na$ [M+Na]$^+$: 313.1416; Found 313.1411.

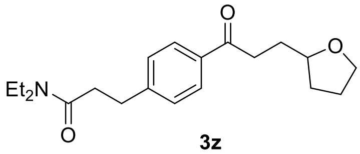

3z

***N*,*N*-diethyl-3-(4-(3-(tetrahydrofuran-2-yl)propanoyl)phenyl)propanamide (3z)**. Prepared according to the general procedure B employing Ir[dF(CF_3)ppy]$_2$(phen)PF_6 (3.3 mg, 0.003 mmol), 2-(4-(3-(diethylamino)-3-oxopropyl)phenyl)-2-oxoacetic acid (83.3 mg, 0.30 mmol), K_2HPO_4 (0.36 mmol, 62.5 mg), H_2O (6.0 mmol, 108 μL), MeOH (6.0 mmol, 243 μL) and THF (12 mL). After 24 h, the reaction was diluted with EtOAc and passed through a short pad of silica using EtOAc. The filtrate was concentrated in *vacuo* before it was purified by flash chromatography (PE/EA = 3:5) on silica gel to afford **3z** (44.7 mg, 0.135 mmol, 45% yield) as a yellow oil. IR *v* 2968, 2930, 2871,1680, 1642 cm^{-1}; ^{1}H NMR (500 MHz, $CDCl_3$) δ 7.91 (d, *J* = 8.0 Hz, 2H), 7.31 (d, *J* = 8.0 Hz, 2H), 3.94-3.82 (m, 2H), 3.76-3.69 (m, 1H), 3.38 (q, *J* = 8.0 Hz, 2H), 3.23 (q, *J* = 7.0 Hz, 2H), 3.18-3.10 (m, 1H), 3.08-2.99 (m, 3H), 2.61 (t, *J* = 7.5 Hz, 2H), 2.07-1.95 (m, 2H), 1.94-1.82 (m, 3H), 1.57-1.48 (m, 1H), 1.11 (t, *J* = 7.0 Hz, 6H); ^{13}C NMR (125 MHz, $CDCl_3$) δ 199.6, 170.6, 147.1, 135.0, 128.6, 128.3, 78.4, 67.6, 41.8, 40.2, 35.3, 34.3, 31.4, 31.3, 29.9, 25.6, 14.2, 13.0; HRMS (ESI-TOF) Calcd for $C_{20}H_{29}NO_3Na$ [M+Na]$^+$: 354.2045; Found 354.2042.

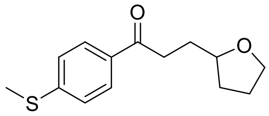

3aa

1-(4-(Methylthio)phenyl)-3-(tetrahydrofuran-2-yl)propan-1-one (3aa). Prepared according to the general procedure B employing Ir[dF(CF_3)ppy]$_2$(phen)PF_6 (3.4 mg, 0.003 mmol), 2-(4-(methylthio)phenyl)-2-oxoacetic acid (58.9 mg, 0.30 mmol), K_2HPO_4 (0.36 mmol, 62.6 mg), H_2O (6.0 mmol, 108 μL), MeOH (6.0 mmol, 243 μL) and THF (12 mL). After 24 h, the reaction was diluted with EtOAc and passed through a short pad of silica using EtOAc. The filtrate was concentrated in *vacuo* before it was purified by flash chromatography (PE/EA = 8:1) on silica gel to afford **3aa** (39.8 mg, 0.159 mmol, 53% yield) as a yellow oil. IR *v* 2962, 2853, 1675, 1638, 1400 cm^{-1}; ^{1}H NMR (500 MHz, $CDCl_3$) δ 7.89 (d, *J* = 8.5Hz, 2H), 7.25 (d, *J* = 8.5 Hz, 2H), 3.94-3.82 (m, 2H), 3.77-3.68 (m, 1H), 3.17-3.07 (m, 1H), 3.05-2.96 (m, 1H), 2.51 (s, 3H), 2.07-1.95 (m, 2H), 1.94-1.82 (m, 3H), 1.57-1.49 (m, 1H); ^{13}C NMR (125 MHz, $CDCl_3$) δ 199.0, 145.5, 133.3, 128.4, 124.9, 78.4, 67.6, 35.2, 31.4, 30.0, 25.7, 14.7; HRMS (ESI-TOF) Calcd for $C_{14}H_{18}O_2SNa$ [M+Na]$^+$: 273.0925; Found 273.0921.

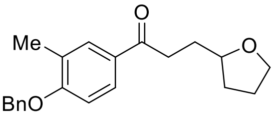

3ab

1-(4-(Benzyloxy)-3-methylphenyl)-3-(tetrahydrofuran-2-yl)propan-1-one (3ab). Prepared according to the general procedure B employing Ir[dF(CF_3)ppy]$_2$(phen)PF_6 (3.5 mg, 0.003 mmol), 2-(4-(benzyloxy)-3-

methylphenyl)-2-oxoacetic acid (81.2 mg, 0.30 mmol), K_2HPO_4 (0.36 mmol, 62.8 mg), H_2O (6.0 mmol, 108 μL), MeOH (6.0 mmol, 243 μL) and THF (12 mL). After 24 h, the reaction was diluted with EtOAc and passed through a short pad of silica using EtOAc. The filtrate was concentrated in vacuo before it was purified by flash chromatography (PE/EA = 6:1) on silica gel to afford **3ab** (59.4 mg, 0.183 mmol, 61% yield) as a yellow solid. mp = 67-68 °C; IR *v* 2924, 2867, 1674, 1600, 1501, 1262, 1135, 738 cm^{-1}; ^{1}H NMR (500 MHz, $CDCl_3$) δ 7.85-7.80 (m, 2H), 7.45-7.35 (m, 4H), 7.35-7.29 (m, 1H), 6.88 (d, *J* = 9.5 Hz, 1H), 5.13 (s, 2H), 3.93-3.82 (m, 2H), 3.75-3.69 (m, 1H), 3.15-3.06 (m, 1H), 3.03-2.93 (m, 1H), 2.30 (s, 3H), 2.05-1.94 (m, 2H), 1.93-1.81 (m, 3H), 1.56-1.47 (m, 1H); ^{13}C NMR (125 MHz, $CDCl_3$) δ 198.8, 160.6, 136.6, 130.8, 129.7, 128.5, 128.0, 127.9, 127.0, 110.4, 78.5, 69.8, 67.6, 35.0, 31.4, 30.1, 25.6, 16.3; HRMS (ESI-TOF) Calcd for $C_{21}H_{24}O_3Na$ [M+Na]$^+$: 347.1623; Found 347.1621.

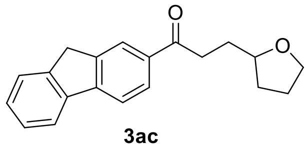

3ac

1-(9H-fluoren-2-yl)-3-(tetrahydrofuran-2-yl)propan-1-one (3ac). Prepared according to the general procedure B employing Ir[dF(CF_3)ppy]$_2$(phen)PF_6 (3.6 mg, 0.003 mmol), 2-(9*H*-fluoren-2-yl)-2-oxoacetic acid (71.5 mg, 0.30 mmol), K_2HPO_4 (0.36 mmol, 62.6 mg), H_2O (6.0 mmol, 108 μL), MeOH (6.0 mmol, 243 μL) and THF (12 mL). After 24 h, the reaction was diluted with EtOAc and passed through a short pad of silica using EtOAc. The filtrate was concentrated in *vacuo* before it was purified by flash chromatography (PE/EA = 10:1) on silica gel to afford **3ac** (33.3 mg, 0.114 mmol, 38% yield) as a yellow oil. IR *v* 2923, 1717, 1677, 1609, 1066, 736 cm^{-1}; ^{1}H NMR (500 MHz, $CDCl_3$) δ 8.14 (s, 1H), 8.01 (d, *J* = 8.0 Hz, 1H), 7.84-7.75 (m, 2H), 7.55 (d, *J* = 7.0 Hz, 1H), 7.42-7.31 (m, 2H), 3.96-3.84 (m, 4H), 3.76-3.70 (m, 1H), 3.25-3.16 (m, 1H), 3.12-3.03 (m, 1H), 2.08-1.99 (m, 2H), 1.96-1.84 (m, 3H), 1.59-1.48 (m, 1H); ^{13}C NMR (126 MHz, $CDCl_3$) δ 199.8, 146.2, 144.4, 143.2, 140.5, 135.4, 127.9, 127.3, 126.9, 125.2, 124.7, 120.8, 119.6, 78.5, 67.6, 36.8, 35.5, 31.4, 30.1, 25.7; HRMS (ESI-TOF) Calcd for $C_{20}H_{20}O_2Na$ [M+Na]$^+$: 315.1361; Found 315.1358.

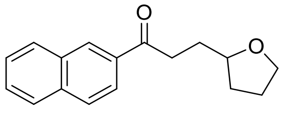

3ad

1-(Naphthalen-2-yl)-3-(tetrahydrofuran-2-yl)propan-1-one (3ad). Prepared according to the general procedure B employing Ir[dF(CF_3)ppy]$_2$(phen)PF_6 (3.7 mg, 0.003 mmol), 2-(naphthalen-2-yl)-2-oxoacetic acid (60.0 mg, 0.30 mmol), K_2HPO_4 (0.36 mmol, 62.8 mg), H_2O (6.0 mmol, 108 μL), MeOH (6.0 mmol, 243 μL) and THF (12 mL). After 24 h, the reaction was diluted with EtOAc and passed through a short pad of silica using EtOAc. The filtrate was concentrated in *vacuo* before it

was purified by flash chromatography (PE/EA = 10:1) on silica gel to afford **3ad** (13.7 mg, 0.054 mmol, 18% yield) as a yellow oil. IR *v* 2955, 2922, 2851, 1677, 1627, 1463, 1065, 742 cm^{-1}; ^1H NMR (500 MHz, $CDCl_3$) δ 8.50 (s, 1H), 8.04 (dd, *J* = 8.5, 1.5 Hz, 1H), 7.94 (d, *J* = 8.0 Hz, 1H), 7.86 (dd, *J* = 9.0, 8.5 Hz, 2H), 7.63-7.49 (m, 2H), 3.99-3.84 (m, 2H), 3.78-3.71 (m, 1H), 3.36-3.26 (m, 1H), 3.22-3.13 (m, 1H), 2.12-2.00 (m, 2H), 1.98-1.84 (m, 3H), 1.60-1.50 (m, 1H); ^{13}C NMR (126 MHz, $CDCl_3$) δ 200.0, 135.5, 134.3, 132.5, 129.7, 129.6, 128.4, 128.3, 127.7, 126.7, 123.9, 78.6, 67.7, 35.6, 31.5, 30.1, 25.8; HRMS (ESI-TOF) Calcd for $C_{17}H_{18}O_2Na$ $[M+Na]^+$: 277.1204; Found 277.1202.

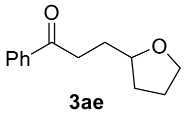

3ae

1-Phenyl-3-(tetrahydrofuran-2-yl)propan-1-one (3ae).[19] Prepared according to the general procedure B employing $Ir[dF(CF_3)ppy]_2(phen)PF_6$ (3.5 mg, 0.003 mmol), benzoylformic acid (45.2 mg, 0.30 mmol), K_2HPO_4 (0.36 mmol, 62.9 mg), H_2O (6.0 mmol, 108 μL), MeOH (6.0 mmol, 243 μL) and THF (12 mL). After 24 h, the reaction was diluted with EtOAc and passed through a short pad of silica using EtOAc. The filtrate was concentrated in *vacuo* before it was purified by flash chromatography (PE/EA = 10:1) on silica gel to afford **3ae** (42.3 mg, 0.207 mmol, 69% yield) as a colorless oil. 1H NMR (500 MHz, CDCl3) δ 7.98 (d, *J* = 8.0 Hz, 2H), 7.54 (t, *J* = 7.0 Hz, 1H), 7.45 (t, *J* = 7.0 Hz, 2H), 3.95-3.82 (m, 2H), 3.77-3.68 (m, 1H), 3.22-3.13 (m, 1H), 3.10-3.01 (m, 1H), 2.08-1.96 (m, 2H), 1.96-1.81 (m, 3H), 1.58-1.47 (m, 1H); ^{13}C NMR (126 MHz, $CDCl_3$) δ 200.1, 137.0, 132.9, 128.5, 128.0, 78.5, 67.7, 35.4, 31.4, 29.9, 25.7.

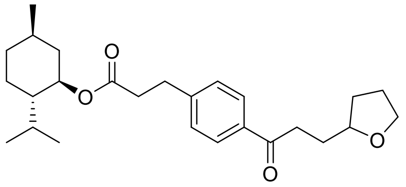

3af

(*1R,2S,5R*)-2-isopropyl-5-methylcyclohexyl 3-(4-(3-(tetrahydrofuran-2-yl)propanoyl)phenyl)propanoate (3af). Prepared according to the general procedure B employing $Ir[dF(CF_3)ppy]_2(phen)PF_6$ (3.6 mg, 0.003 mmol), **1w** (108.0 mg, 0.30 mmol), K_2HPO_4 (0.36 mmol, 62.8 mg), H_2O (6.0 mmol, 108 μL), MeOH (6.0 mmol, 243 μL) and THF (12 mL). After 24 h, the reaction was diluted with EtOAc and passed through a short pad of silica using EtOAc. The filtrate was concentrated in *vacuo* before it was purified by flash chromatography (PE/EA = 5:1) on silica gel to afford **3af** (49.8 mg, 0.120 mmol, 40% yield) as a yellow oil. IR *v* 2955, 2924, 2868, 1729, 1685, 1455, 1180, 983 cm^{-1}; ^1H NMR (500 MHz, $CDCl_3$) δ 7.90 (d, *J* = 8.5 Hz, 2H), 7.29 (d, *J* = 8.0 Hz, 2H), 4.67 (td, *J* = 11.0, 4.5 Hz, 1H),

3.94-3.82 (m, 2H), 3.76-3.68 (m, 1H), 3.18-3.09 (m, 1H), 3.07-2.95 (m, 3H), 2.63 (t, J = 7.5 Hz, 2H), 2.07-1.96 (m, 2H), 1.95-1.82 (m, 4H), 1.80-1.59 (m, 4H), 1.57-1.40 (m, 2H), 1.37-1.27 (m, 1H), 1.08-0.97 (m, 1H), 0.96-0.90 (m, 1H), 0.89 (d, J = 6.5 Hz, 3H), 0.83 (d, J = 7.0 Hz, 3H), 0.69 (d, J = 7.0 Hz, 3H); ^{13}C NMR (126 MHz, $CDCl_3$) δ 199.6, 172.0, 146.0, 135.4, 128.5, 128.3, 78.5, 74.4, 67.6, 46.9, 40.8, 35.6, 35.3, 34.2, 31.4, 31.3, 30.9, 30.0, 26.1, 25.7, 23.3, 21.9, 20.7, 16.2; HRMS (ESI-TOF) Calcd for $C_{26}H_{38}O_4Na$ $[M+Na]^+$: 437.2668; Found 437.2664.

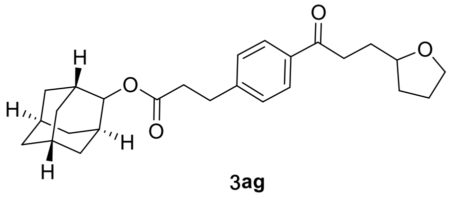

3ag

(1*r*,3*r*,5*r*,7*r*)-adamantan-2-yl 3-(4-(3-(tetrahydrofuran-2-yl)propanoyl)phenyl)propanoate (3ag). Prepared according to the general procedure B employing Ir[dF(CF_3)ppy]$_2$(phen)PF_6 (3.7 mg, 0.003 mmol), 2-(4-(3-(((1*r*,3*r*,5*r*,7*r*)-adamantan-2-yl)oxy)-3-oxopropyl)phenyl)-2-oxoacetic acid **1v** (106.9 mg, 0.30 mmol), K_2HPO_4 (0.36 mmol, 62.6 mg), H_2O (6.0 mmol, 108 μL), MeOH (6.0 mmol, 243 μL) and THF (12 mL). After 24 h, the reaction was diluted with EtOAc and passed through a short pad of silica using EtOAc. The filtrate was concentrated in *vacuo* before it was purified by flash chromatography (PE/EA = 5:1) on silica gel to afford **3ag** (67.7 mg, 0.165 mmol, 55% yield) as a yellow oil. IR *v* 2921, 1728, 1682, 1639, 1401 cm^{-1}; ^{1}H NMR (500 MHz, $CDCl_3$) δ 7.96 (d, J = 8.0 Hz, 0.44H) 7.90 (d, J = 8.5 Hz, 1.56H), 7.35-7.26 (m, 2H), 4.95-4.89 (m, 1H), 3.97-3.81 (m, 2H), 3.76-3.67 (m, 0.86H), 3.44-3.40 (s, 0.24 H), 3.19-3.09 (m, 1H), 3.08-2.97 (m, 3H), 2.74-2.64 (m, 2H), 2.07-1.64 (m, 19H), 1.58-1.45 (m, 3H); ^{13}C NMR (126 MHz, $CDCl_3$) (major) δ 199.6, 171.9, 146.1, 135.2, 128.4, 128.3, 78.5, 67.6, 37.3, 36.3, 35.7, 35.4, 31.8, 31.7, 31.4, 31.0, 30.0, 27.1, 26.9, 25.7; HRMS (ESI-TOF) Calcd for $C_{26}H_{34}O_4Na$ $[M+Na]^+$: 433.2355; Found 433.2350.

<u>Exploration of Different Cyclic Molecules Bearing With Weak C-H bonds with Acetylene</u>

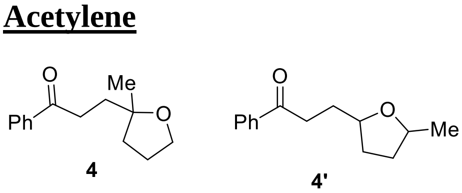

4 4'

3-(2-Methyltetrahydrofuran-2-yl)-1-phenylpropan-1-one (4); 3-(5-methyltetrahydrofuran-2-yl)-1-phenylpropan-1-one (4′). Prepared according to the modified general procedure B employing Ir[dF(CF_3)ppy]$_2$(phen)PF_6 (3.6 mg, 0.003 mmol), benzoylformic acid (45.1 mg, 0.30 mmol), K_2HPO_4 (0.36 mmol, 62.7 mg), H_2O (6.0 mmol, 108 μL) and 2-methyltetrahydrofuran (12 mL). After 24 h, the reaction was diluted with EtOAc and passed

through a short pad of silica using EtOAc. The filtrate was concentrated in *vacuo* before it was purified by flash chromatography (PE/EA = 10:1) on silica gel to afford a mixture of **4** and **4′** (total: 29.5 mg, 0.135 mmol, 45% yield, **4/4′** = 4/1) as a yellow oil. IR *v* 2921, 1728, 1682, 1639, 1401 cm^{-1}; ^1H NMR (500 MHz, $CDCl_3$) δ 8.00-7.94 (m, 2H), 7.56-7.49 (m, 1H), 7.47-7.40 (m, 2H), 4.12-4.04 (m, 0.36H), 3.98-3.75 (m, 1.68H), 3.21-2.98 (m, 2H), 2.13-1.84 (m, 4H), 1.82-1.66 (m, 1.45H), 1.62-1.53 (m, 0.32H), 1.51-1.41 (m, 0.32H), 1.24 (s, 2.32H), 1.20 (d, *J* = 6.0 Hz, 0.59H); ^{13}C NMR (126 MHz, $CDCl_3$) δ 200.1, 199.79, 199.76, 136.83, 136.81, 136.79, 132.6, 128.30, 128.27, 127.8, 81.6, 78.3, 77.7, 75.2, 74.2, 67.0, 37.1, 35.3, 35.1, 34.6, 33.7, 33.6, 32.7, 32.1, 31.2, 30.3, 30.2, 25.8, 25.5, 21.2, 21.1; HRMS (ESI-TOF) Calcd for $C_{14}H_{18}O_2Na$ $[M+Na]^+$: 241.1204; Found 241.1199.

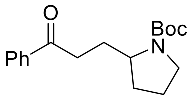

5

***Tert*-butyl 2-(3-oxo-3-phenylpropyl)pyrrolidine-1-carboxylate (5)**. [19] Prepared according to the modified general procedure B employing $Ir[dF(CF_3)ppy]_2(phen)PF_6$ (3.6 mg, 0.003 mmol), benzoylformic acid (45.0 mg, 0.30 mmol), K_2HPO_4 (0.36 mmol, 62.6 mg), H_2O (6.0 mmol, 108 μL), *N*-Boc pyrrolidine (256.1 mg, 1.50 mmol) and 1,2-Dichloroethane (12 mL). After 24 h, the reaction was diluted with EtOAc and passed through a short pad of silica using EtOAc. The filtrate was concentrated in *vacuo* before it was purified by flash chromatography (PE/EA = 7:1) on silica gel to afford **5** (28.2 mg, 0.093 mmol, 31% yield) as a yellow oil. ^1H NMR (500 MHz, $CDCl_3$) δ 7.96 (d, *J* = 8.0 Hz, 2H), 7.59-7.38 (m, 3H), 4.04-3.82 (m, 1H), 3.56-3.23 (m, 2H), 3.16-2.88 (m, 2H), 2.15-1.76 (m, 5H), 1.75-1.61 (m, 1H), 1.43 (s, 9H); ^{13}C NMR (126 MHz, $CDCl_3$) δ 199.8 (199.5), 154.7, 136.8, 132.8, 128.4, 127.9, 79.1 (78.8), 56.6, 46.2 (45.9), 35.6 (35.2), 30.8 (30.3), 29.1, 28.3, 23.6 (22.9).

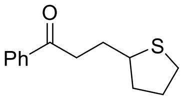

6

1-Phenyl-3-(tetrahydrothiophen-2-yl)propan-1-one (6). Prepared according to the modified general procedure B employing $Ir[dF(CF_3)ppy]_2(phen)PF_6$ (3.7 mg, 0.003 mmol), benzoylformic acid (45.1 mg, 0.30 mmol), K_2HPO_4 (0.36 mmol, 62.6 mg), H_2O (3.0 mmol, 54 μL), tetrahydrothiophene (132.6 mg, 1.50 mmol) and MeCN (6 mL). After 24 h, the reaction was diluted with EtOAc and passed through a short pad of silica using EtOAc. The filtrate was concentrated in *vacuo* before it was purified by flash chromatography (PE/EA = 15:1) on silica gel to afford **6** (19.8 mg, 0.090 mmol, 30% yield) as a yellow oil. IR 2924, 2849, 1685, 1596, 987 cm^{-1}; ^1H NMR (500 MHz, $CDCl_3$) δ 8.00 -7.92 (m, 2H), 7.60-7.54 (m, 1H), 7.47 (t, *J* = 8.0 Hz, 2H), 3.45-3.35 (m, 1H), 3.14 (dd, *J* = 16.5, 5.5 Hz, 1H), 3.06 (dd, *J* = 16.5, 8.0 Hz, 1H), 2.80-2.72 (m, 1H), 2.65-2.58 (m, 1H), 2.16-2.08 (m, 1H), 1.97-1.91 (m,

1H), 1.89-1.80 (m, 1H), 1.68-1.56 (m, 1H), 1.52-1.38 (m, 2H); ^{13}C NMR (126 MHz, CDCl$_3$) δ 197.7, 136.9, 133.2, 128.6, 128.1, 44.4, 37.6, 34.3, 29.4, 26.9, 25.8; HRMS (ESI-TOF) Calcd for $C_{13}H_{16}OSNa$ [M+Na]$^+$: 243.0820; Found 243.0815.

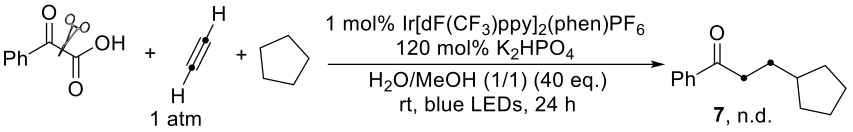

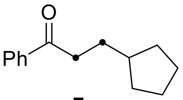
7

3-Cyclopentyl-1-phenylpropan-1-one (7). Prepared according to the modified general procedure B employing Ir[dF(CF$_3$)ppy]$_2$(phen)PF$_6$ (3.7 mg, 0.003 mmol), benzoylformic acid (45.0 mg, 0.30 mmol), K_2HPO_4 (0.36 mmol, 63.0 mg), H_2O (6.0 mmol, 108 μL), MeOH (6.0 mmol, 243 μL) and cyclopentane (12 mL). After 24 h, the reaction was diluted with EtOAc and passed through a short pad of silica using EtOAc. The filtrate was concentrated in *vacuo* and the crude product was monitored by ^{1}H NMR analysis with mesitylene (30 μL) as an internal standard. No desired product **7** was detected from ^{1}H NMR analysis.

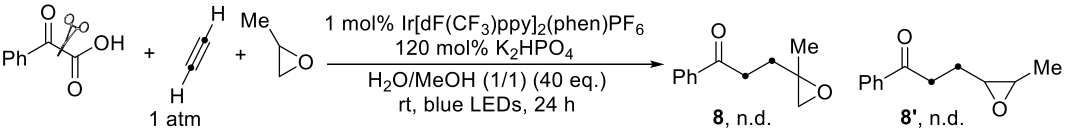

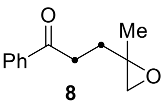
8

3-(2-methyloxiran-2-yl)-1-phenylpropan-1-one (8). Prepared according to the modified general procedure B employing Ir[dF(CF$_3$)ppy]$_2$(phen)PF$_6$ (3.3 mg, 0.003 mmol), benzoylformic acid (45.2 mg, 0.30 mmol), K_2HPO_4 (0.36 mmol, 62.9 mg), H_2O (6.0 mmol, 108 μL), MeOH (6.0 mmol, 243 μL) and cyclopentane (12 mL). After 24 h, the reaction was diluted with EtOAc and passed through a short pad of silica using EtOAc. The filtrate was concentrated in *vacuo* and the crude product was monitored by ^{1}H NMR analysis with mesitylene (30 μL) as an internal standard. No desired product **8** or **8'**was detected from ^{1}H NMR analysis.

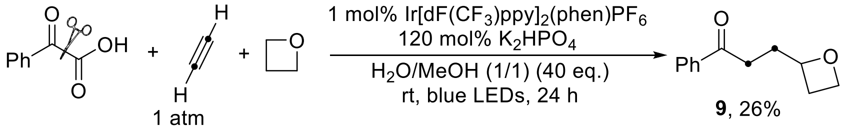

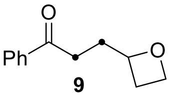
9

3-(oxetan-2-yl)-1-phenylpropan-1-one (9). Prepared according to the modified general procedure B employing Ir[dF(CF$_3$)ppy]$_2$(phen)PF$_6$ (3.4 mg, 0.003 mmol), benzoylformic acid (45.1 mg, 0.30 mmol), K_2HPO_4 (0.36 mmol, 62.5 mg), H_2O (3.0 mmol, 108 μL), MeOH (6.0 mmol, 243 μL) and oxetane (6 mL). After 24 h, the reaction was

diluted with EtOAc and passed through a short pad of silica using EtOAc. The filtrate was concentrated in *vacuo* before it was purified by flash chromatography (PE/EA = 7:1) on silica gel to afford **9** (15.0 mg, 0.079 mmol, 26% yield) as a yellow oil, and **2a** (6.9 mg, 0.0289 mmol, 19% yield) as a white solid. ^{1}H NMR (500 MHz, $CDCl_3$) δ 7.97 (d, *J* = 7.5 Hz, 2H), 7.58-7.53 (m, 1H), 7.46 (t, *J* = 7.7 Hz, 2H), 4.95-4.87 (m, 1H), 4.72-4.64 (m, 1H), 4.54 (dt, *J* = 9.5, 5.5 Hz, 1H), 3.17-3.07 (m, 1H), 3.05-2.95 (m, 1H), 2.77-2.66 (m, 1H), 2.44-2.33 (m, 1H), 2.26-2.09 (m, 2H); ^{13}C NMR (126 MHz, $CDCl_3$) δ 199.6, 136.8, 133.0, 128.6, 128.0, 81.8, 68.1, 33.2, 32.1, 27.4; HRMS (ESI-TOF) Calcd for $C_{12}H_{15}O_2$ $[M+H]^+$: 191.1072; Found 191.1066.

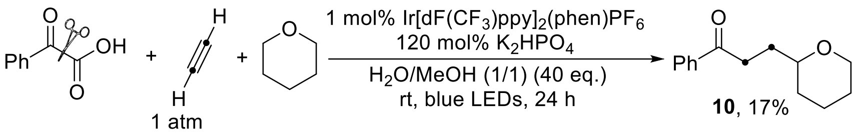

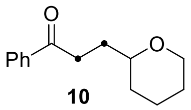
10

1-phenyl-3-(tetrahydro-2*H*-pyran-2-yl)propan-1-one (10). Prepared according to the modified general procedure B employing Ir[dF(CF_3)ppy]$_2$(phen)PF_6 (3.2 mg, 0.003 mmol), benzoylformic acid (45.3 mg, 0.30 mmol), K_2HPO_4 (0.36 mmol, 62.1 mg), H_2O (3.0 mmol, 108 μL), MeOH (6.0 mmol, 243 μL) and tetrahydropyran (6 mL). After 24 h, the reaction was diluted with EtOAc and passed through a short pad of silica using EtOAc. The filtrate was concentrated in *vacuo* before it was purified by flash chromatography (PE/EA = 10:1) on silica gel to afford **10** (11.1 mg, 0.051 mmol, 17% yield) as a yellow oil. For **10**: ^{1}H NMR (500 MHz, $CDCl_3$) δ 7.98 (d, *J* = 8.0 Hz, 2H), 7.55 (t, *J* = 7.5 Hz, 1H), 7.45 (t, *J* = 7.5 Hz, 2H), 4.01-3.92 (m, 1H), 3.43-3.35 (m, 1H), 3.35-3.27 (m, 1H), 3.19-3.02 (m, 2H), 1.99-1.89 (m, 1H), 1.88-1.76 (m, 2H), 1.68-1.59 (m, 1H), 1.59-1.43 (m, 3H), 1.37-1.27 (m, 1H); ^{13}C NMR (126 MHz, $CDCl_3$) δ 200.3, 137.1, 132.8, 128.5, 128.0, 76.9, 68.4, 34.5, 32.0, 30.7, 26.1, 23.4; HRMS (ESI-TOF) Calcd for $C_{14}H_{19}O_2$ $[M+H]^+$: 219.1385; Found 219.1385.

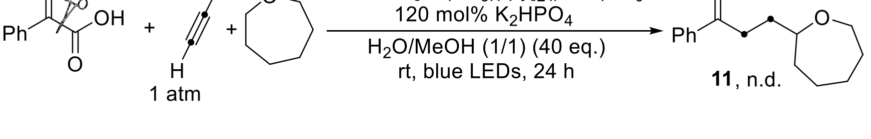

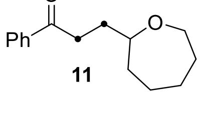
11

3-(oxepan-2-yl)-1-phenylpropan-1-one (11). Prepared according to the modified general procedure B employing Ir[dF(CF_3)ppy]$_2$(phen)PF_6 (3.3 mg,

0.003 mmol), benzoylformic acid (45.3 mg, 0.30 mmol), K_2HPO_4 (0.36 mmol, 62.7 mg), H_2O (6.0 mmol, 108 μL), MeOH (6.0 mmol, 243 μL) and oxocane (5 mL). After 24 h, the reaction was diluted with EtOAc and passed through a short pad of silica using EtOAc. The filtrate was concentrated in *vacuo* and the crude product was monitored by ^{1}H NMR analysis with mesitylene (30 μL) as an internal standard. No desired product **11** was detected from ^{1}H NMR analysis.

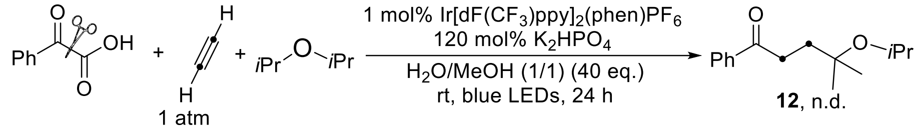

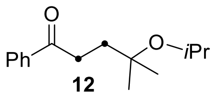

4-isopropoxy-4-methyl-1-phenylpentan-1-one (12). Prepared according to the modified general procedure B employing Ir[dF(CF3)ppy]2(phen)PF_6 (3.3 mg, 0.003 mmol), benzoylformic acid (45.5 mg, 0.30 mmol), K_2HPO_4 (0.36 mmol, 63.0 mg), H_2O (6.0 mmol, 108 μL), MeOH (6.0 mmol, 243 μL) and isopropyl ether (6 mL). After 24 h, the reaction was diluted with EtOAc and passed through a short pad of silica using EtOAc. The filtrate was concentrated in *vacuo* and the crude product was monitored by ^{1}H NMR analysis with mesitylene (30 μL) as an internal standard. No desired product **12** was detected from ^{1}H NMR analysis.

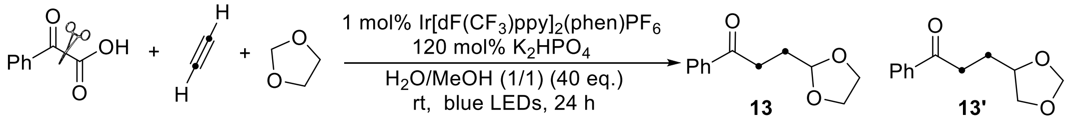

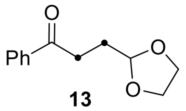

3-(1,3-dioxolan-2-yl)-1-phenylpropan-1-one (13).[20] Prepared according to the modified general procedure B employing Ir[dF(CF3)ppy]2(phen)PF_6 (5.2 mg, 0.005 mmol), benzoylformic acid (82.2 mg, 0.50 mmol), K_2HPO_4 (104.7 mg, 0. 6 mmol), H_2O (10.0 mmol, 180 μL), MeOH (10.0 mmol, 405 μL) and 1,3-dioxolane (20 mL). After 24 h, the reaction was diluted with EtOAc and passed through a short pad of silica using EtOAc. The filtrate was concentrated in *vacuo* before it was purified by flash chromatography (PE/EA = 10:1) on silica gel to afford a mixture of **13** and **13'** (39.0 mg, 0.1891 mmol, 38% yield, **13**/**13'** = 1/5) as a yellow oil. ^{1}H NMR (500 MHz, $CDCl_3$) δ 8.01-7.94 (m, 2H), 7.55 (t, *J* = 7.5 Hz, 1H), 7.49-7.41 (m, 2H), 5.03 (s, 0.14H), 5.01 (t, *J* = 4.0 Hz, 0.73H), 4.87 (s, 0.13H), 4.16-4.08 (m, 0.26H), 4.05-3.94 (m, 1.80H), 3.91-3.81 (m, 1.74H), 3.53 (dd, *J* = 7.5, 6.5 Hz, 0.17H), 3.20-3.07 (m, 2H), 2.15 (td, *J* = 7.5, 4.5 Hz, 1.61H), 2.10-2.05 (m, 0.19H), 2.00-1.91 (m, 0.19H); ^{13}C NMR (126 MHz, $CDCl_3$) δ 199.3, 136.9, 136.7, 133.1, 132.9, 128.6, 128.5, 128.0, 103.4, 94.8, 75.2, 69.5, 64.9, 34.5, 32.4, 27.9, 27.2.

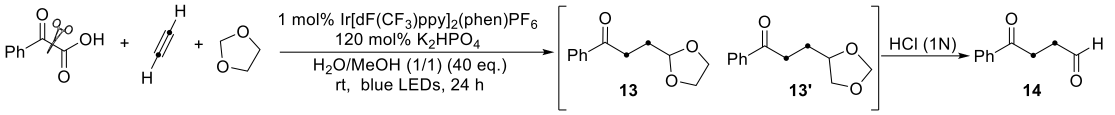

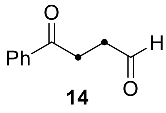

4-oxo-4-phenylbutanal (14).[21] Prepared according to the modified general procedure B employing Ir[dF(CF_3)ppy]$_2$(phen)PF_6 (3.2 mg, 0.003 mmol), benzoylformic acid (45.1 mg, 0.30 mmol), K_2HPO_4 (62.9 mg, 0.36 mmol), H_2O (6.0 mmol, 108 μL), MeOH (6.0 mmol, 243 μL) and 1,3-dioxolane (12 mL). After 24 h, the reaction was diluted with EtOAc and passed through a short pad of silica using EtOAc. The filtrate was concentrated in *vacuo* to afford a crude mixture. The mixture was dissolved in acetone (5 mL) and then HCl (1N, 6 mL) was added. The mixture was stirred at room temperature for 7 h. After extraction with EtOAc, the combined organic layers were dried over anhydrous Na_2SO_4. Then filtered and evaporation to afford a crude product, which was purified by flash chromatography (PE/EA = 10:1) on silica gel to afford **14** (14.3 mg, 0.0883 mmol, 29% yield) as a yellow oil. ^{1}H NMR (500 MHz, $CDCl_3$) δ 9.92 (s, 1H), 8.01 (d, J = 7.0 Hz, 2H), 7.63-7.56 (m, 1H), 7.49 (t, J = 7.5 Hz, 2H), 3.35 (t, J = 6.0 Hz, 2H), 2.95 (t, J = 6.0 Hz, 2H).

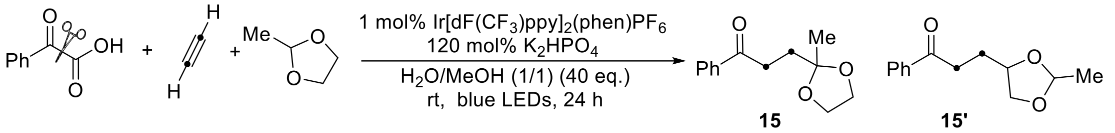

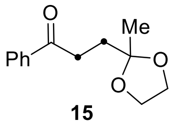

3-(2-methyl-1,3-dioxolan-2-yl)-1-phenylpropan-1-one (15). Prepared according to the modified general procedure B employing Ir[dF(CF_3)ppy]$_2$(phen)PF_6 (3.3 mg, 0.003 mmol), benzoylformic acid (45.2 mg, 0.30 mmol), K_2HPO_4 (63.1 mg, 0.36 mmol), H_2O (6.0 mmol, 108 μL), MeOH (6.0 mmol, 243 μL), 2-methyl-1,3-dioxolane (5 mL) and MeCN (1 mL). After 24 h, the reaction was diluted with EtOAc and passed through a short pad of silica using EtOAc. The filtrate was concentrated in *vacuo* before it was purified by flash chromatography (PE/EA = 10:1) on silica gel to afford a mixture of **15** and **15'** (24.1 mg, 0.1094 mmol, 36% yield) as a yellow oil. The ratio of **15** and **15'** (**15**/**15'** = 1/6) was determined through ^{1}H NMR analysis from a crude product. For **15**: ^{1}H NMR (500 MHz, $CDCl_3$) δ 7.97 (d, J = 7.5 Hz, 2H), 7.55 (t, J = 7.5 Hz, 1H), 7.46 (t, J = 7.5 Hz, 2H), 4.02-3.86 (m, 4H), 3.07 (t, J = 7.5 Hz, 2H), 2.15 (t, J = 7.5 Hz, 2H), 1.38 (s, 3H); ^{13}C NMR (126 MHz, $CDCl_3$) δ 199.8, 137.0, 132.9, 128.5, 128.0, 109.4, 64.7, 33.2, 33.1, 24.0; HRMS (ESI-TOF) Calcd for $C_{13}H_{16}O_3Na$

[M+Na]$^+$: 243.0997; Found 243.0987.

Application Potentials in the Syntheses of Bioactive Molecules and Transformations

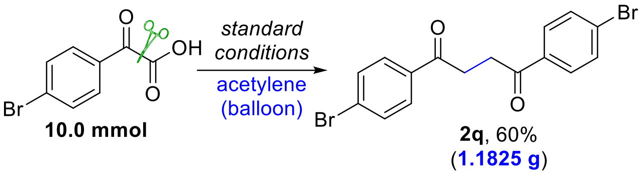

Prepared according to the modified procedure A: A 1L flask was evacuated and backfilled with acetylene gas for three times, then an acetylene gas balloon was attached. DCM (400 mL) was added and stirred for 1 hour. Ir[dF(CF_3)ppy]$_2$(phen)PF_6 (101.6 mg, 0.10 mmol), **1q** (2.2920 g, 10.0 mmol), K_2HPO_4 (2.0908 g, 12.00 mmol) and H_2O (200.0 mmol, 3.6 mL) were added sequentially. After being irradiated with blue LEDs for 55 h (12 W blue LEDs x2), the reaction was diluted with EtOAc and passed through a short pad of silica using EtOAc. The filtrate was concentrated in *vacuo* before it was purified by flash chromatography (PE/EA = 10:1) on silica gel to afford **2q** (1.1825 g, 2.986 mmol, 60% yield) as a white solid.

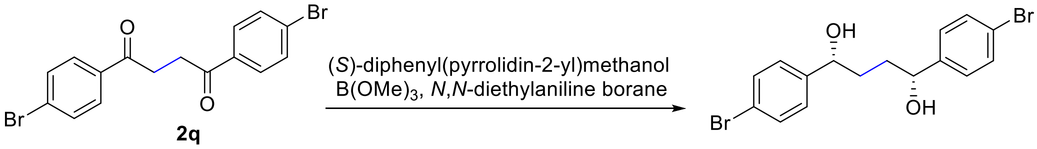

(1*R*,4*R*)-1,4-bis(4-bromophenyl)butane-1,4-diol.[22] Prepared according to a known procedure. To a stirred mixture of (*S*)-(-)-α,α-diphenyl-2-pyrrolidinemethanol (215.3 mg, 0.85 mmol) in anhydrous THF (8 mL) at room temperature was added trimethyl borate (114.3 mg, 1.10 mmol), and the resulting solution was stirred at room temperature for 2 hour. The solution was cooled to 0 °C, and *N*,*N*-diethylaniline borane (1.7940 g, 11.0 mmol) was added dropwise via syringe over 3-5 minutes (caution: vigorous H_2 evolution), while the internal temperature was maintained at <10 °C. When the H_2 evolution had ceased. The resulting borane mixture was transferred via syringe to a separate flask that contained **2q** (1.9690 g, 5.0 mmol) in anhydrous THF (12 mL) slowly. During the transfer period, the internal temperature of the reaction was maintained < 25 °C. After the addition was complete, the reaction was maintained at 15 °C for 30 min and stirred at room temperature for 3 hours. After reaction completion, the mixture was cooled to 0 °C and methanol (1.8 mL) was added dropwise. Then the reaction was brought to room temperature, and stirring was continued until complete dissolution of the solids had occurred. Ethyl acetate (28 mL) and 1 M HCl

(12.5 mL) were added, and the phases were separated. The organic phase was washed successively with 1M HCl (2 x12.5 mL), H_2O (65 mL), and 10% aqueous NaCl (65 mL). The solvent was removed and purified through flash column chromatography using DCM/MeOH (50/1) to afford a crude mixture, which was used directly in the next step.

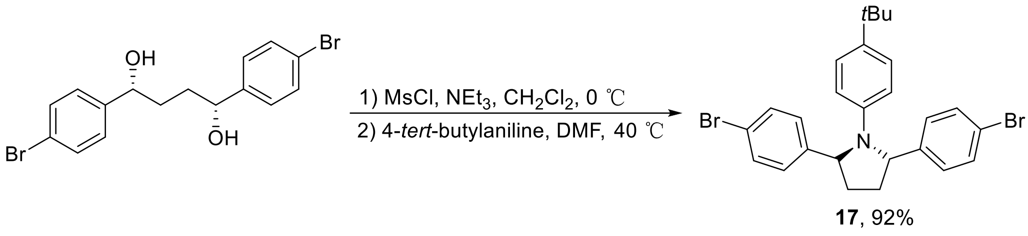

To a solution of the obtained crude alcohol (1.791 g, ca. 4.5 mmol) in anhydrous DCM (45 mL) at 0 °C was added TEA (3.75 mL, 27 mmol), and the resulting mixture was stirred at 0 °C for 10 min until a homogenous solution was obtained. To the cooled solution was added MsCl (1.7 mL, 22.5 mmol) dropwise, and the resulting mixture was stirred at 0 °C for 4 h until the reaction was completed as determined by TLC. Solvent was removed in *vacuo* to give a solid, which was dried in *vacuo*. The solid was dissolved in anhydrous DMF (30 mL), and 4-*tert*-butylaniline (7.2 mL, 45 mmol) was added. The resulting mixture was stirred at 40 °C for 5 h and then was partitioned between 1 N aq. HCl (80 mL) and EA (90 mL). The organic layers were washed with H_2O and dried over Na_2SO_4. The drying agent was filtered off and the solution was concentrated in *vacuo* to give a crude product that was purified by column chromatography on silica gel (PE/EA = 50/1) to give a colorless solid (2*S*,5*R*)-2,5-bis(4-bromophenyl)-1-(*p*-tolyl)pyrrolidine **17** (2.105 g, 4.12 mmol, 92% yield, *dr* 5/1, 91% ee). Chiral HPLC: (MD, 1 mL/min, hexane/*i*-PrOH =100/0, λ = 254 nm): t_R(major) = 14.1 min, t_R(minor) = 11.1 min. ^{1}H NMR (500 MHz, $CDCl_3$) δ 7.60-7.45 (m, 5H), 7.23-7.10 (m, 5H), 6.53 (d, *J* = 9.0 Hz, 0.33H), 6.34 (d, *J* = 9.0 Hz, 1.67H), 5.28-5.17 (m, 1.67H), 4.86-4.77 (m, 0.33H), 2.65-2.43 (m, 2H), 2.07-1.98 (m, 0.34H), 1.92-1.78 (m, 1.66H), 1.33 (s, 1.53H), 1.29 (s, 7.47H).

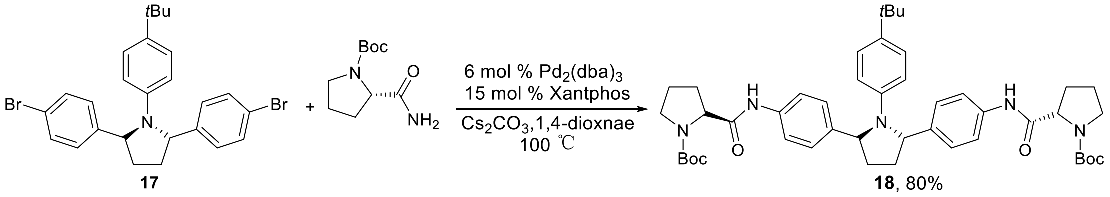

A mixture of (2*S*,5*R*)-2,5-bis(4-bromophenyl)-1-(*p*-tolyl)pyrrolidine **17** (613 mg, 1.2 mmol), *tert*-butyl (*S*)-2-carbamoylpyrrolidine-1-carboxylate (668 mg, 2.6 mmol), $Pd_2(dba)_3$ (66 mg, 0.072 mmol), Xantphos (104 mg, 0.18 mmol) and Cs_2CO_3 (1.173 g, 3.6 mmol) was added in 1,4-dioxane (12 mL) under N_2. The reaction container was sealed and the mixture was stirred while being heated at 100 ºC for 12 h. The mixture was cooled to rt and diluted into ethyl acetate, and the solution was washed with water and brine. The organic layers were dried over anhydrous Na_2SO_4, filtered and concentrated under *vacuum*. The crude product was purified by column chromatography on silica gel (PE/EA = 2/1) to give **18** (772.9 mg, 0.966 mmol, 80%, *dr* 5/1) as a white solid. ^{1}H NMR (500 MHz, $CDCl_3$) (major) δ 9.44 (s, 1H), 7.44 (d, *J* = 8.0 Hz, 4H), 7.13 (d, *J* = 6.5 Hz, 4H), 6.99 (d, *J* = 8.5 Hz, 2H), 6.25 (d, *J* = 9.0 Hz, 2H), 5.11 (d, *J* = 6.0 Hz, 2H), 4.56-4.31 (m, 2H), 3.56-3.23 (m, 4H), 2.60-2.40 (m, 3H), 2.02-1.80 (m, 7H), 1.79-1.65 (m, 2H), 1.48 (s, 18H), 1.17 (s, 9H).

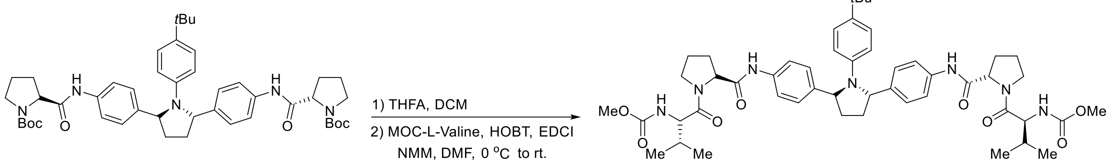

To an oven-dried flask cooled under N_2, **18** (312.1 mg, 0.4 mmol) and DCM/TFA (2 mL/3mL) were added. The reaction mixture was stirred at rt. When the starting material was consumed (monitored by TLC), the mixture was condensed to afford a crude product which was used directly without further purification.

The crude product obtained above was dissolved in DMF (10 mL) and cooled to 0 °C, followed by addition of MOC-L-Valine (210 mg, 1.2 mmol), 1-hydroxybenzotriazole hydrate (184 mg, 1.200 mmol), *N*-methylmorpholine (0.22 mL, 2.0 mmol), and *N*-(3-dimethylaminopropyl)-*N*'-ethylcarbodiimide hydrochloride (230 mg, 1.2 mmol). The resulting mixture was stirred at room temperature for 12 hours. The mixture was partitioned between ethyl acetate and water, and the organic layer was washed with saturated aqueous $NaHCO_3$, brine, and dried with Na_2SO_4. The drying agent was filtered off and the solution was concentrated in vacuo to give a crude product that was purified by column chromatography on silica gel (DCM/MeOH = 30/1) to give **19** (296.8 mg, 0.332 mmol, 83%) as white solid. ^{1}H NMR (500 MHz, $CDCl_3$) δ 9.28 (s, 2H), 7.38 (d, *J* = 8.0, 4H), 7.08 (d, *J* = 8.0, 4H), 6.97 (d, *J* = 8.0 Hz, 2H), 6.22 (d, *J* = 9.0 Hz, 2H), 5.89-5.75 (m, 2H), 5.08 (d, *J* = 6.0 Hz, 2H), 4.79-4.70 (m, 2H), 4.37-4.29 (m, 2H), 4.19-4.05 (m, 1H), 3.86-3.75 (m, 2H), 3.69-

3.56 (m, 8H), 2.53-2.36 (m, 3H), 2.25-2.10 (m, 2H), 2.09-1.97 (m, 5H), 1.95-1.86 (m, 2H), 1.72-1.63 (m, 2H), 1.15 (d, J = 1.0 Hz, 9H), 0.97 (d, J = 6.5 Hz, 6H), 0.94 (d, J = 6.5 Hz, 6H); ^{13}C NMR (125 MHz, $CDCl_3$) δ 172.9, 168.8, 157.2, 142.4, 140.0, 137.8, 136.6, 126.6, 125.4, 119.9, 113.3, 62.7, 60.7, 57.7, 52.3, 47.9, 33.6, 32.3, 31.5, 31.3, 26.8, 25.2, 19.3, 17.8.

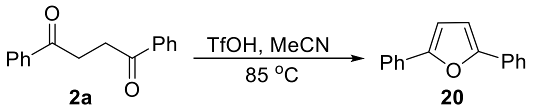

2a **20**

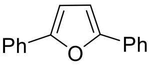

20

2,5-Diphenylfuran (20).[23] To an oven-dried flask cooled under N_2, **2a** (63.3 mg, 0.266 mmol), MeCN (2.5 mL) and TfOH (0.25 mmol, 22μL) were added. The reaction mixture was heated to 85 °C overnight. The reaction was quenched by Sat. $NaHCO_3$ and extracted by DCM. The combined organic layers were dried by anhydrous $MgSO_4$. After filtration, the filtrate was condensed and the residue was purified by column chromatography (PE/EA = 50:1) through silica gel to obtain **20** (56.5 mg, 0.26 mmol, 97% yield) as a white solid. ^{1}H NMR (500 MHz, $CDCl_3$) δ 7.73 (d, J = 7.5 Hz, 4H), 7.39 (t, J = 7.5 Hz, 4H), 7.25 (t, J = 7.5 Hz, 2H), 6.7 (s, 2H); ^{13}C NMR (126 MHz, $CDCl_3$) δ 153.3, 130.7, 128.7, 127.3, 123.7, 107.2.

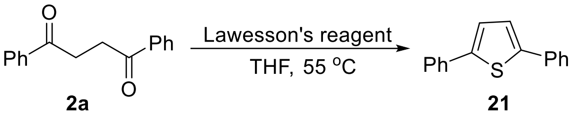

2a **21**

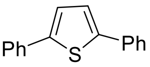

21

2,5-Diphenylthiophene (21).[23] To an oven-dried flask cooled under N_2, **2a** (47.6 mg, 0.20 mmol), THF (6 mL) and Lawesson's reagent (0.21 mmol, 83.6 mg) were added. The reaction mixture was heated to 55 °C for 5 h. The reaction mixture was condensed and the residue was purified by column chromatography (PE/EA = 30:1) through silica gel to obtain **21** (30.6 mg, 0.13 mmol, 65% yield) as a white solid. ^{1}H NMR (500 MHz, $CDCl_3$) δ 7.70-7.65 (m, 4H), 7.45-7.39 (m, 4H), 7.35-7.29 (m, 4H); ^{13}C NMR (126 MHz, $CDCl_3$) δ 143.6, 134.3, 128.9, 127.5, 125.6, 124.0.

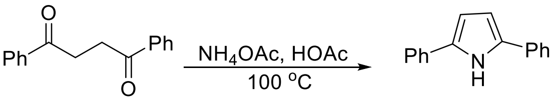

2a **22**

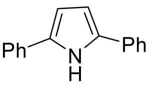

22

2,5-Diphenyl-1*H*-pyrrole (22).[24] To an oven-dried flask cooled under N_2, **2a** (73.1 mg, 0.31 mmol), NH_4OAc (136.6 mg, 1.77 mmol) and HOAc (2 mL) were added. The reaction mixture was refluxed at 100 °C overnight. The reaction mixture was cooled to rt and poured into ice-water. The solid was collected and washed by H_2O. The wet solid

was dried to afford **22** (63.4 mg, 0.29 mmol, 94% yield) as a gray solid. ^{1}H NMR (500 MHz, $CDCl_3$) δ 8.51 (s, 1H), 7.49 (d, J = 7.5 Hz, 4H), 7.35 (t, J = 7.5 Hz, 4H), 7.19 (t, J = 7.5 Hz, 2H), 6.56 (d, J = 2.5 Hz, 2H); ^{13}C NMR (126 MHz, $CDCl_3$) δ 133.1, 132.5, 128.9, 126.4, 123.8, 107.9.

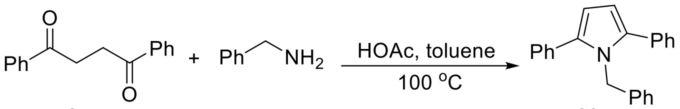

2a **23**

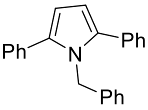

23

1-Benzyl-2,5-diphenyl-1*H*-pyrrole (23).[25] To an oven-dried flask cooled under N_2, **2a** (65.5 mg, 0.27 mmol), benzylamine, (32.1 mg, 0.3 mmol), HOAc (3 drops) and toluene (2 mL) were added. The reaction mixture was refluxed at 100 °C overnight. The reaction mixture was cooled to rt, condensed and purified by column chromatography (PE/EA = 10:1) through silica gel to obtain **23** (30.5 mg, 0.099 mmol, 36% yield) as a white solid. ^{1}H NMR (500 MHz, $CDCl_3$) δ 7.39-7.32 (m, 4H), 7.32-7.27 (m, 4H), 7.26-7.21 (m, 2H), 7.15-7.05 (m, 3H), 6.65 (d, J = 6.5 Hz, 2H), 6.36 (s, 2H), 5.23 (s, 2H); ^{13}C NMR (126 MHz, $CDCl_3$) δ 139.2, 136.8, 133.7, 129.0, 128.3, 128.2, 127.0, 126.7, 125.9, 109.7, 48.7.

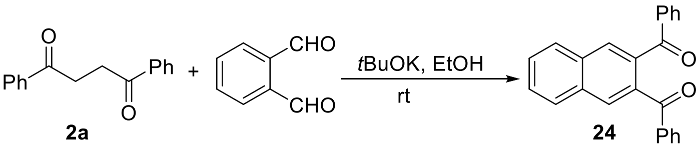

2a **24**

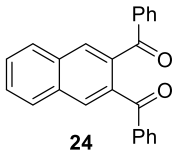

24

Naphthalene-2,3-diylbis(phenylmethanone) (24).[26] To a flask, **2a** (73.5 mg, 0.31 mmol), *o*-Phthalaldehyde (41.5 mg, 0.31 mmol) and EtOH (3 mL) were added. *t*BuOK (88.6 mg, 0.79 mmol) was added slowly. Then the reaction was stirred at rt overnight. The reaction mixture was quenched by H_2O and extracted by EtOAc for 3 times. The combined organic layers were dried over anhydrous Na_2SO_4. After filtered, the filtration was condensed and purified by column chromatography (PE/EA = 10:1) through silica gel to obtain **24** (102.1 mg, 0.30 mmol, 98% yield) as a yellow oil. ^{1}H NMR (500 MHz, $CDCl_3$) δ 8.08 (s, 2H), 7.94-7.88 (m, 2H), 7.85-7.77 (m, 4H), 7.66-7.60 (m, 2H), 7.55-7.48 (m, 2H), 7.44-7.34 (m, 4H); ^{13}C NMR (126 MHz, $CDCl_3$) δ 196.2, 137.3, 136.9, 132.9, 132.8, 130.7, 129.8, 128.7, 128.6, 128.3.

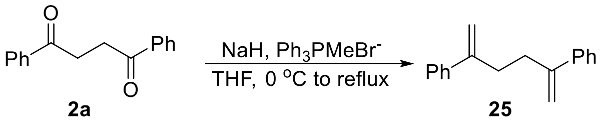

2a **25**

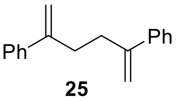

25

Hexa-1,5-diene-2,5-diyldibenzene (25).[27] To an oven-dried flask cooled under N_2, Methyltriphenylphosphonium bromide (357.2 mg, 1.0 mmol), NaH (40 mg, 1.0 mmol, 60% in mineral oil) and THF (2.5 mL) were added. The reaction mixture was refluxed for 30 min. The reaction mixture was cooled to rt, then **2a** (75.0 mg, 0.31 mmol, dissolved in 2.5 mL of THF) was added dropwise at 0 °C. After addition, the reaction was refluxed overnight. The reaction was quenched by Sat. NH_4Cl, extracted by EtOAc and dried over anhydrous Na_2SO_4. After filtration, the filtrate was condensed and purified by column chromatography (PE/EA = 10:1) through silica gel to obtain **25** (60.0 mg, 0.26 mmol, 81% yield) as a courless oil. ^{1}H NMR (500 MHz, $CDCl_3$) δ 7.41-7.36 (m, 4H), 7.34-7.29 (m, 4H), 7.29-7.24 (m, 2H), 5.27 (d, *J* = 5.0 Hz, 2H), 5.04 (s, 2H), 2.65 (s, 4H); ^{13}C NMR (126 MHz, $CDCl_3$) δ 148.1, 141.2, 128.3, 127.4, 126.2, 112.5, 34.3.

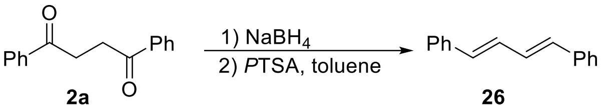

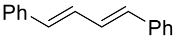

26

(*1E,3E*)-1,4-diphenylbuta-1,3-diene (26).[28] To an oven-dried flask cooled under N_2, **2a** (60.0 mg, 0.25 mmol) and THF/MeOH (3 mL/ 1 mL) were added. $NaBH_4$ (37.8 mg, 1.0 mmol) was added slowly at 0 °C. The reaction mixture was stirred at 0 °C to rt. The reaction mixture was monitored by TLC until **2a** was consumed. The reaction was quenched by HCl (1N) at 0 °C, extracted by EA and dried over anhydrous Na_2SO_4. After filtration, the filtrate was condensed to afford the corresponding crude alcohol (86.6 mg) and used directly without further purification. The corresponding crude alcohol (60.5 mg) was dissolved in toluene (5 mL) and *p*-TSA (9.6 mg, 0.050 mmol) was added. The reaction was refluxed for 6 h. After cooled to rt, the mixture was condensed and purified by column chromatography (PE/DCM = 10:1) through silica gel to obtain **26** (18.0 mg, 0.087 mmol, 35% yield) as a courless oil. ^{1}H NMR (500 MHz, $CDCl_3$) δ 7.44 (d, *J* = 7.5 Hz, 4H), 7.33 (dd, *J* = 7.5, 7.5 Hz, 4H), 7.23 (dd, *J* = 7.5, 7.5 Hz, 2H), 7.00-6.92 (m, 2H), 6.72-6.63 (m, 2H).

II. Supplementary Discussion

Supplementary Mechanistic studies

a) Radical trapping experiment and light-on-off experiment

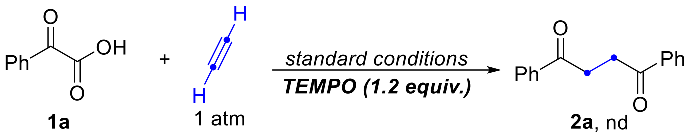

To a 25 mL flask, α-Oxocarboxylic Acid **1a** (0.3 mmol), Ir[dF(CF_3)ppy]$_2$(phen)PF_6 (0.003 mmol), K_2HPO_4 (0.36 mmol, 1.2 equiv.), H_2O (6.0 mmol, 20 equiv.) and DCM (12 mL) were added sequentially under N_2 atmosphere. The mixture was degassed through three freeze−pump−thaw cycles under acetylene gas and then an acetylene gas balloon was attached through a long syringe needle. The reaction mixture was irradiated by 12 W blue LEDs at a distance of 5 cm for 24 h with a cooling fan. The reaction mixture was then diluted with EtOAc and filtered through a short pad of silica using EtOAc. The filtrate was concentrated in *vacuo*. The crude mixture was monitored by ^{1}H NMR (0% NMR yield) with mesitylene as an internal standard.

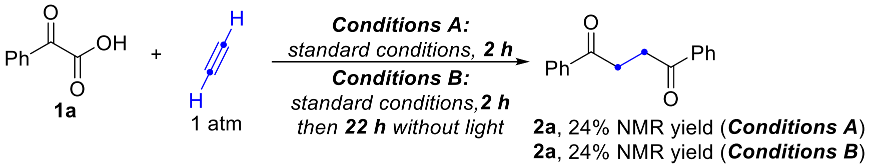

To a 25 mL flask, α-Oxocarboxylic Acid **1a** (0.3 mmol), Ir[dF(CF_3)ppy]$_2$(phen)PF_6 (0.003 mmol), K_2HPO_4 (0.36 mmol, 1.2 equiv.), H_2O (6.0 mmol, 20 equiv.) and DCM (12 mL) were added sequentially under N_2 atmosphere. The mixture was degassed through three freeze−pump−thaw cycles under acetylene gas and then an acetylene gas balloon was attached through a long syringe needle. The reaction mixture was irradiated by 12 W blue LEDs at a distance of 5 cm for 2 h with a cooling fan. The reaction mixture was then diluted with EtOAc and filtered through a short pad of silica using EtOAc. The filtrate was concentrated in *vacuo*. The crude mixture was monitored by ^{1}H NMR (24% NMR yield) with mesitylene as an internal standard.

To a another 25 mL flask, α-Oxocarboxylic Acid **1a** (0.3 mmol), Ir[dF(CF_3)ppy]$_2$(phen)PF_6 (0.003 mmol), K_2HPO_4 (0.36 mmol, 1.2 equiv.), H_2O (6.0 mmol, 20 equiv.) and DCM (12 mL) were added sequentially under N_2 atmosphere. The mixture was degassed through three freeze-pump-thaw cycles under acetylene gas and then an acetylene gas balloon was attached through a long syringe needle. The reaction mixture was irradiated by 12 W blue LEDs at a distance of 5 cm for 2 h with a cooling fan. The reaction was then performed for another 22 h without light. The reaction mixture was then diluted with EtOAc and filtered through a short pad of silica using EtOAc. The filtrate was

concentrated in *vacuo*. The crude mixture was monitored by ^{1}H NMR (24% NMR yield) with mesitylene as an internal standard.

b) Exploration of intermediate involved

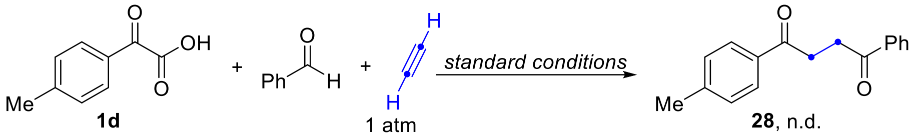

To a 25 mL flask, α-Oxocarboxylic Acid **1d** (0.3 mmol), Ir[dF(CF_3)ppy]$_2$(phen)PF_6 (0.003 mmol), K_2HPO_4 (0.36 mmol, 1.2 equiv.), H_2O (6.0 mmol, 20 equiv.), benzaldehyde (0.36 mmol) and DCM (12 mL) were added sequentially under N_2 atmosphere. The mixture was degassed through three freeze-pump-thaw cycles under acetylene gas and then an acetylene gas balloon was attached through a long syringe needle. The reaction mixture was irradiated by 12 W blue LEDs at a distance of 5 cm for 24 h with a cooling fan. The reaction mixture was then diluted with EtOAc and filtered through a short pad of silica using EtOAc. The crude mixture was monitored by ^{1}H NMR (0 % nmr yield of desired compound **28**).

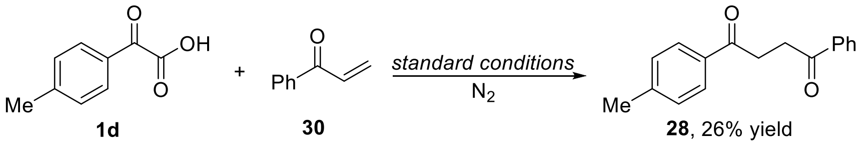

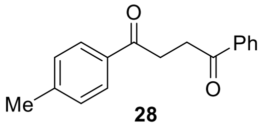

1-Phenyl-4-(*p*-tolyl)butane-1,4-dione (28).[29] To a 25 mL flask, α-Oxocarboxylic Acid **1d** (50.9 mg, 0.31 mmol), Ir[dF(CF_3)ppy]$_2$(phen)PF_6 (3.5 mg, 0.003 mmol), K_2HPO_4 (62.8 mg, 0.36 mmol), H_2O (6.0 mmol, 20 equiv.), alkene (44.5 mg, 0.34 mmol) and DCM (12 mL) were added sequentially under N_2 atmosphere. The mixture was degassed through three freeze-pump-thaw cycles under N_2 atmosphere. The reaction mixture was irradiated by 12 W blue LEDs at a distance of 5 cm for 24 h with a cooling fan. The reaction mixture was then diluted with EtOAc and filtered through a short pad of silica using EtOAc. The filtrate was concentrated in *vacuo*. The filtrate was concentrated in *vacuo* before it was purified by flash chromatography (PE/EA = 10:1) on silica gel to afford **28** (20.0 mg, 0.079 mmol, 26% yield) as a white solid. ^{1}H NMR (500 MHz, $CDCl_3$) δ 8.06-8.01 (m, 2H), 7.94 (d, *J* = 5.0 Hz, 2H), 7.60-7.54 (m, 1H), 7.50-7.45 (m, 2H), 7.27 (d, *J* = 8.0 Hz, 2H), 3.46-3.43 (m, 4H), 2.42 (s, 3H); ^{13}C NMR (126 MHz, $CDCl_3$) δ 198.7, 198.2, 143.9, 136.9, 134.4, 133.1, 129.3, 128.6, 128.2, 128.1, 32.6, 32.5, 21.6.

c) Exploration of hydrogen source

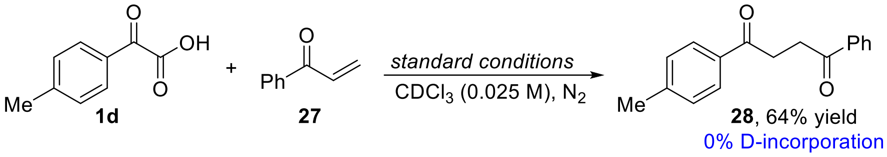

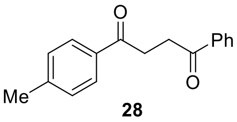

1-Phenyl-4-(p-tolyl)butane-1,4-dione (28). [30] To a 25 mL flask, α-Oxocarboxylic Acid **1d** (50.2 mg, 0.30 mmol), Ir[dF(CF_3)ppy]$_2$(phen)PF_6 (3.3 mg, 0.003 mmol), K_2HPO_4 (62.9 mg, 0.36 mmol), H_2O (6.0 mmol, 20 equiv.), alkene (46.4 mg, 0.35 mmol) and $CDCl_3$ (12 mL) were added sequentially under N_2 atmosphere. The mixture was degassed through three freeze-pump-thaw cycles under N_2 atmosphere. The reaction mixture was irradiated by 12 W blue LEDs at a distance of 5 cm for 24 h with a cooling fan. The reaction mixture was then diluted with EtOAc and filtered through a short pad of silica using EtOAc. The filtrate was concentrated in *vacuo* before it was purified by flash chromatography (PE/EA = 10:1) on silica gel to afford **28** (49.2 mg, 0.195 mmol, 64% yield) as a white solid. 0% D-incorporation in α-position of carbonyl group according to ^1H NMR analysis (**See Supplementary Fig. 1** for details).

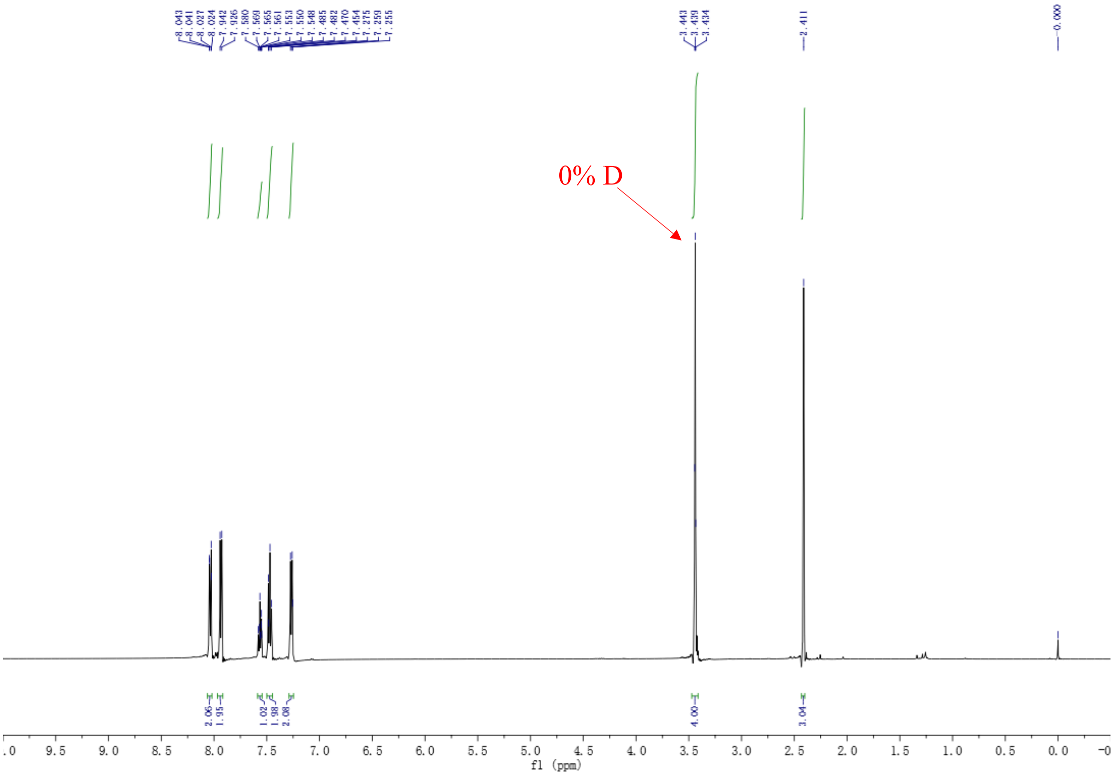

Supplementary Fig. 1 ^1H NMR spectrum of compound **28**.

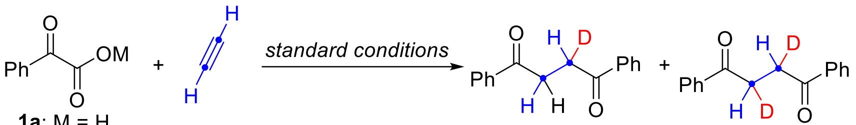

1a: M = H
1aa: M= K

1 atm

30a **31a**

entry	substrate	variation from the standard conditions	**30a**	**31a**
1[a]	**1a**	CD_2Cl_2, instead of DCM	51% (>90% D)	/
2[a]	**1aa**	D_2O, CD_2Cl_2, instead of H_2O and DCM	/	70% (>90% D)
3[a]	**1aa**	D_2O, instead of H_2O	75% (>90% D)	/
4[b]	**2a**	D_2O, instead of H_2O	/	/

[a] Yield was determined by ^1H NMR with mesithlene as internal standard, D% was determined through ^1H NMR; [b] 96% recovery of **2a**.

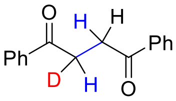

30a

1,4-Diphenylbutane-1,4-dione-2-d (30a). To a 25 mL flask, α-Oxocarboxylic Acid **1a** (0.3 mmol), Ir[dF(CF_3)ppy]$_2$(phen)PF_6 (0.003 mmol), K_2HPO_4 (0.36 mmol, 1.2 equiv.), H_2O (6.0 mmol, 20 equiv.) and CD_2Cl_2 (12 mL) were added sequentially under N_2 atmosphere. The mixture was degassed through three freeze-pump-thaw cycles under acetylene gas and then an acetylene gas balloon was attached through a long syringe needle. The reaction mixture was irradiated by 12 W blue LEDs at a distance of 5 cm for 24 h with a cooling fan. The reaction mixture was then diluted with EtOAc and filtered through a short pad of silica using EtOAc. The filtrate was concentrated in *vacuo* before it was purified by flash chromatography (PE/EA = 10:1) on silica gel to afford **30a** (18.4 mg, 0.077 mmol, 51% yield, > 90% D) as a white solid (**See Supplementary Fig. 2** for details). ^1H NMR (500 MHz, $CDCl_3$) δ 8.08-8.01 (m, 4H), 7.62-7.54 (m, 2H), 7.53-7.44 (m, 4H), 3.49- 3.41 (m, 3.05H); HRMS (ESI-TOF) Calcd for $C_{16}H_{13}DO_2Na$ $[M+Na]^+$: 262.0954; found 262.0949.

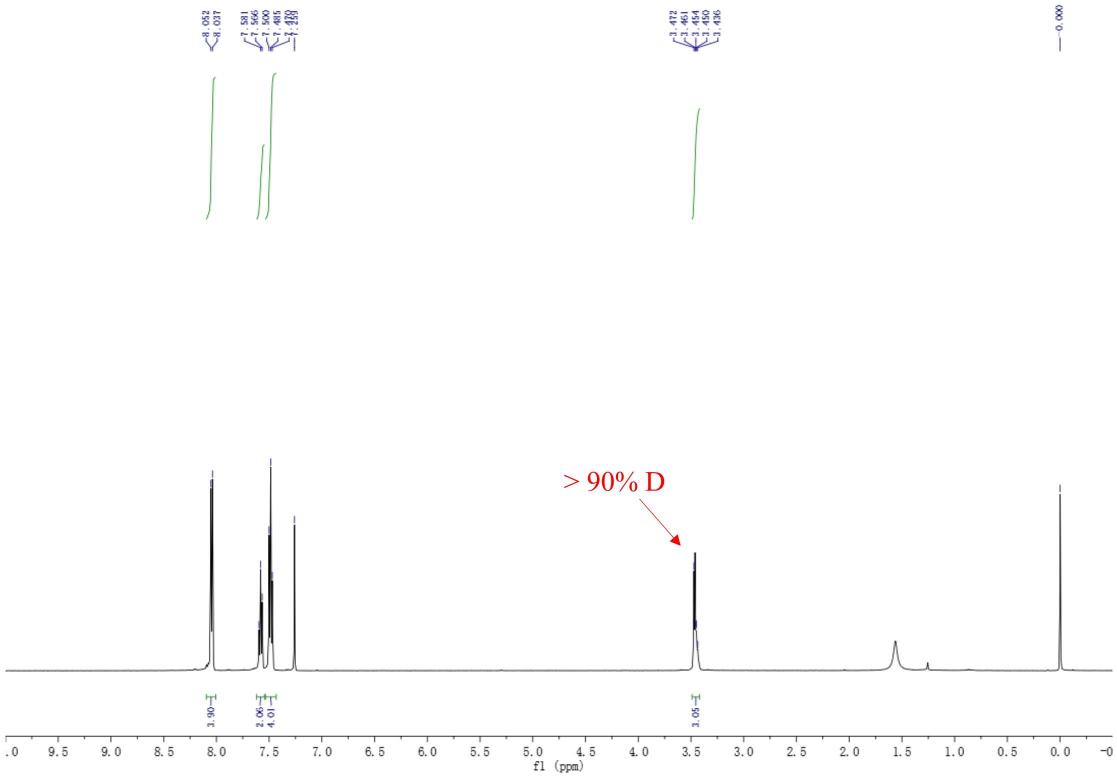

Supplementary Fig. 2 ^{1}H NMR spectrum of compound **30a**.

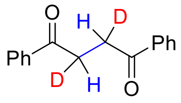

31a

1,4-Diphenylbutane-1,4-dione-2,3-d2 (31a). To a 25 mL flask, potassium 2-oxo-2-phenylacetate **1aa** (55.8 mg, 0.30 mmol), Ir[dF(CF_3)ppy]$_2$(phen)PF_6 (3.1 mg, 0.003 mmol), D_2O (6.0 mmol, 108 μL) and CD_2Cl_2 (12 mL) were added sequentially under N_2 atmosphere. The mixture was degassed through three freeze-pump-thaw cycles under acetylene gas and then an acetylene gas balloon was attached through a long syringe needle. The reaction mixture was irradiated by 12 W blue LEDs at a distance of 5 cm for 24 h with a cooling fan. The reaction mixture was then diluted with EtOAc and filtered through a short pad of silica using EtOAc. The filtrate was concentrated in *vacuo* before it was purified by flash chromatography (PE/EA = 10:1) on silica gel to afford **31a** (18.4 mg, 0.077 mmol, 51% yield) as a white solid (**See Supplementary Fig. 3** for details). ^{1}H NMR (500 MHz, $CDCl_3$) δ 8.05 (d, *J* = 8.0 Hz, 4H), 7.58 (t, *J* = 7.5 Hz, 2H), 7.49 (t, *J* = 7.5 Hz, 4H), 3.49-3.45 (m, 1.01H), 3.45-3.42 (m, 1.09 H); HRMS (ESI-TOF) Calcd for $C_{16}H_{13}D_2O_2$ $[M+H]^+$: 241.1198; found 241.1207.

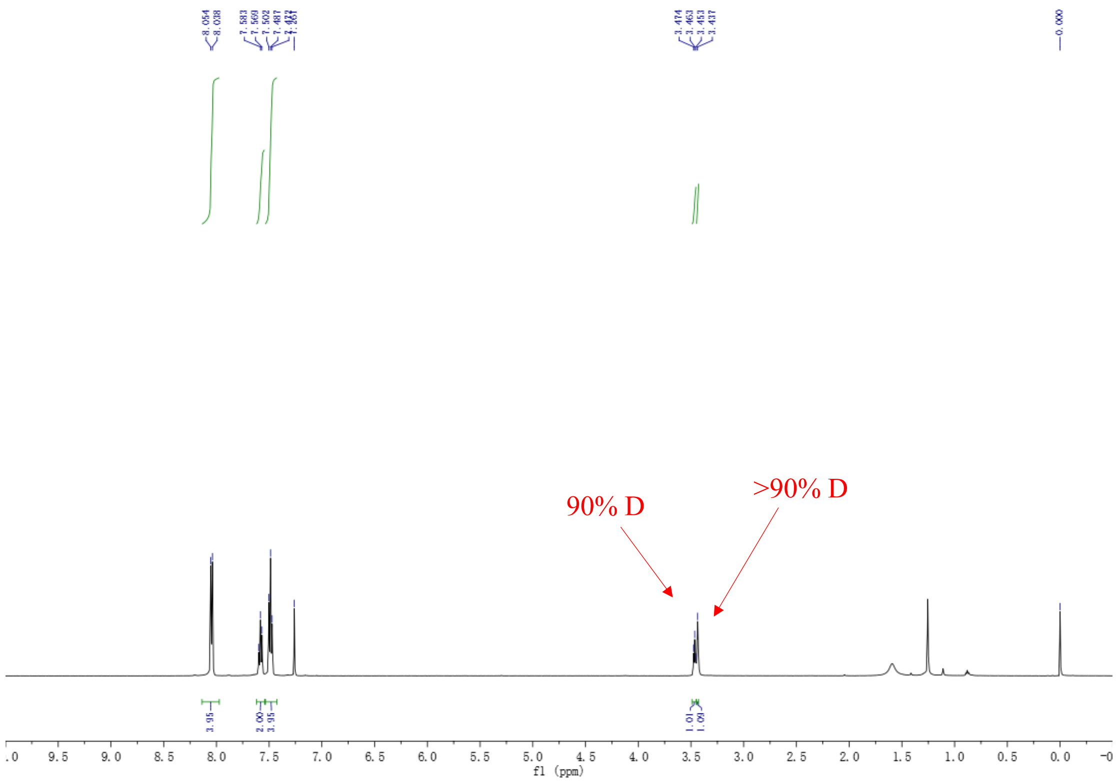

Supplementary Fig. 3 ^{1}H NMR spectrum of compound **31a**.

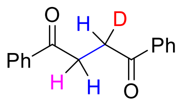

30a

1,4-Diphenylbutane-1,4-dione-2-d (30a). Prepared according to the modified general procedure A employing Ir[dF(CF_3)ppy]$_2$(phen)PF_6 (3.0 mg, 0.003 mmol), **1aa** (56.0 mg, 0.30 mmol), D_2O (6.0 mmol, 108 μL) and DCM (12 mL). After 24 h, the reaction was diluted with EtOAc and passed through a short pad of silica using EtOAc. The filtrate was concentrated in *vacuo* before it was purified by flash chromatography (PE/EA = 20:1) on silica gel to afford **30a** (26.9 mg, 0.113 mmol, 75% yield) as a white solid (**See Supplementary Fig. 4** for details). ^{1}H NMR (500 MHz, $CDCl_3$) δ 8.08-8.01 (m, 2H), 7.62-7.55 (m, 2H), 7.52-7.45 (m, 4H), 3.49- 3.41 (m, 3.01H); HRMS (ESI-TOF) Calcd for $C_{16}H_{13}DO_2Na$ $[M+Na]^+$: 262.0954; found 262.0949.

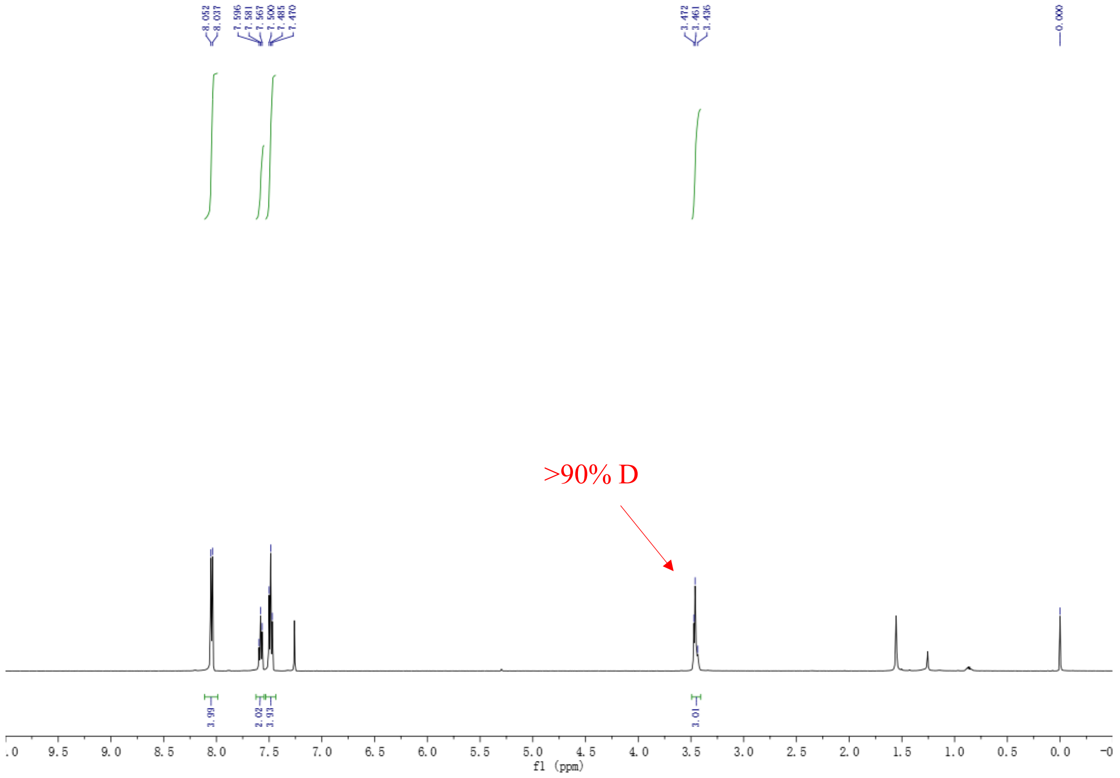

Supplementary Fig. 4 ^{1}H NMR spectrum of compound **30a**.

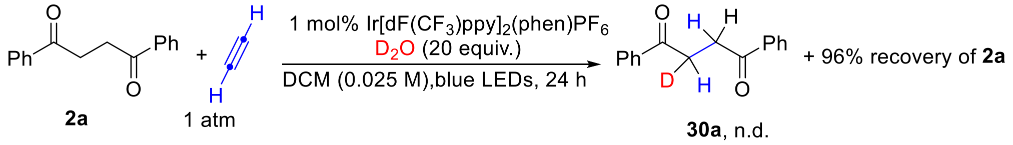

To a 25 mL flask, **2a** (35.7 mg, 0.15 mmol), Ir[dF(CF_3)ppy]$_2$(phen)PF_6 (2.0 mg, 0.0015 mmol), D_2O (6.0 mmol, 54 μL) and DCM (6 mL) were added sequentially under N_2 atmosphere. The mixture was degassed through three freeze-pump-thaw cycles under acetylene gas and then an acetylene gas balloon was attached through a long syringe needle. The reaction mixture was irradiated by 12 W blue LEDs at a distance of 5 cm for 24 h with a cooling fan. The reaction mixture was then diluted with EtOAc and filtered through a short pad of silica using EtOAc. The filtrate was concentrated in *vacuo*. The crude mixture was monitored by ^{1}H NMR (no D was incorporated) (**See Supplementary Fig. 5** for details).

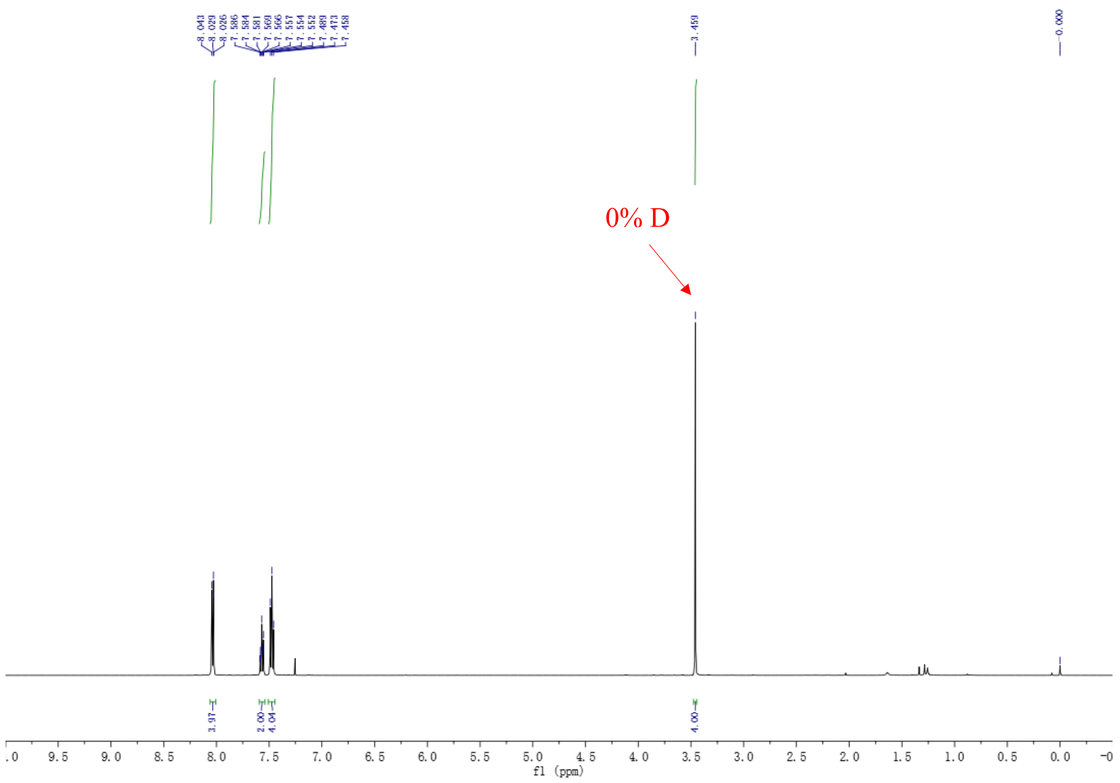

Supplementary Fig. 5 ^{1}H NMR spectrum of corresponding crude mixture.

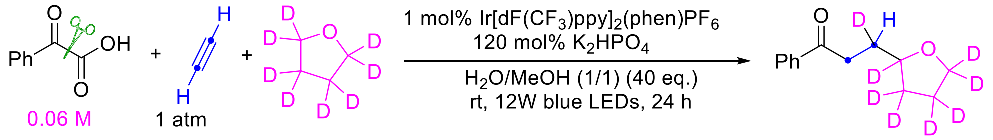

3ae-d8

To a 25 mL flask, α-Oxocarboxylic Acid **1a** (45.0 mg, 0.3 mmol), Ir[dF(CF_3)ppy]$_2$(phen)PF_6 (3.1 mg, 0.003 mmol), K_2HPO_4 (62.6 mg, 0.36 mmol, 1.2 equiv.), H_2O (6.0 mmol, 20 equiv.), MeOH (6.0 mmol, 20 equiv.) and tetrahydrofuran-d8 (5 mL) were added sequentially under N_2 atmosphere. The mixture was degassed through three freeze-pump-thaw cycles under acetylene gas and then an acetylene gas balloon was attached through a long syringe needle. The reaction mixture was irradiated by 12 W blue LEDs at a distance of 5 cm for 24 h with a cooling fan. The reaction mixture was then diluted with EtOAc and filtered through a short pad of silica using EtOAc. The filtrate was concentrated in *vacuo* before it was purified by flash chromatography (PE/EA = 10:1) on silica gel to afford **3ae-d8** (9.4 mg, 0.044 mmol, 15% yield, > 90% D) as a colorless oil (**See Supplementary Fig. 6** for details). ^{1}H NMR (500 MHz, $CDCl_3$) δ 7.98 (d, J = 7.0 Hz, 2H), 7.55 (t, J = 7.0 Hz, 1H), 7.46 (t, J = 7.5 Hz, 2H), 3.20-3.12 (m, 1H), 3.09-3.01 (m, 1H), 2.01-1.94 (m, 0.54H), 1.90-1.82 (m, 0.55H).

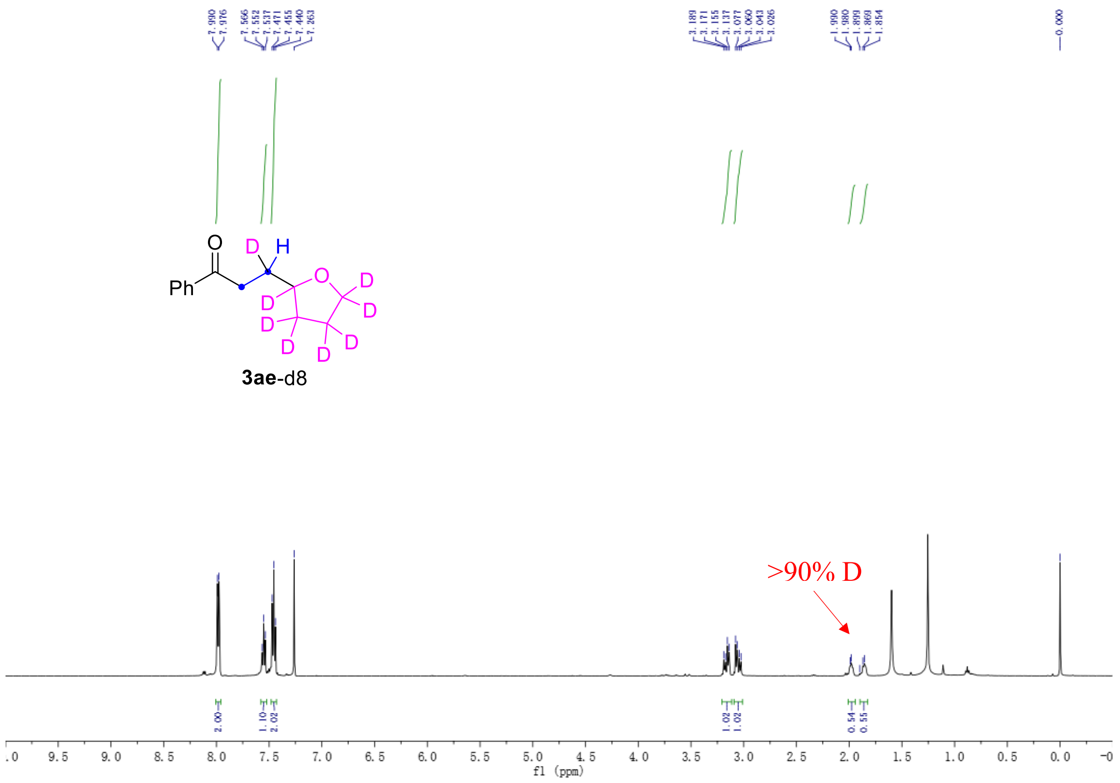

Supplementary Fig. 6 ^{1}H NMR spectrum of compound **3ae-d8**

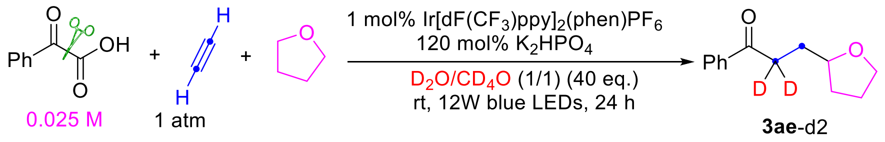

To a 25 mL flask, α-Oxocarboxylic Acid **1a** (45.1 mg, 0.3 mmol), Ir[dF(CF_3)ppy]$_2$(phen)PF_6 (3.1 mg, 0.003 mmol), K_2HPO_4 (62.9 mg, 0.36 mmol, 1.2 equiv.), D_2O (6.0 mmol, 20 equiv.), CD_3OD (6.0 mmol, 20 equiv.) and tetrahydrofuran (12 mL) were added sequentially under N_2 atmosphere. The mixture was degassed through three freeze-pump-thaw cycles under acetylene gas and then an acetylene gas balloon was attached through a long syringe needle. The reaction mixture was irradiated by 12 W blue LEDs at a distance of 5 cm for 24 h with a cooling fan. The reaction mixture was then diluted with EtOAc and filtered through a short pad of silica using EtOAc. The filtrate was concentrated in *vacuo* before it was purified by flash chromatography (PE/EA = 10:1) on silica gel to afford **3ae-d2** (43.1 mg, 0.209 mmol, 69% yield) as a colorless oil (**See Supplementary Fig. S7** for details). ^{1}H NMR (500 MHz, $CDCl_3$) δ 7.98 (d, J = 7.5 Hz, 2H), 7.55 (t, J = 7.5 Hz, 1H), 7.45 (t, J = 7.5 Hz, 2H), 3.95-3.82 (m, 2H), 3.77-3.68 (m, 1H), 3.22-3.12 (m, 0.72H), 3.10-3.01 (m, 0.70H), 2.09-1.97 (m, 2H), 1.96-1.82 (m, 3H), 1.59-1.47 (m, 1H). The relatively lower D-

incorporation may be attributed to the hydrogen source in acid **1a** and K_2HPO_4.

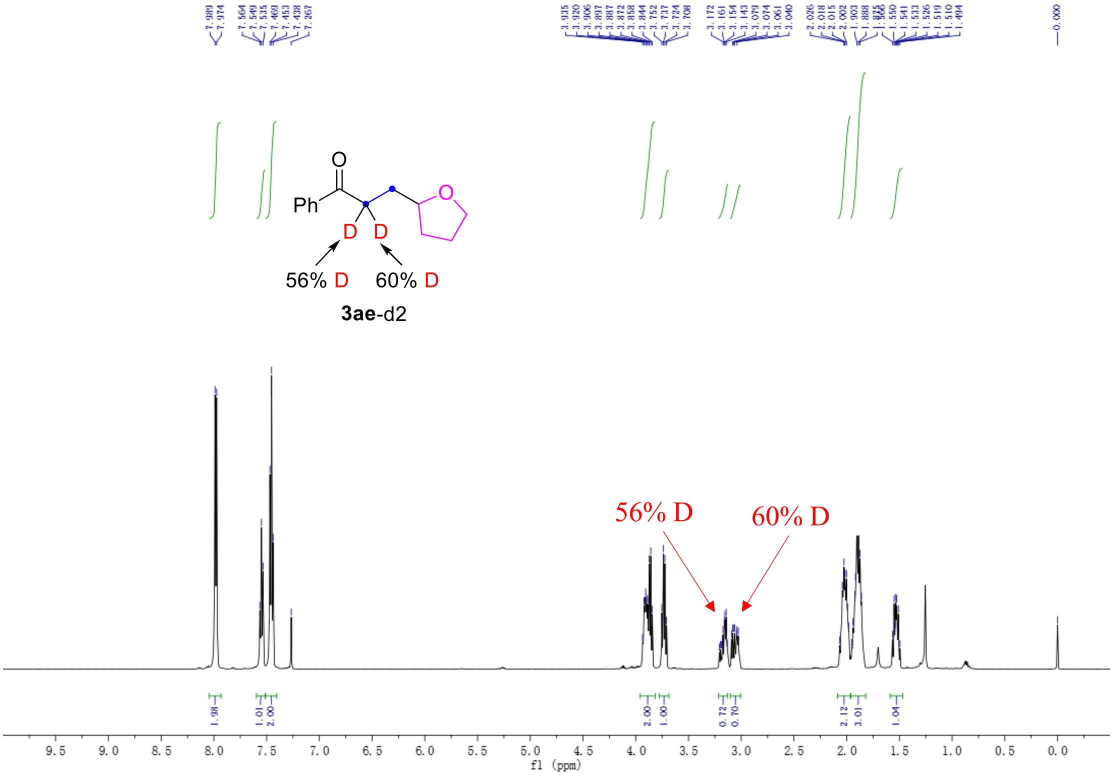

Supplementary Fig. 7 ^{1}H NMR spectrum of compound **3ae-d2**

III. Supplementary NMR Spectra and HPLC Spectra

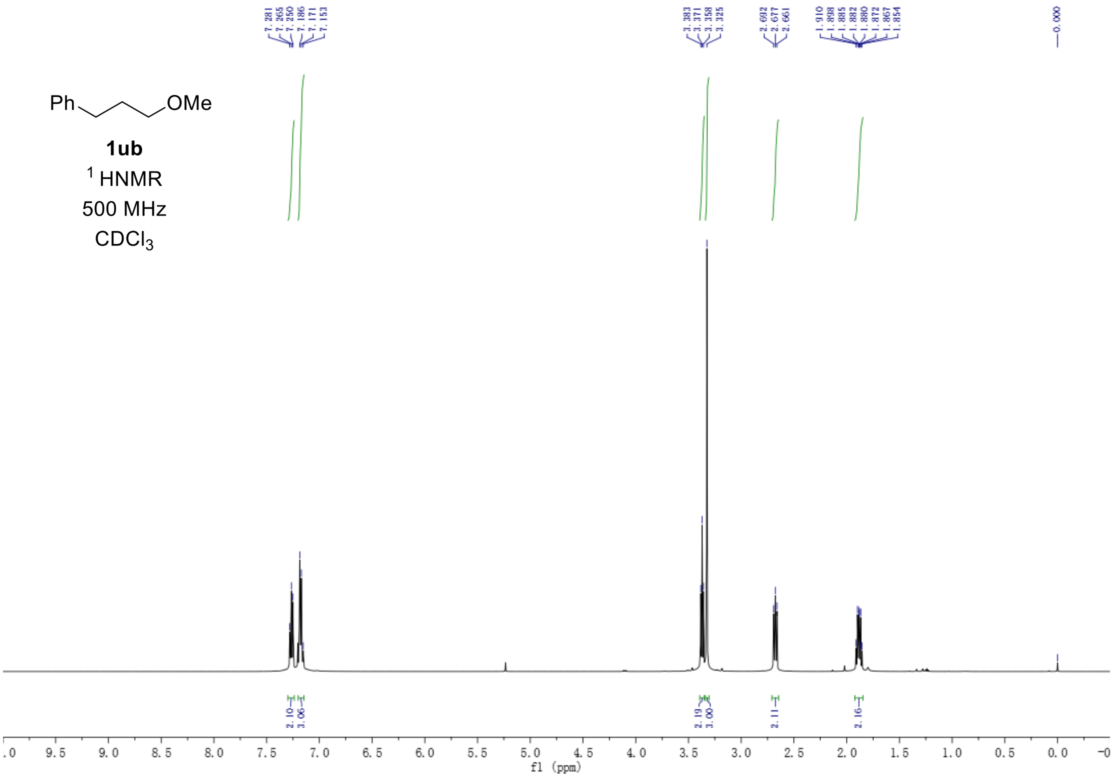

Supplementary Fig. 8 ^{1}H NMR spectrum of compound **1ub**

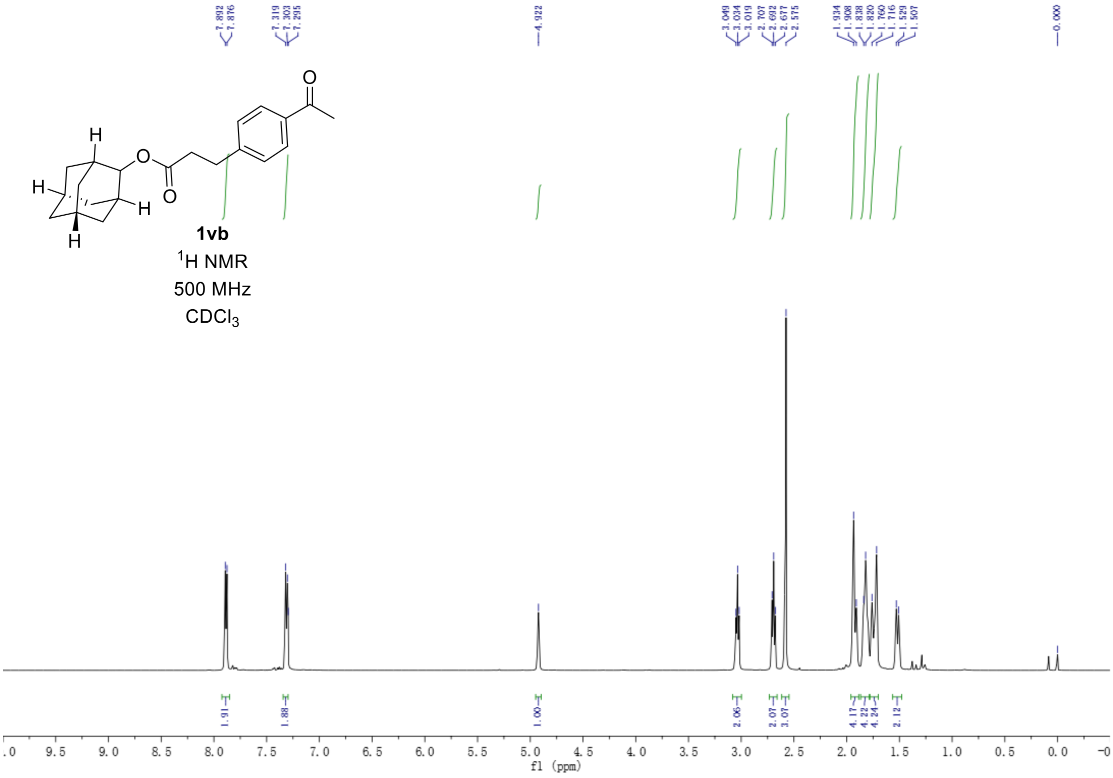

Supplementary Fig. 9 ^{1}H NMR spectrum of compound **1vb**

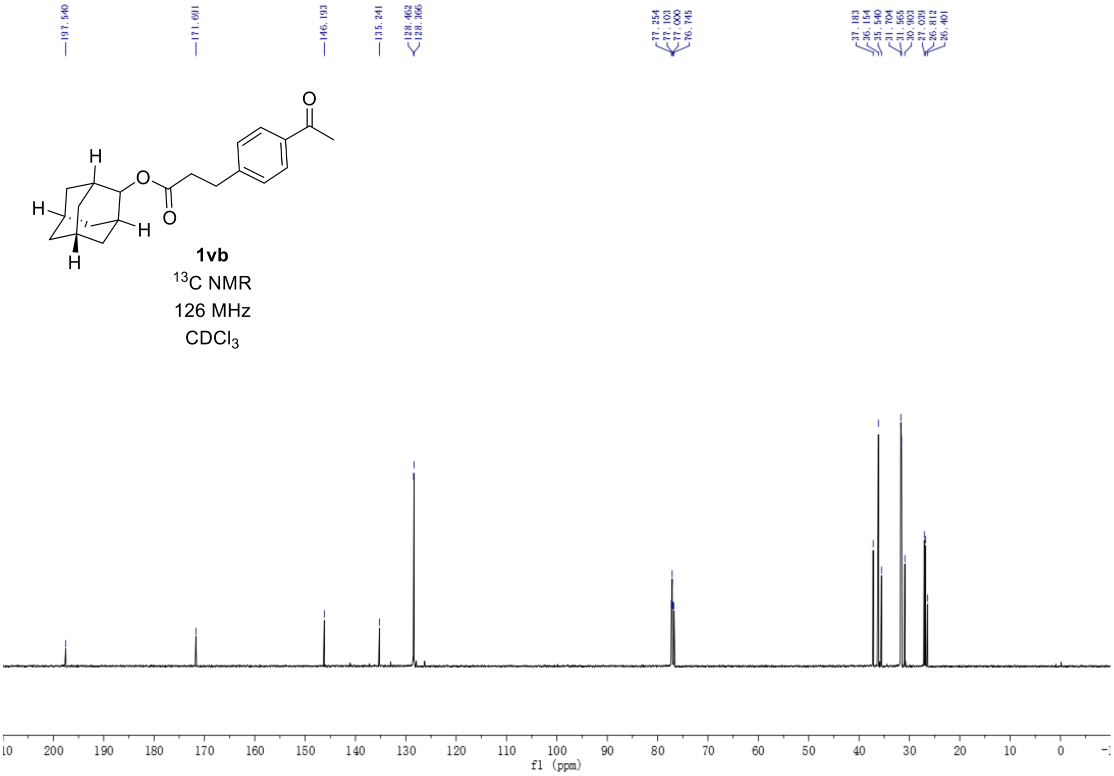

Supplementary Fig. 10 ^{13}C NMR spectrum of compound **1vb**

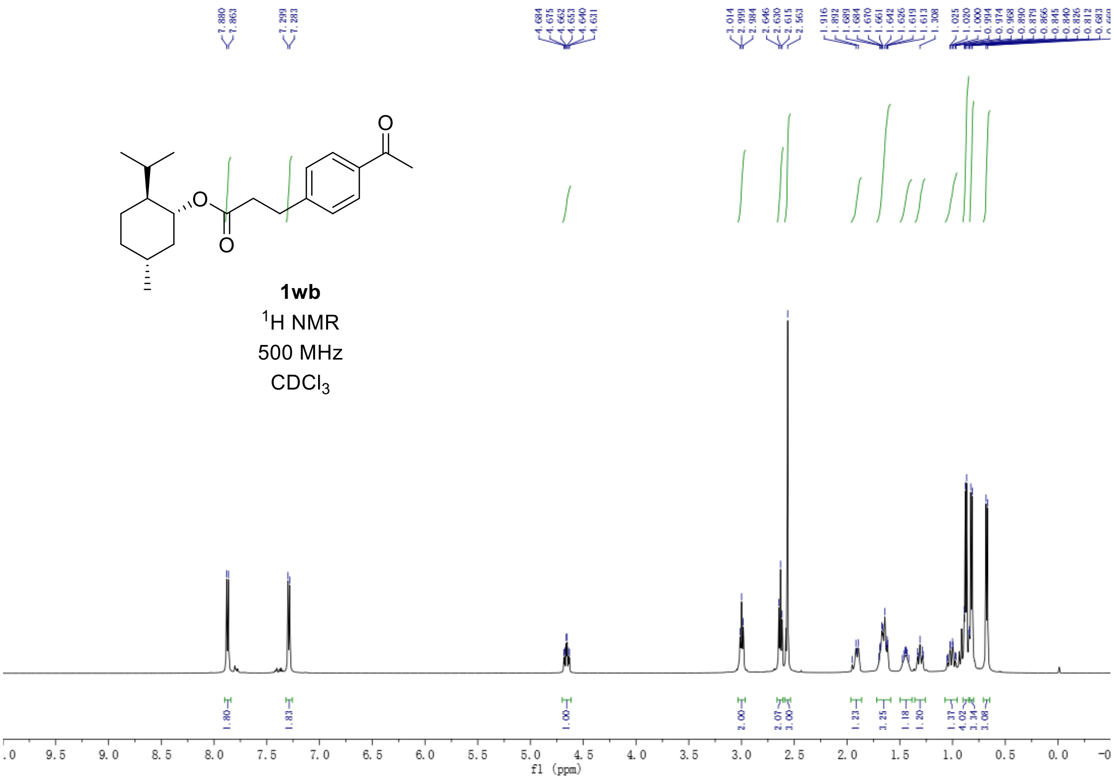

Supplementary Fig. 11 ^{1}H NMR spectrum of compound **1wb**

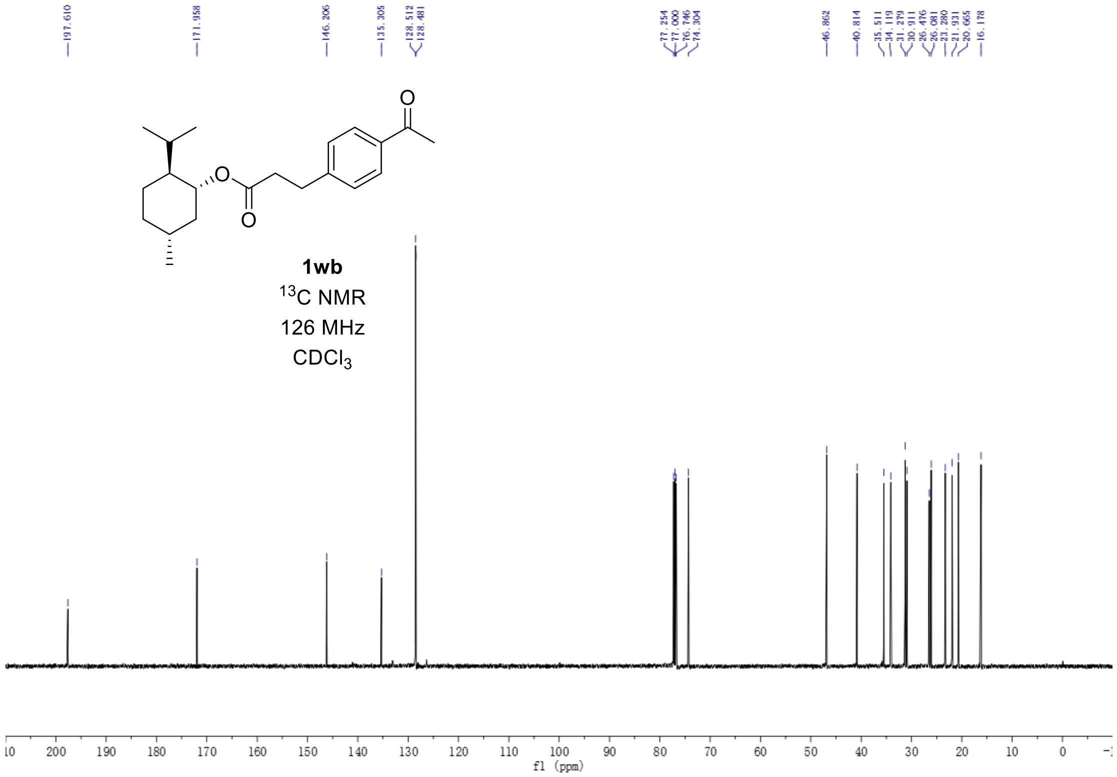

Supplementary Fig. 12 ^{13}C NMR spectrum of compound **1wb**

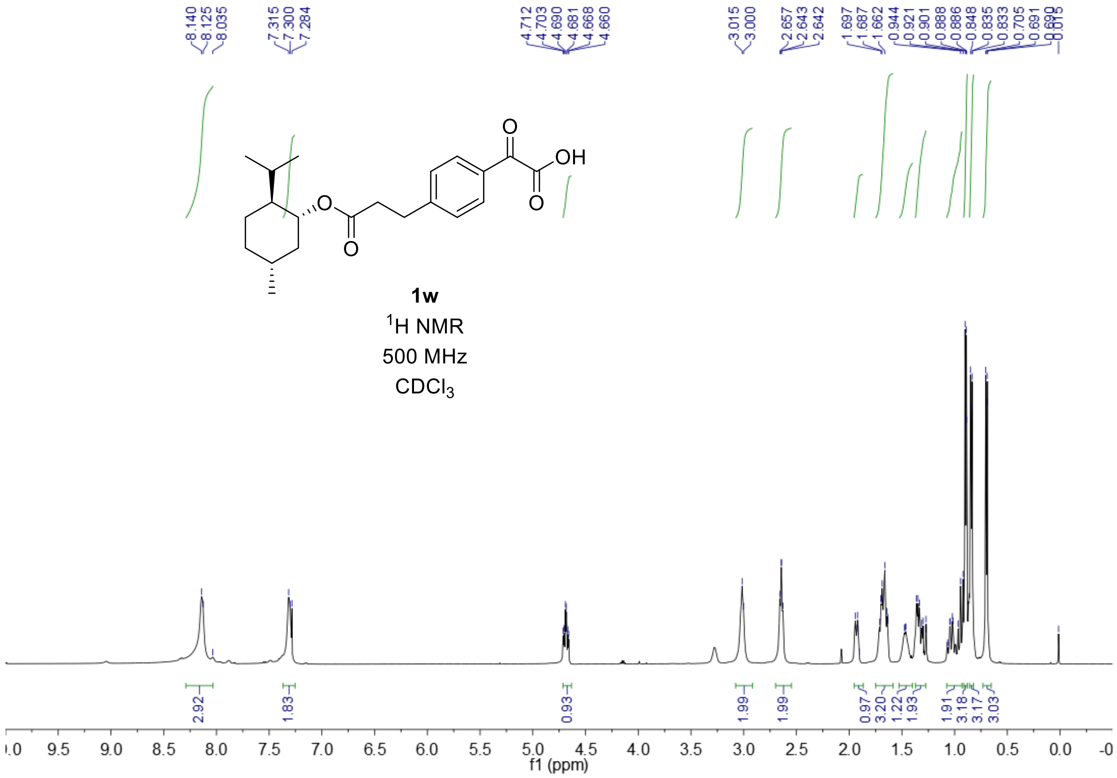

Supplementary Fig. 13 ^{1}H NMR spectrum of compound **1w**

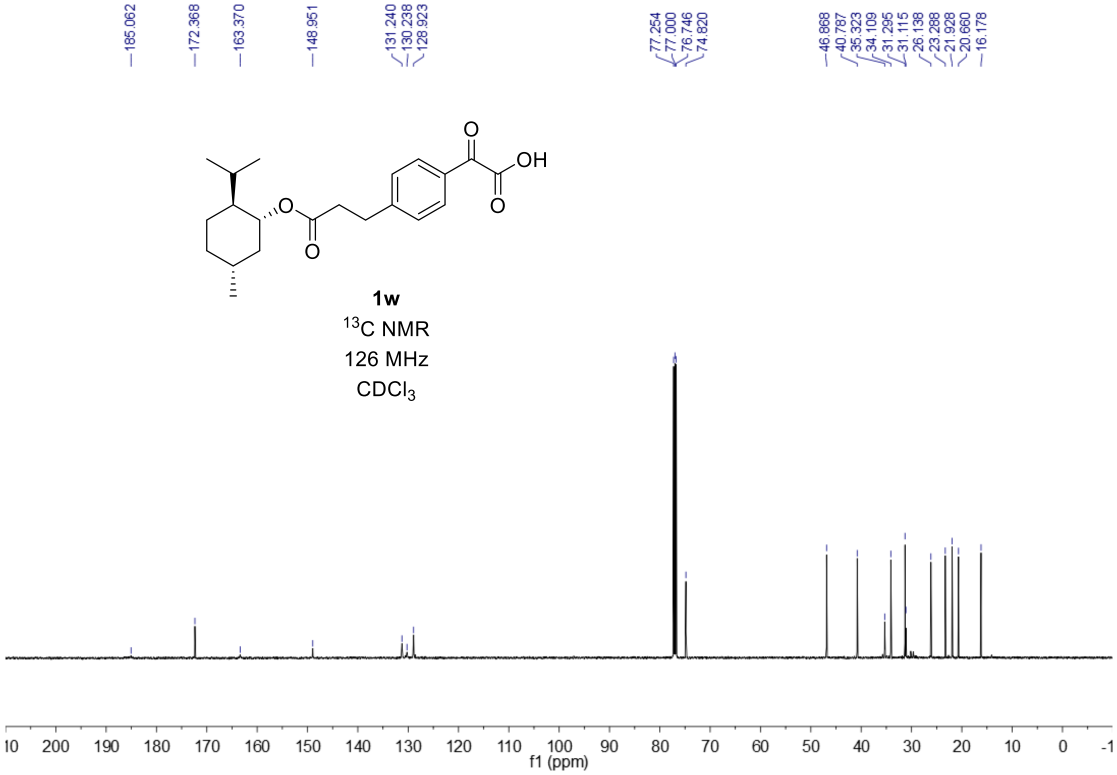

Supplementary Fig. 14 ^{13}C NMR spectrum of compound **1w**

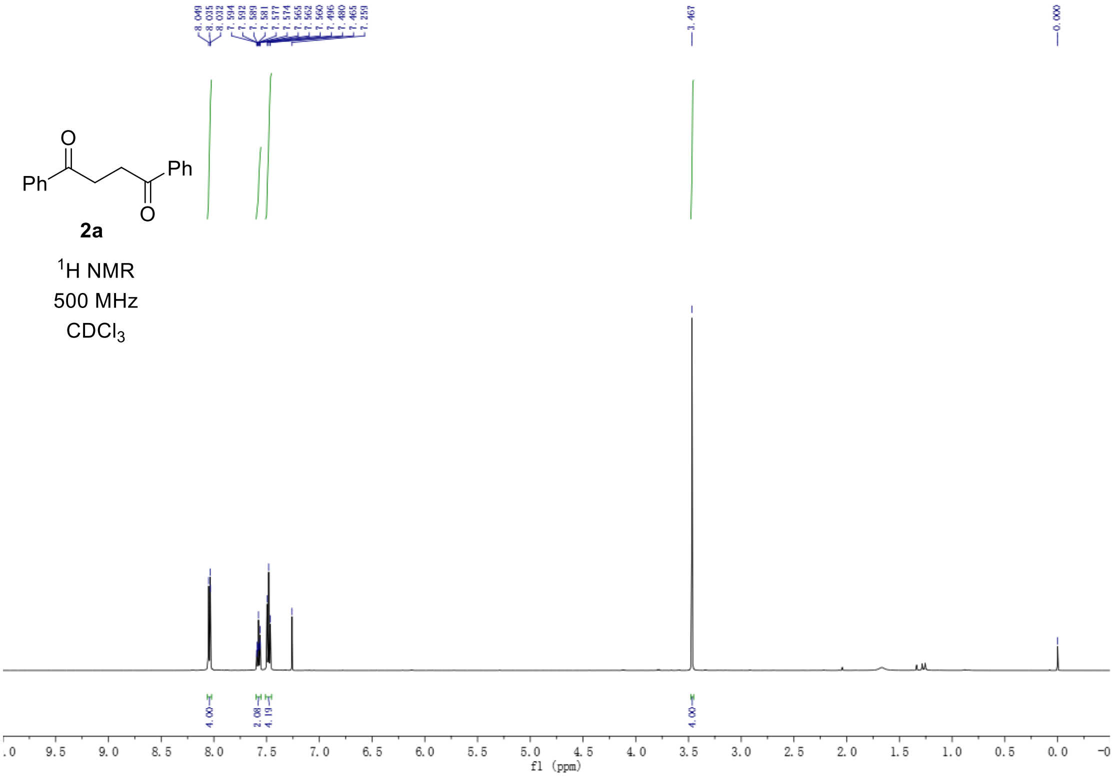

Supplementary Fig. 15 ^{1}H NMR spectrum of compound **2a**

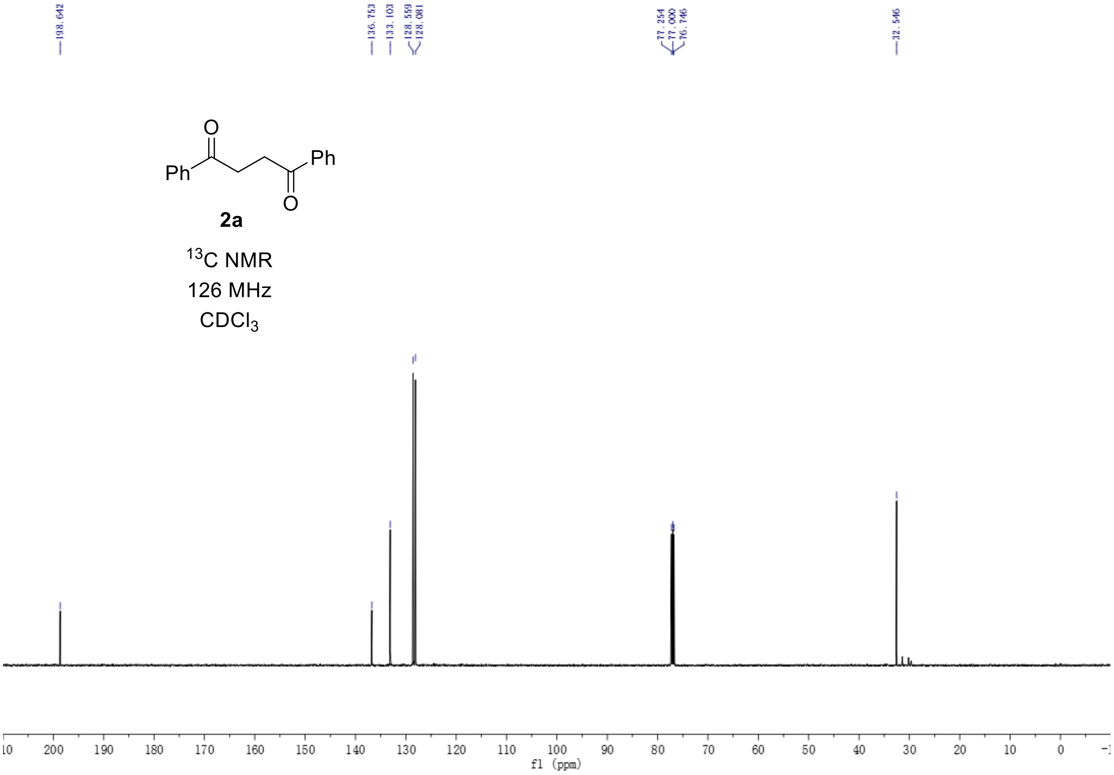

Supplementary Fig. 16 ^{13}C NMR spectrum of compound **2a**

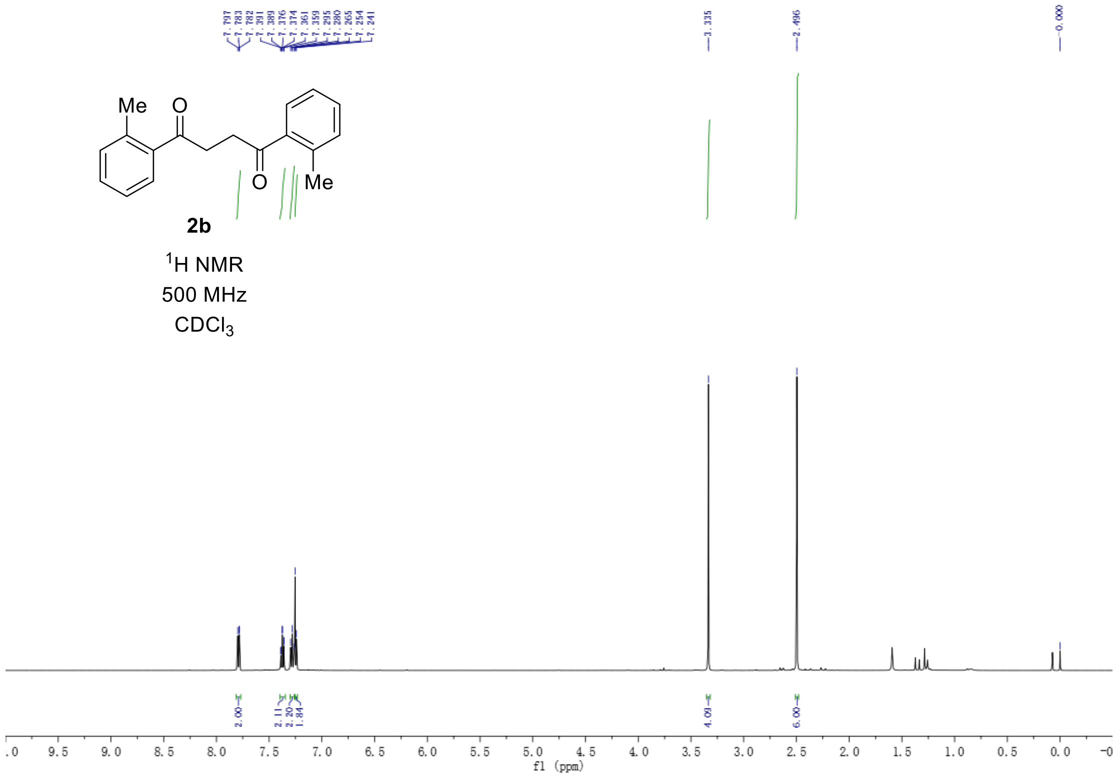

Supplementary Fig. 17 ^{1}H NMR spectrum of compound **2b**

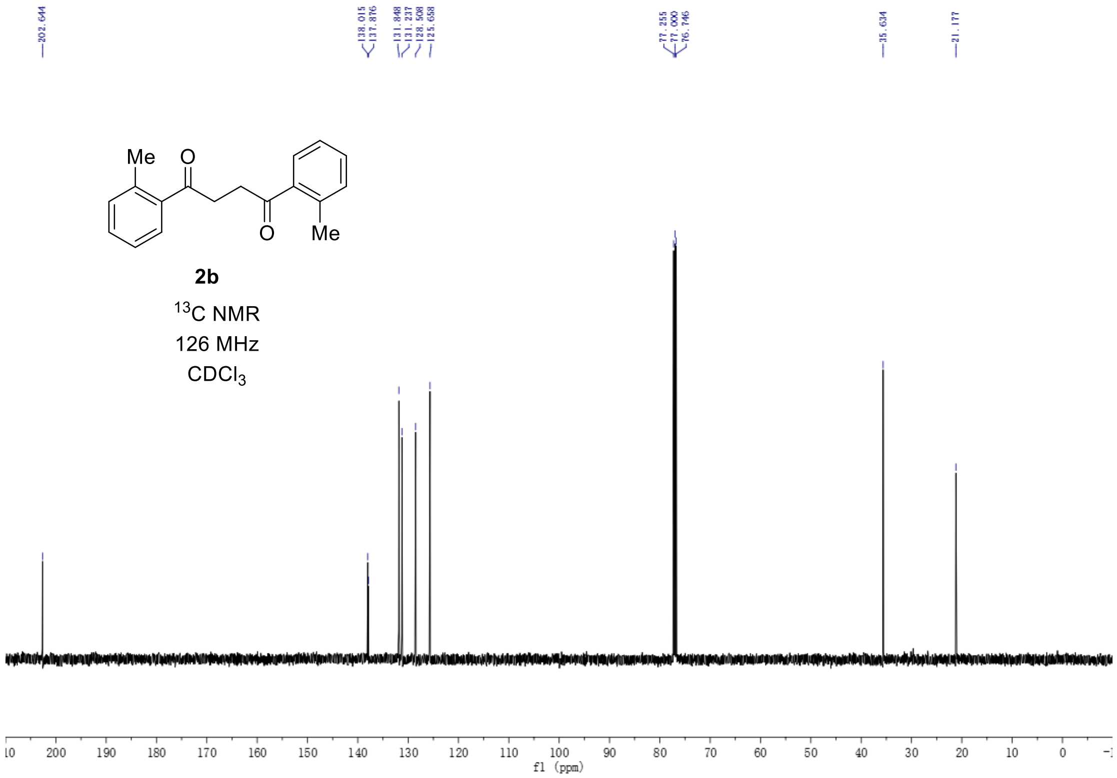

Supplementary Fig. 18 ^{13}C NMR spectrum of compound **2b**

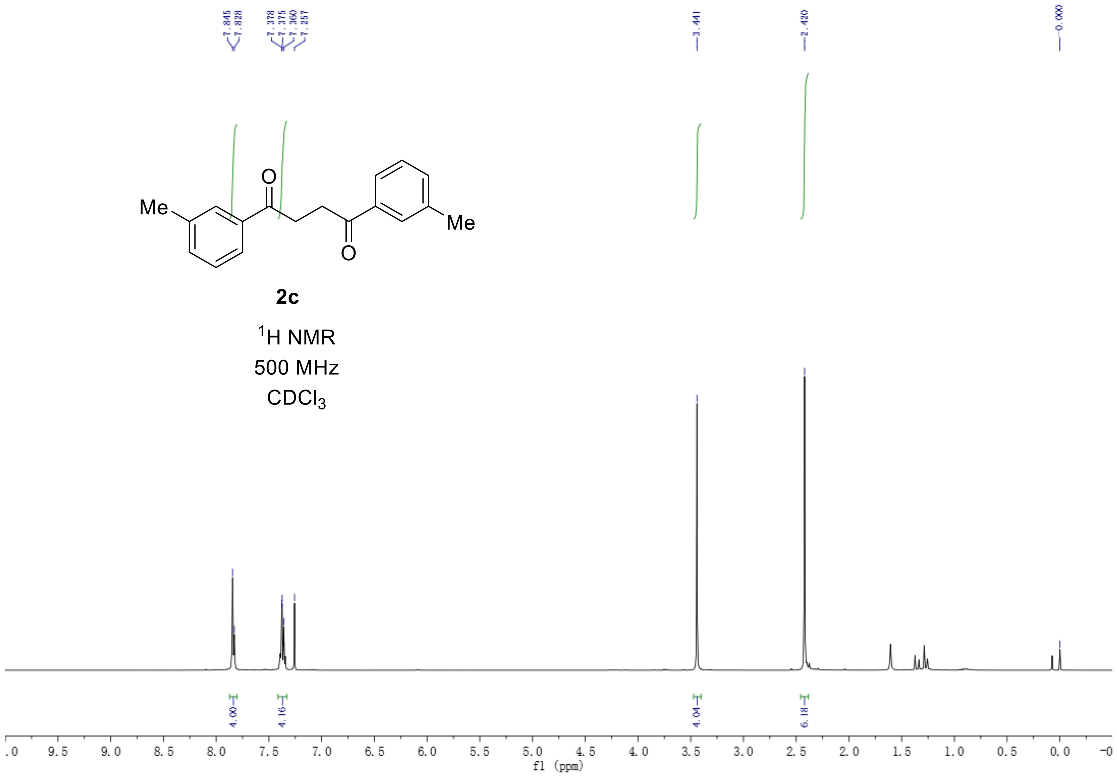

Supplementary Fig. 19 ^{1}H NMR spectrum of compound **2c**

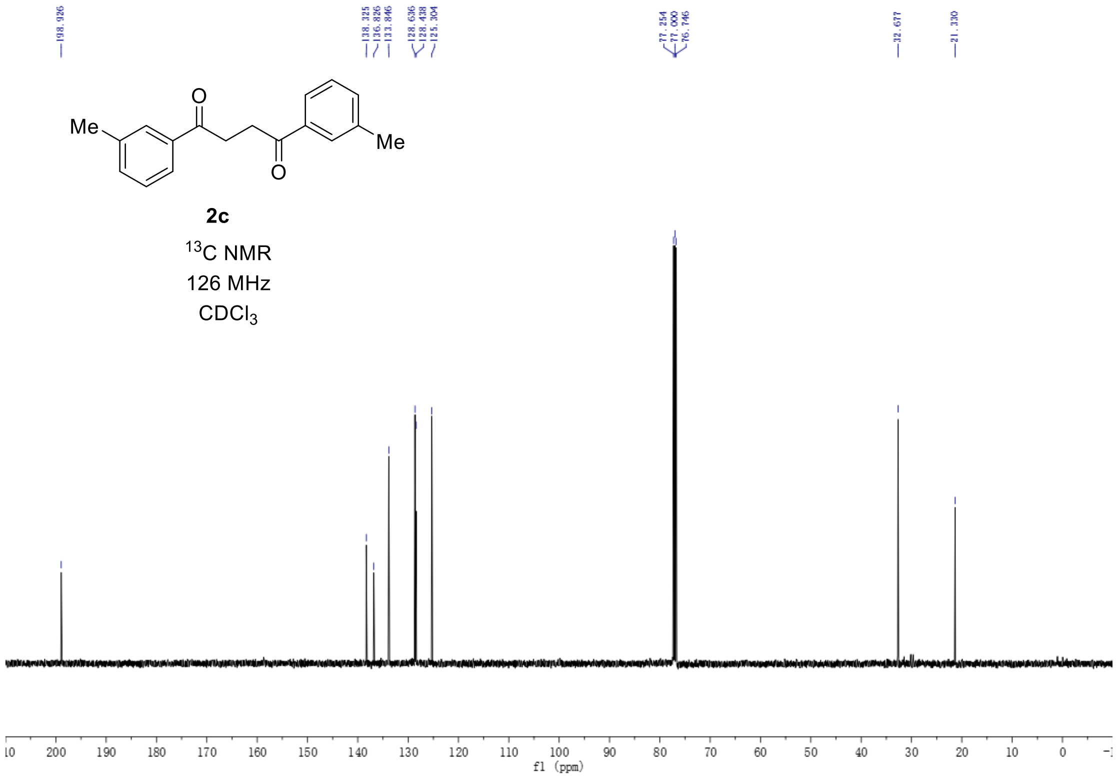

Supplementary Fig. 20 ^{13}C NMR spectrum of compound **2c**

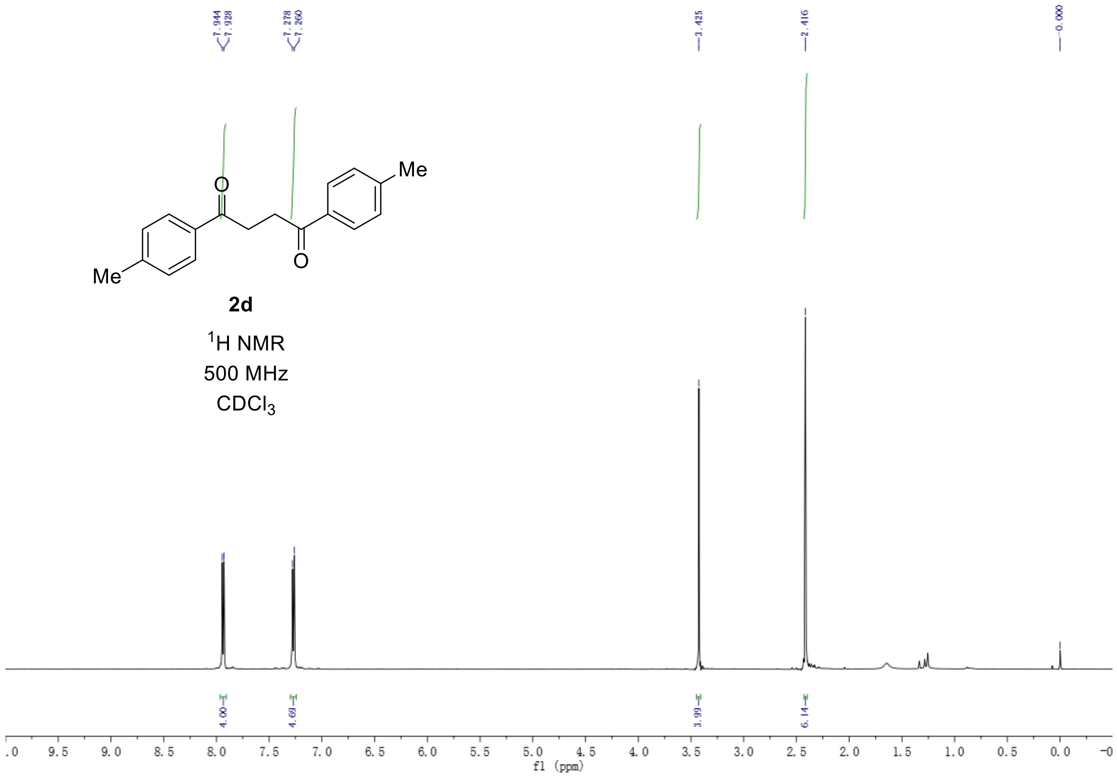

Supplementary Fig. 21 ^{1}H NMR spectrum of compound **2d**

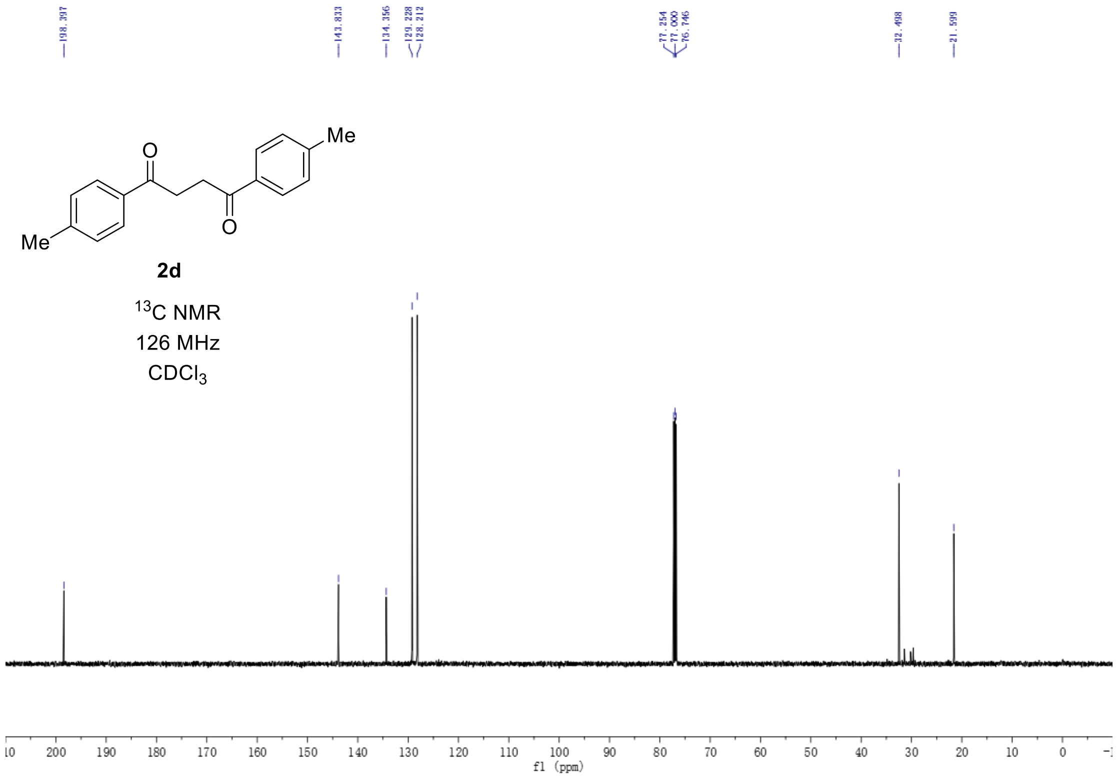

Supplementary Fig. 22 ^{13}C NMR spectrum of compound **2d**

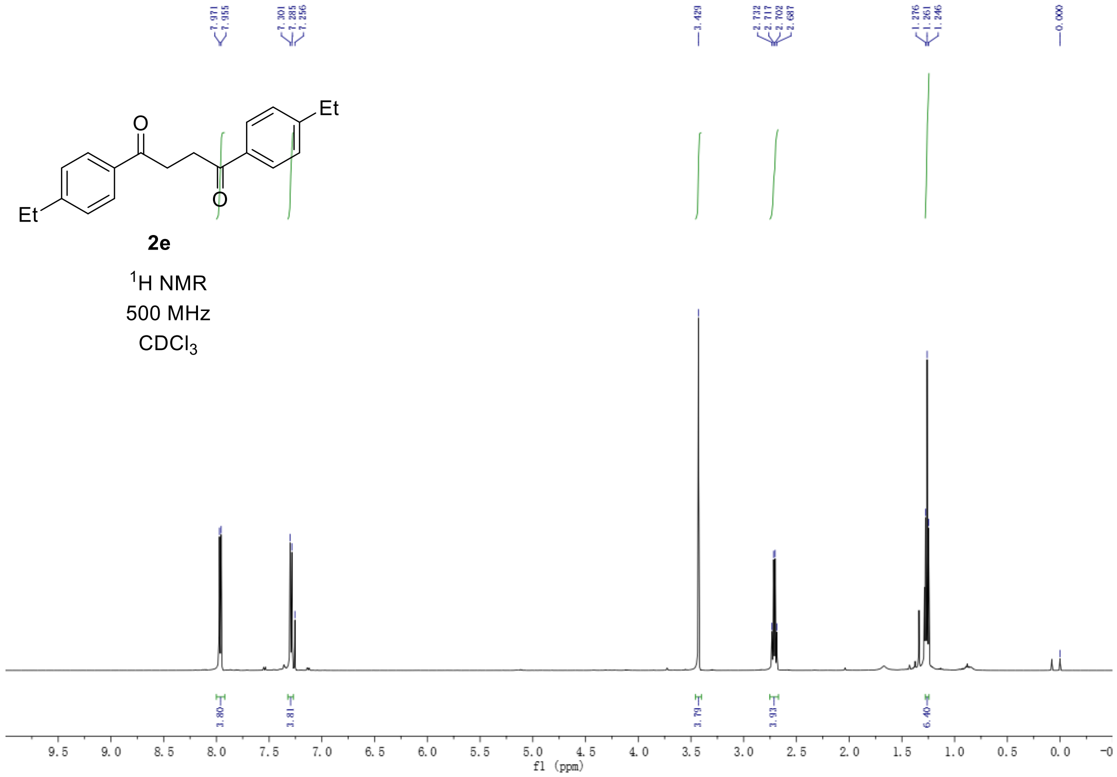

Supplementary Fig. 23 ^{1}H NMR spectrum of compound **2e**

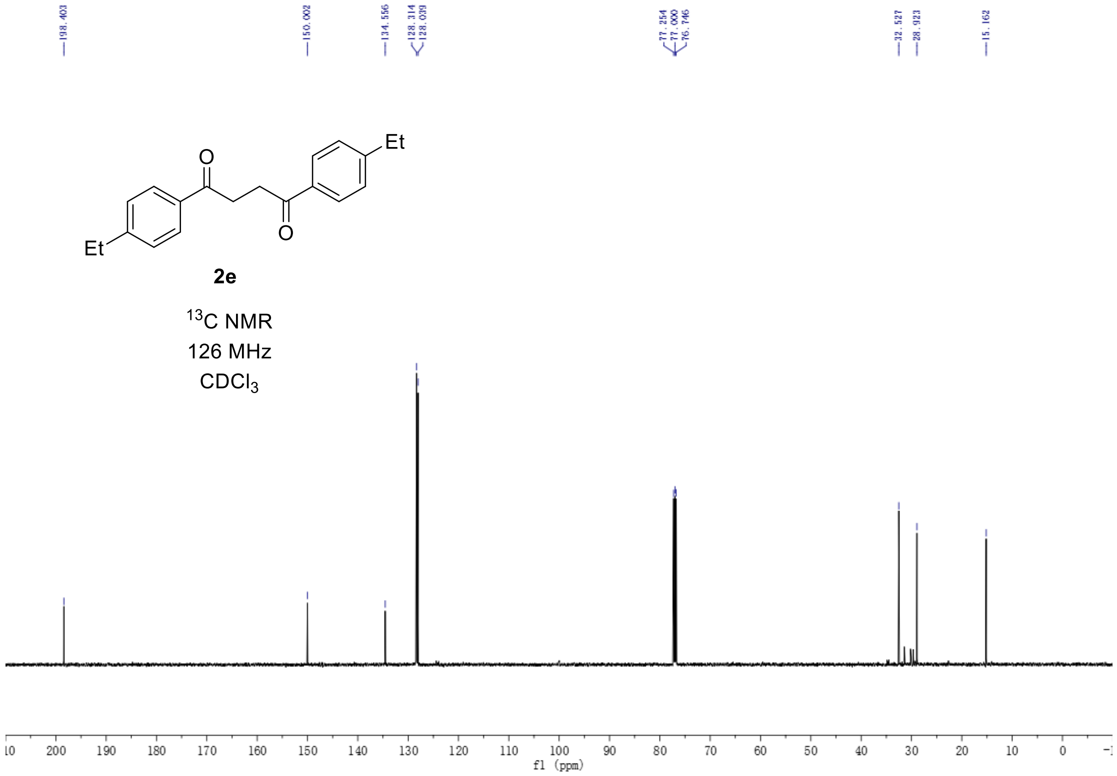

Supplementary Fig. 24 ^{13}C NMR spectrum of compound **2e**

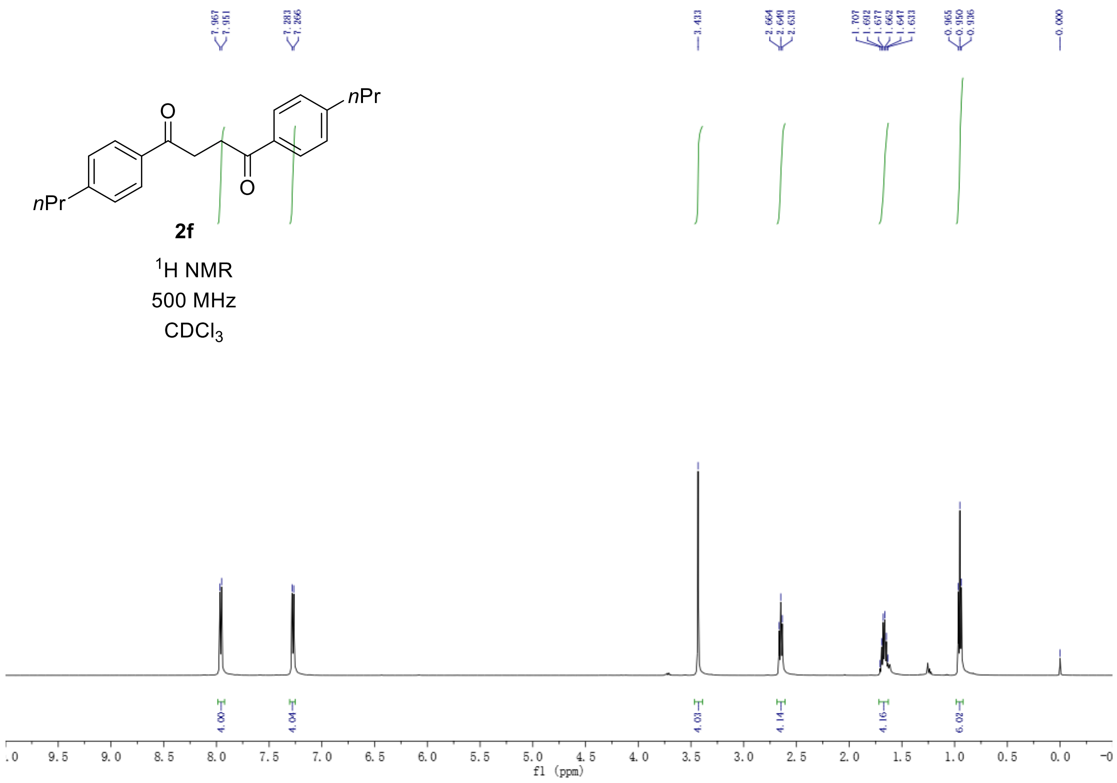

Supplementary Fig. 25 ^{1}H NMR spectrum of compound **2f**

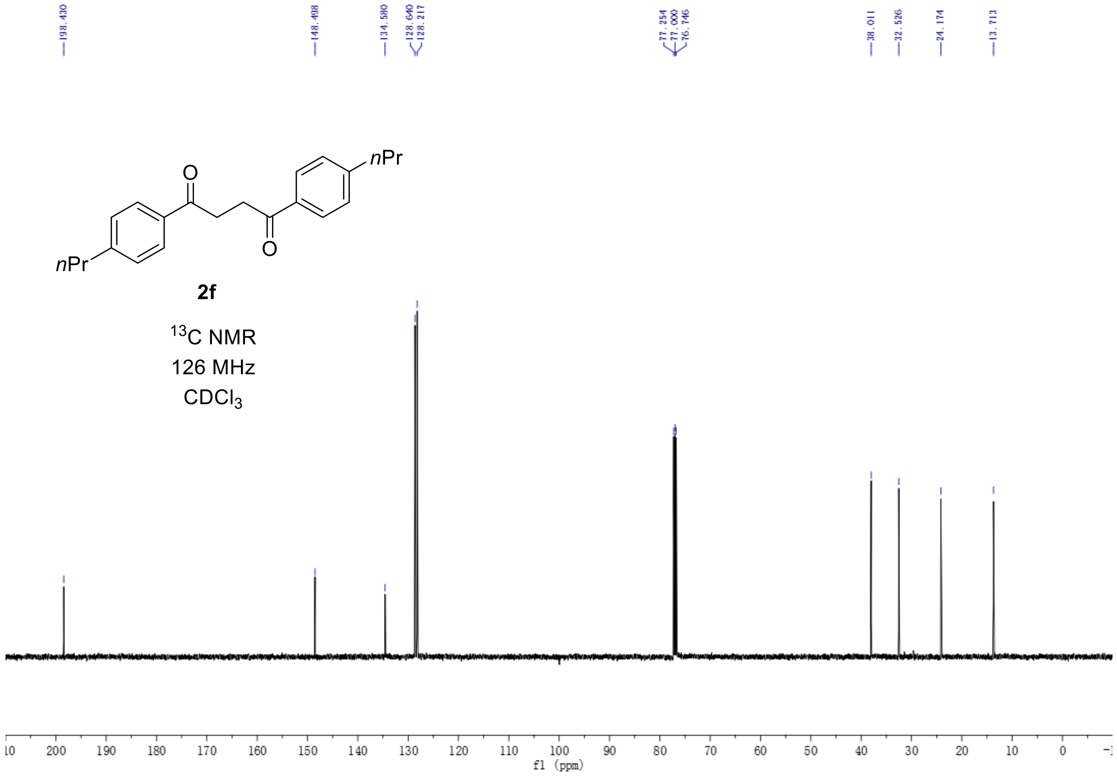

Supplementary Fig. 26 ^{13}C NMR spectrum of compound **2f**

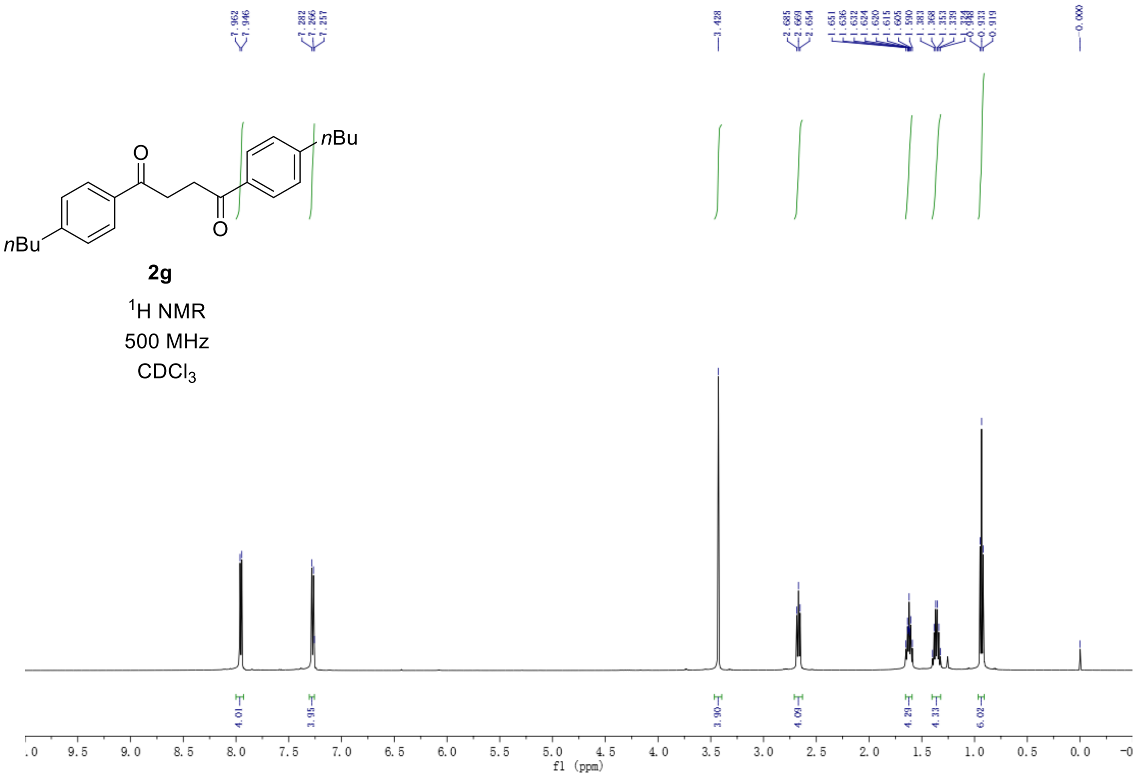

Supplementary Fig. 27 ^{1}H NMR spectrum of compound **2g**

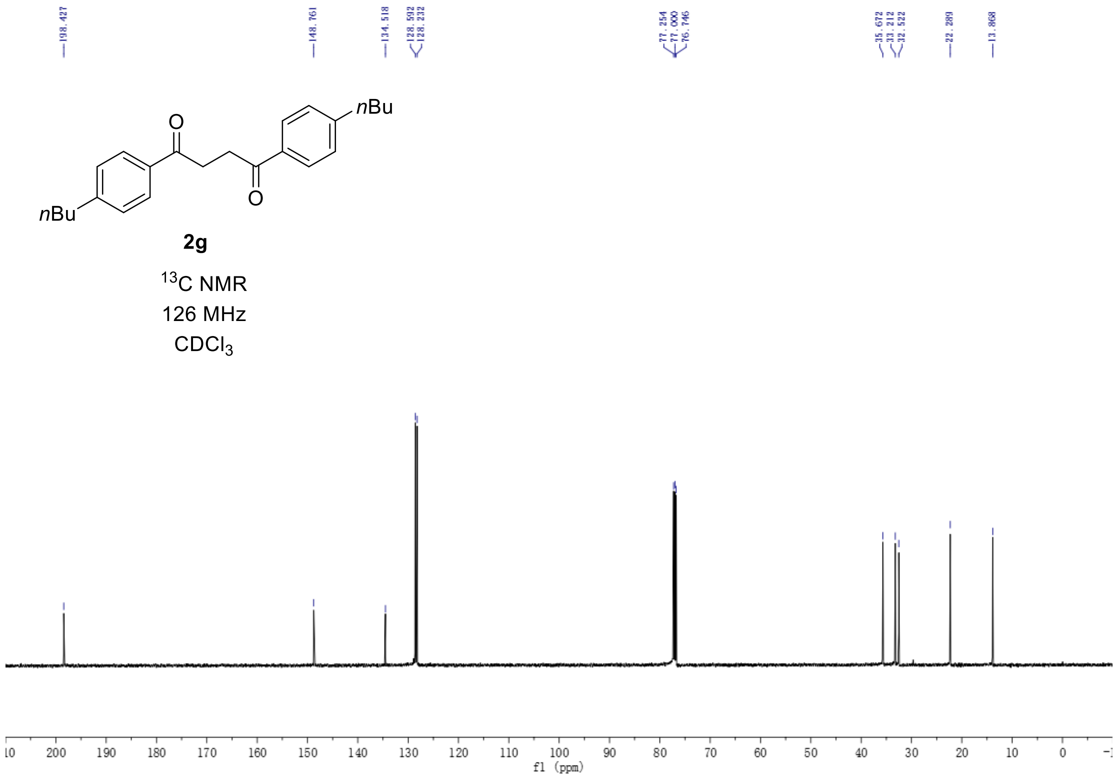

Supplementary Fig. 28 ^{13}C NMR spectrum of compound **2g**

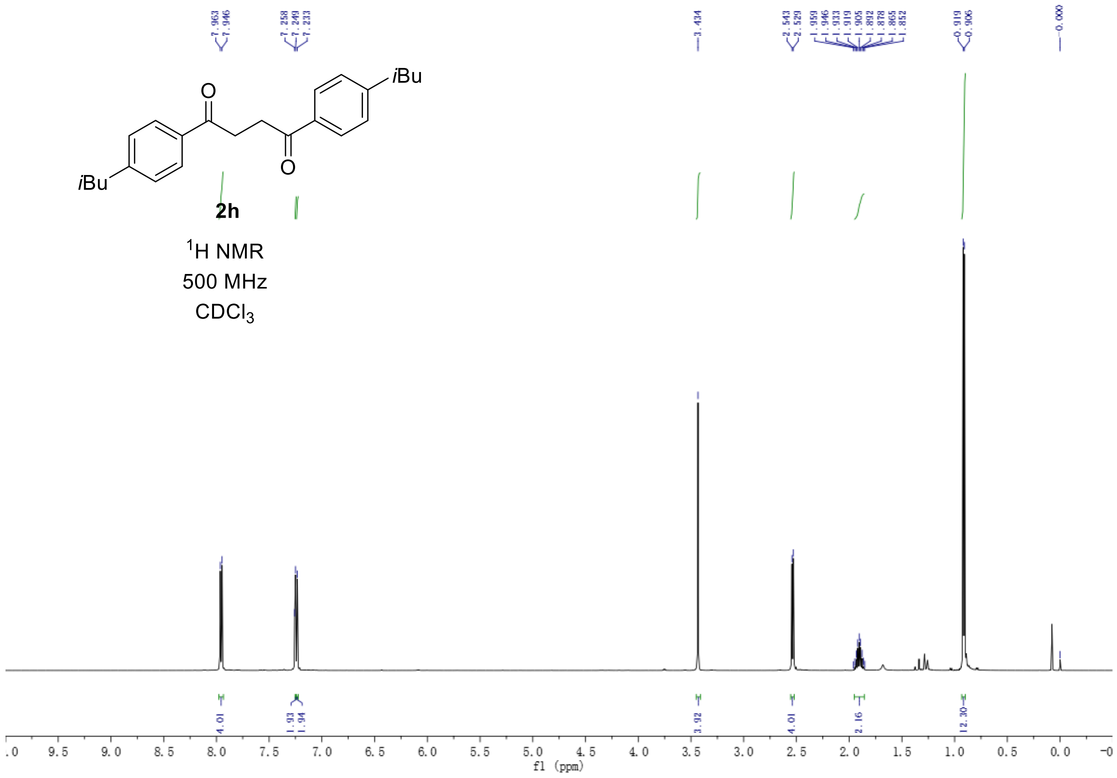

Supplementary Fig. 29 ^{1}H NMR spectrum of compound **2h**

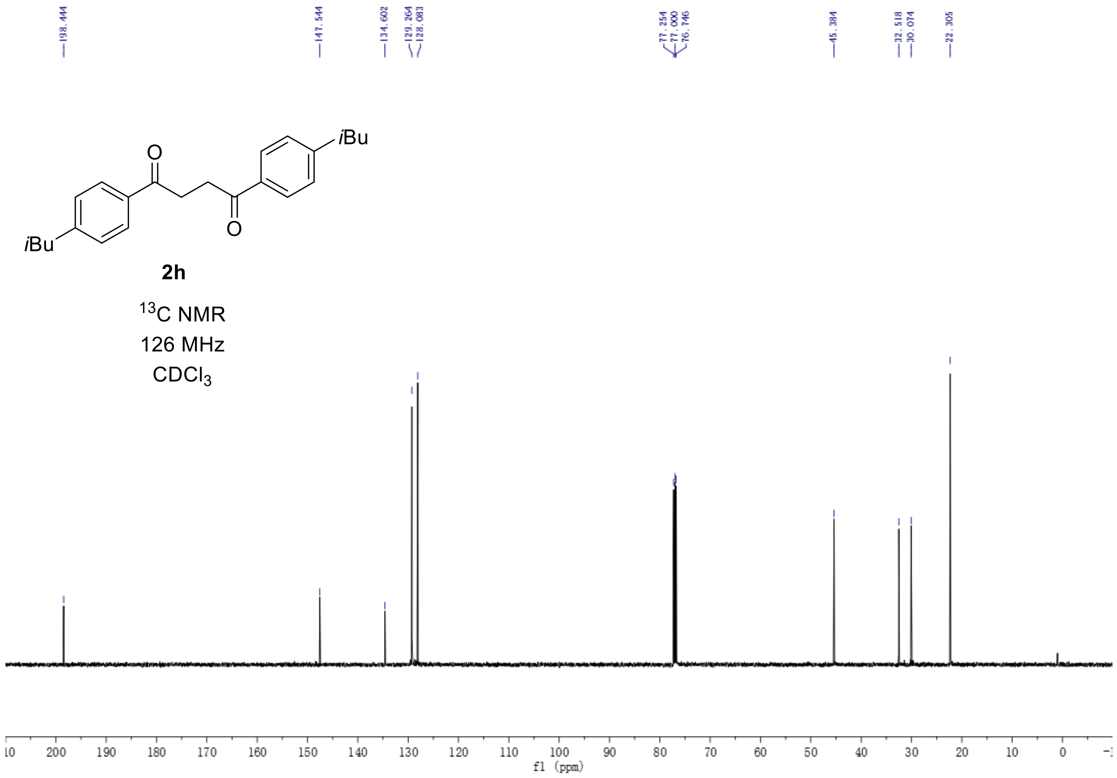

Supplementary Fig. 30 ^{13}C NMR spectrum of compound **2h**

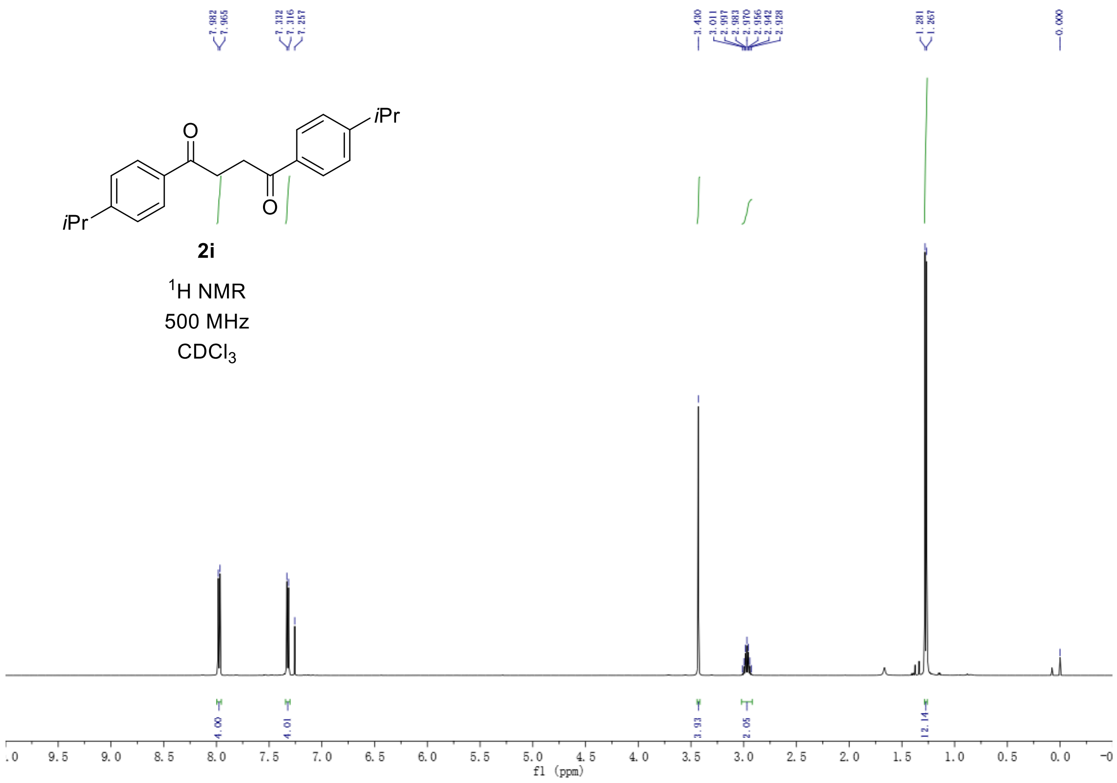

Supplementary Fig. 31 ^{1}H NMR spectrum of compound **2i**

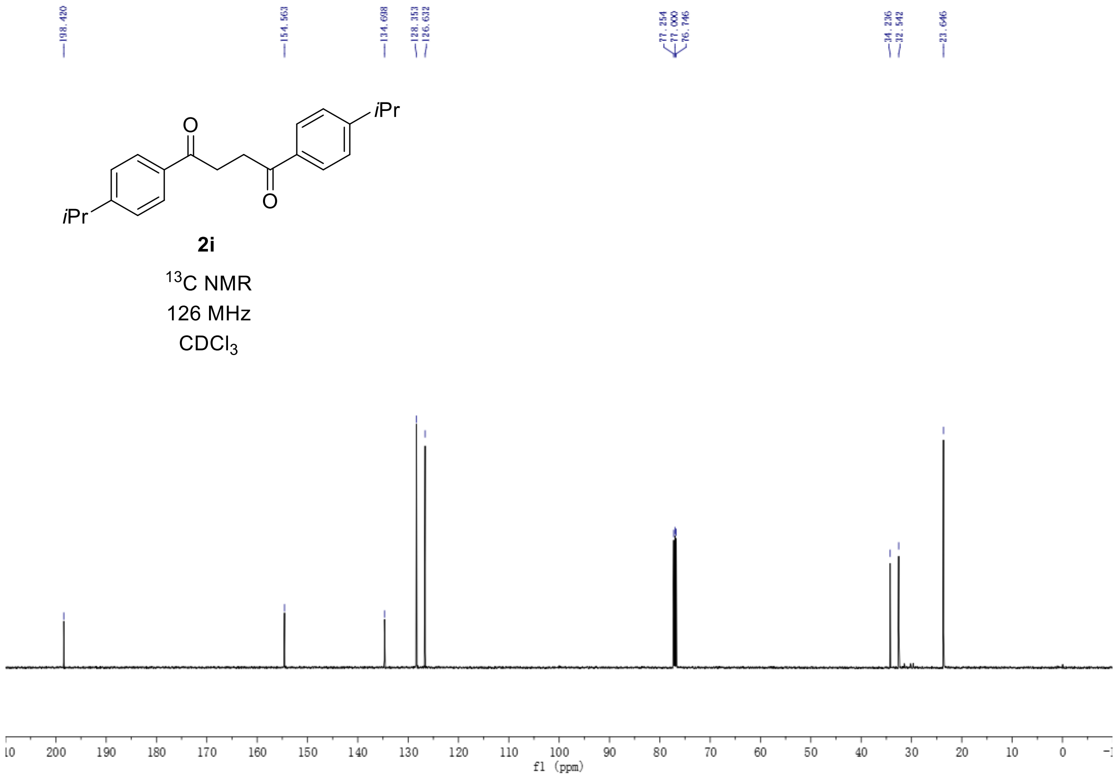

Supplementary Fig. 32 ^{13}C NMR spectrum of compound **2i**

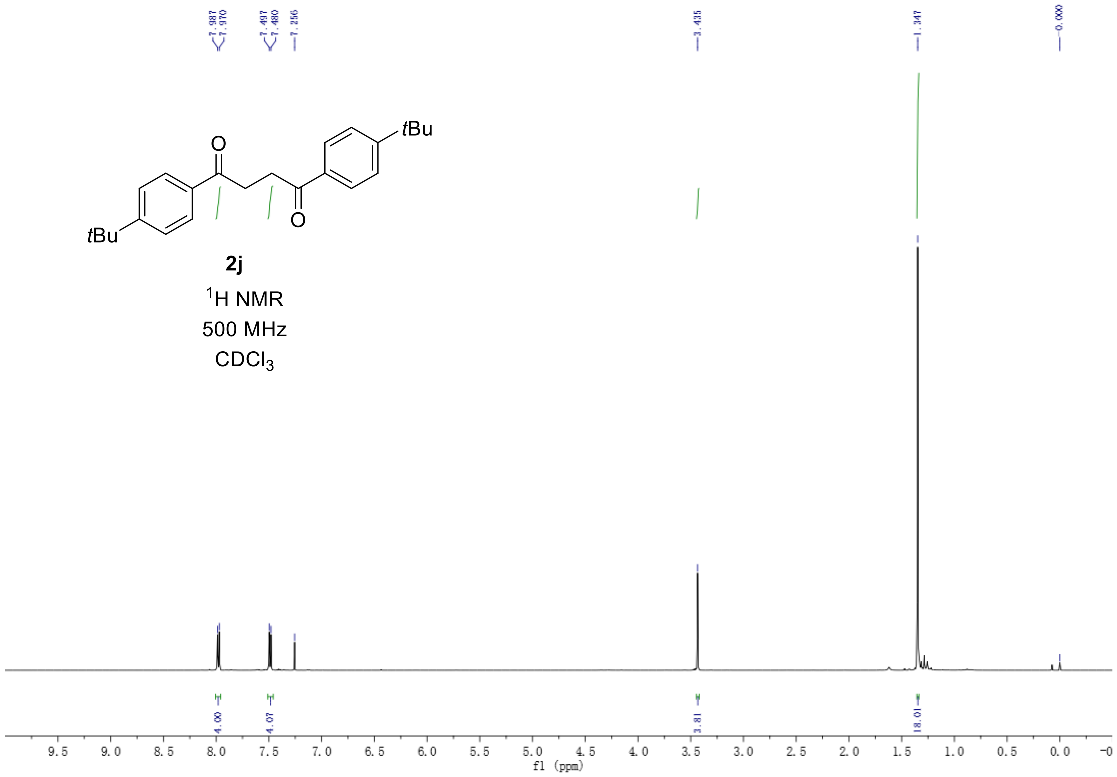

Supplementary Fig. 33 ^{1}H NMR spectrum of compound **2j**

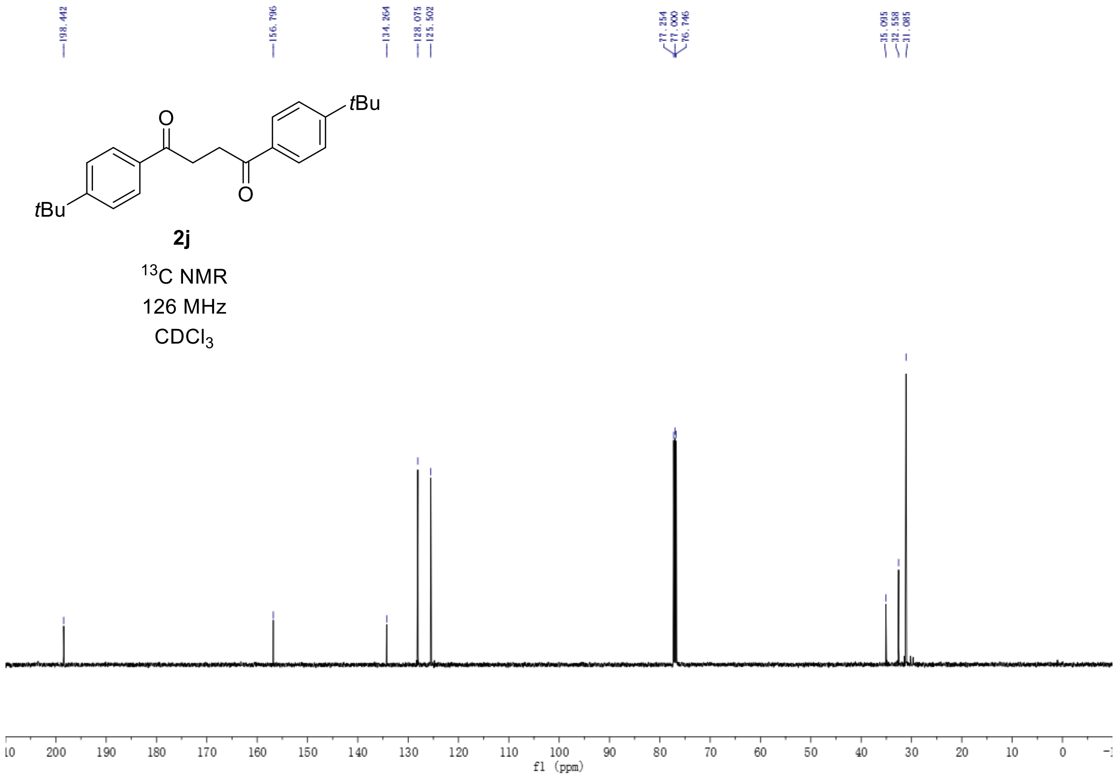

Supplementary Fig. 34 ^{13}C NMR spectrum of compound **2j**

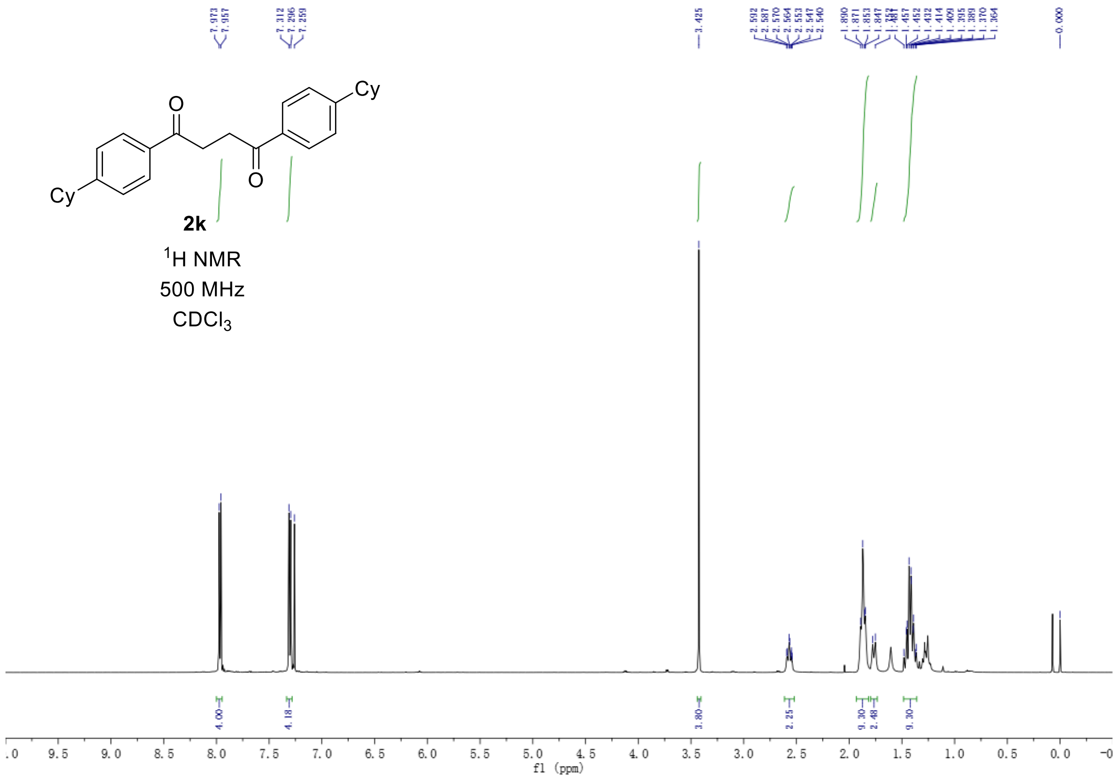

Supplementary Fig. 35 ^{1}H NMR spectrum of compound **2k**

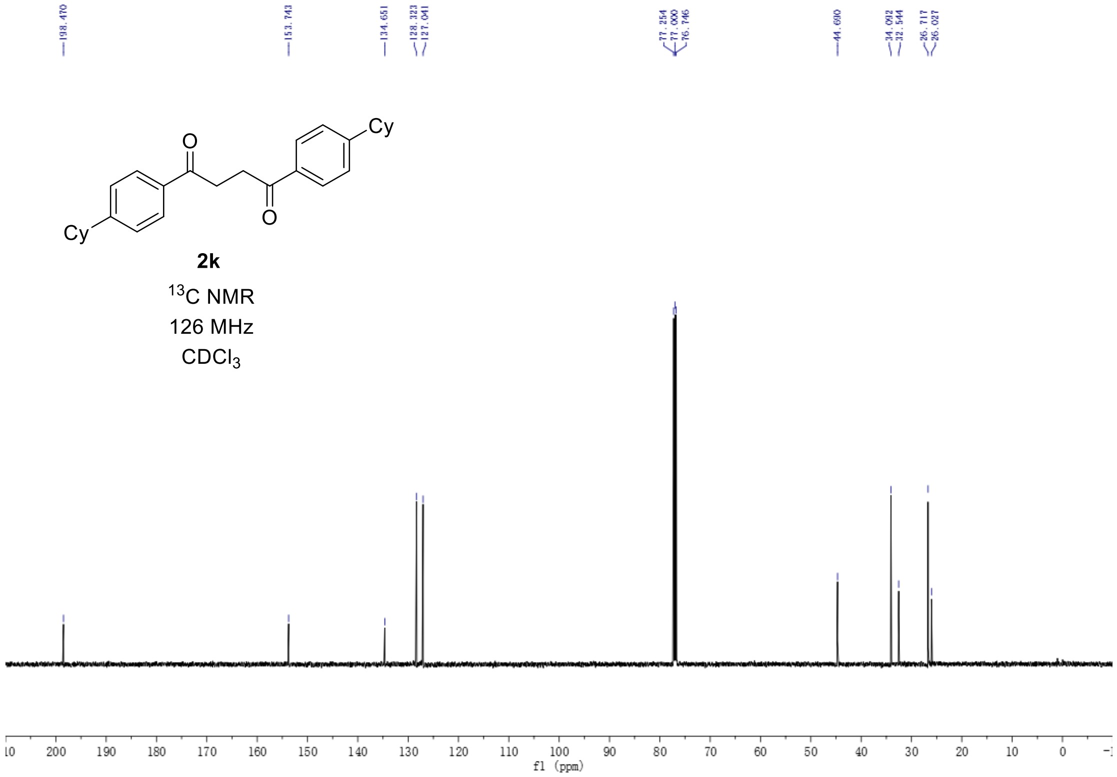

Supplementary Fig. 36 ^{13}C NMR spectrum of compound **2k**

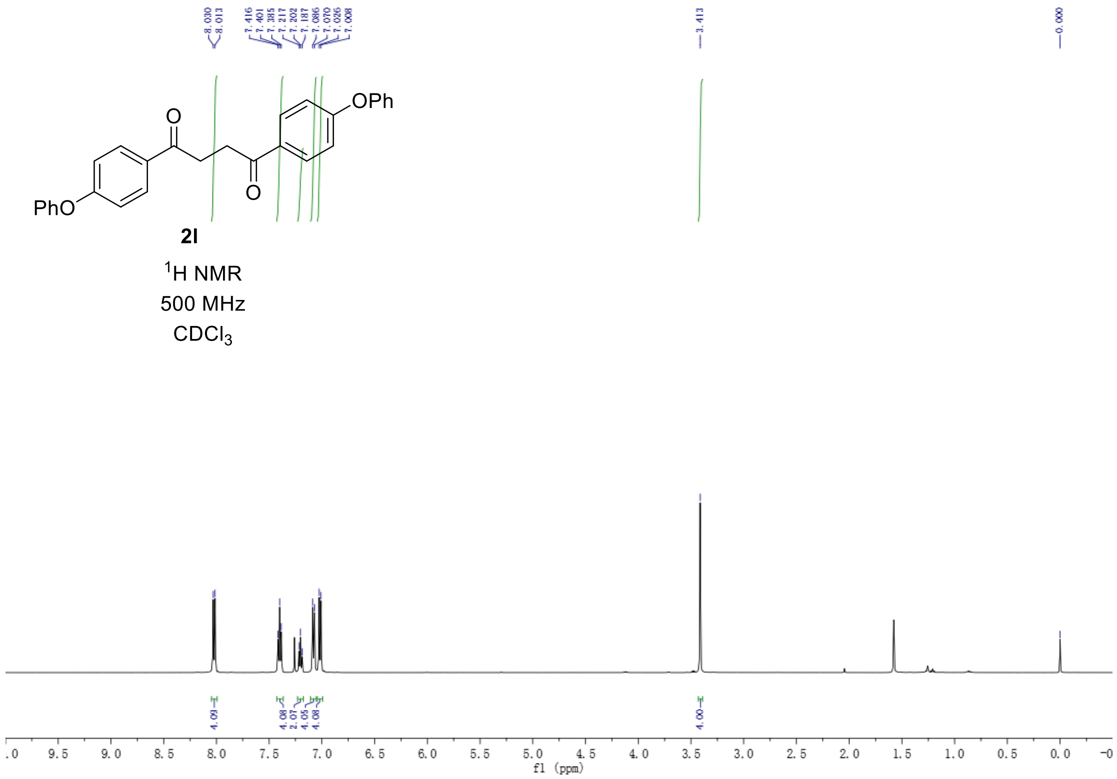

Supplementary Fig. 37 ^{1}H NMR spectrum of compound **2l**

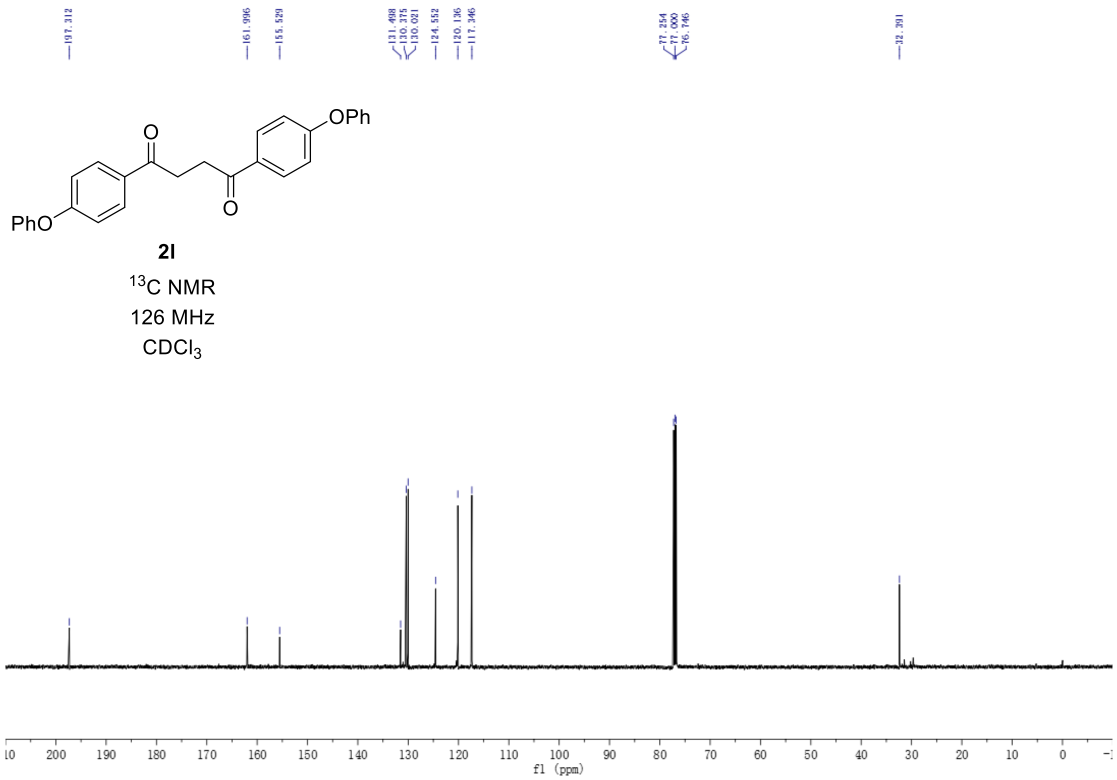

Supplementary Fig. 38 ^{13}C NMR spectrum of compound **2l**

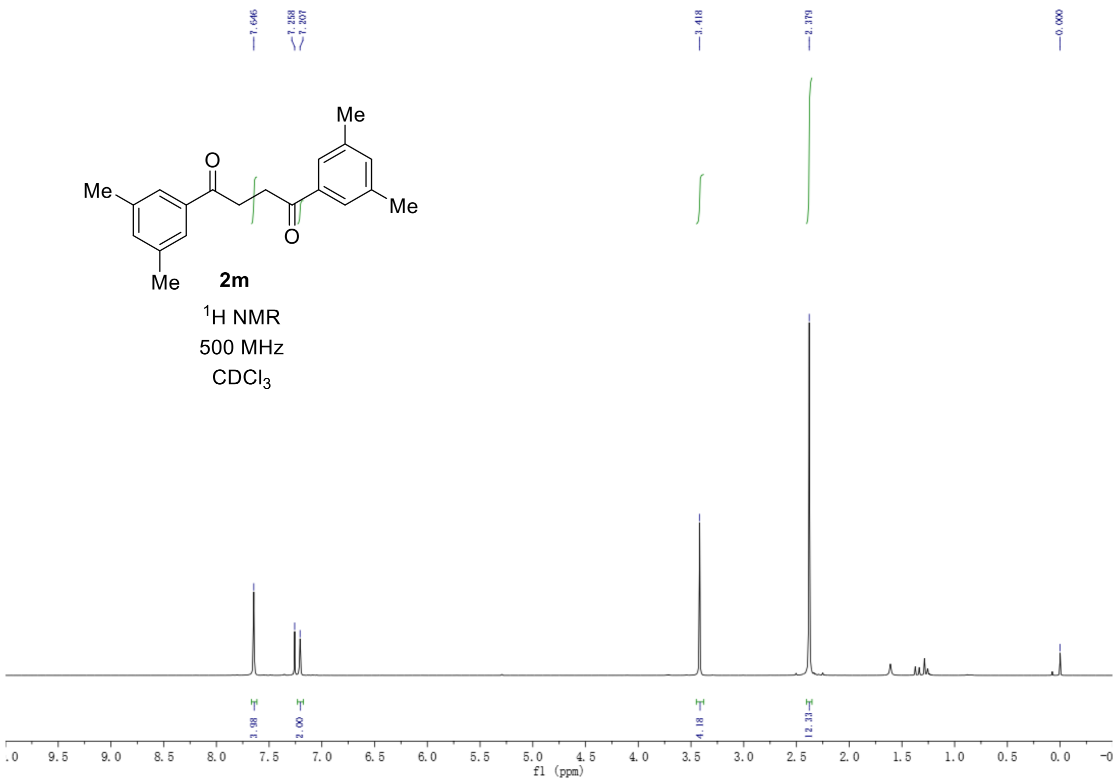

Supplementary Fig. 39 ^{1}H NMR spectrum of compound **2m**

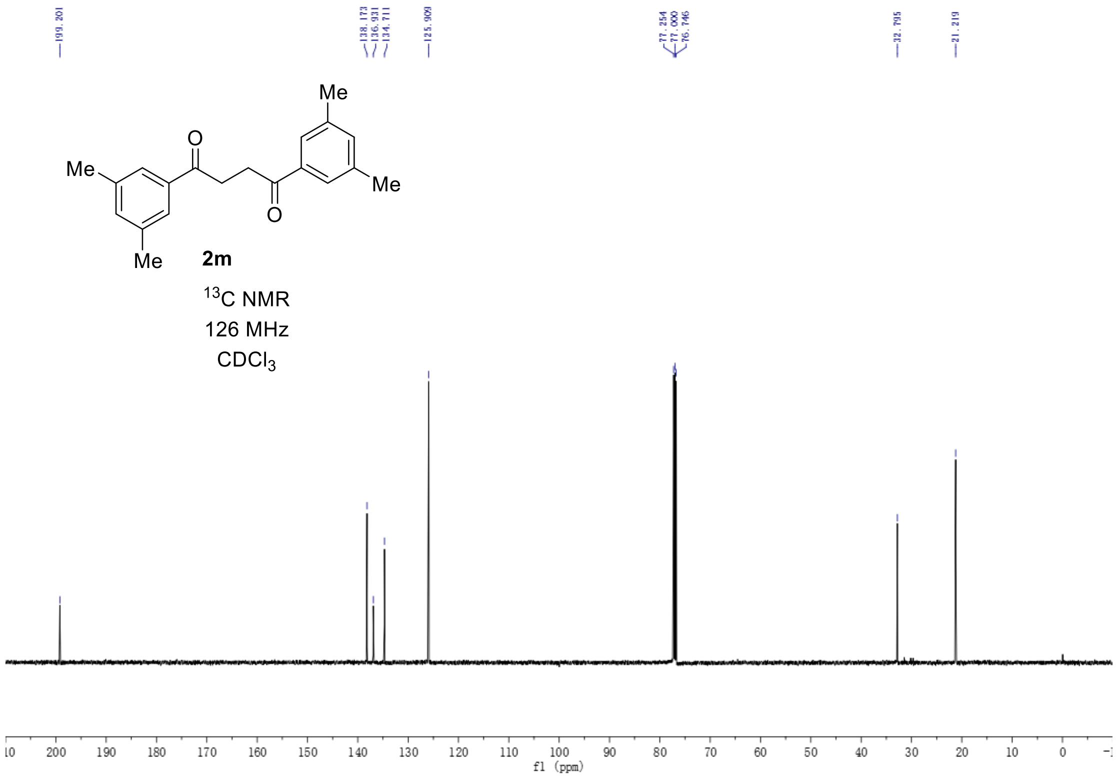

Supplementary Fig. 40 ^{13}C NMR spectrum of compound **2m**

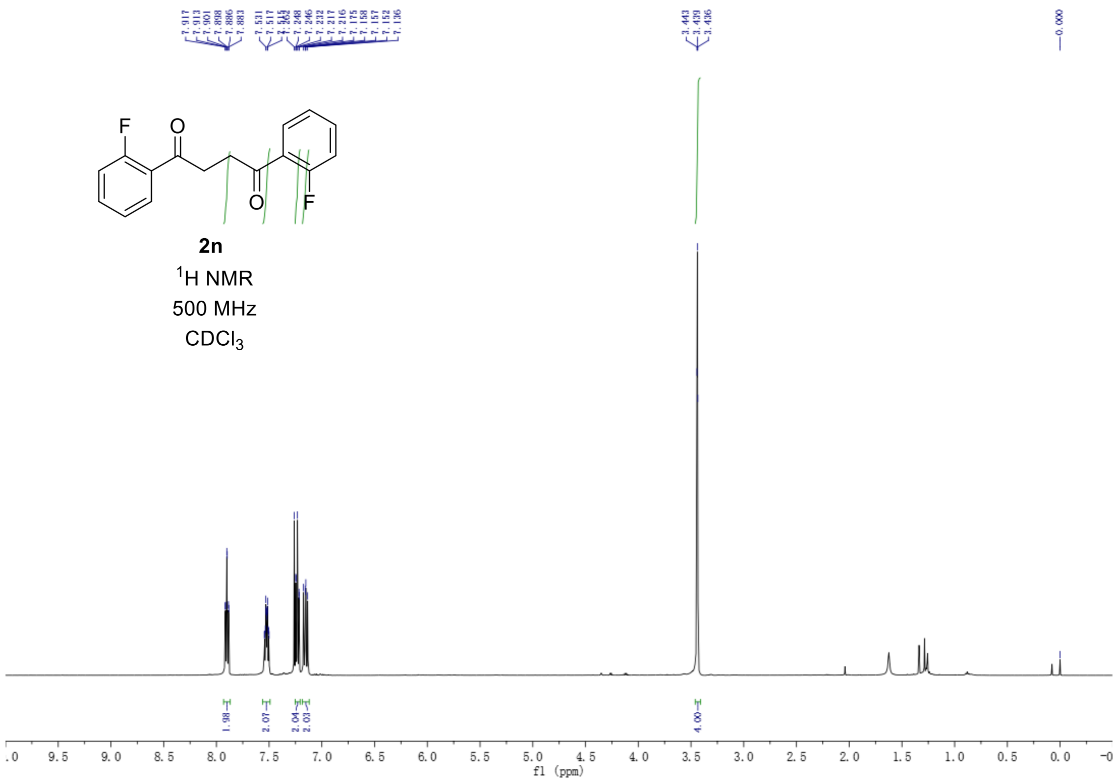

Supplementary Fig. 41 ^{1}H NMR spectrum of compound **2n**

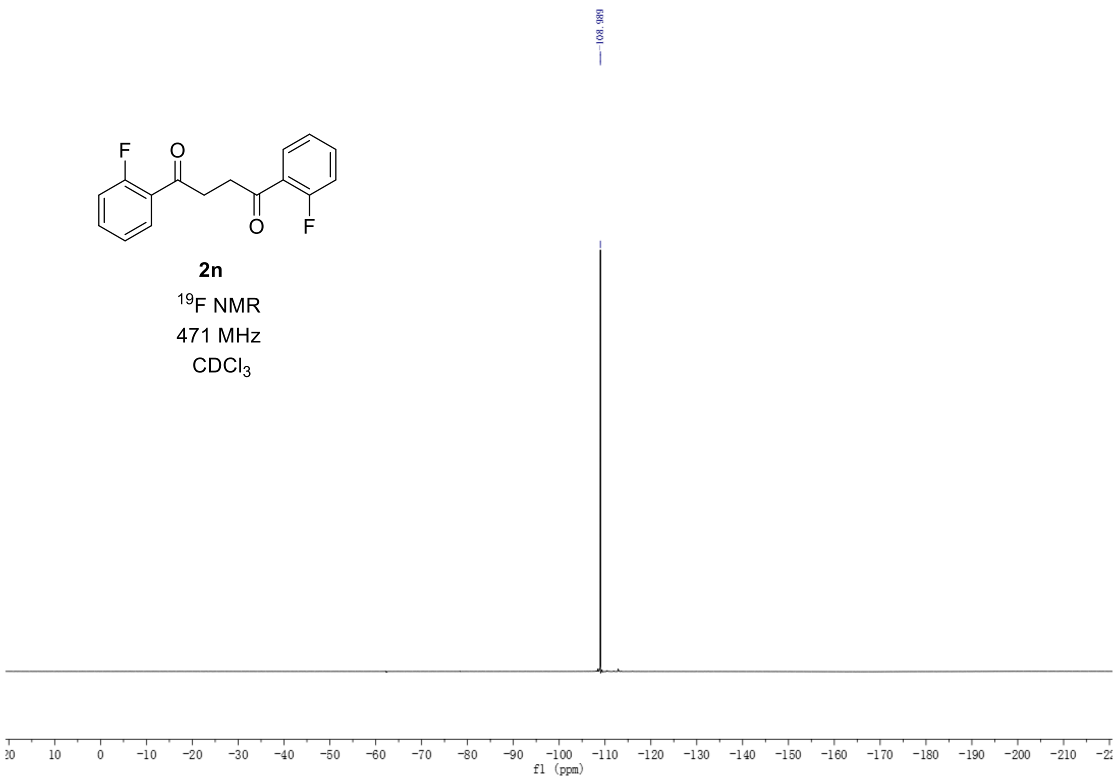

Supplementary Fig. 42 ^{19}F NMR spectrum of compound **2n**

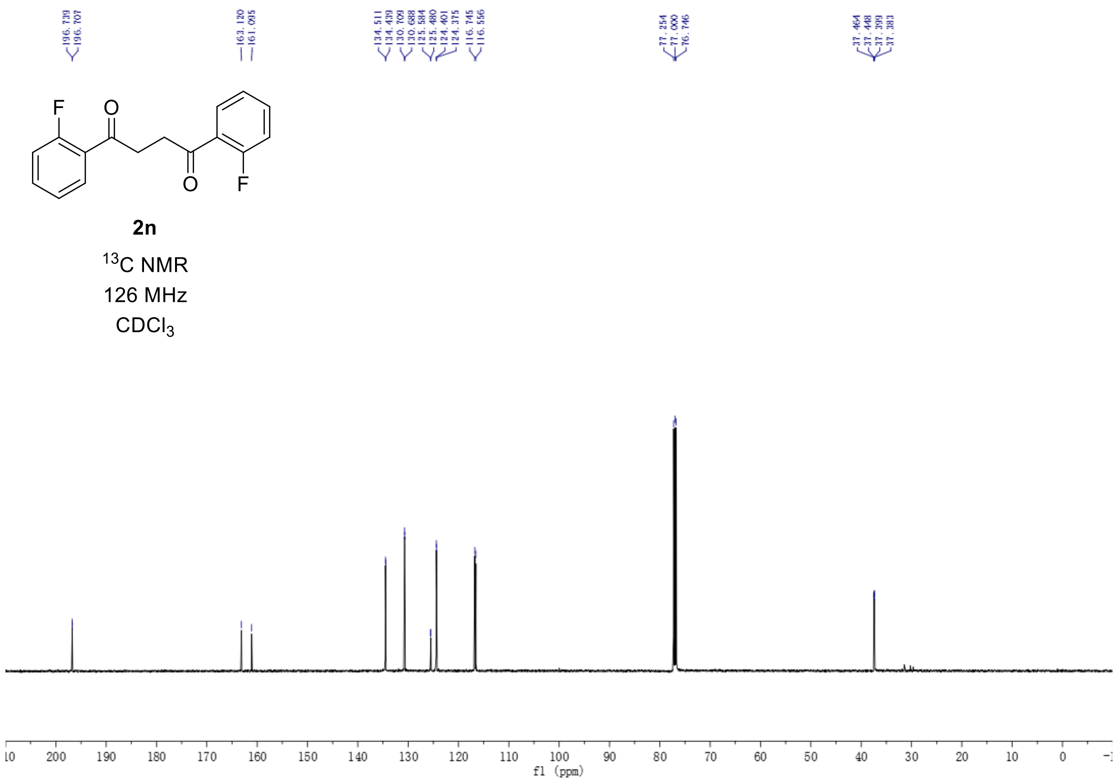

Supplementary Fig. 43 ^{13}C NMR spectrum of compound **2n**

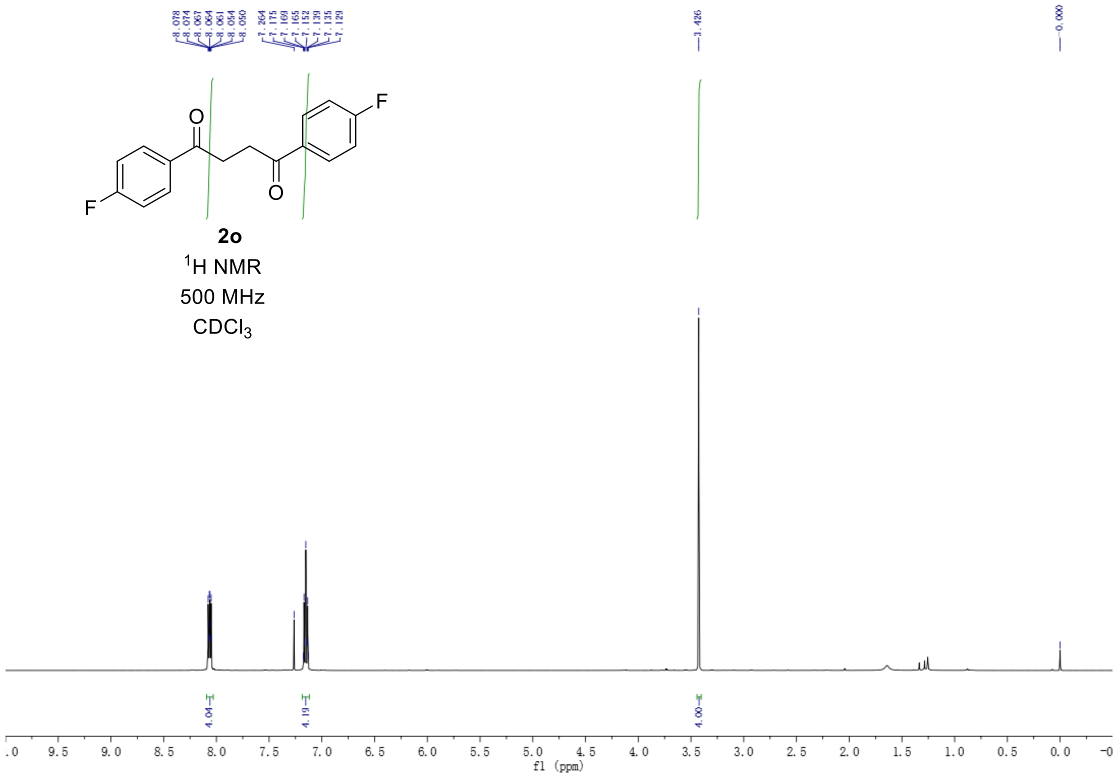

Supplementary Fig. 44 ^{1}H NMR spectrum of compound **2o**

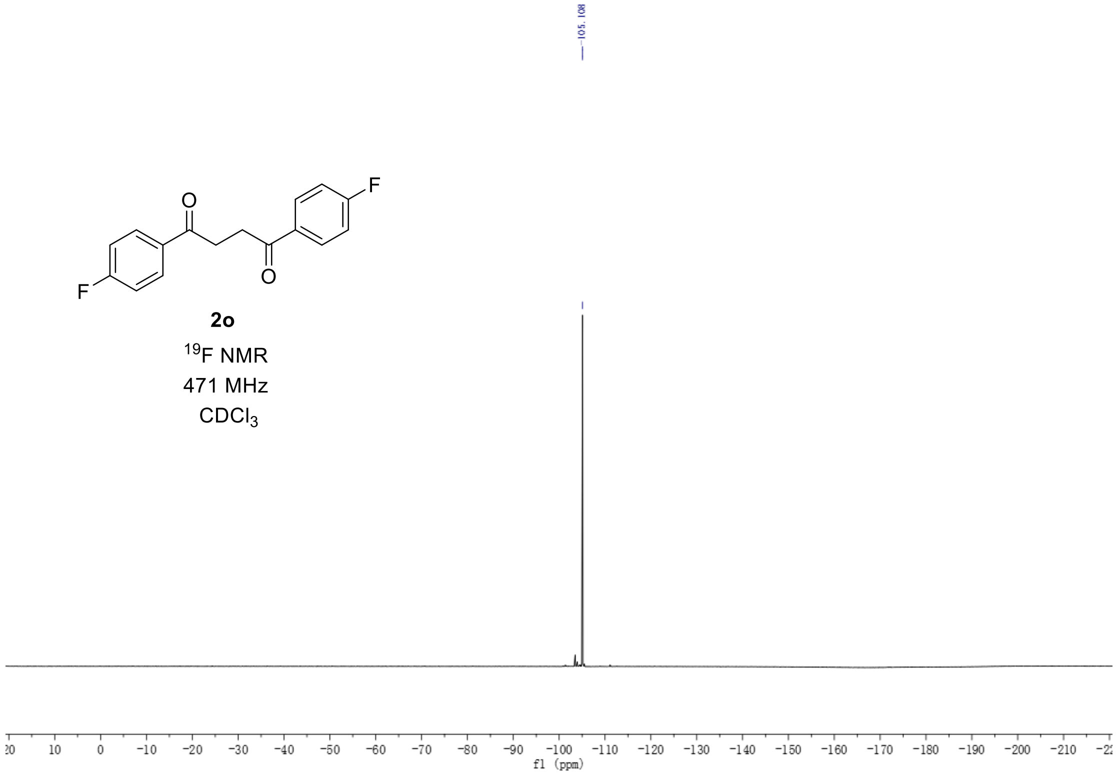

Supplementary Fig. 45 ^{19}F NMR spectrum of compound **2o**

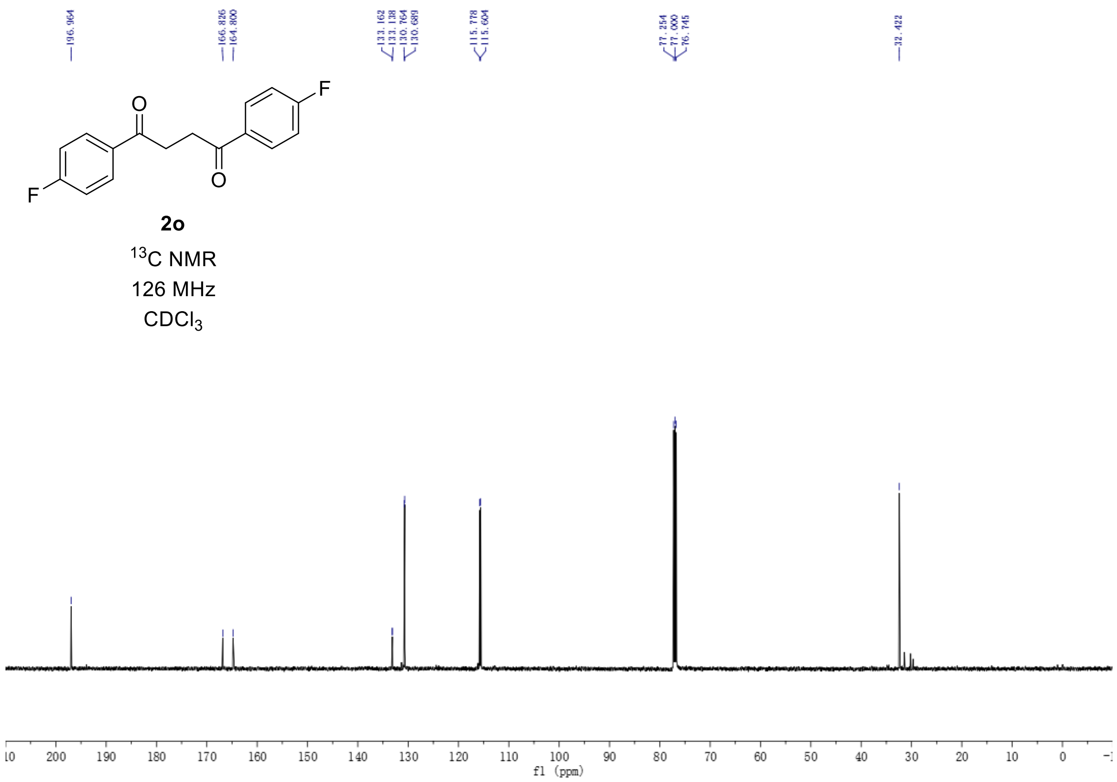

Supplementary Fig. 46 ^{13}C NMR spectrum of compound **2o**

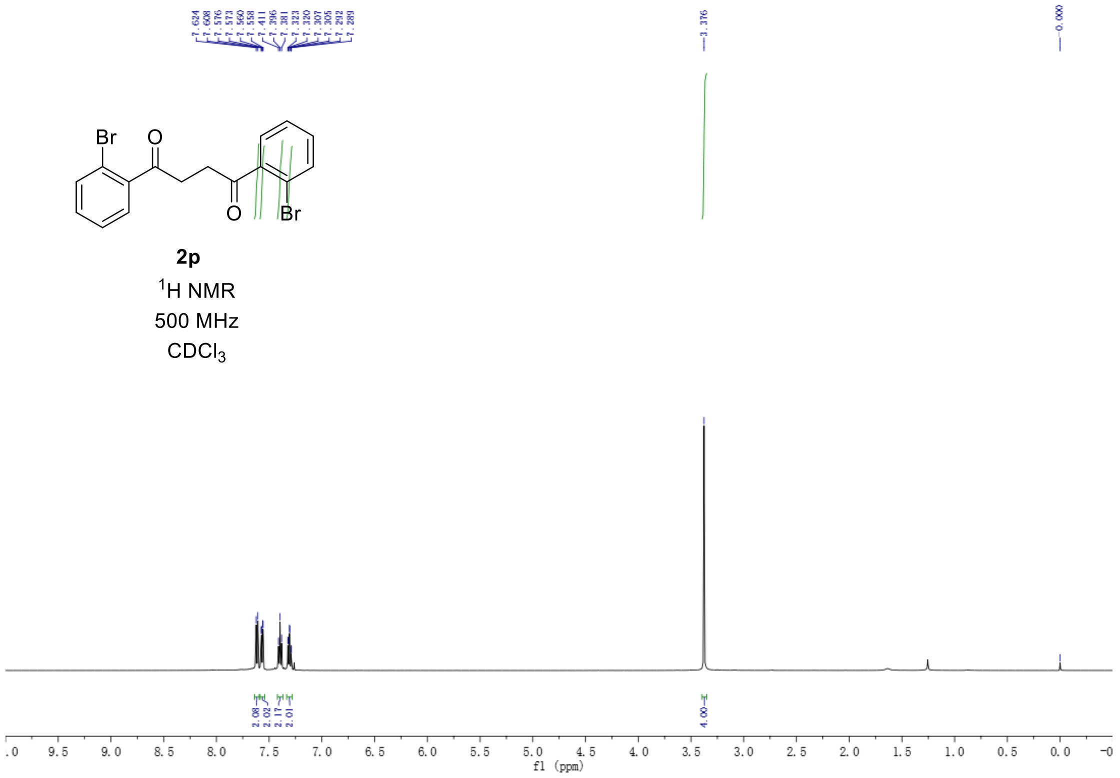

Supplementary Fig. 47 ^{1}H NMR spectrum of compound **2p**

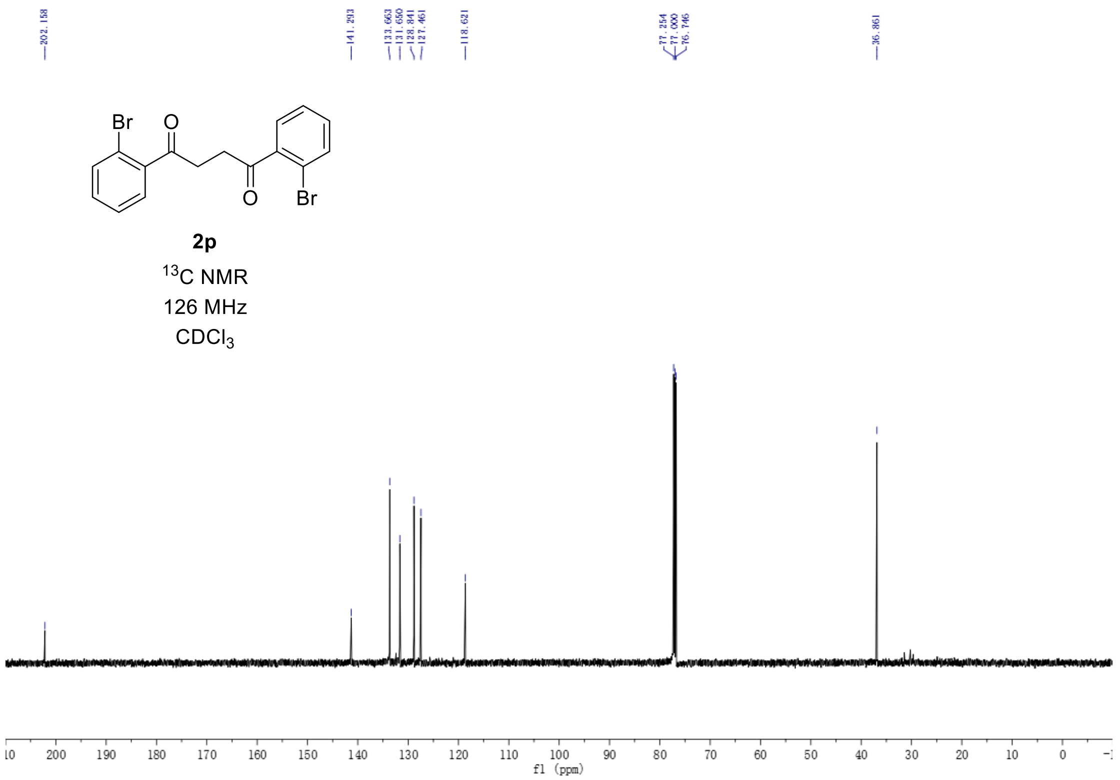

Supplementary Fig. 48 ^{13}C NMR spectrum of compound **2p**

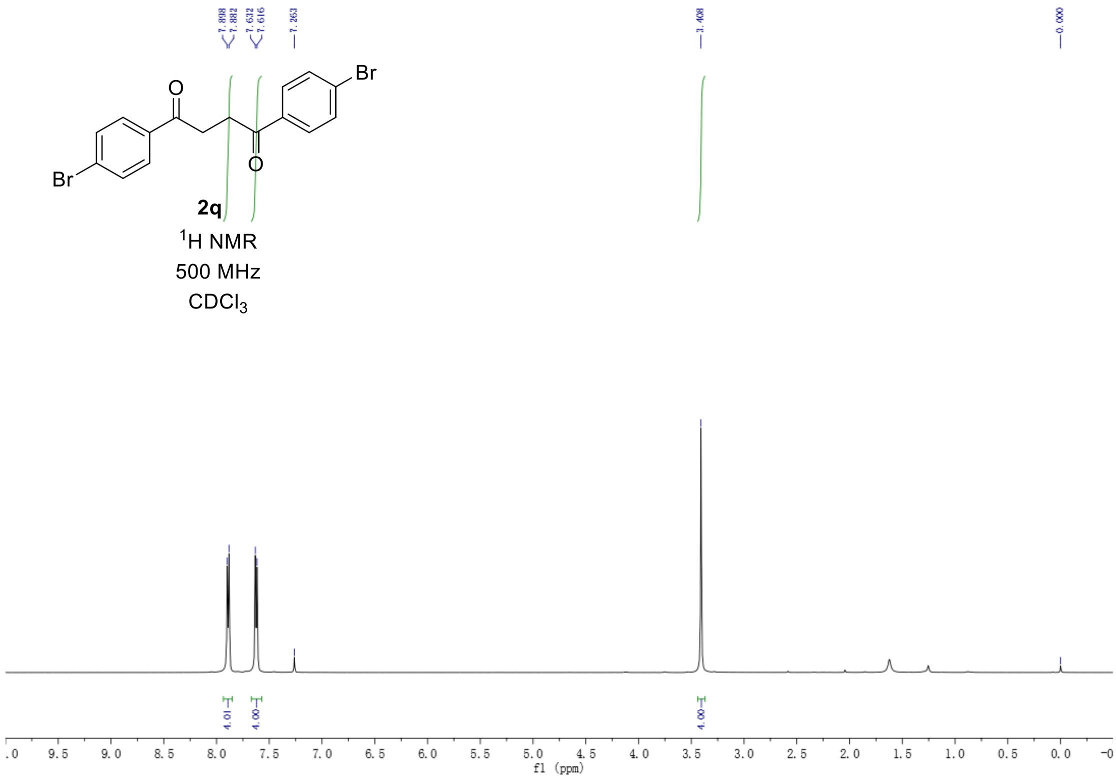

Supplementary Fig. 49 ^{1}H NMR spectrum of compound **2q**

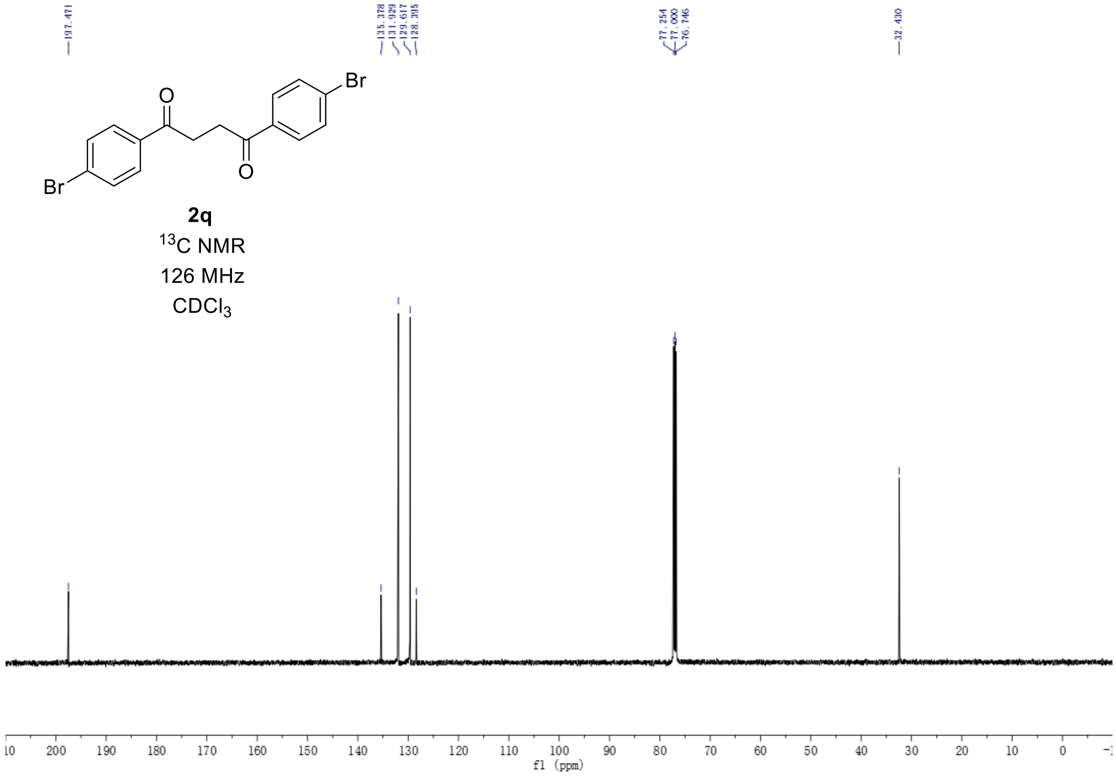

Supplementary Fig. 50 ^{13}C NMR spectrum of compound **2q**

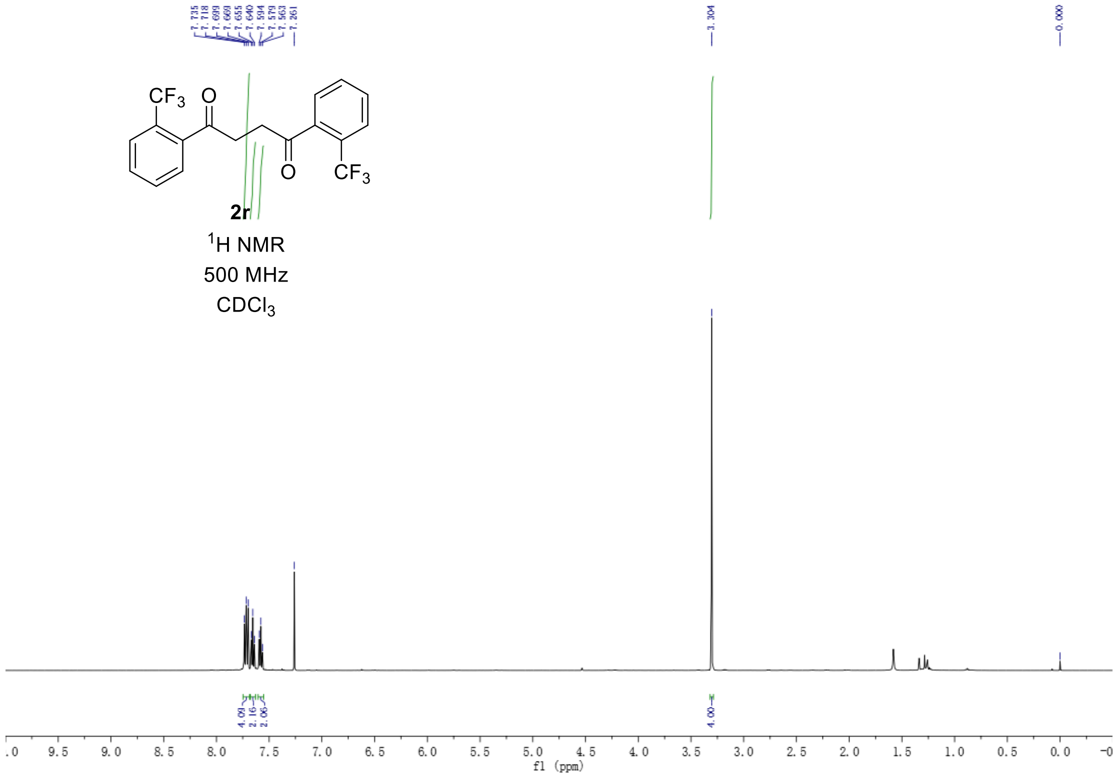

Supplementary Fig. 51 ^{1}H NMR spectrum of compound **2r**

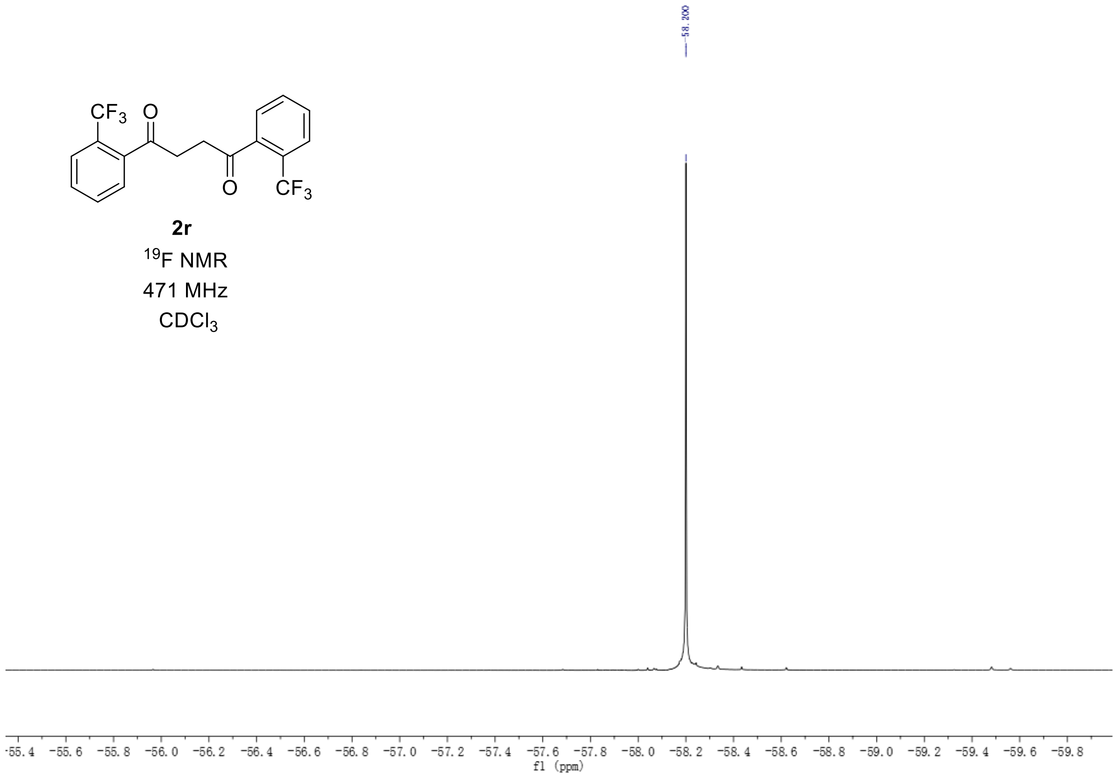

Supplementary Fig. 52 ^{19}F NMR spectrum of compound **2r**

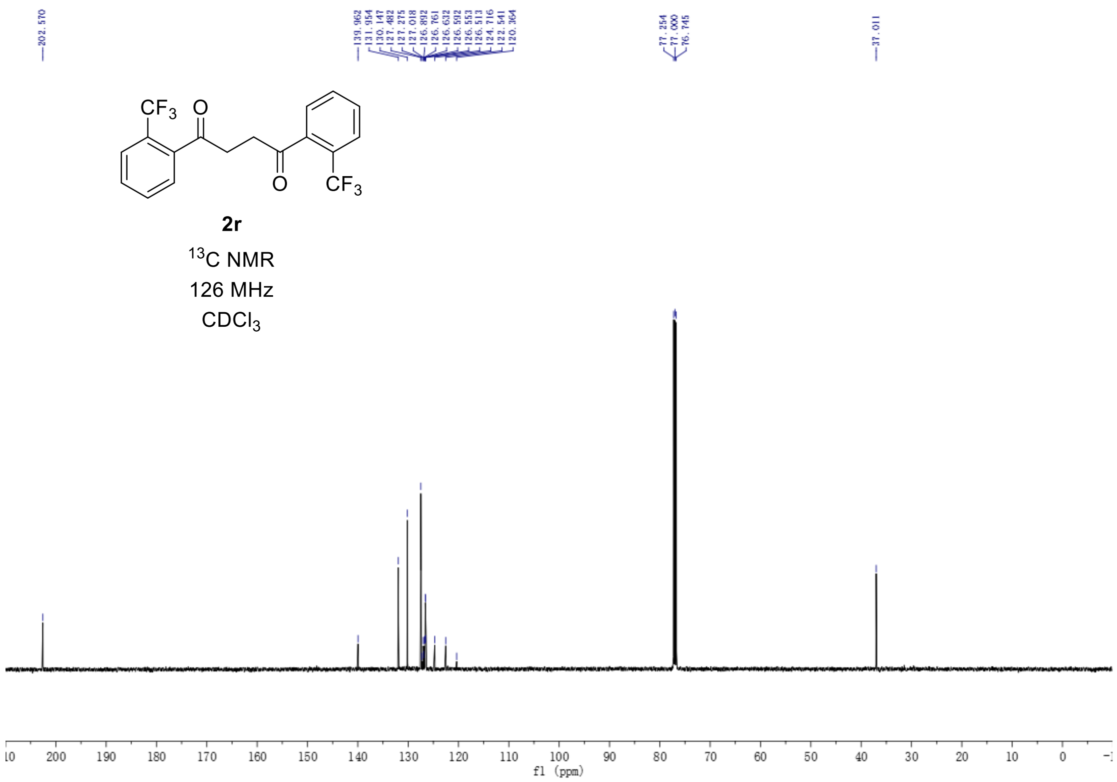

Supplementary Fig. 53 ^{13}C NMR spectrum of compound **2r**

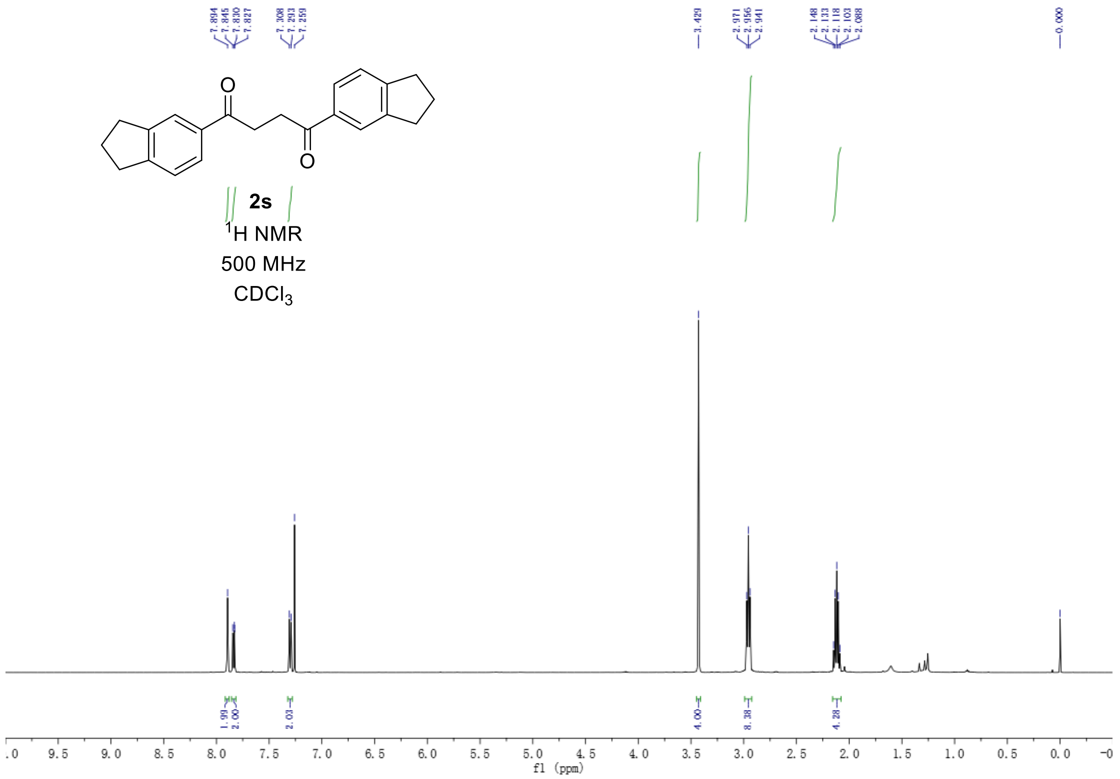

Supplementary Fig. 54 ^{1}H NMR spectrum of compound **2s**

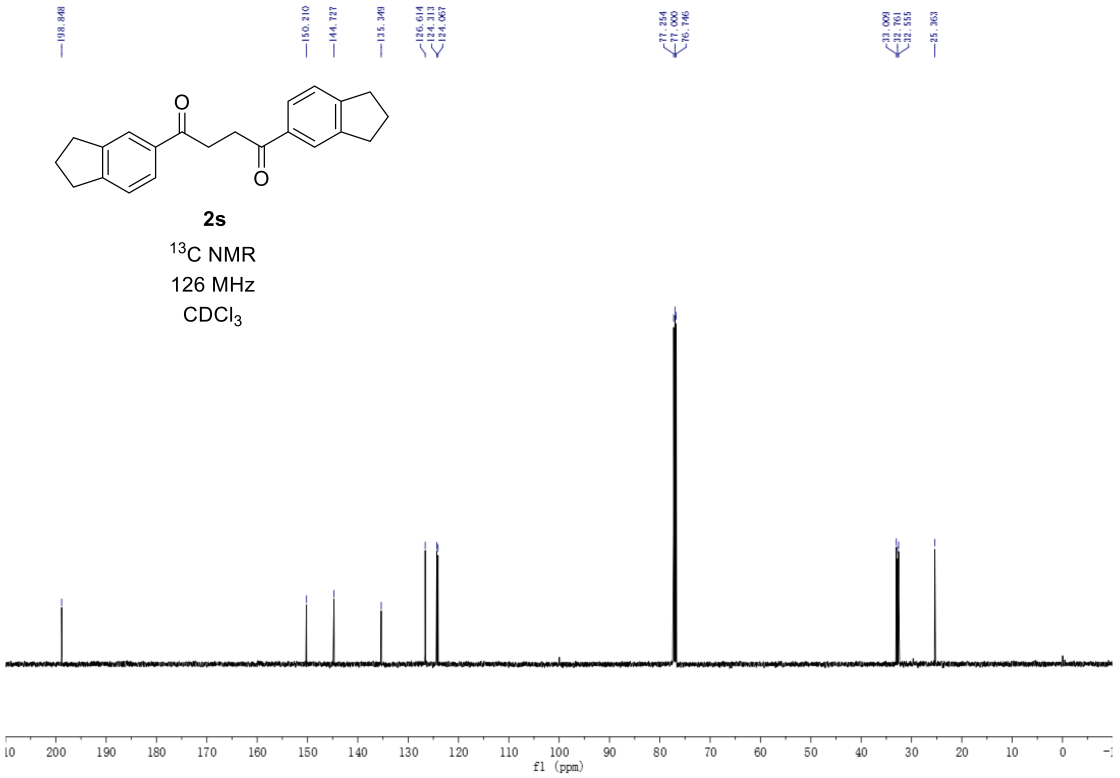

Supplementary Fig. 55 ^{13}C NMR spectrum of compound **2s**

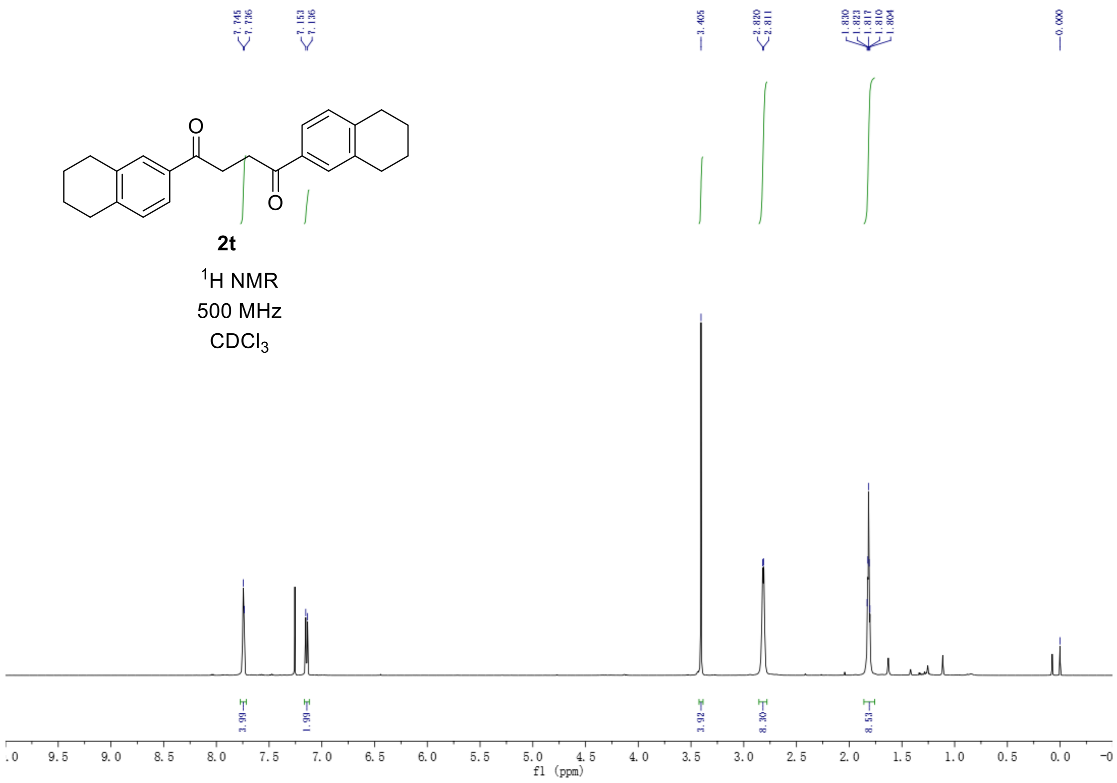

Supplementary Fig. 56 ^{1}H NMR spectrum of compound **2t**

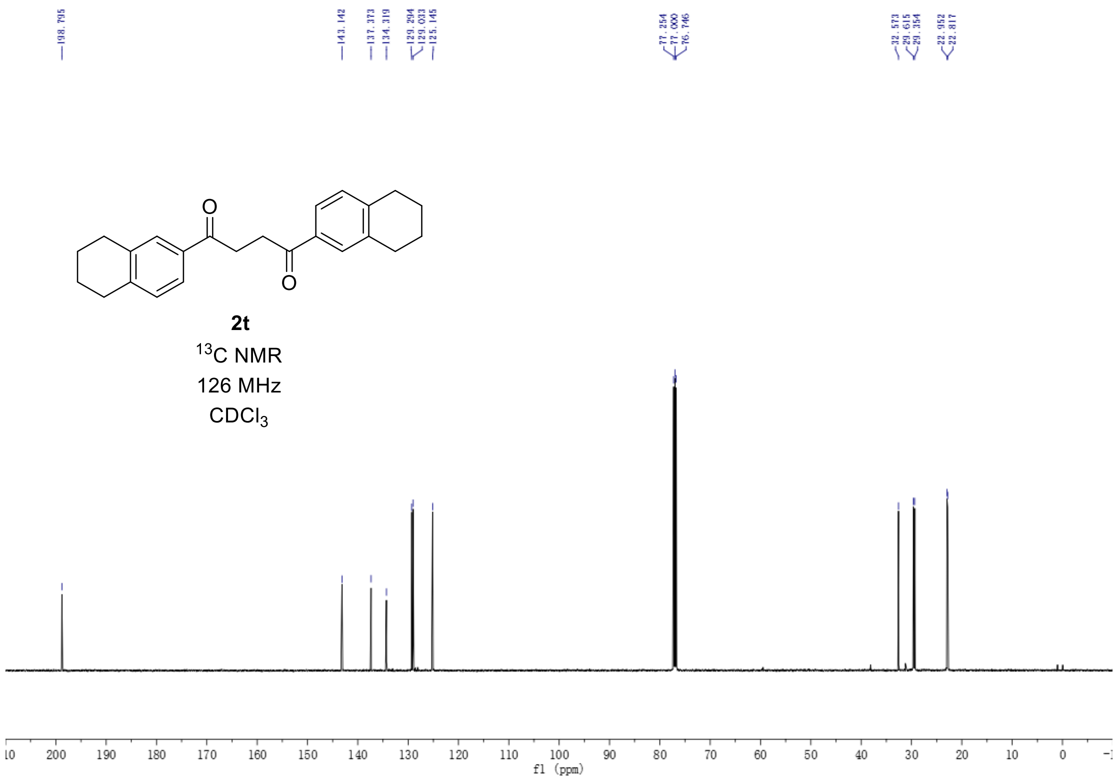

Supplementary Fig. 57 ^{13}C NMR spectrum of compound **2t**

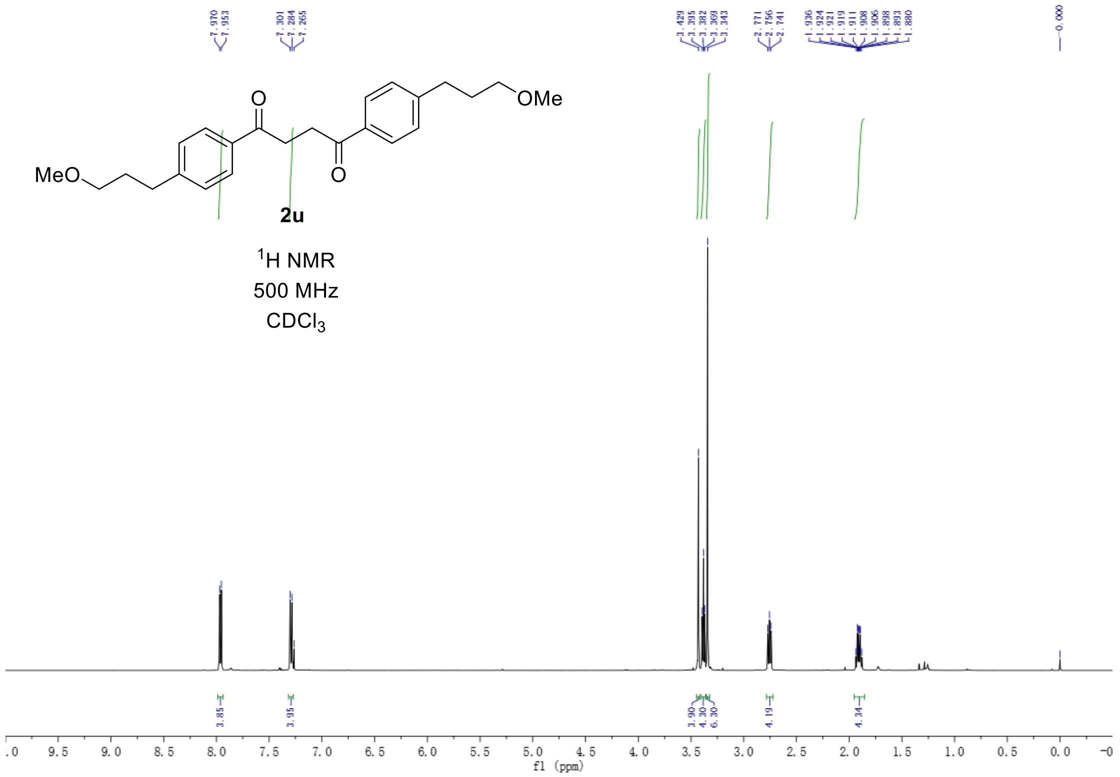

Supplementary Fig. 58 ^{1}H NMR spectrum of compound **2u**

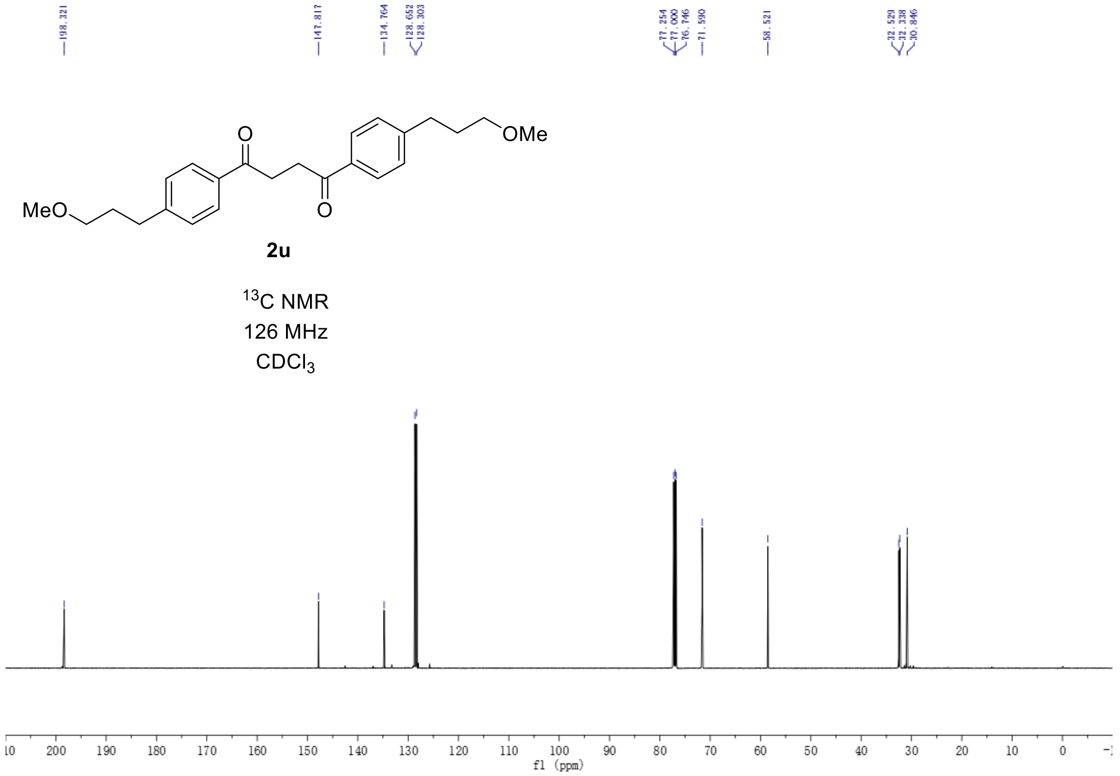

Supplementary Fig. 59 ^{13}C NMR spectrum of compound **2u**

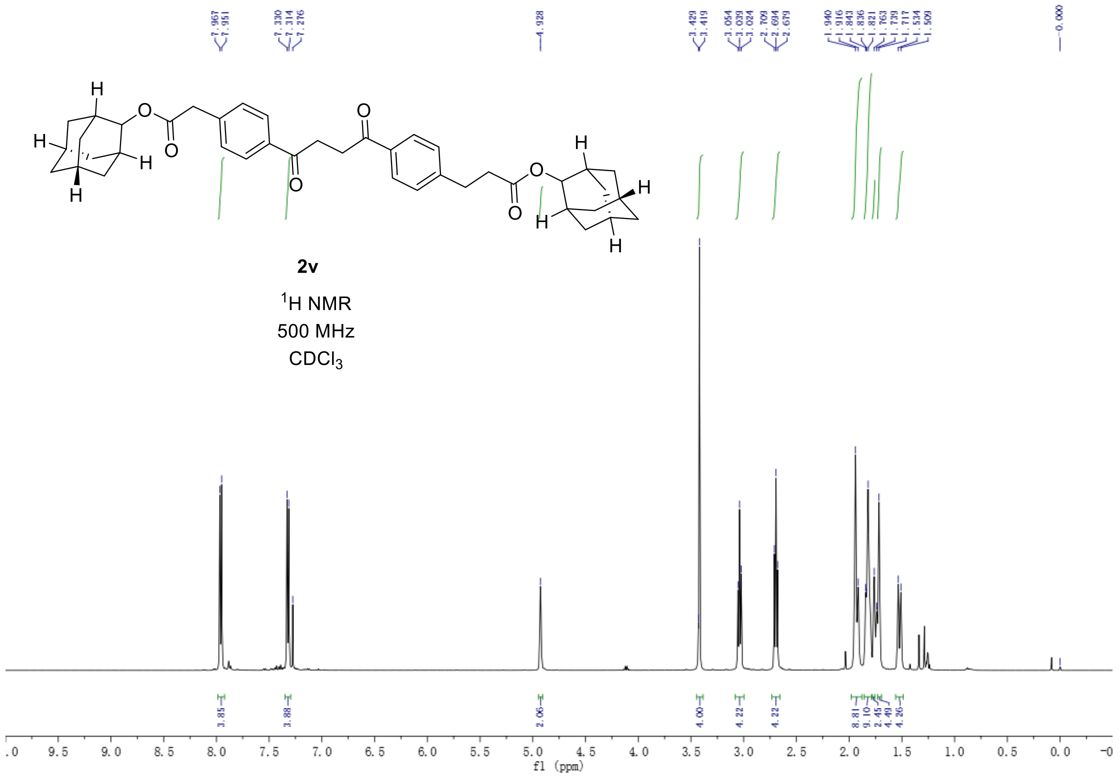

Supplementary Fig. 60 ^{1}H NMR spectrum of compound **2v**

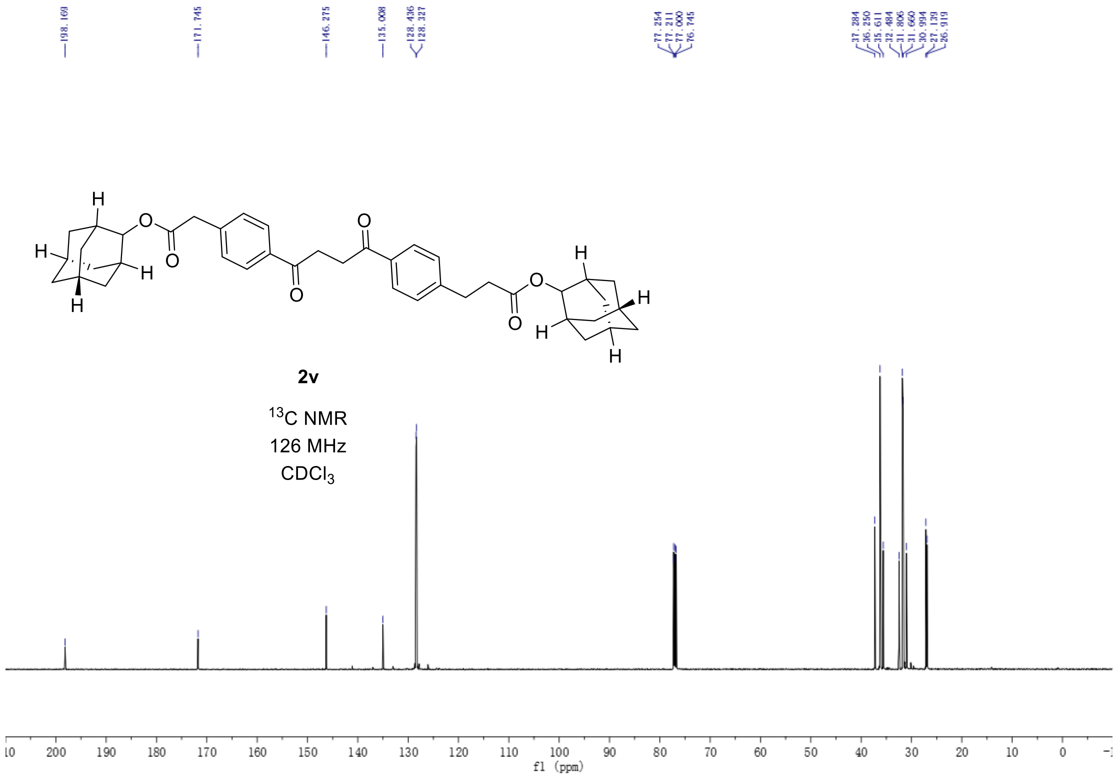

Supplementary Fig. 61 ^{13}C NMR spectrum of compound **2v**

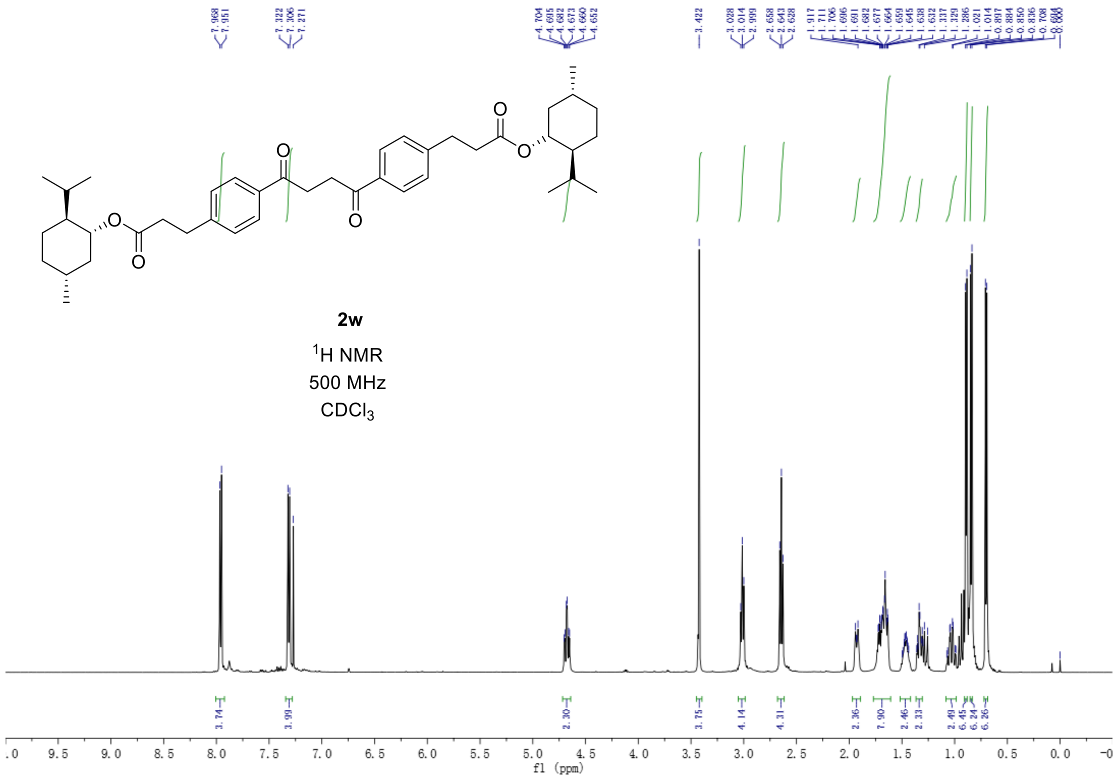

Supplementary Fig. 62 ^{1}H NMR spectrum of compound **2w**

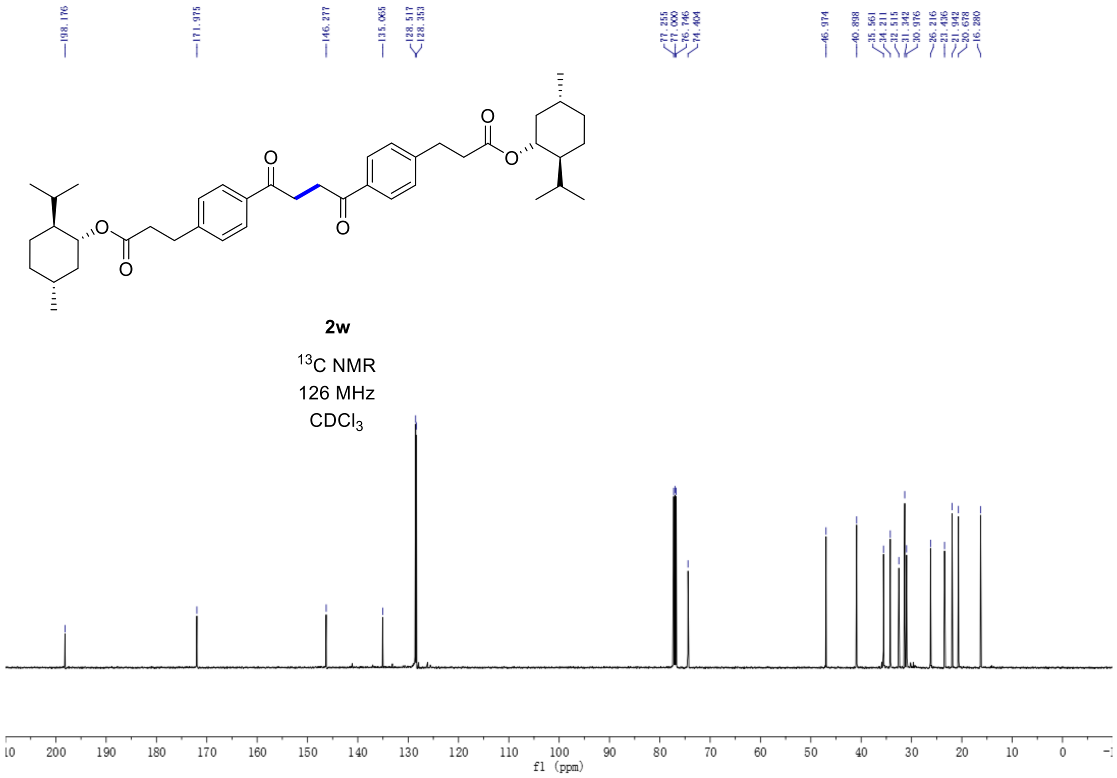

Supplementary Fig. 63 ^{13}C NMR spectrum of compound **2w**

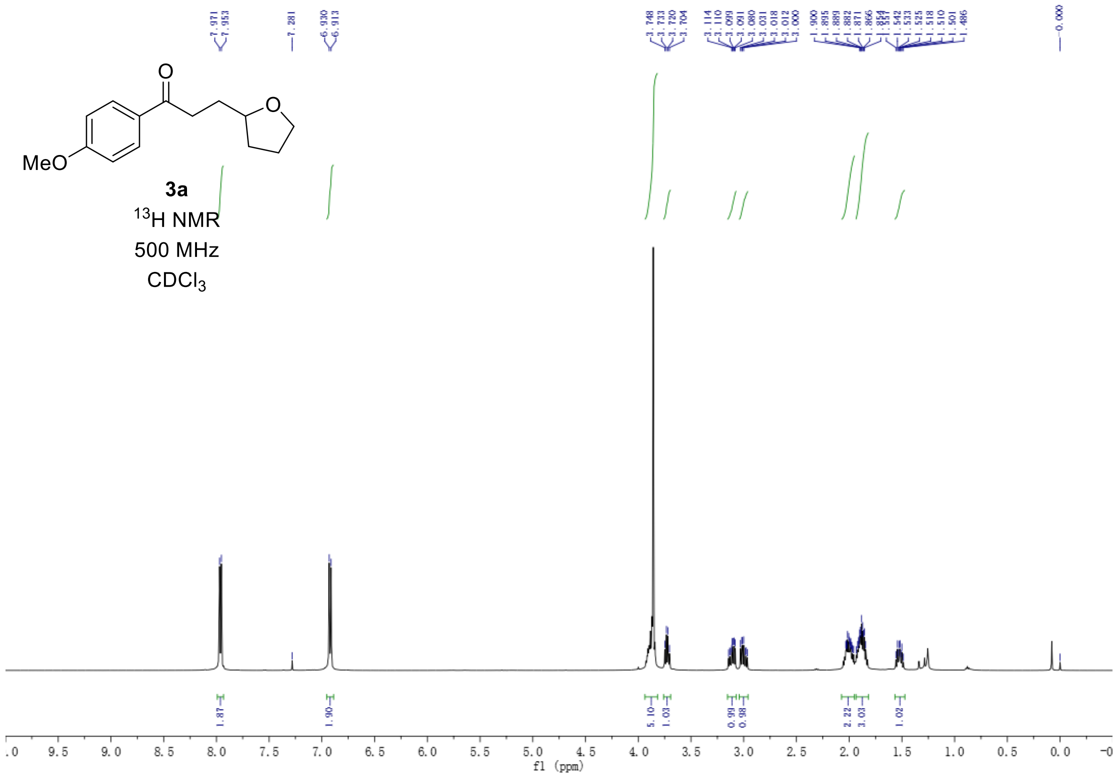

Supplementary Fig. 64 ^{1}H NMR spectrum of compound **3a**

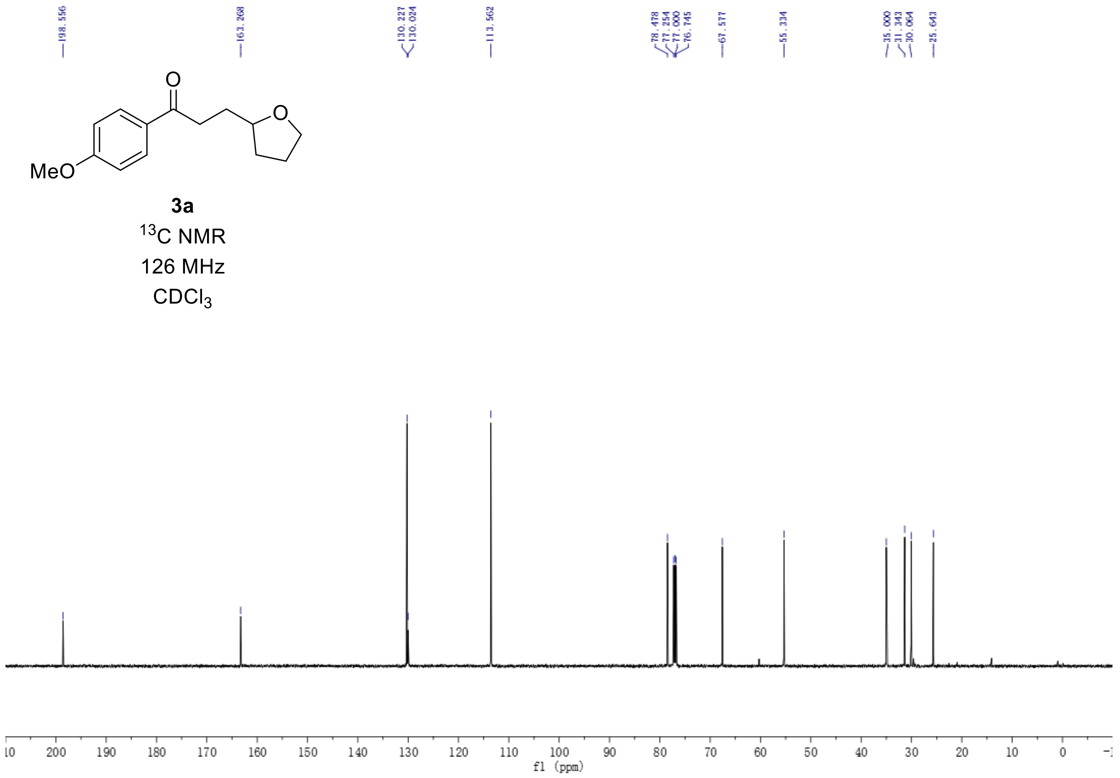

Supplementary Fig. 65 ^{13}C NMR spectrum of compound **3a**

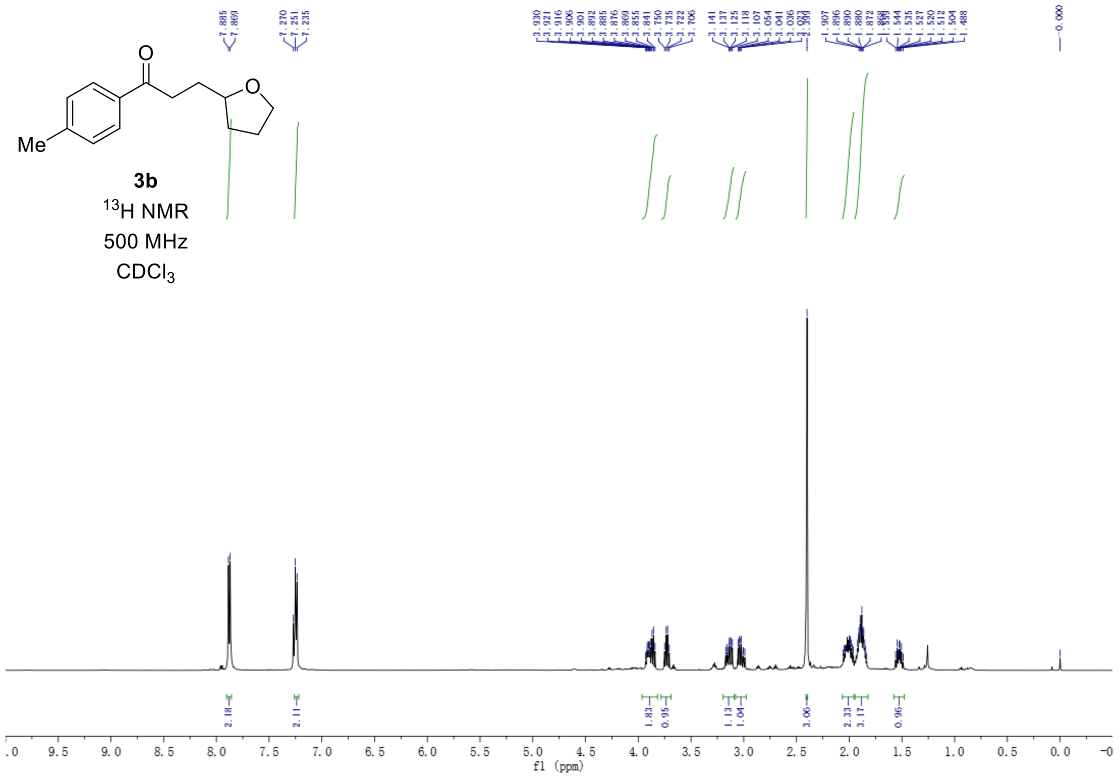

Supplementary Fig. 66 ^{1}H NMR spectrum of compound **3b**

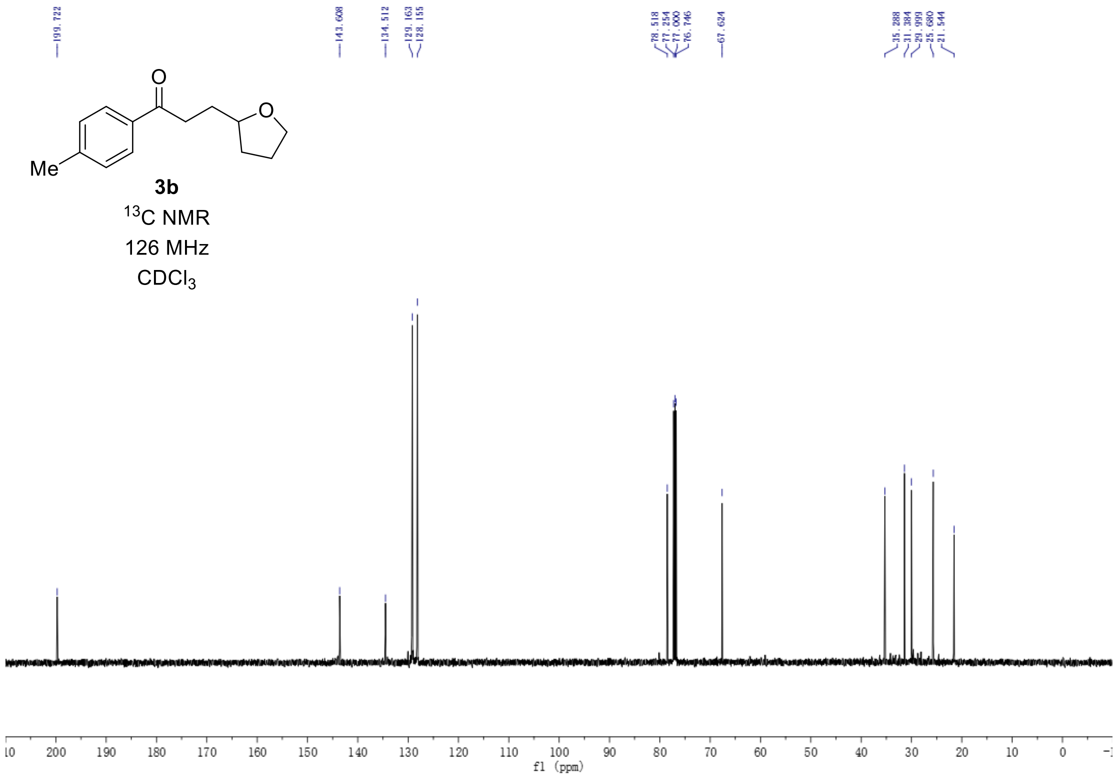

Supplementary Fig. 67 ^{13}C NMR spectrum of compound **3b**

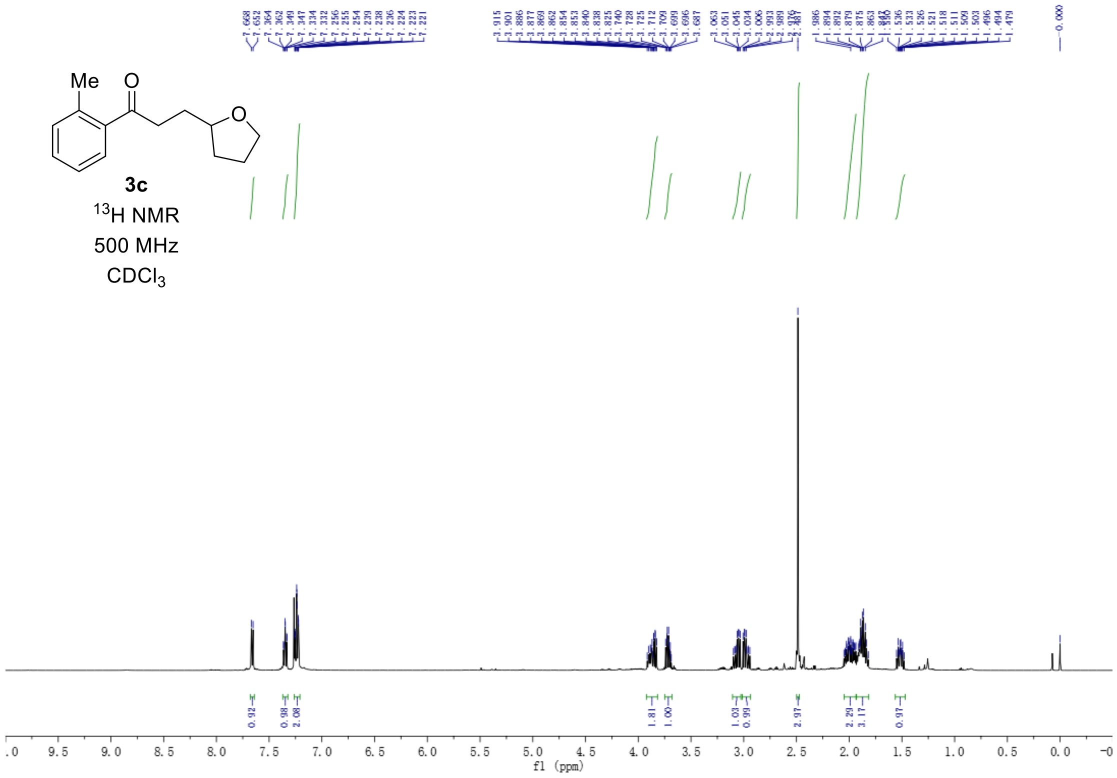

Supplementary Fig. 68 ^{1}H NMR spectrum of compound **3c**

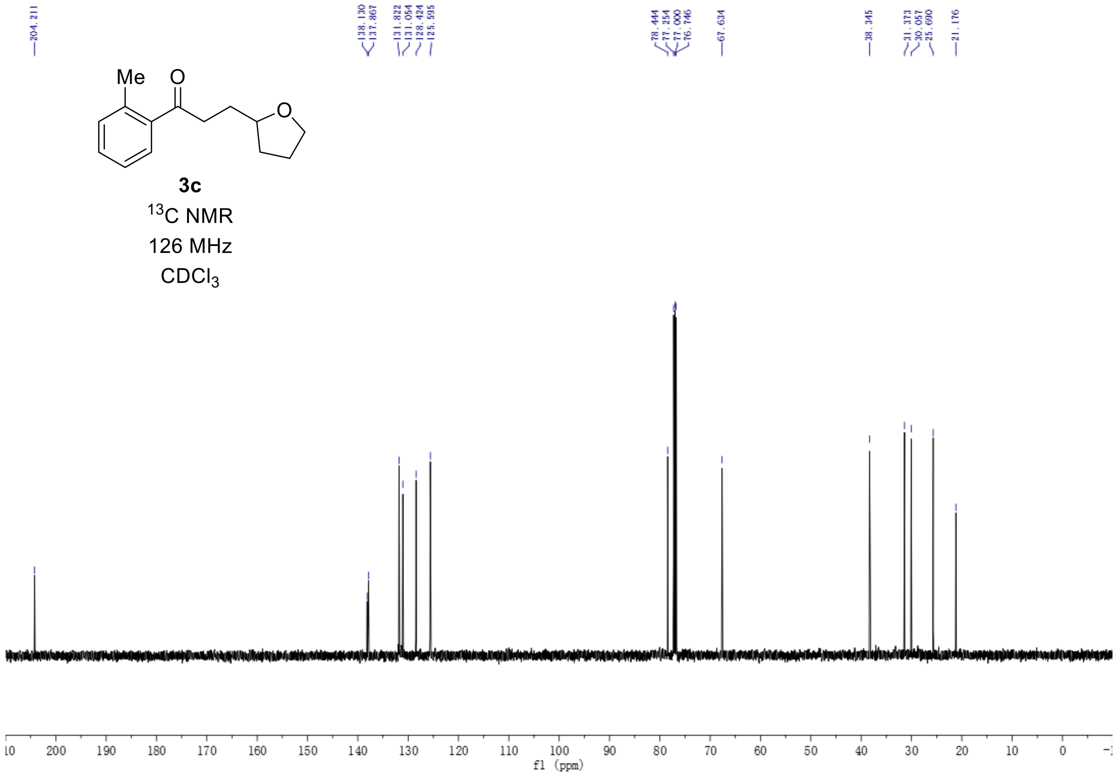

Supplementary Fig. 69 ^{13}C NMR spectrum of compound **3c**

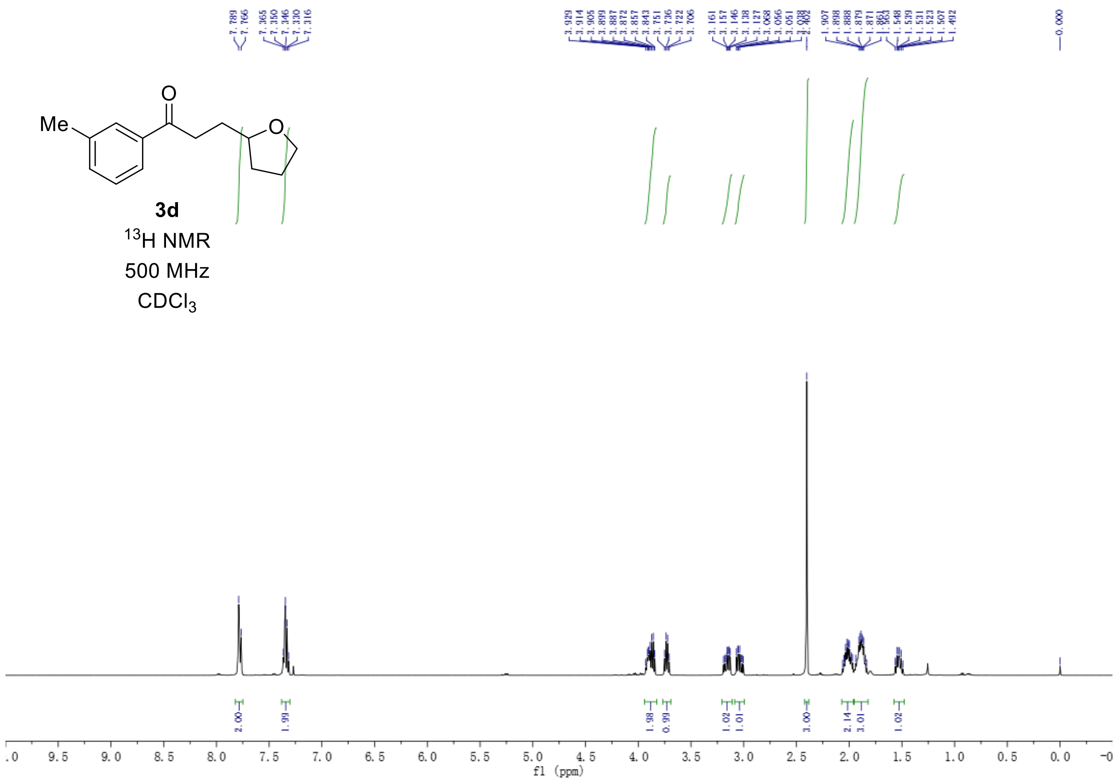

Supplementary Fig. 70 ^{1}H NMR spectrum of compound **3d**

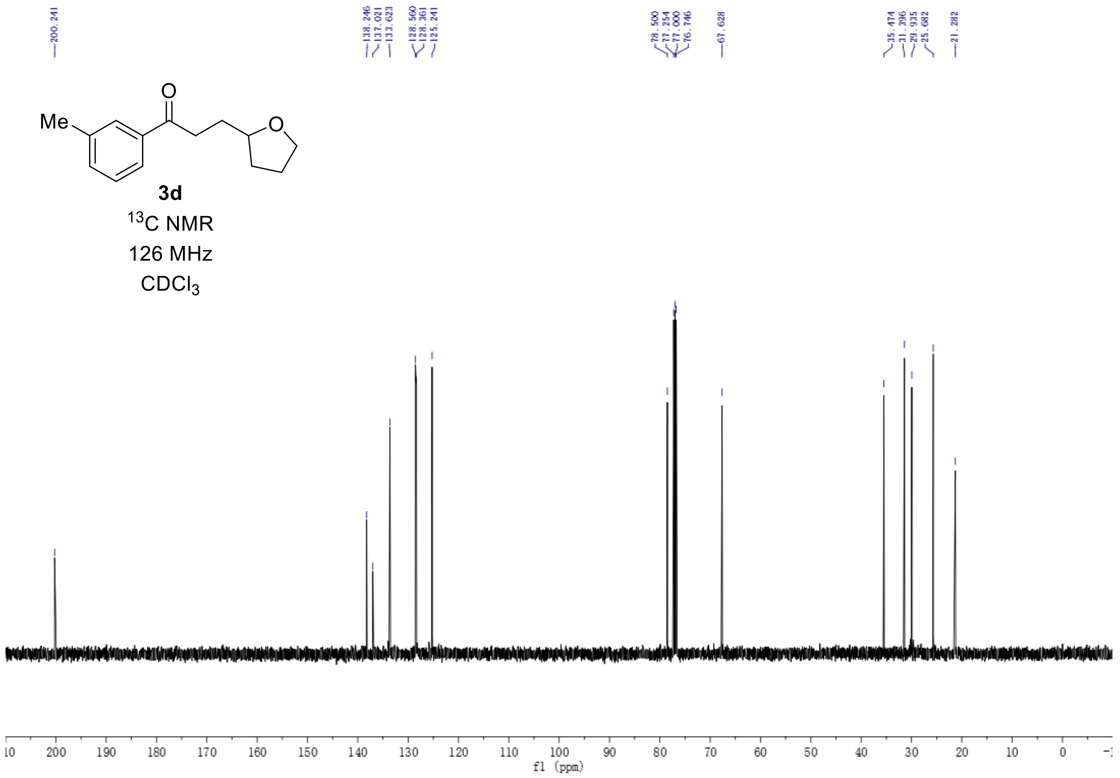

Supplementary Fig. 71 ^{13}C NMR spectrum of compound **3d**

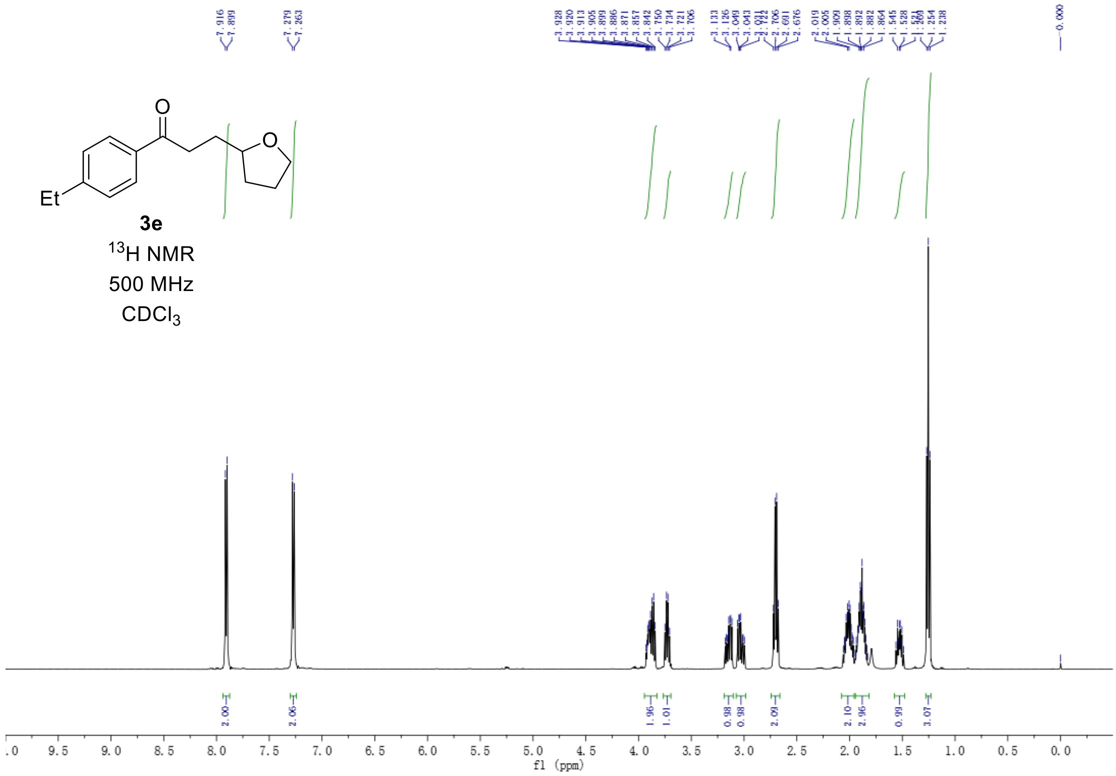

Supplementary Fig. 72 ^{1}H NMR spectrum of compound **3e**

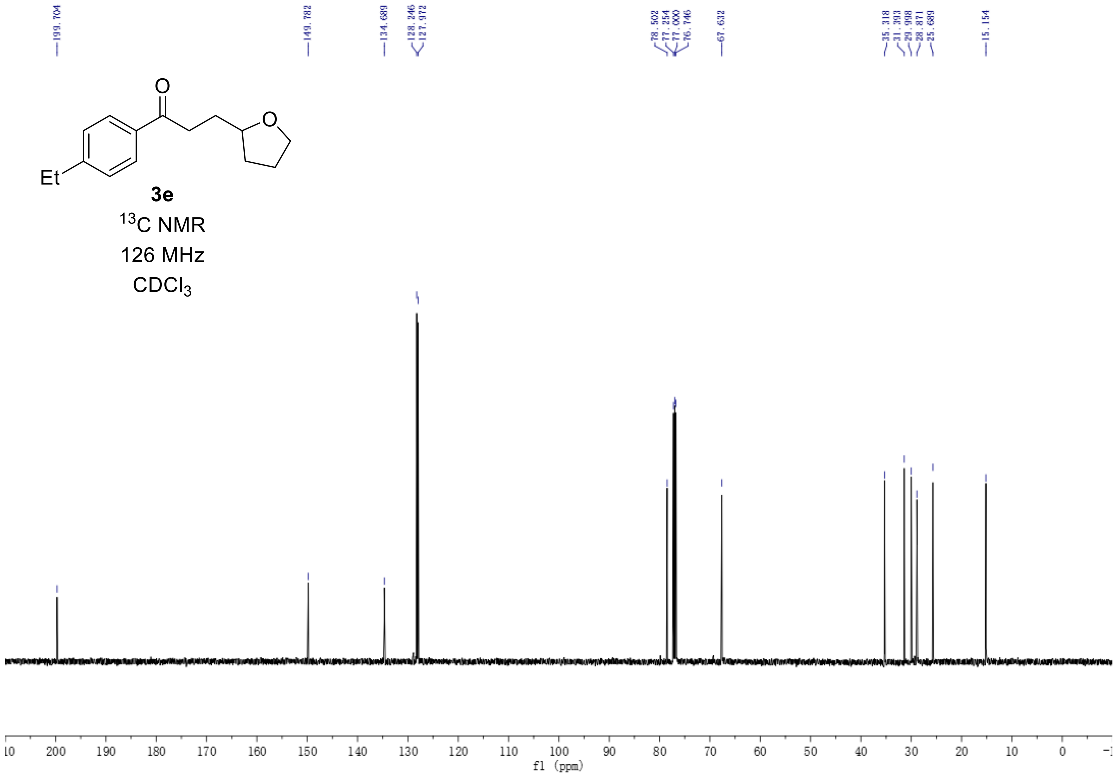

Supplementary Fig. 73 ^{13}C NMR spectrum of compound **3e**

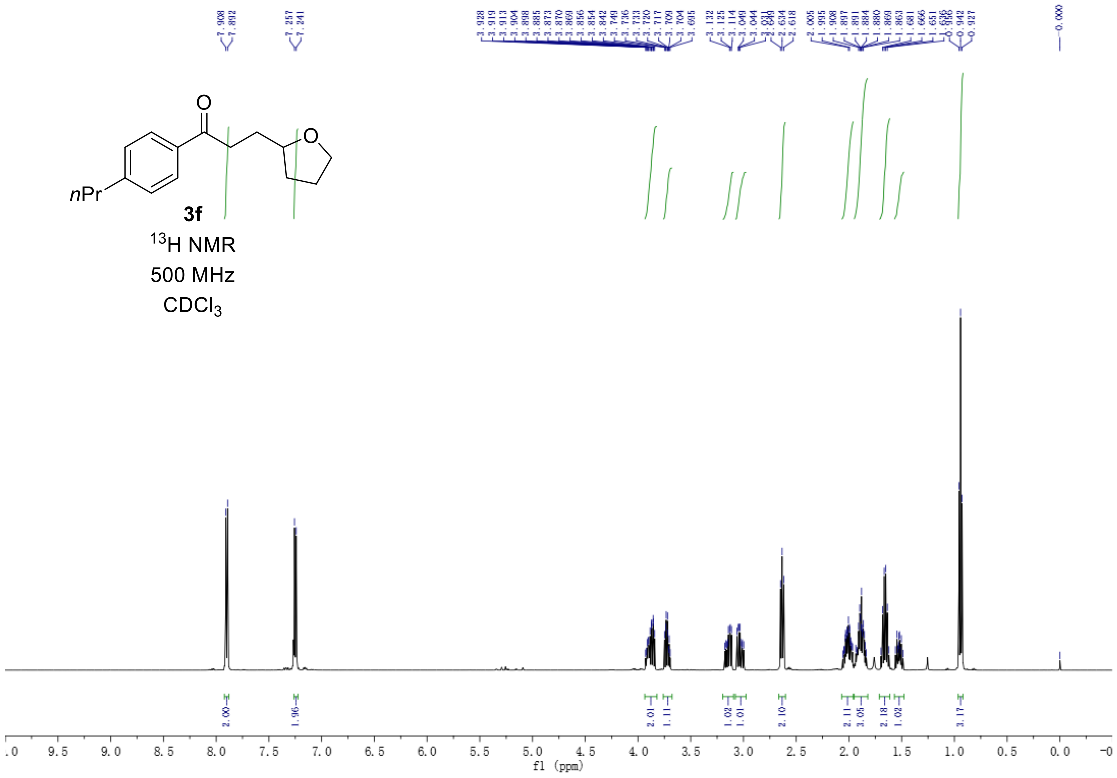

Supplementary Fig. 74 ^{1}H NMR spectrum of compound **3f**

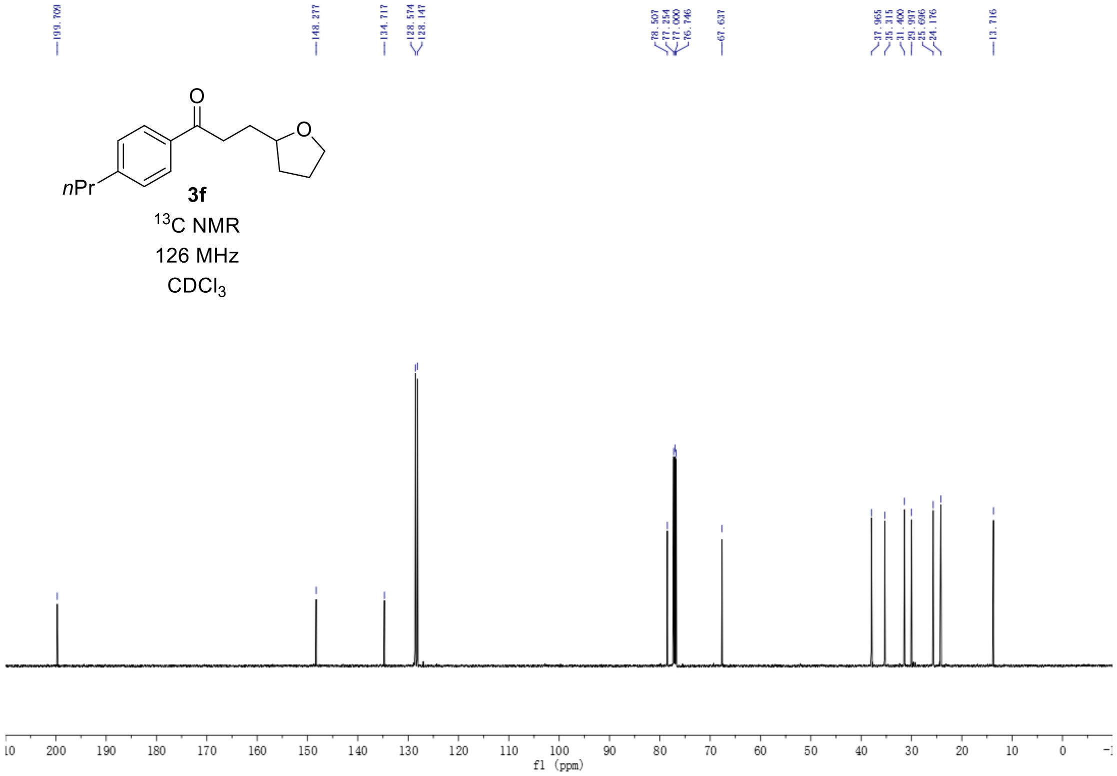

Supplementary Fig. 75 ^{13}C NMR spectrum of compound **3f**

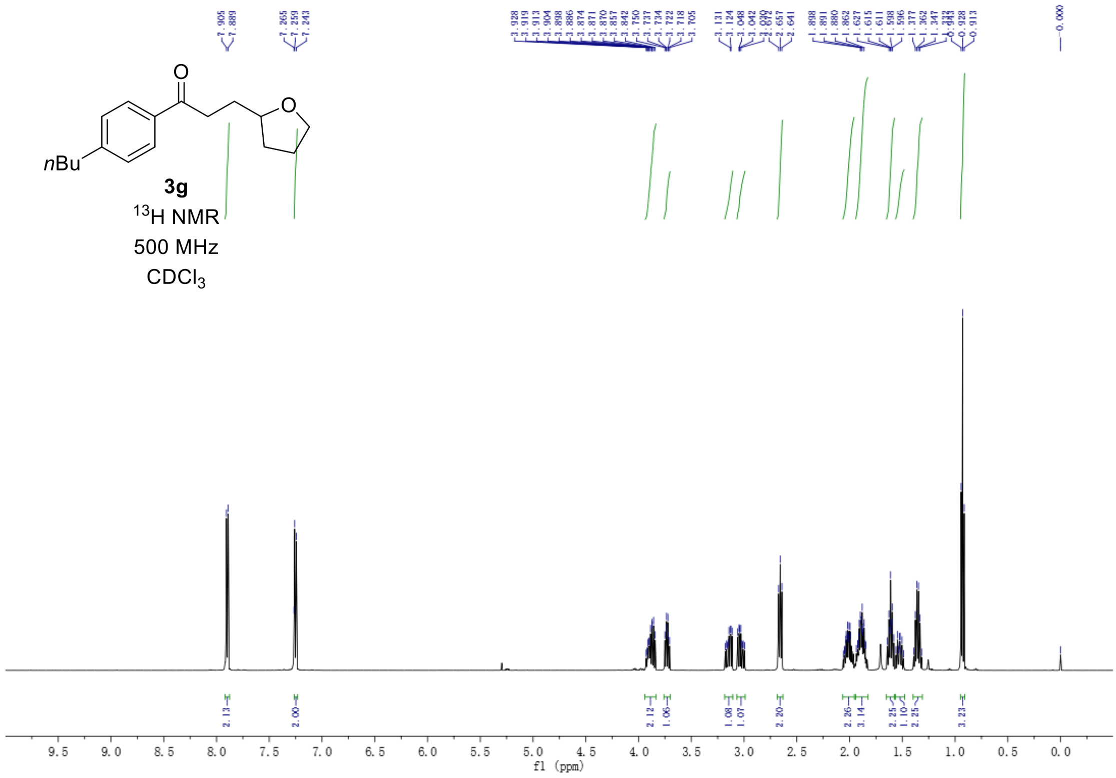

Supplementary Fig. 76 ^{1}H NMR spectrum of compound **3g**

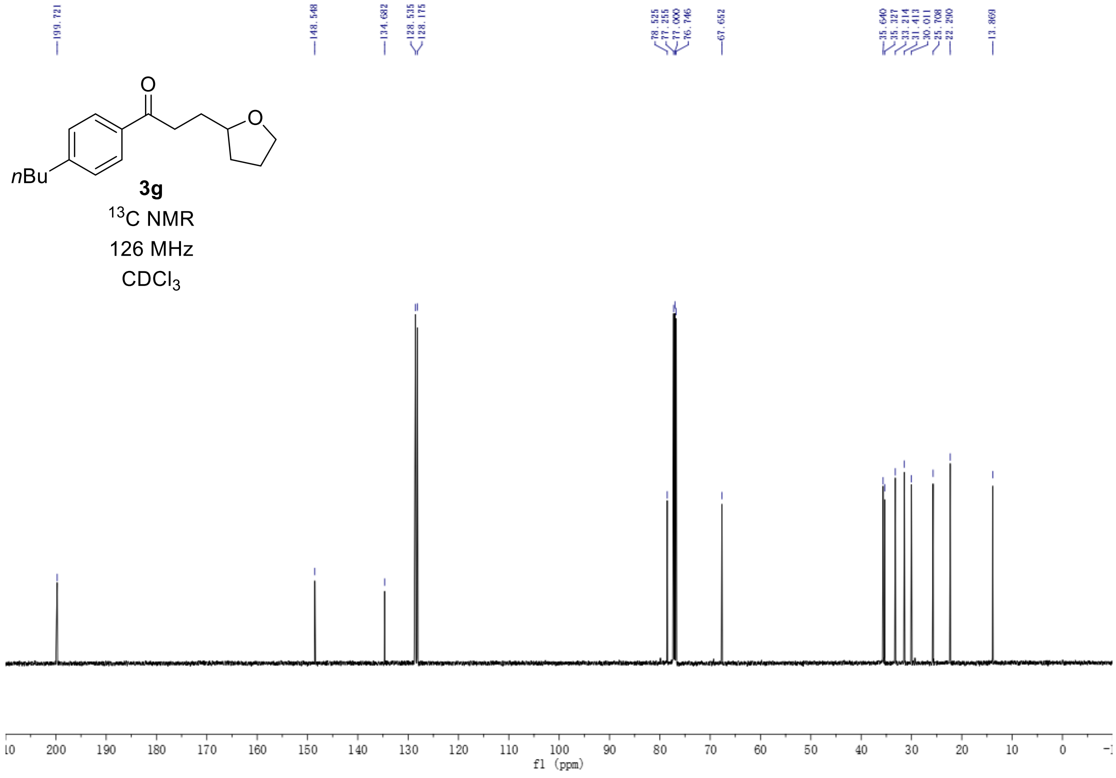

Supplementary Fig. 77 ^{13}C NMR spectrum of compound **3g**

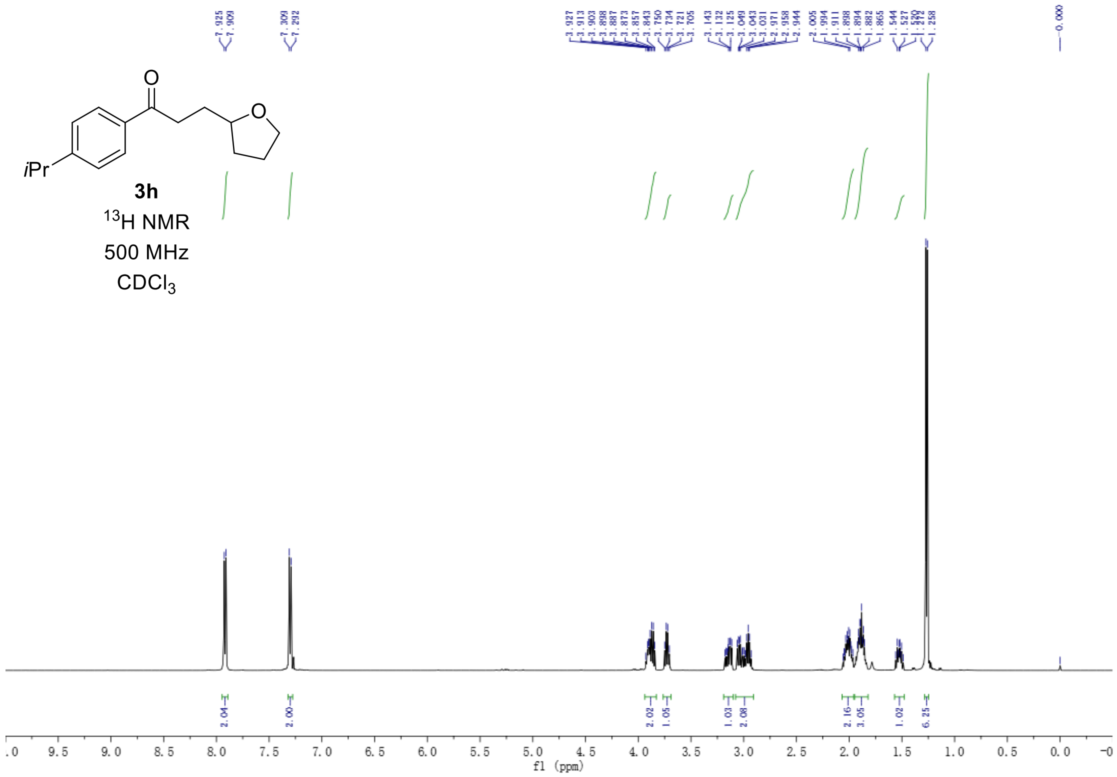

Supplementary Fig. 78 ^{1}H NMR spectrum of compound **3h**

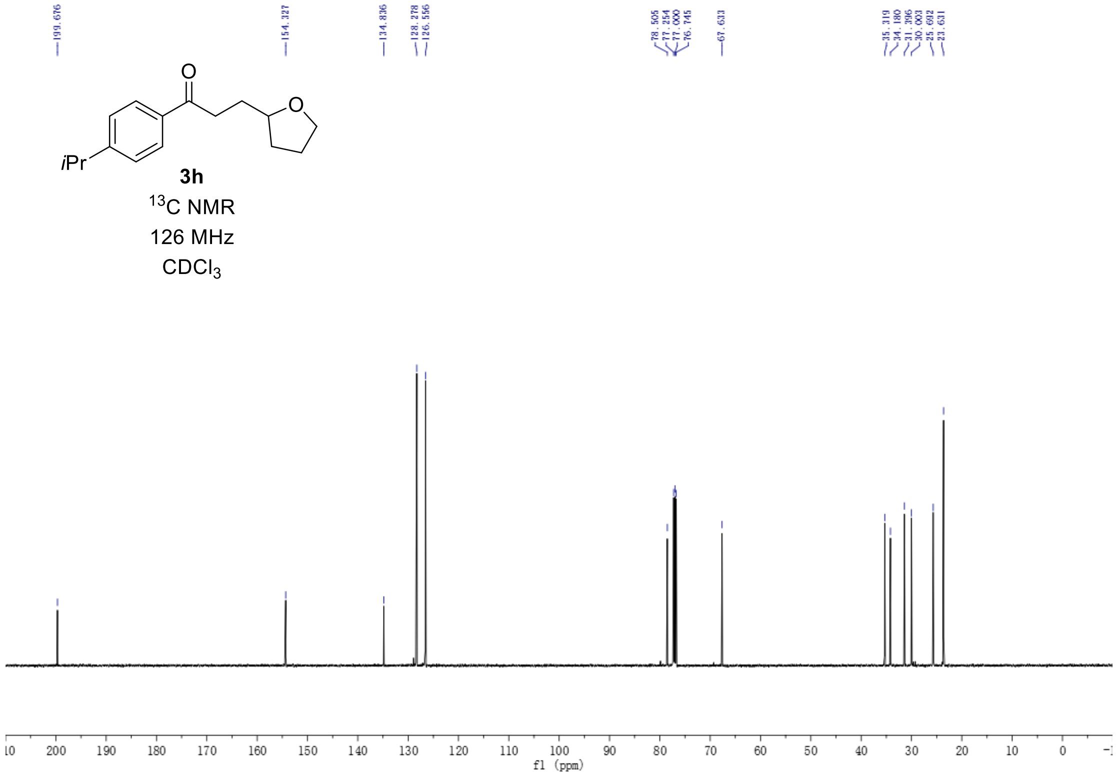

Supplementary Fig. 79 ^{13}C NMR spectrum of compound **3h**

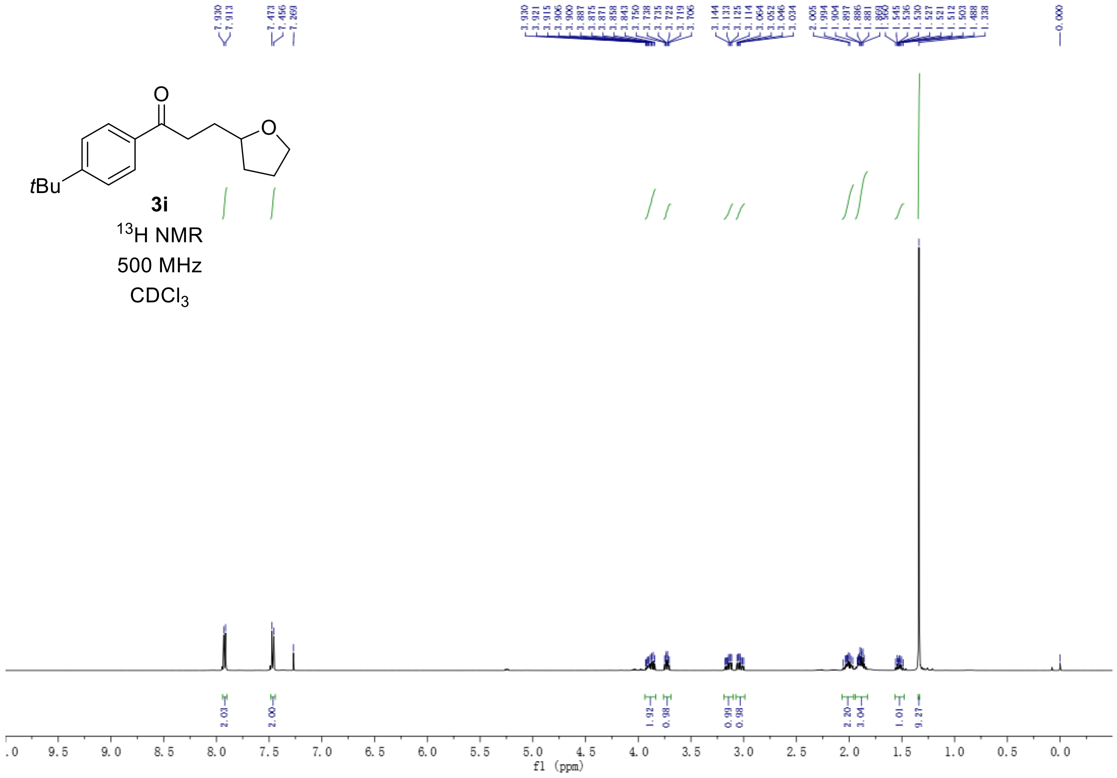

Supplementary Fig. 80 ^{1}H NMR spectrum of compound **3i**

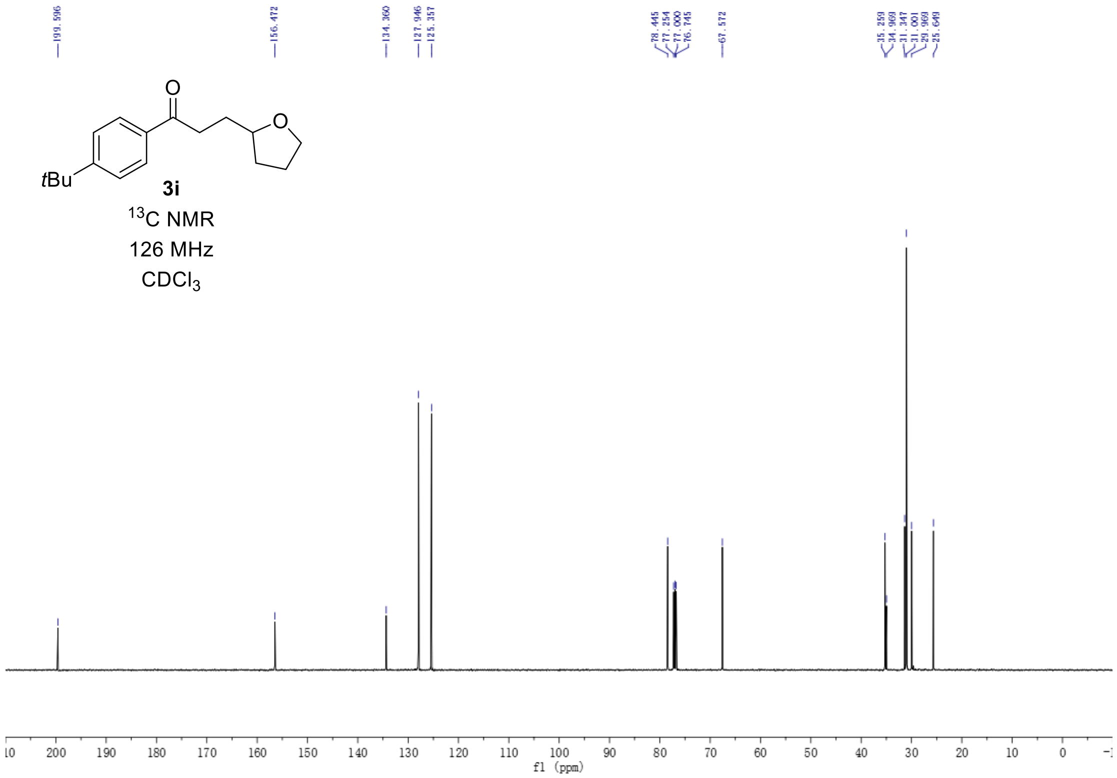

Supplementary Fig. 81 ^{13}C NMR spectrum of compound **3i**

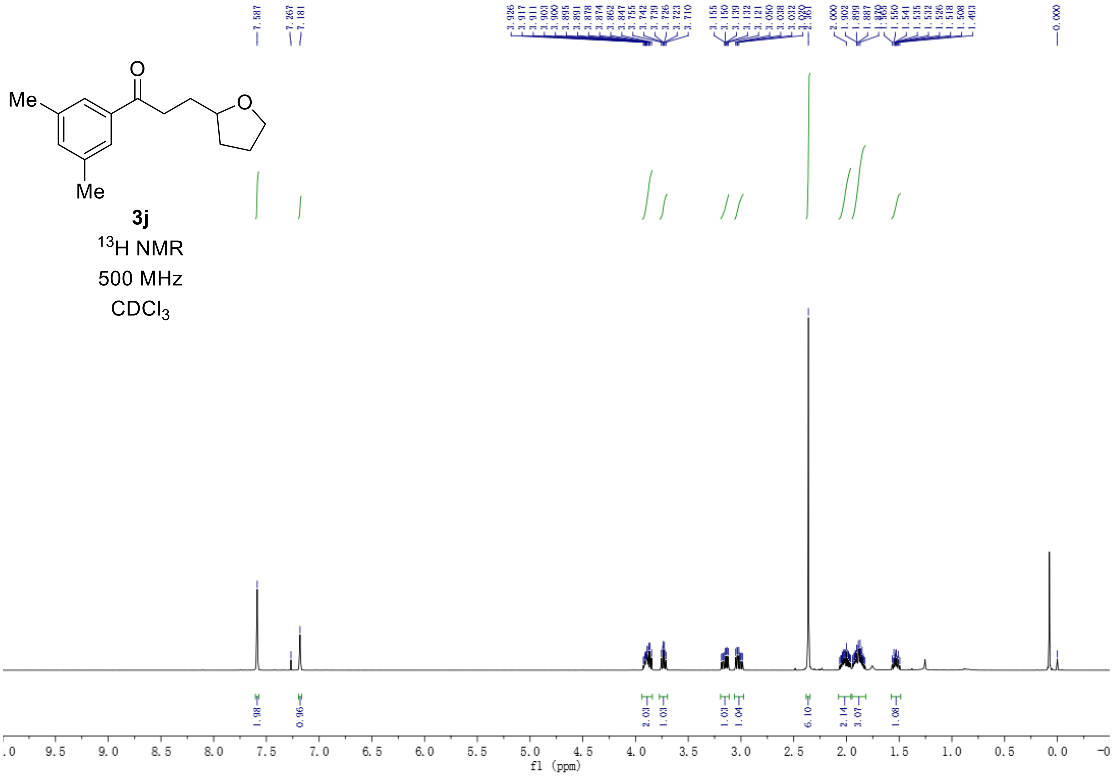

Supplementary Fig. 82 ^{1}H NMR spectrum of compound **3j**

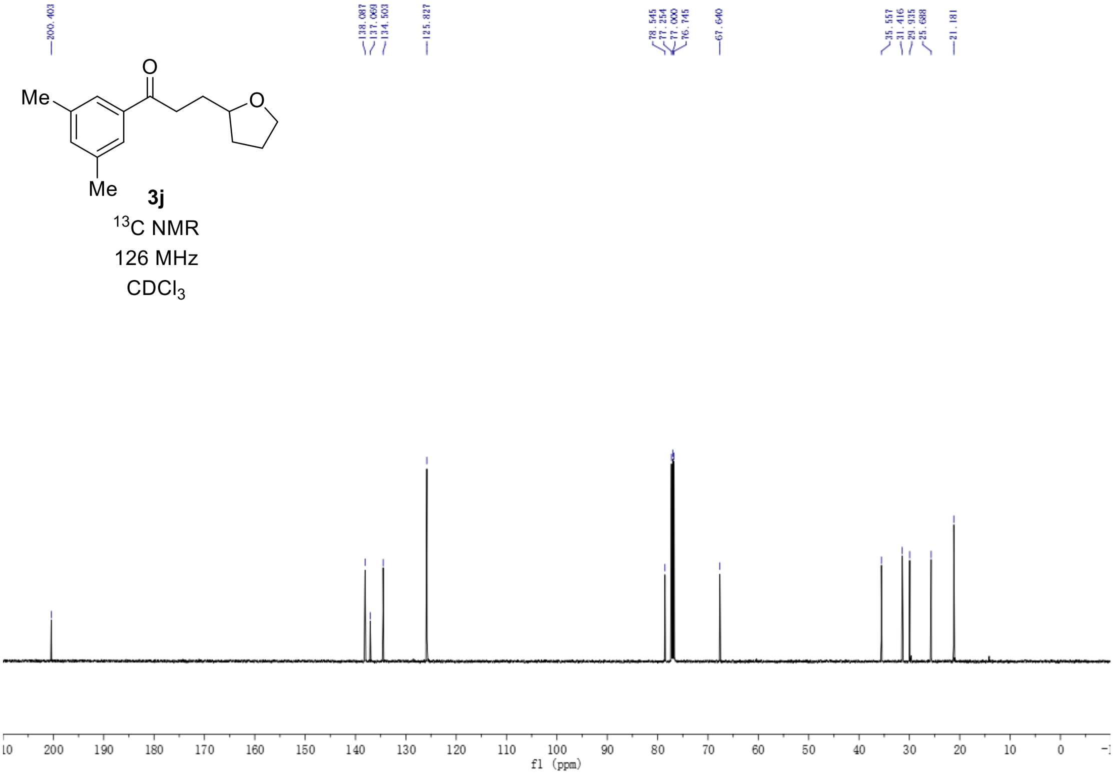

Supplementary Fig. 83 ^{13}C NMR spectrum of compound **3j**

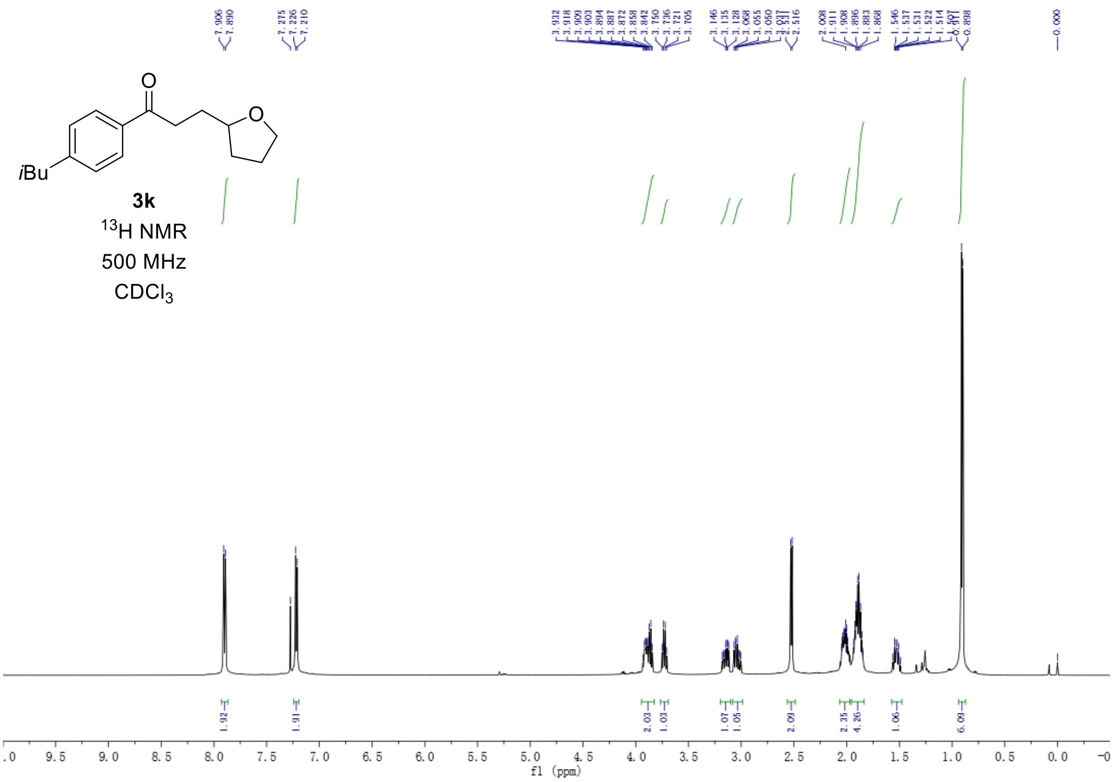

Supplementary Fig. 84 ^{1}H NMR spectrum of compound **3k**

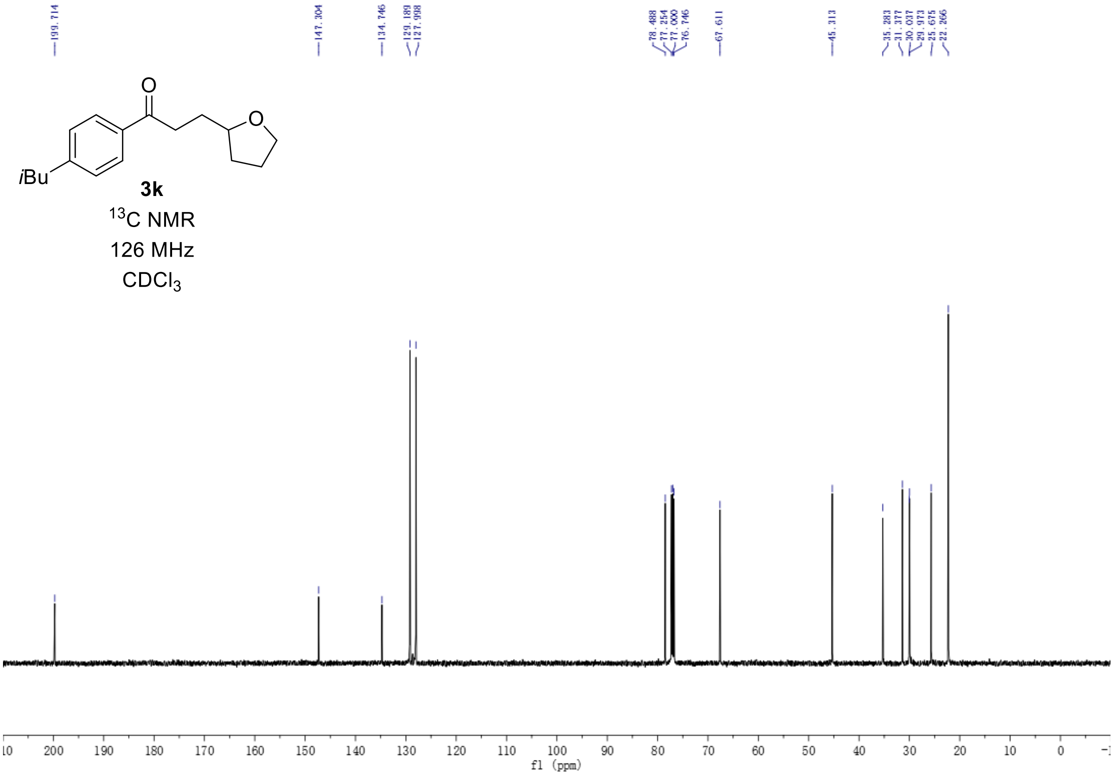

Supplementary Fig. 85 ^{13}C NMR spectrum of compound **3k**

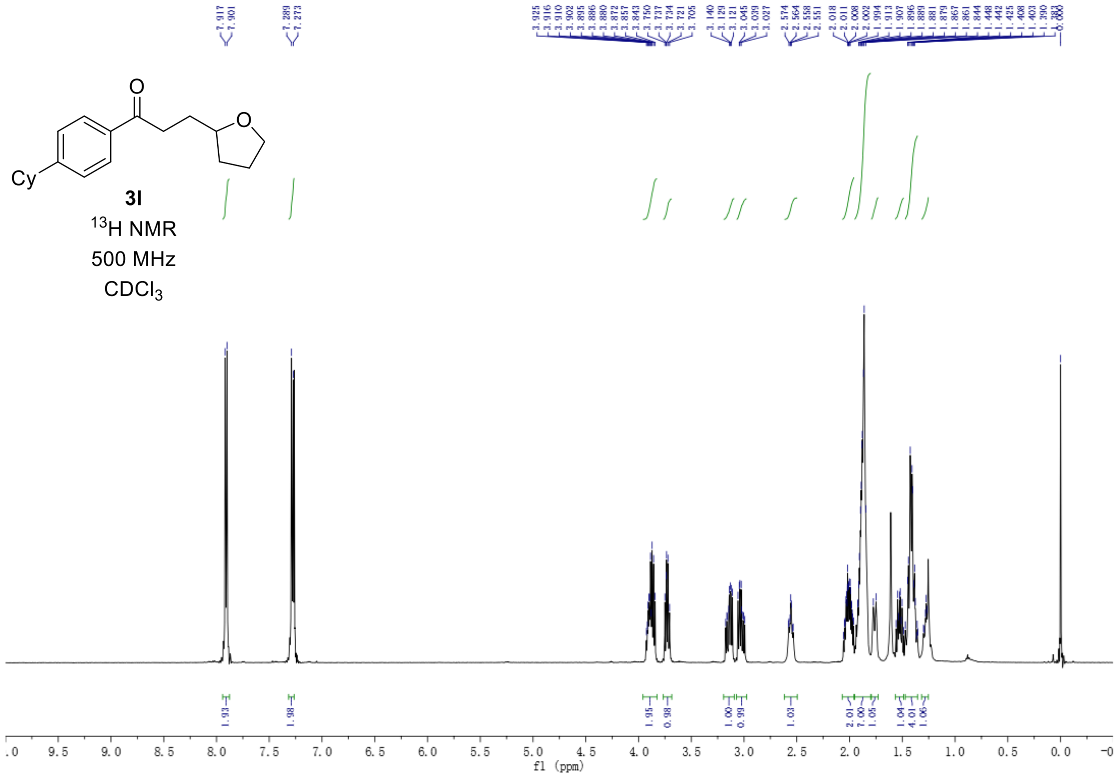

Supplementary Fig. 86 ^{1}H NMR spectrum of compound **3l**

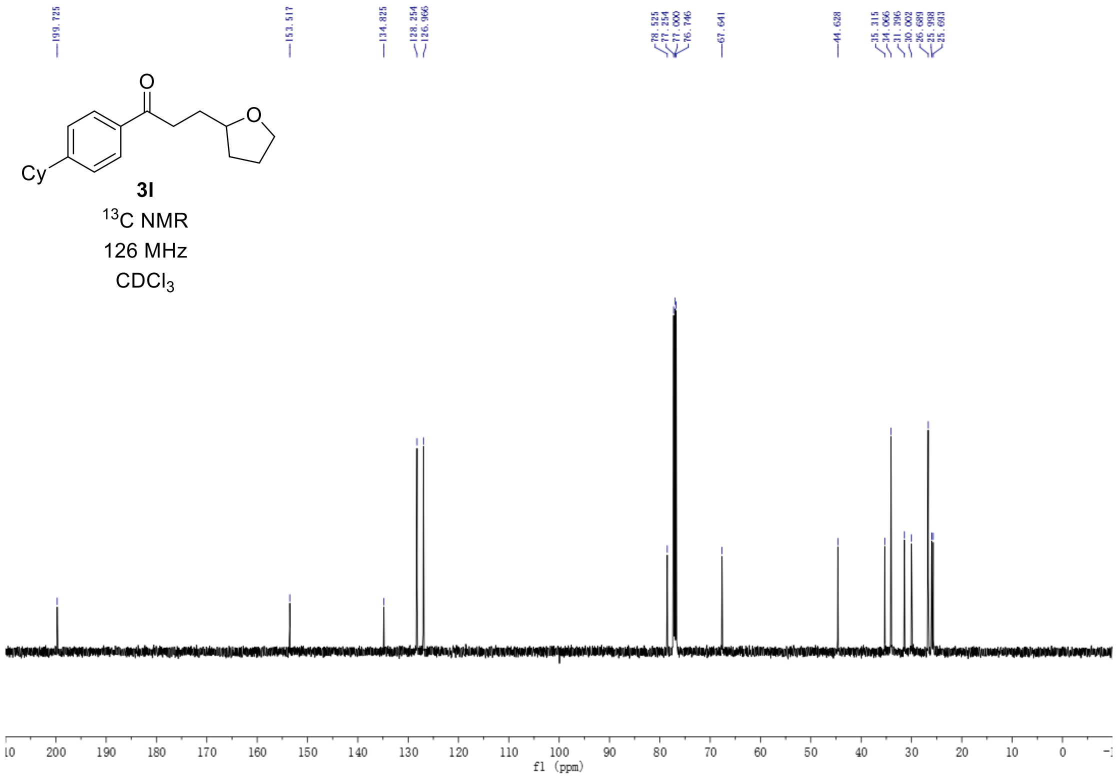

Supplementary Fig. 87 ^{13}C NMR spectrum of compound **3l**

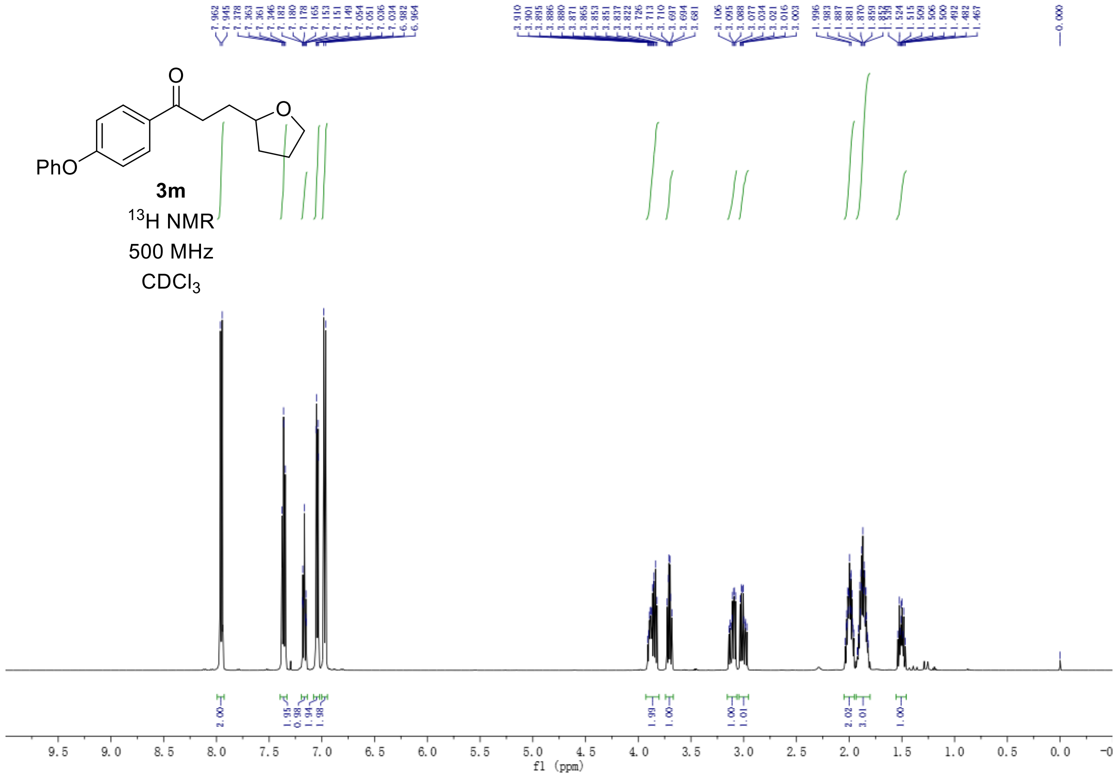

Supplementary Fig. 88 ^{1}H NMR spectrum of compound **3m**

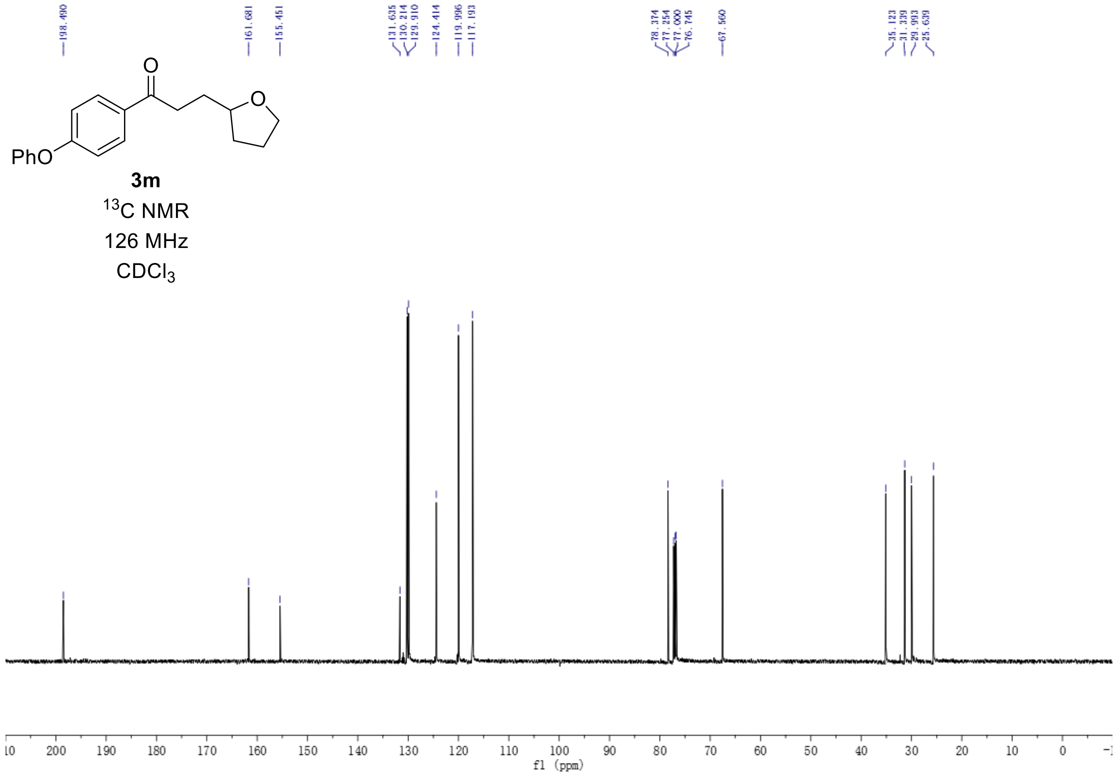

Supplementary Fig. 89 ^{13}C NMR spectrum of compound **3m**

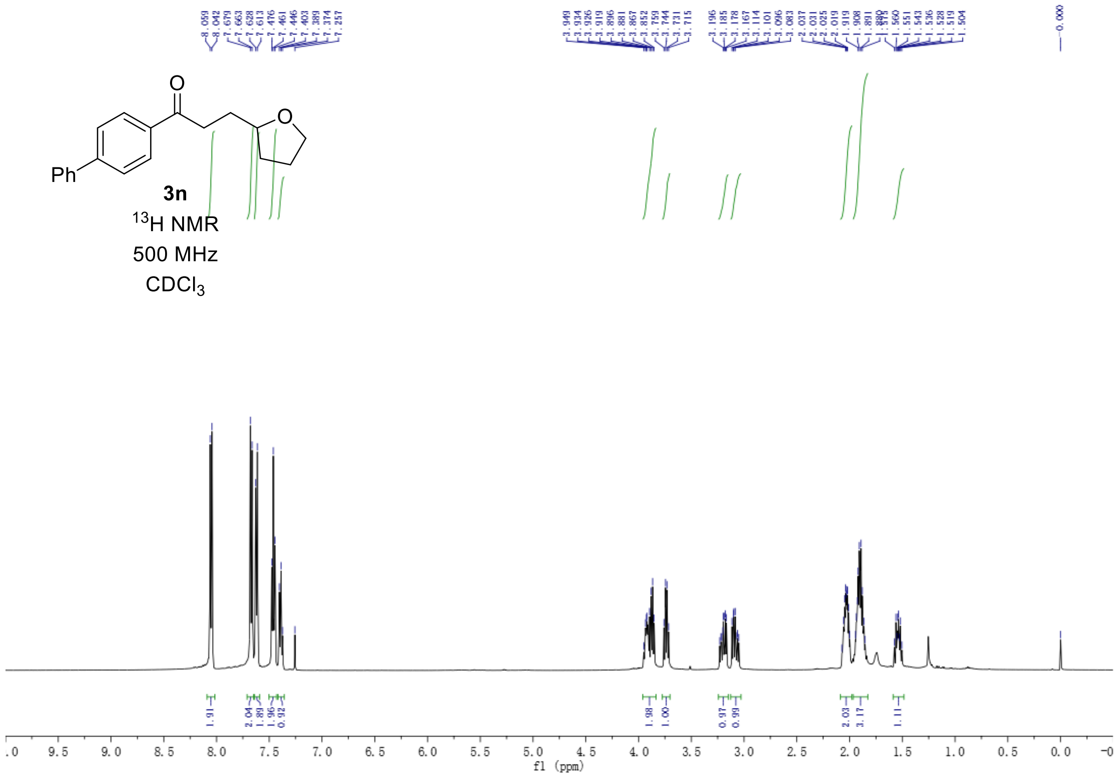

Supplementary Fig. 90 ^{1}H NMR spectrum of compound **3n**

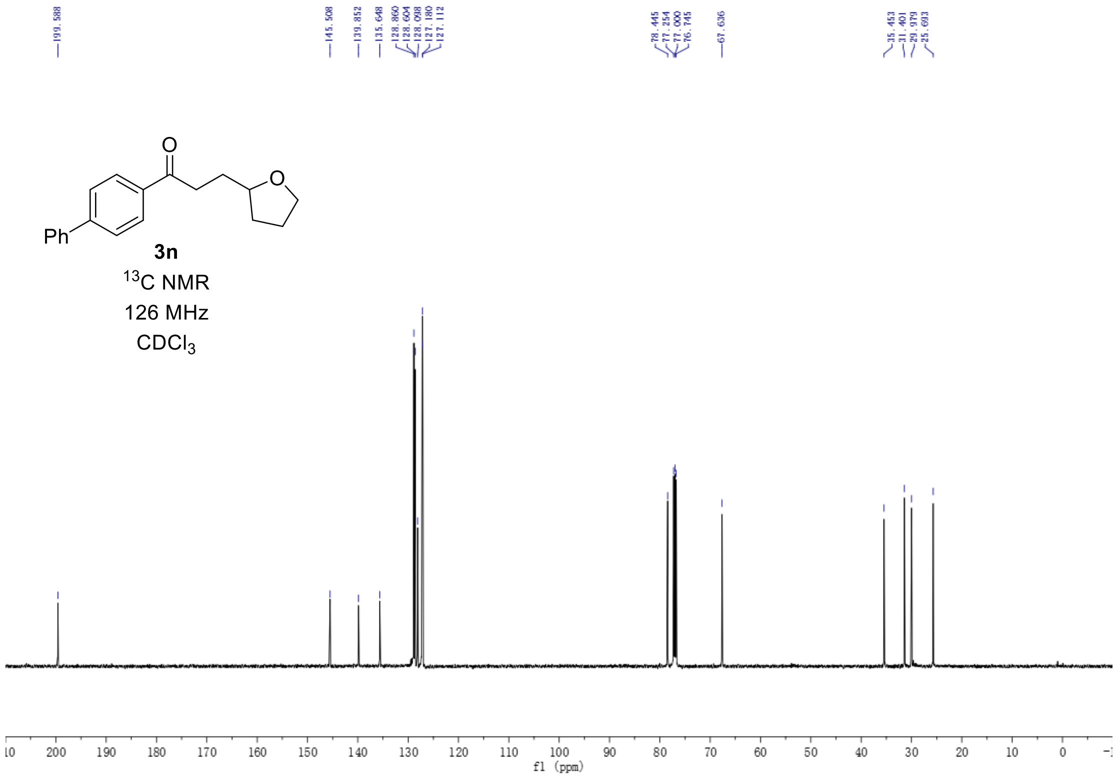

Supplementary Fig. 91 ^{13}C NMR spectrum of compound **3n**

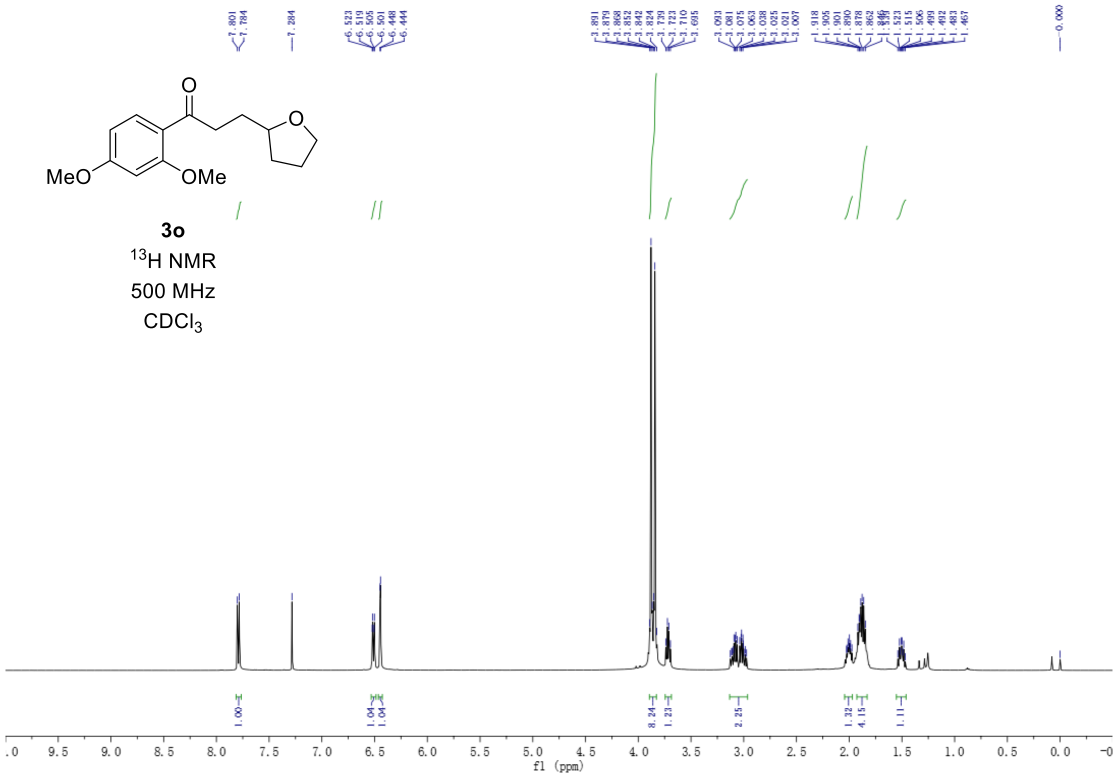

Supplementary Fig. 92 ^{1}H NMR spectrum of compound **3o**

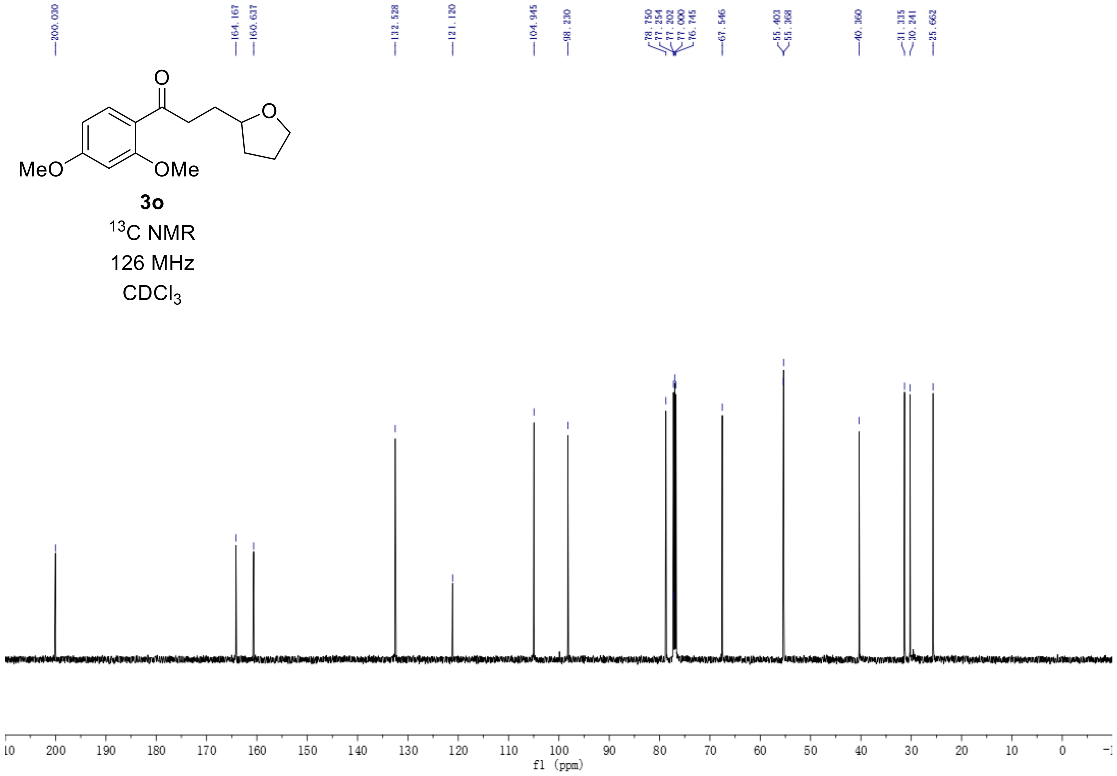

Supplementary Fig. 93 ^{13}C NMR spectrum of compound **3o**

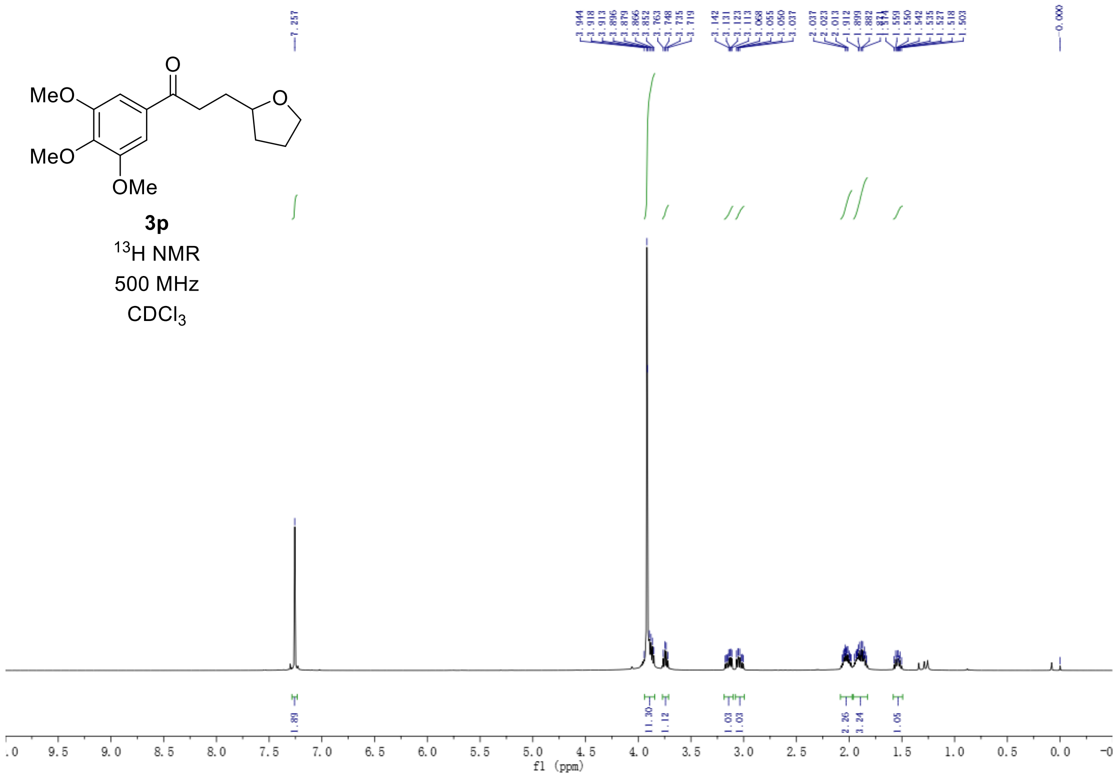

Supplementary Fig. 94 ^{1}H NMR spectrum of compound **3p**

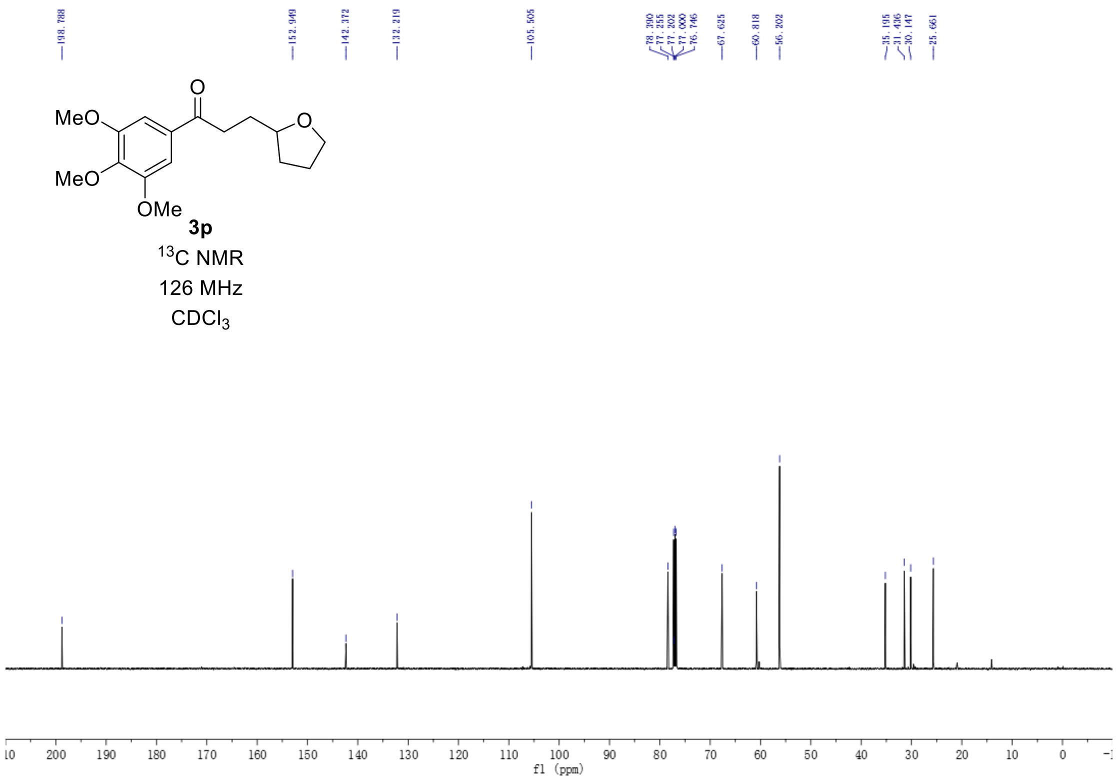

Supplementary Fig. 95 ^{13}C NMR spectrum of compound **3p**

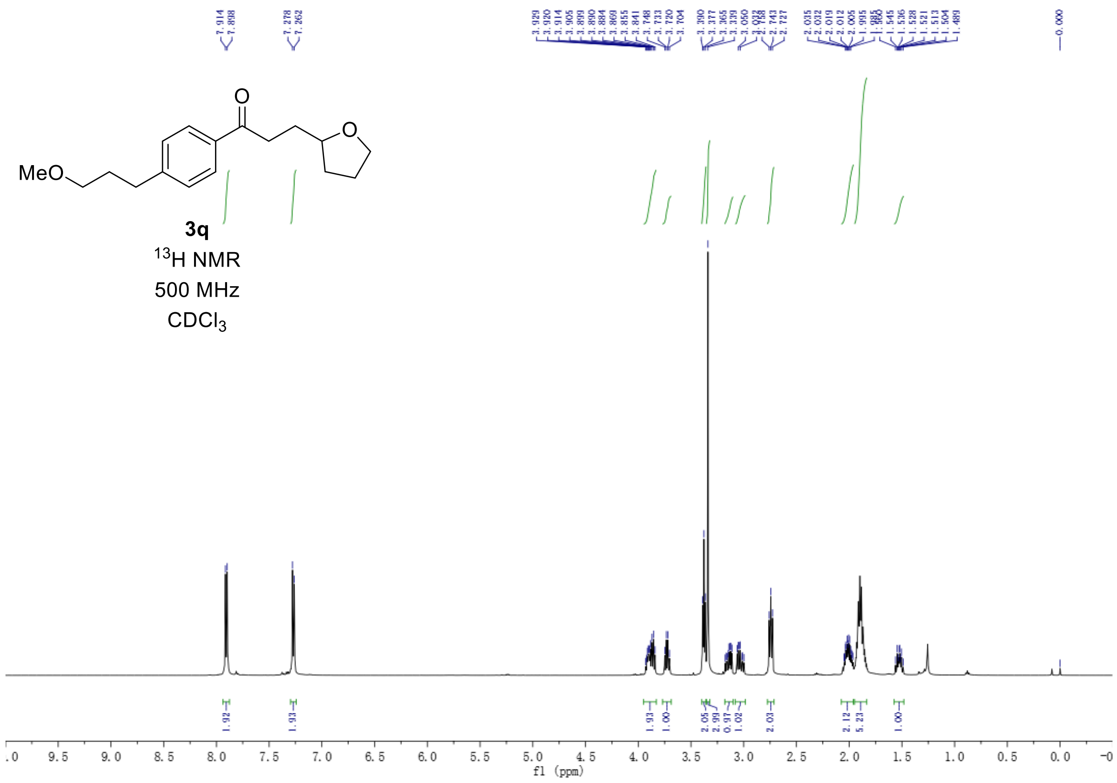

Supplementary Fig. 96 ^{1}H NMR spectrum of compound **3q**

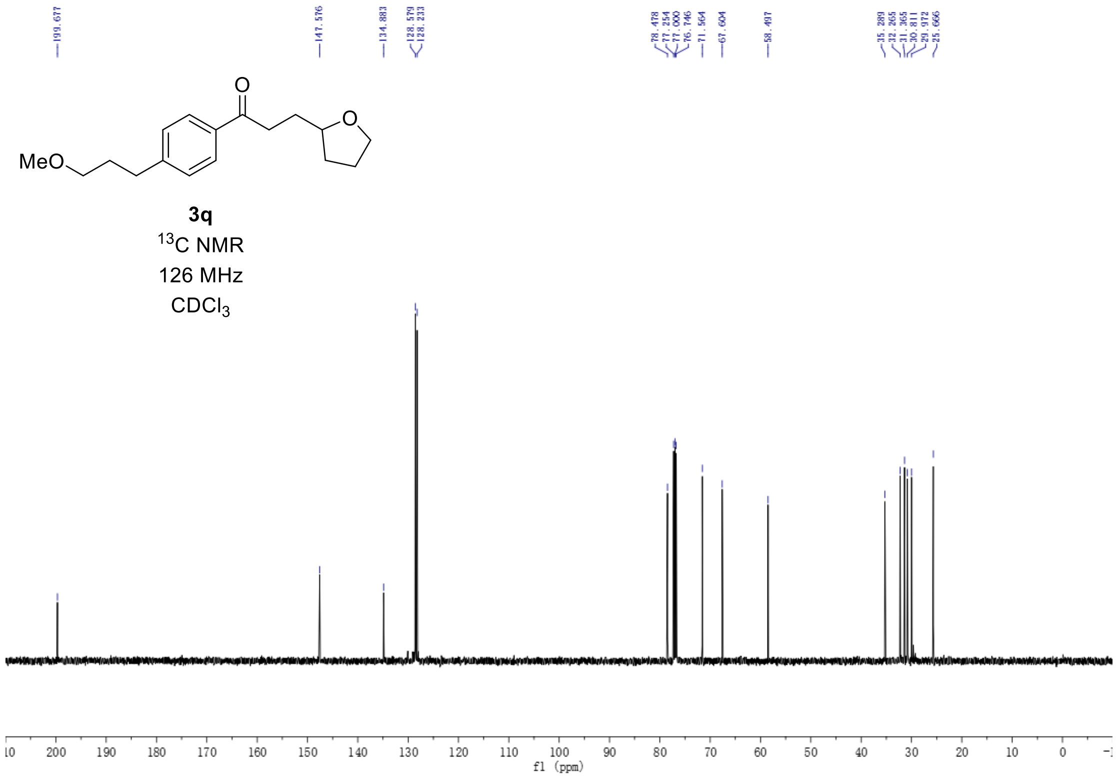

Supplementary Fig. 97 ^{13}C NMR spectrum of compound **3q**

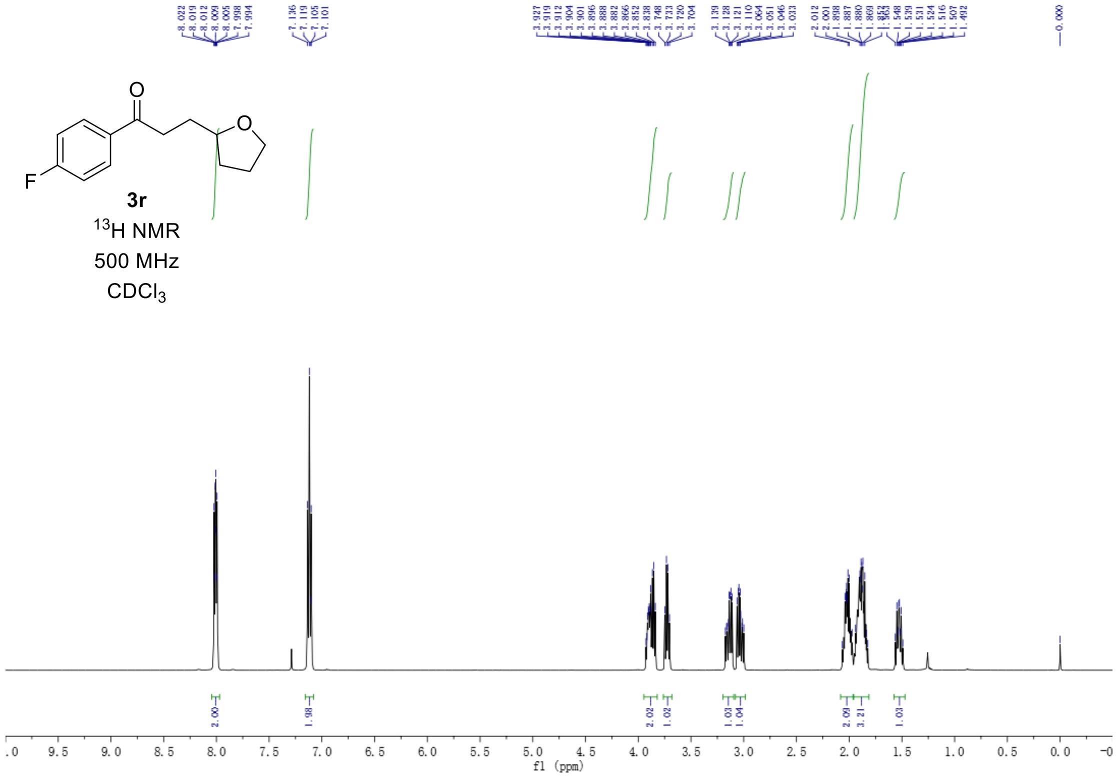

Supplementary Fig. 98 ^{1}H NMR spectrum of compound **3r**

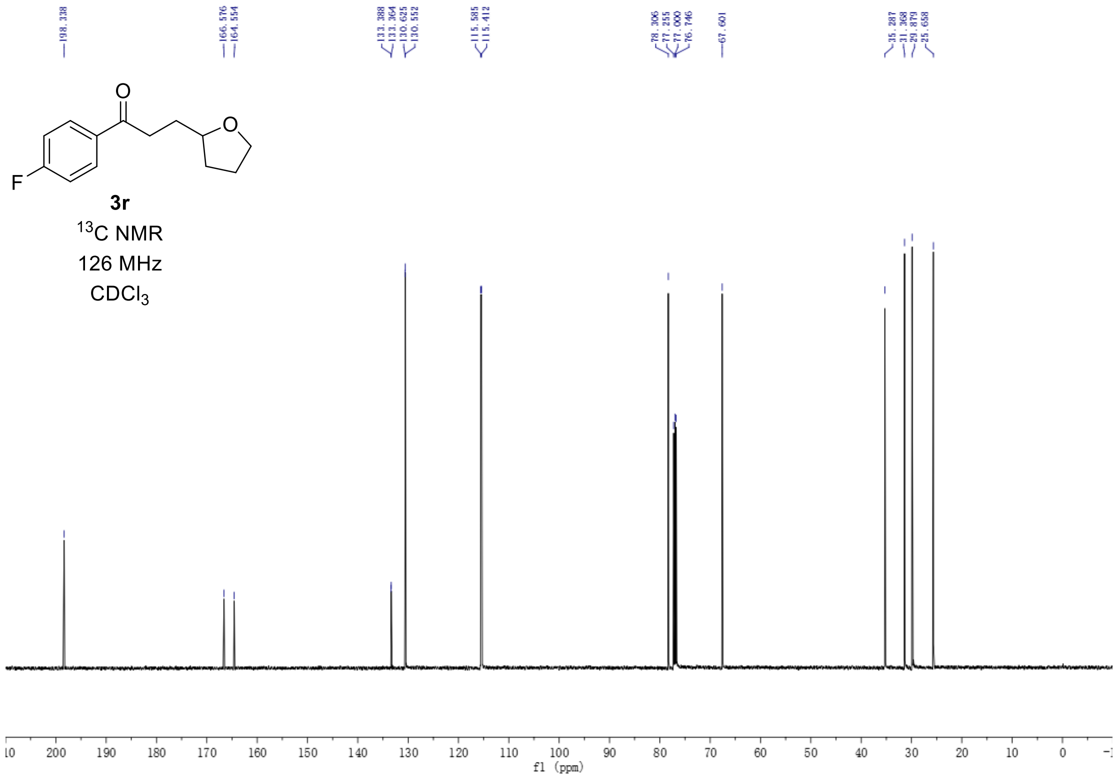

Supplementary Fig. 99 ^{13}C NMR spectrum of compound **3r**

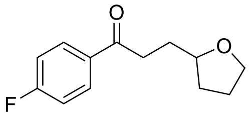

3r
^{19}F NMR
471 MHz
$CDCl_3$

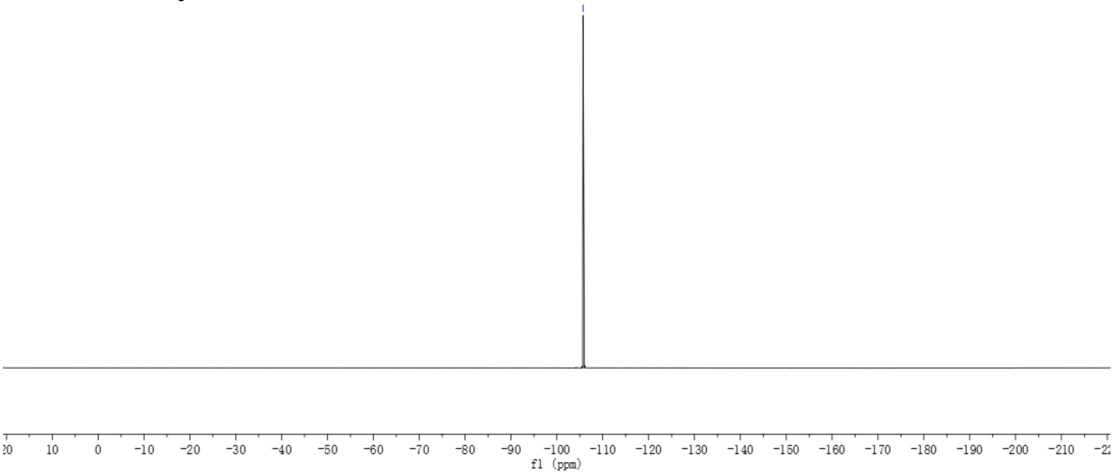

Supplementary Fig. 100 ^{19}F NMR spectrum of compound **3r**

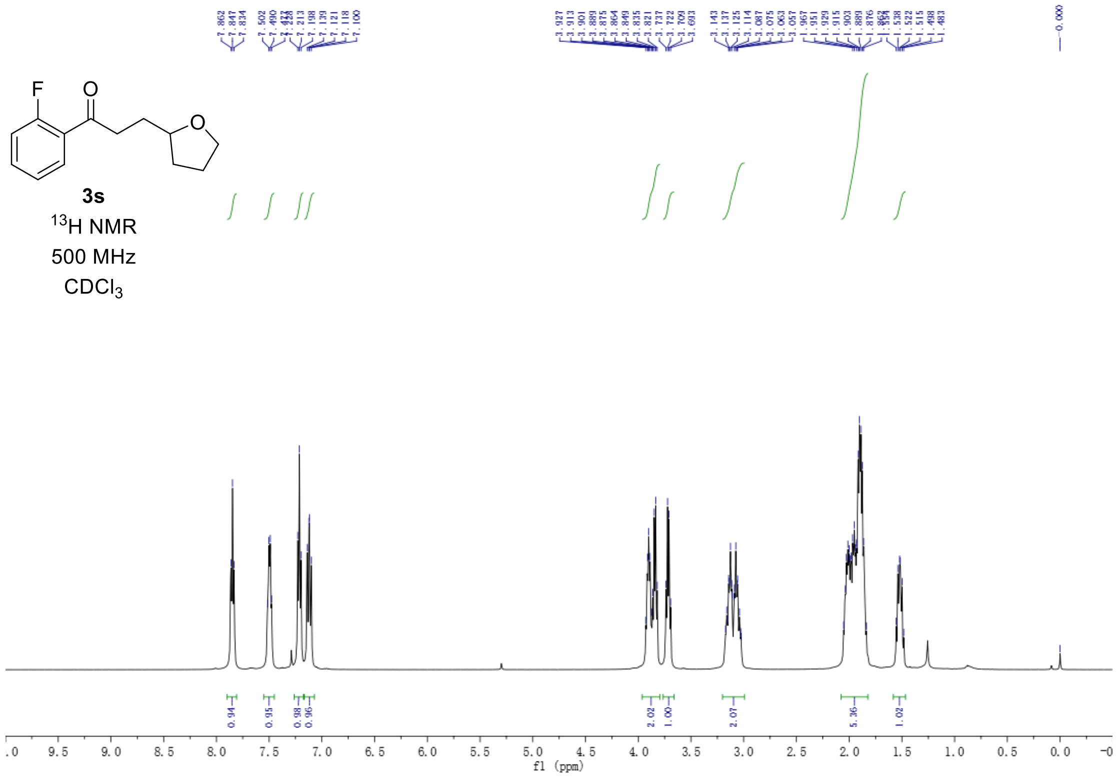

Supplementary Fig. 101 ^{1}H NMR spectrum of compound **3s**

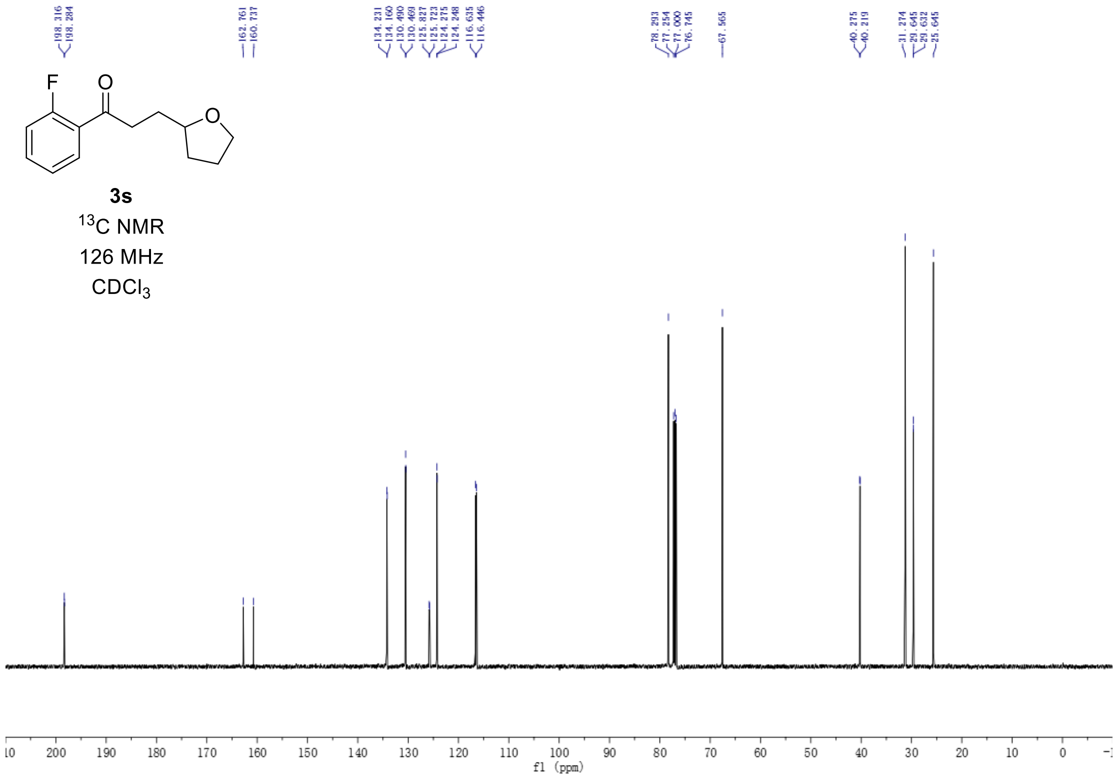

Supplementary Fig. 102 ^{13}C NMR spectrum of compound **3s**

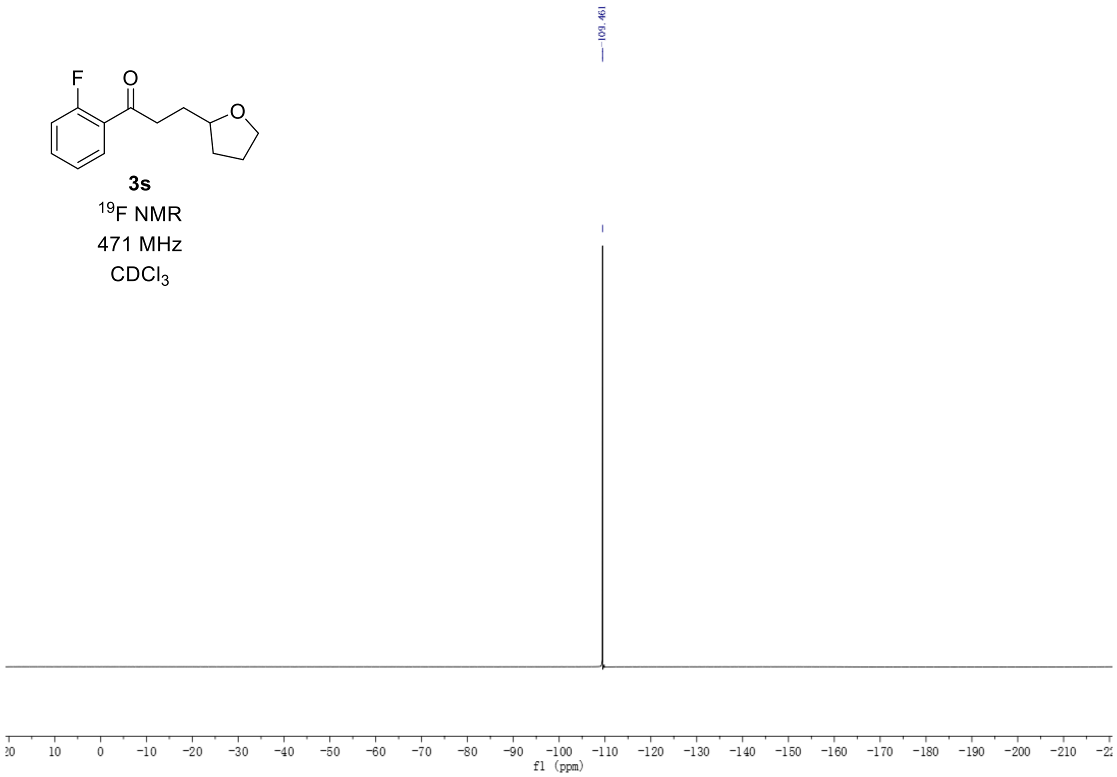

Supplementary Fig. 103 ^{19}F NMR spectrum of compound **3s**

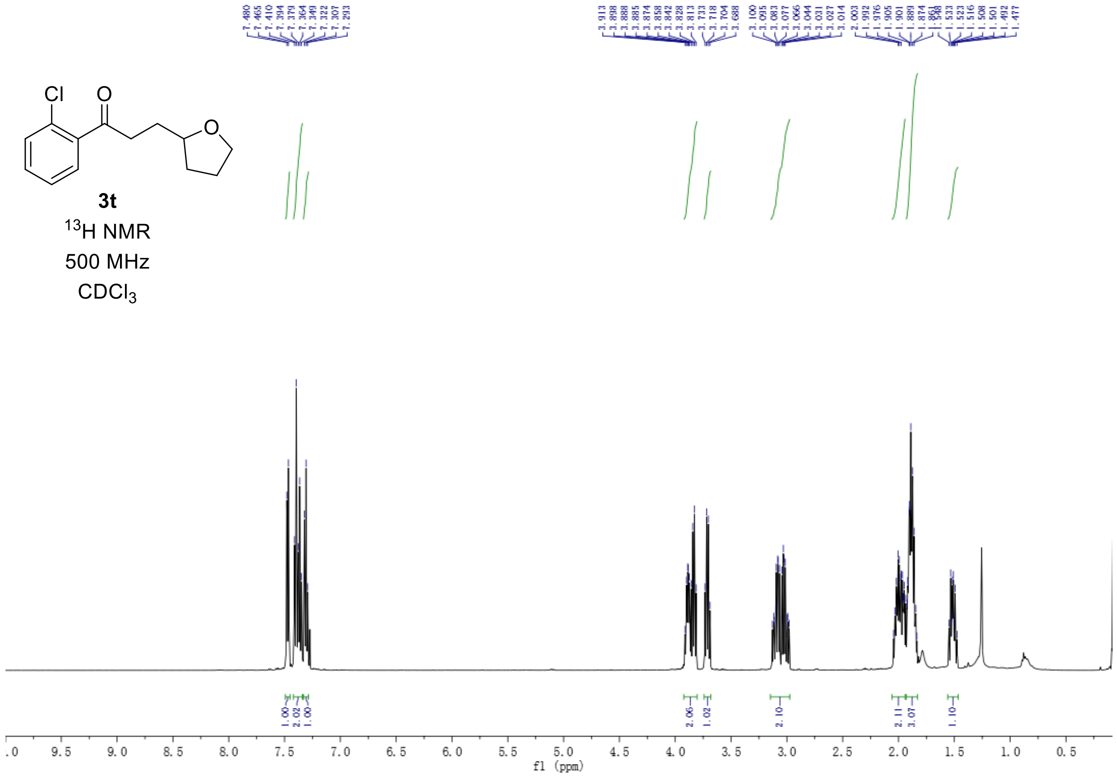

Supplementary Fig. 104 ^{1}H NMR spectrum of compound **3t**

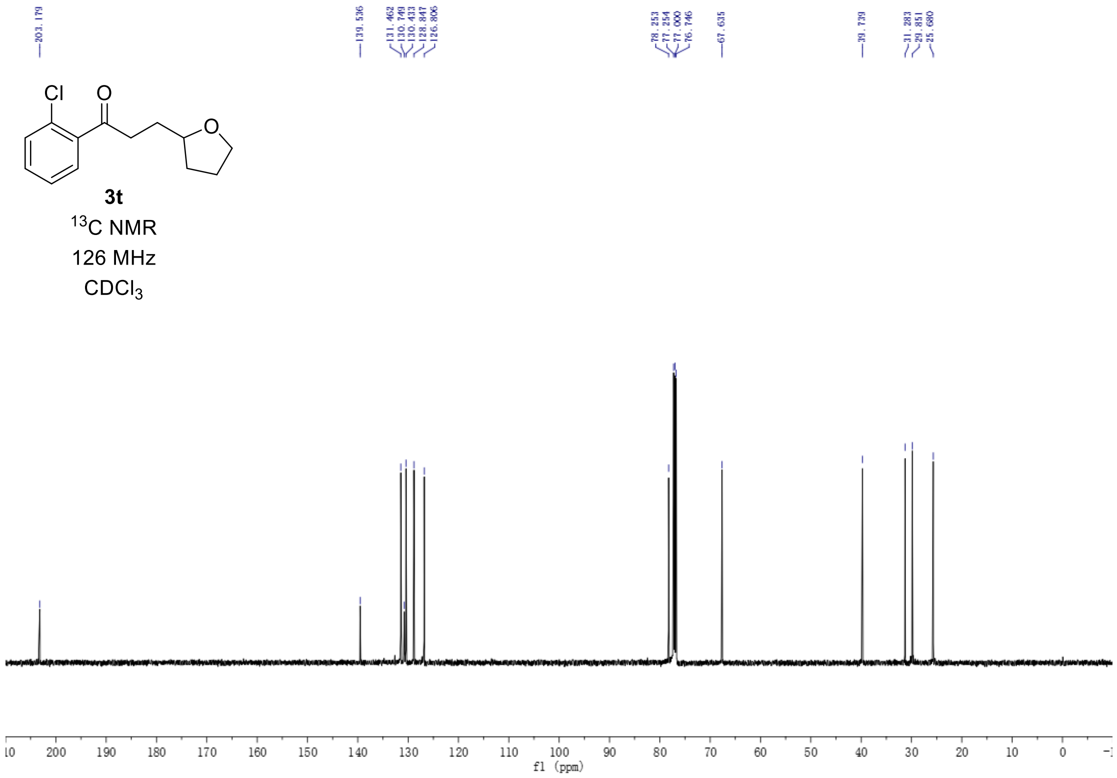

Supplementary Fig. 105 ^{13}C NMR spectrum of compound **3t**

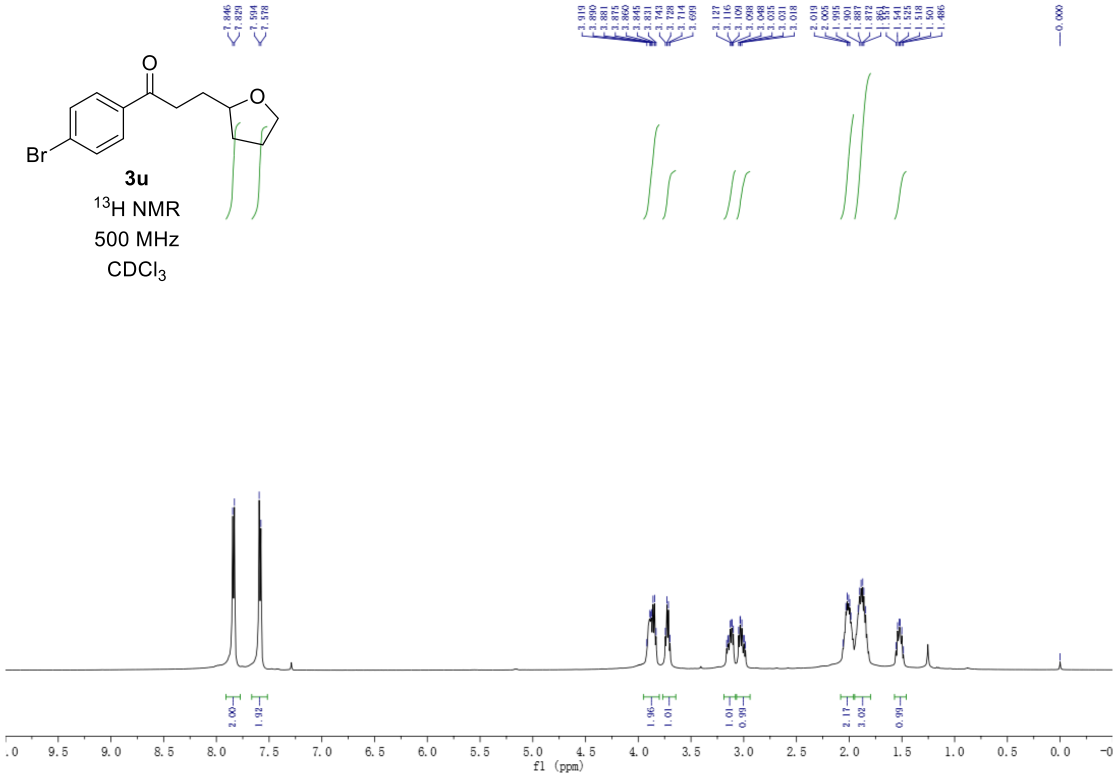

Supplementary Fig. 106 ^{1}H NMR spectrum of compound **3u**

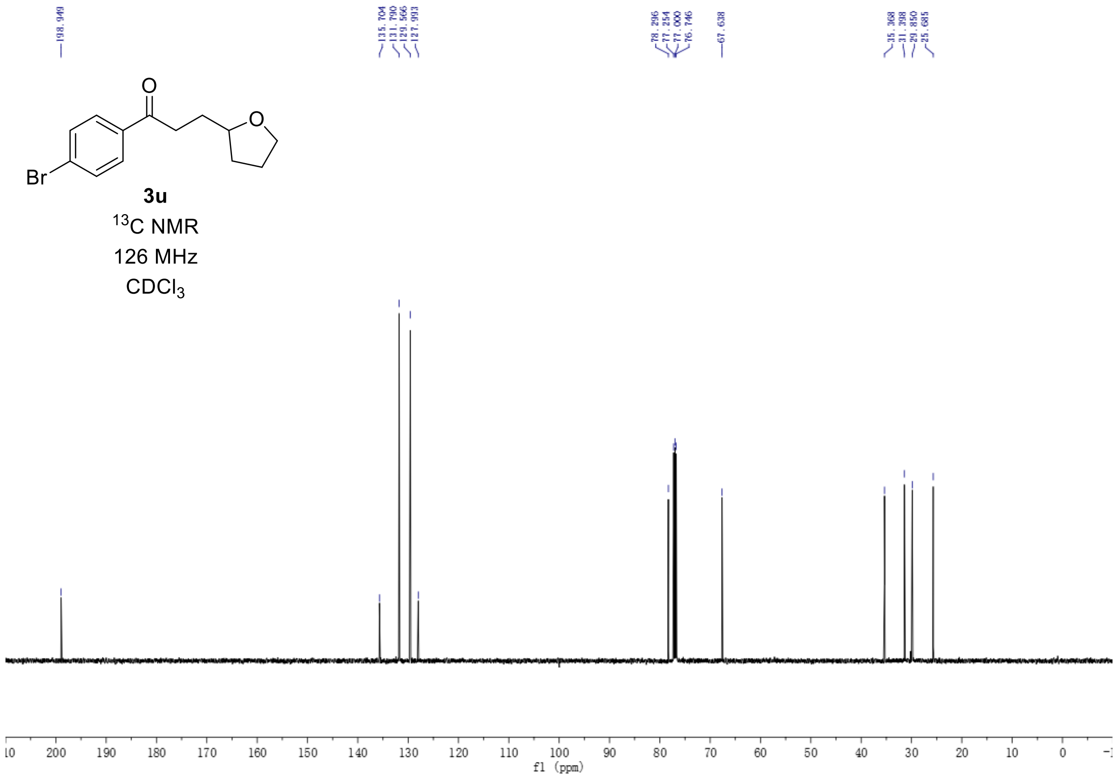

Supplementary Fig. 107 ^{13}C NMR spectrum of compound **3u**

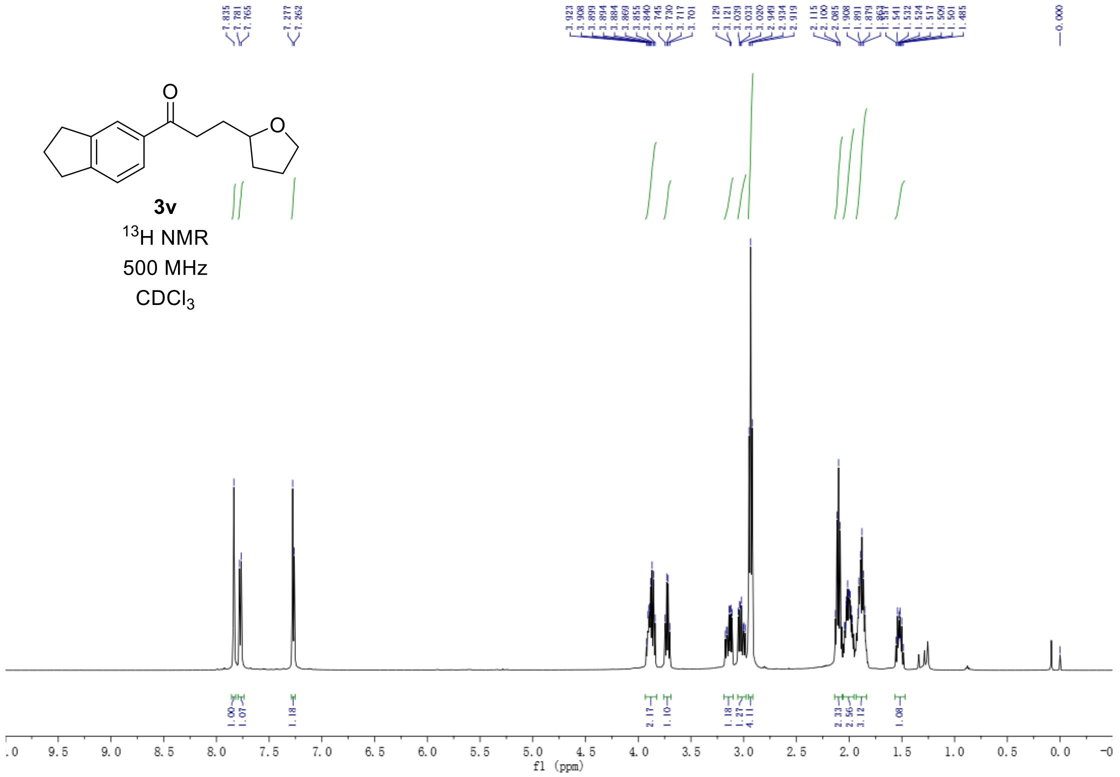

Supplementary Fig. 108 ^{1}H NMR spectrum of compound **3v**

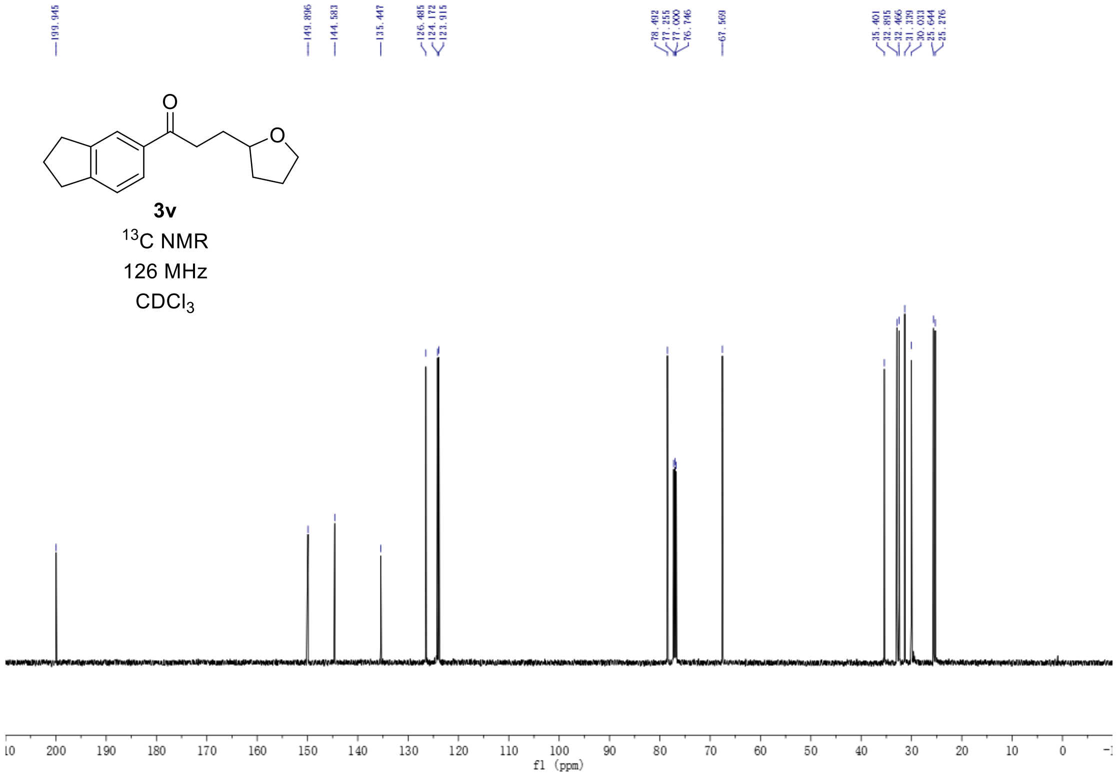

Supplementary Fig. 109 ^{13}C NMR spectrum of compound **3v**

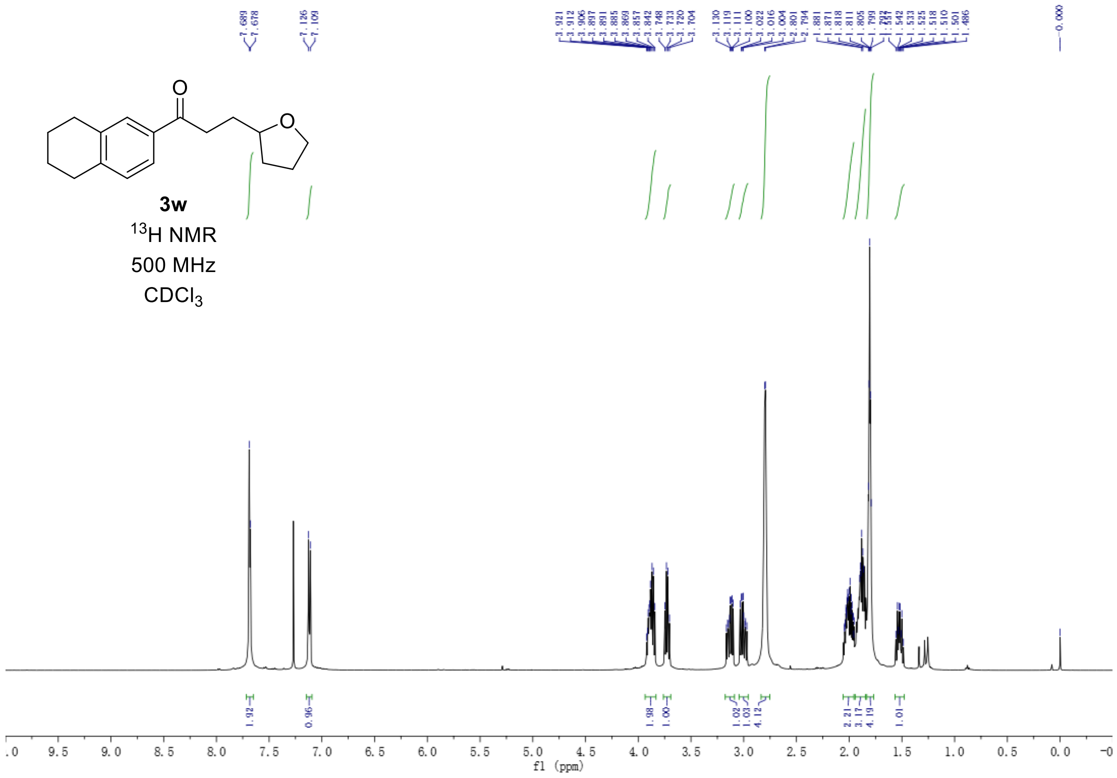

Supplementary Fig. 110 ^{1}H NMR spectrum of compound **3w**

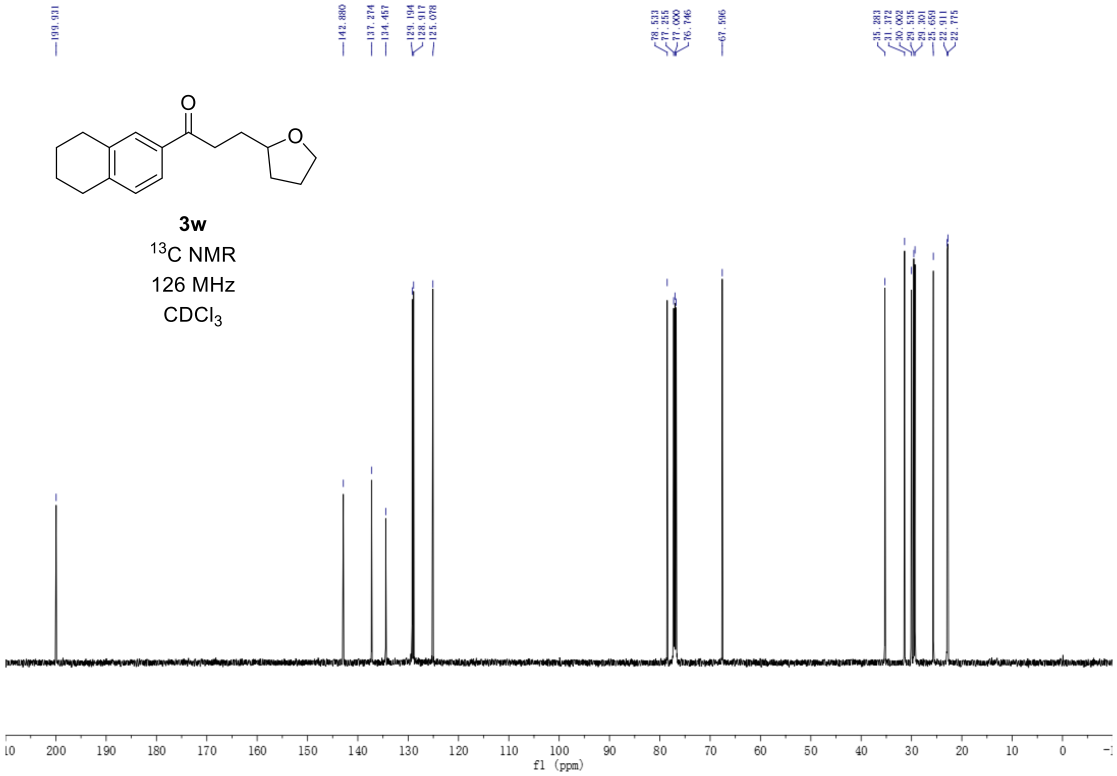

Supplementary Fig. 111 ^{13}C NMR spectrum of compound **3w**

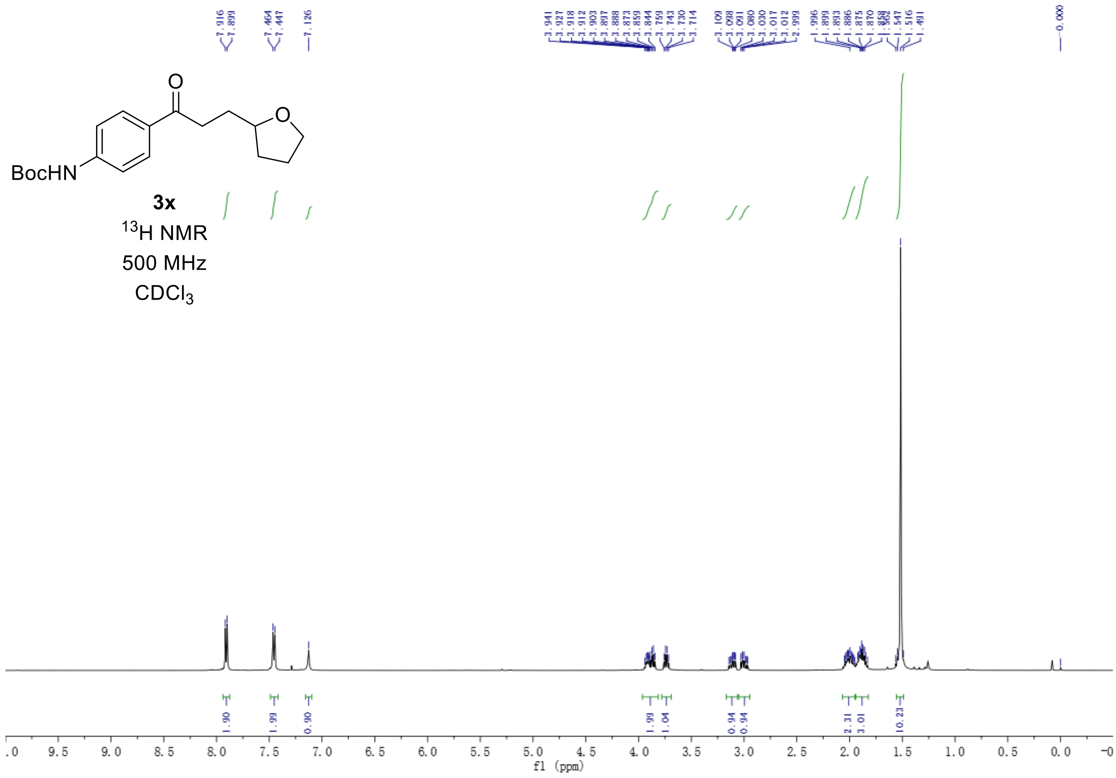

Supplementary Fig. 112 ^{1}H NMR spectrum of compound **3x**

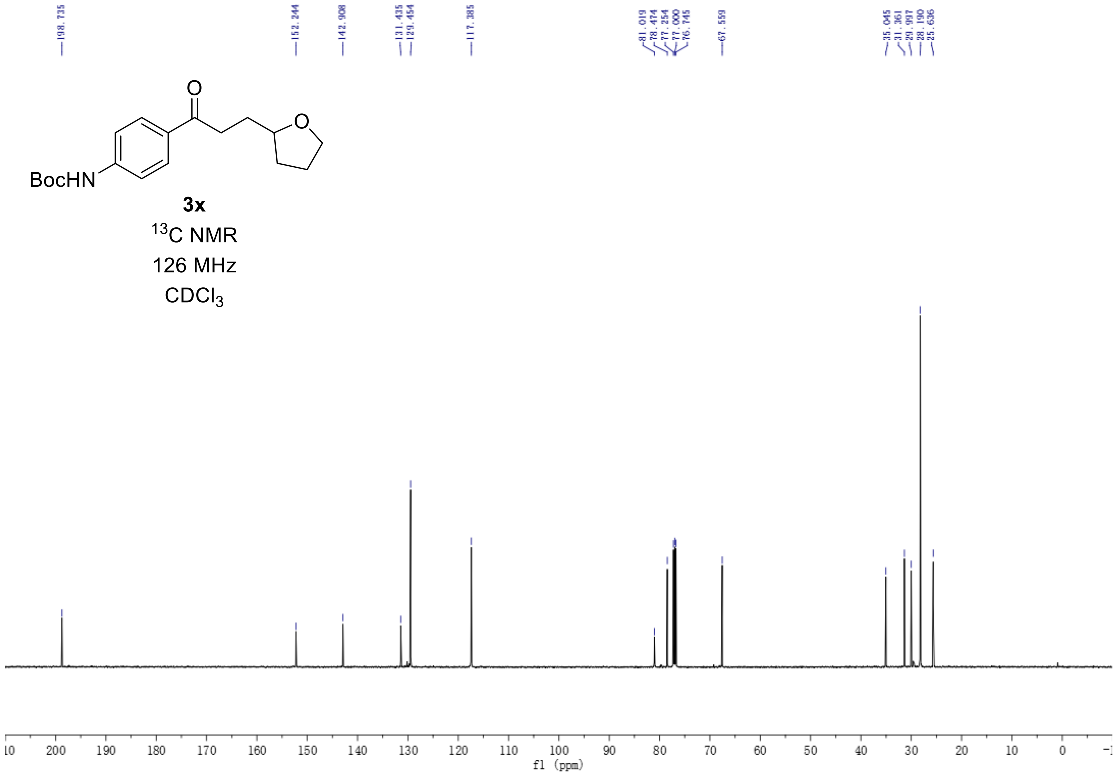

Supplementary Fig. 113 ^{13}C NMR spectrum of compound **3x**

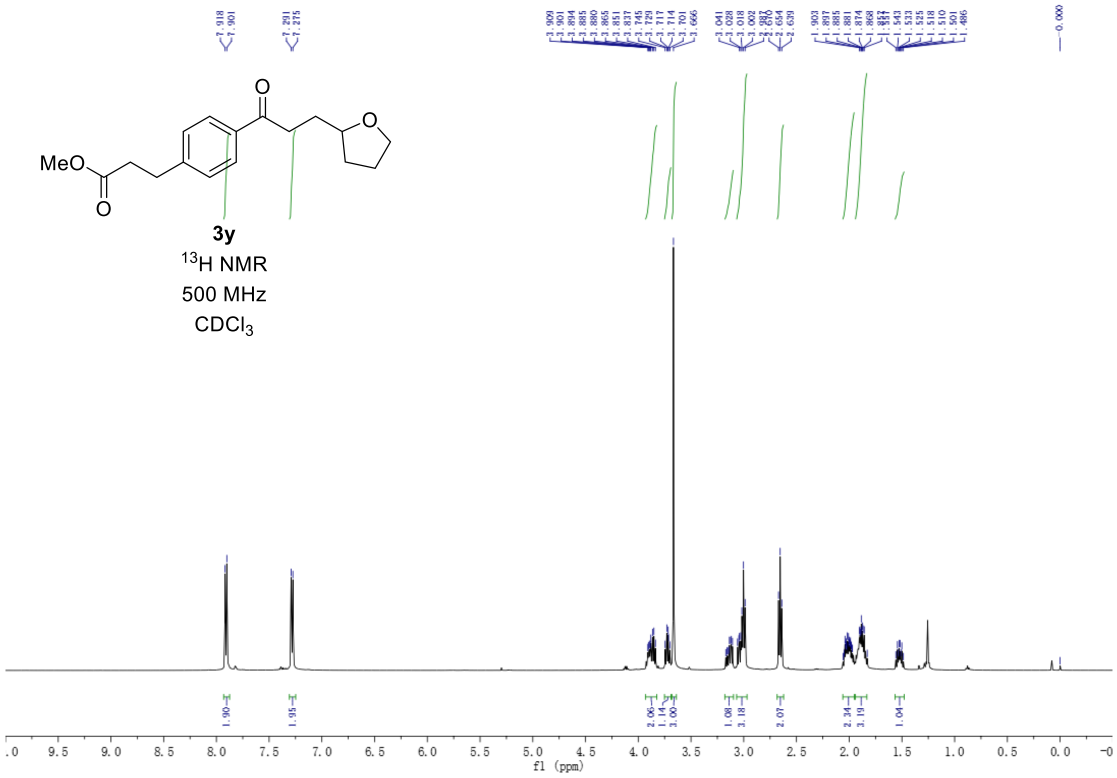

Supplementary Fig. 114 ^{1}H NMR spectrum of compound **3y**

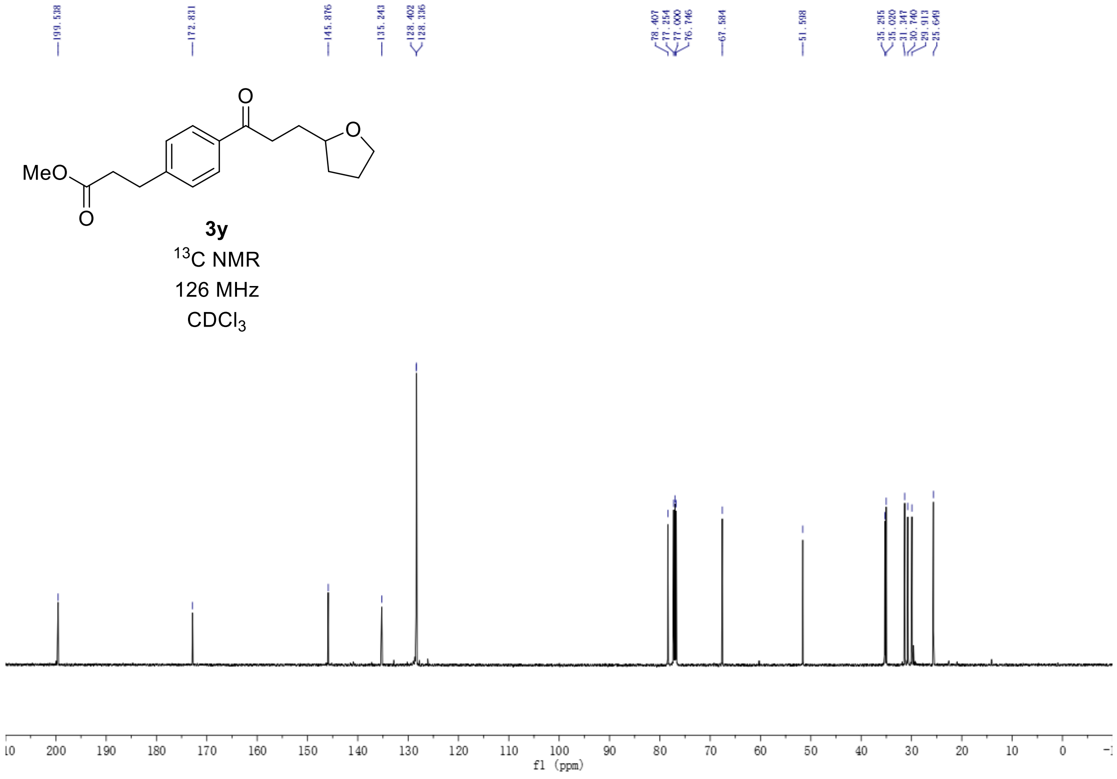

Supplementary Fig. 115 ^{13}C NMR spectrum of compound **3y**

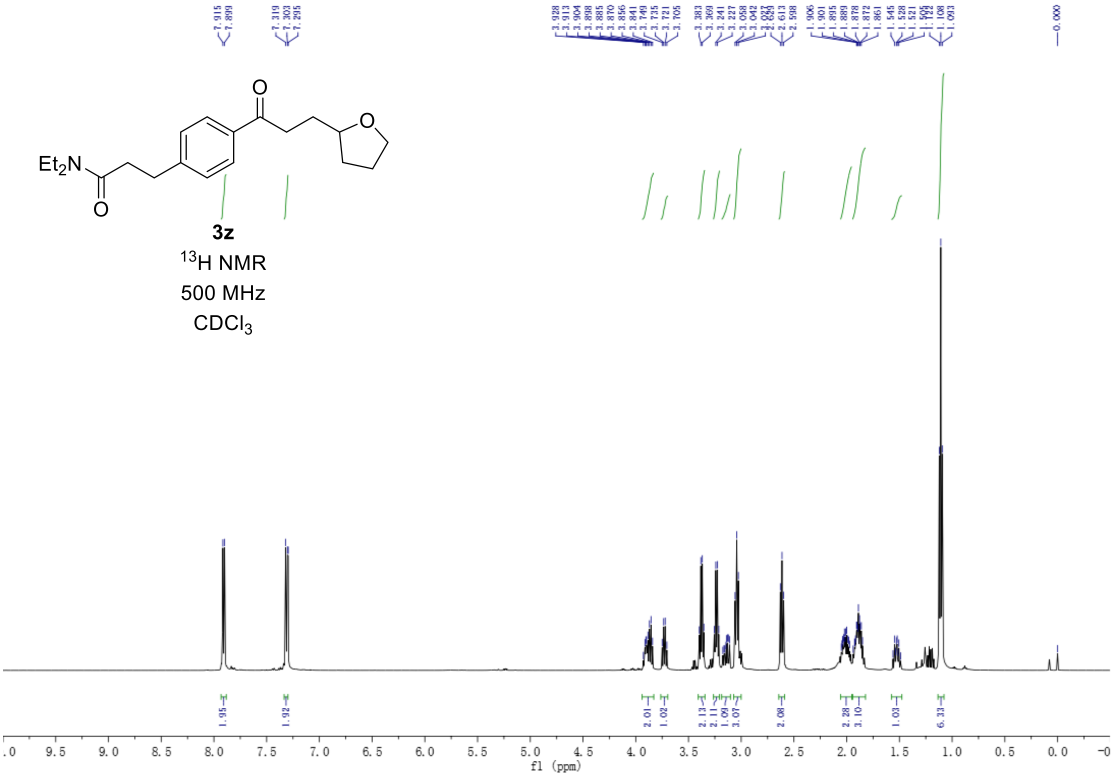

Supplementary Fig. 116 ^{1}H NMR spectrum of compound **3z**

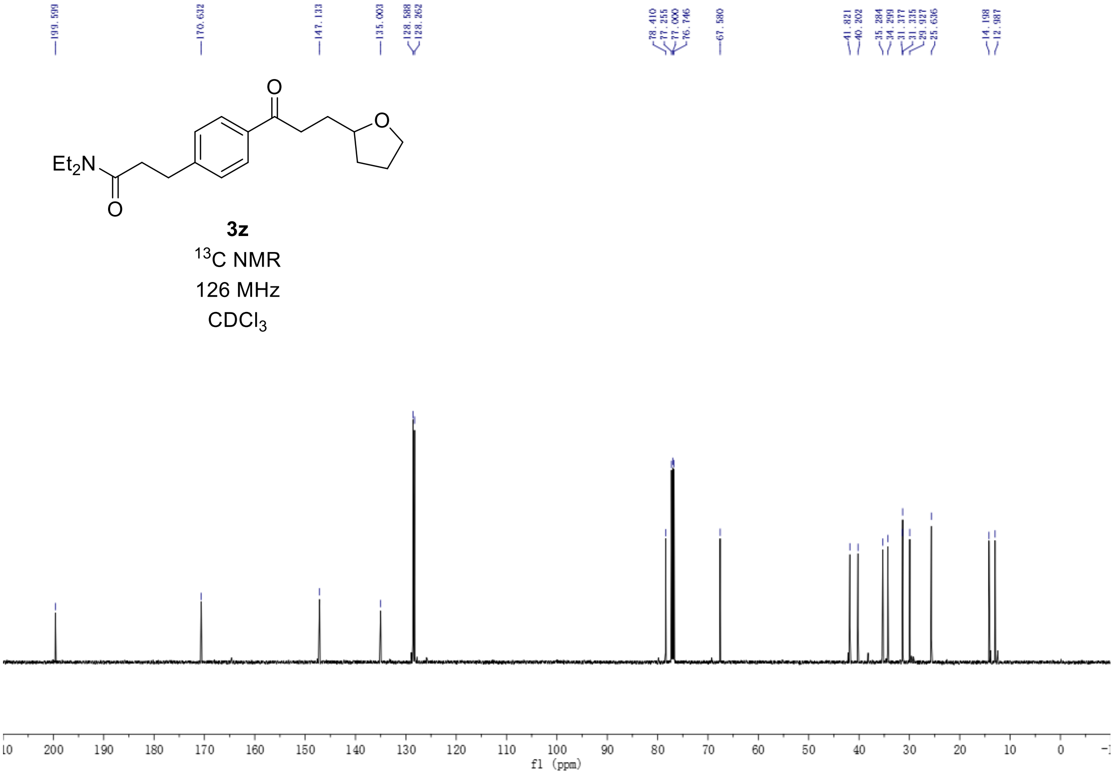

Supplementary Fig. 117 ^{13}C NMR spectrum of compound **3z**

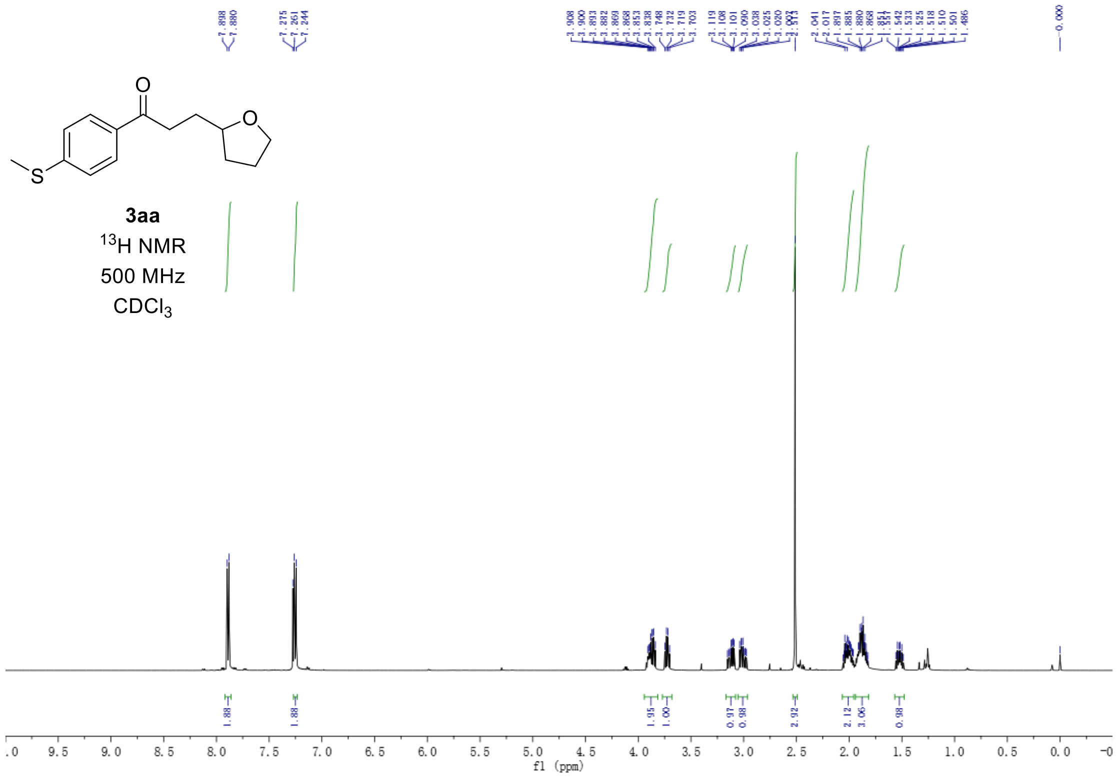

Supplementary Fig. 118 ^{1}H NMR spectrum of compound **3aa**

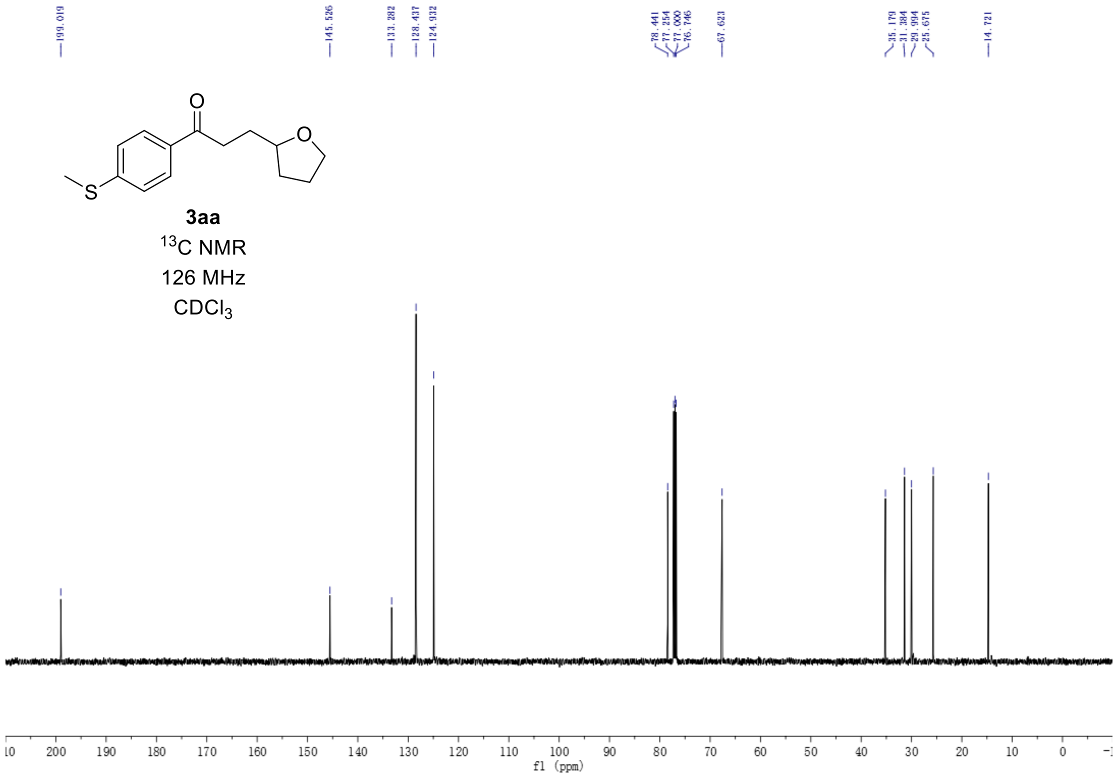

Supplementary Fig. 119 ^{13}C NMR spectrum of compound **3aa**

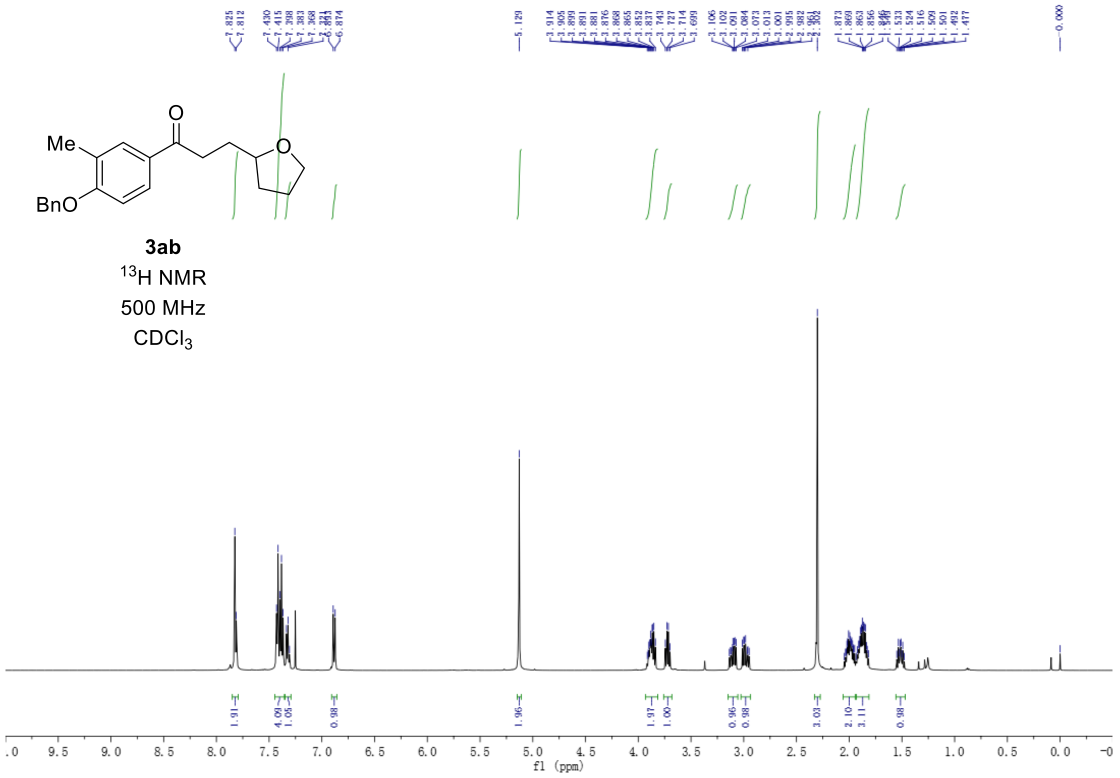

Supplementary Fig. 120 ^{1}H NMR spectrum of compound **3ab**

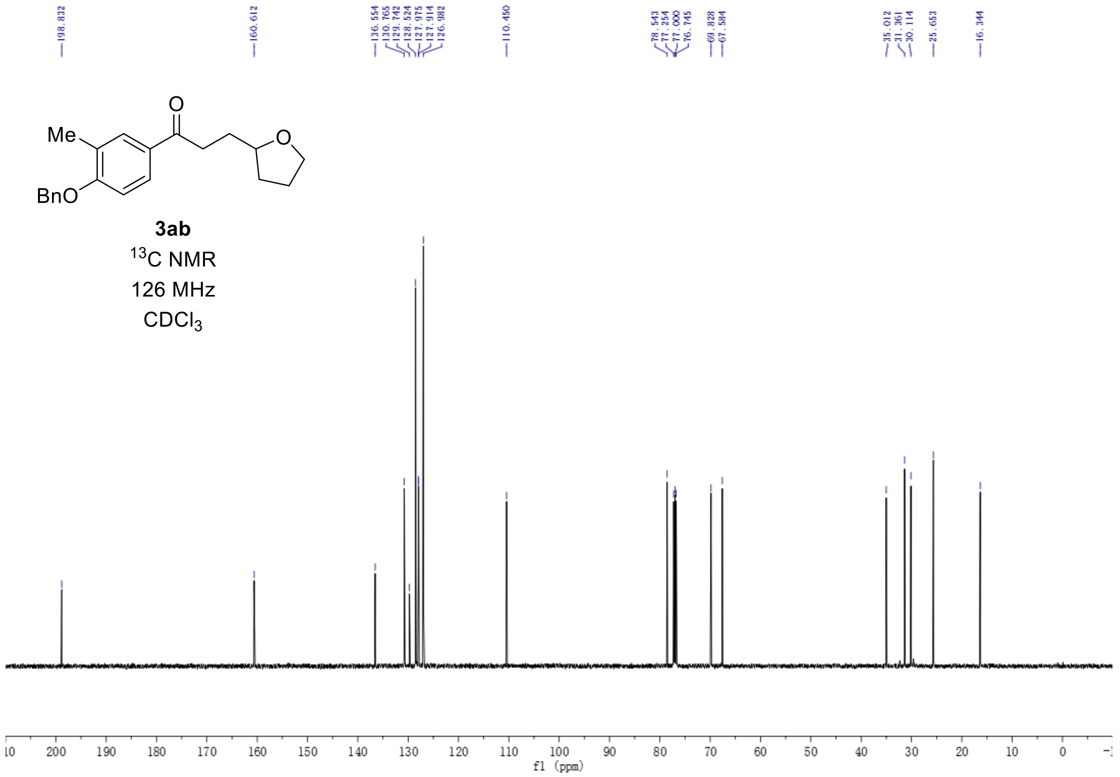

Supplementary Fig. 121 ^{13}C NMR spectrum of compound **3ab**

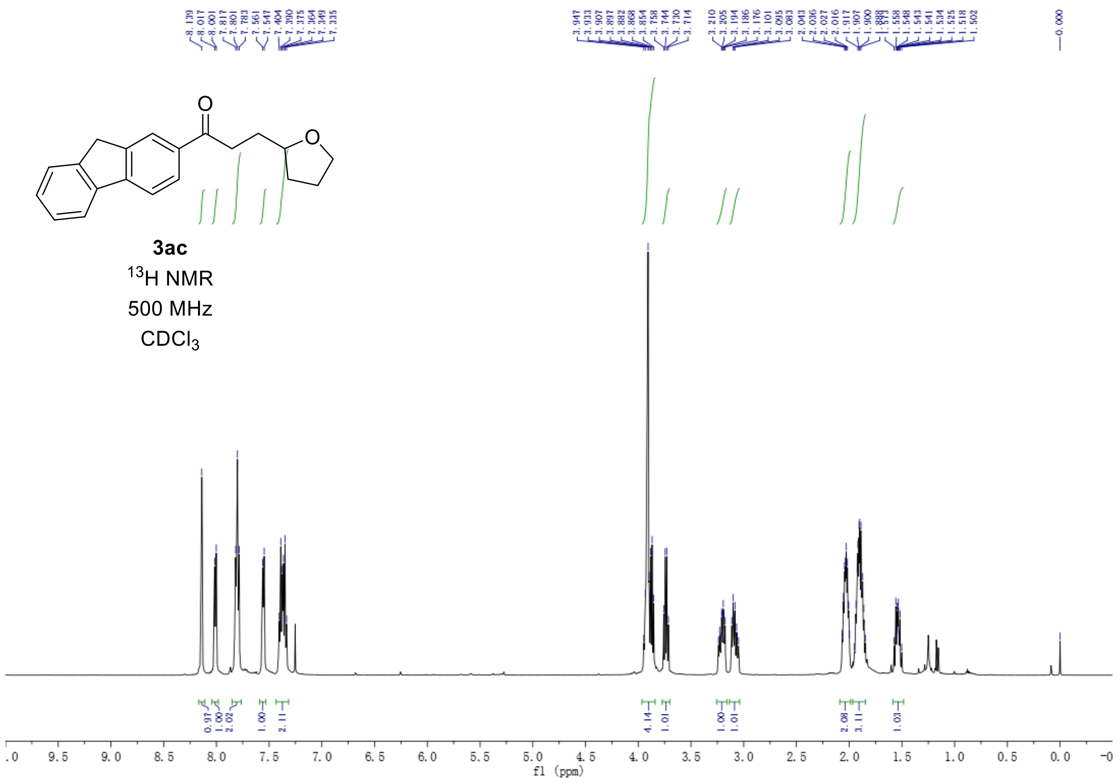

Supplementary Fig. 122 ^{1}H NMR spectrum of compound **3ac**

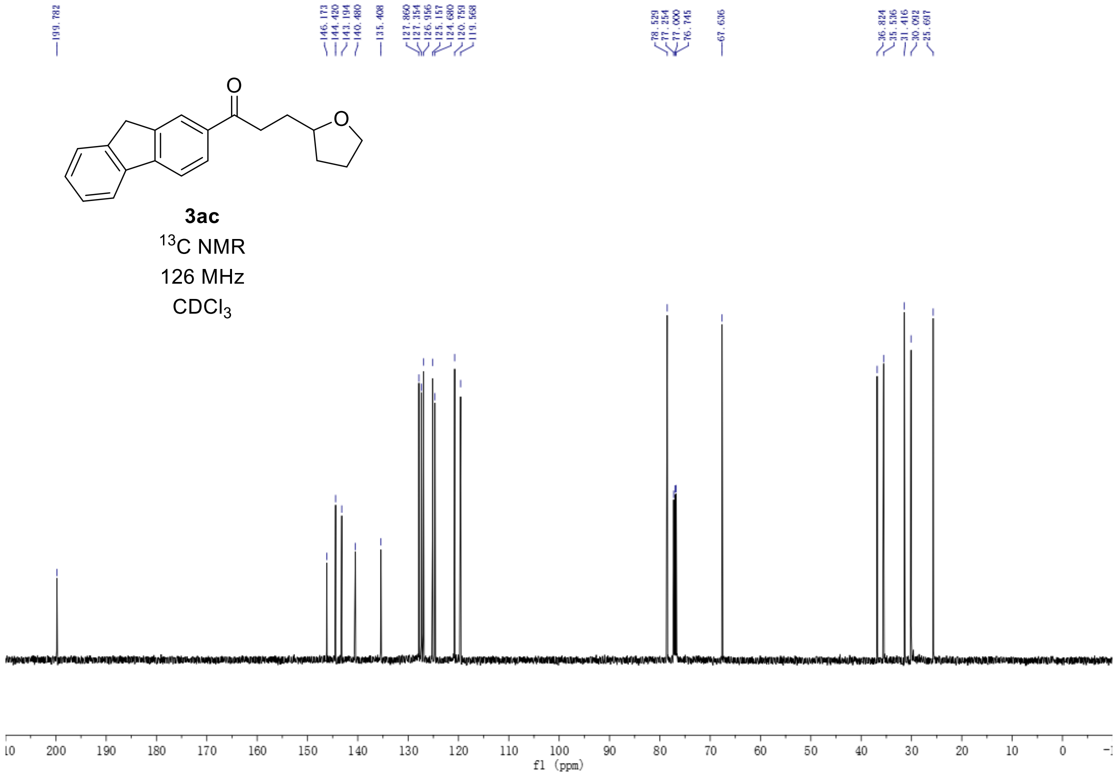

Supplementary Fig. 123 ^{13}C NMR spectrum of compound **3ac**

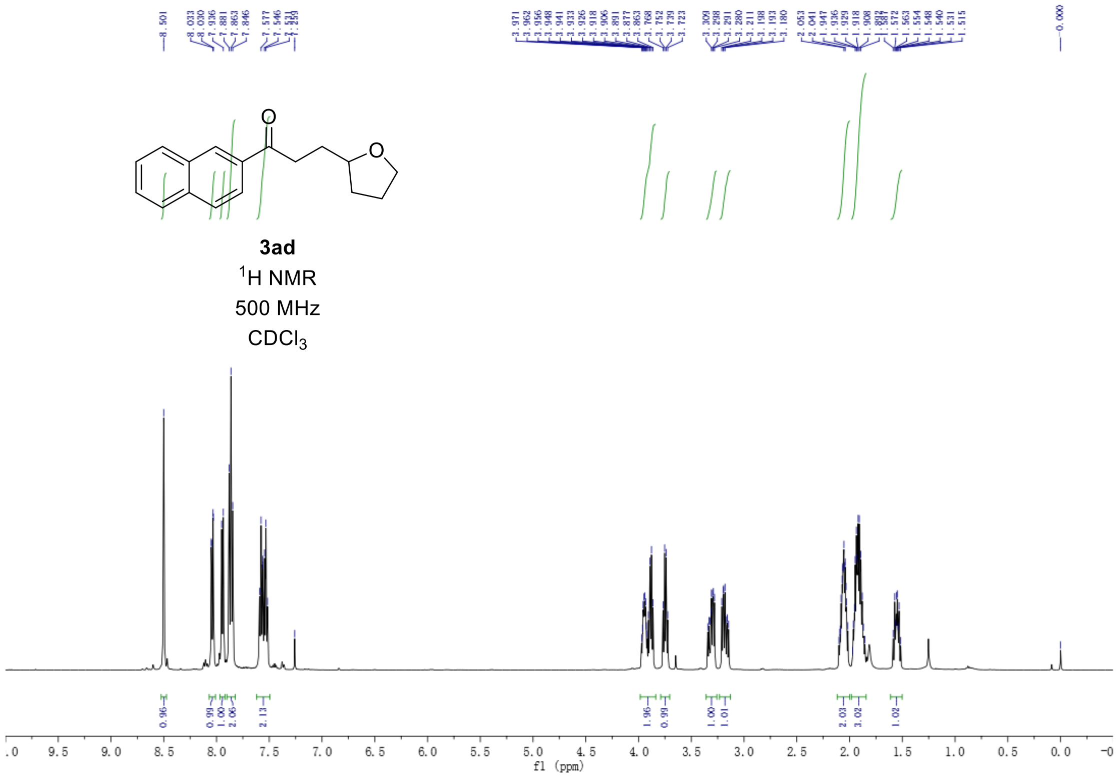

Supplementary Fig. 124 ^{1}H NMR spectrum of compound **3ad**

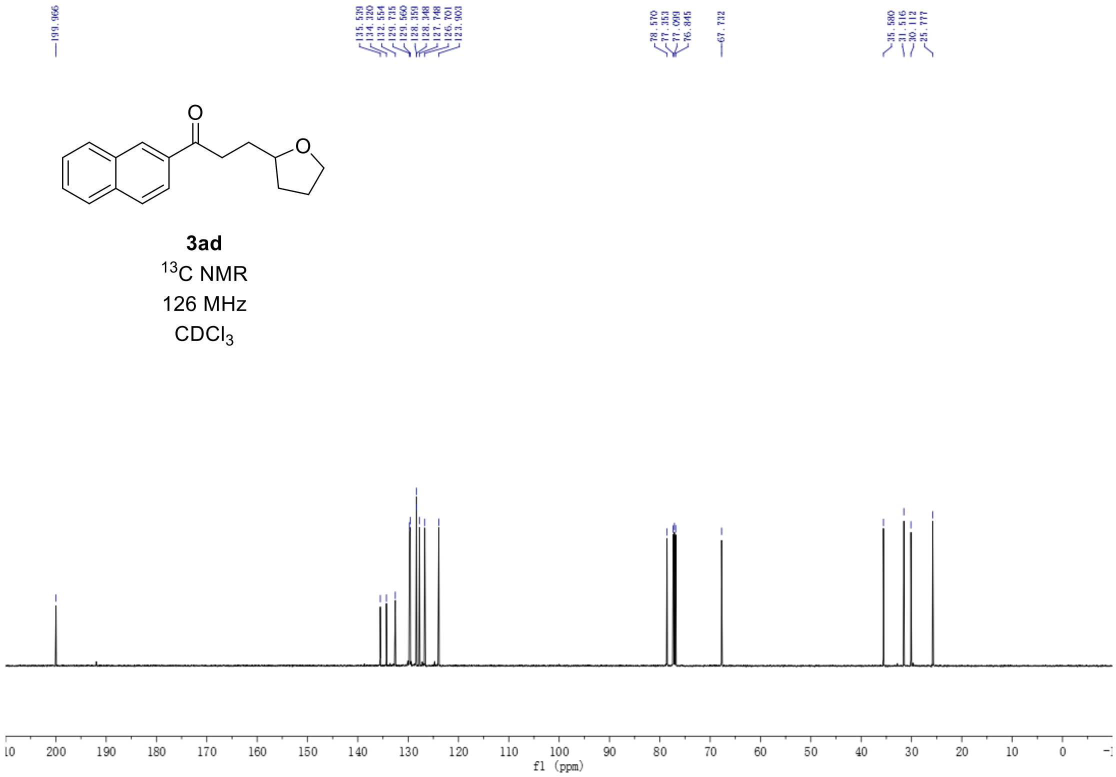

Supplementary Fig. 125 ^{13}C NMR spectrum of compound **3ad**

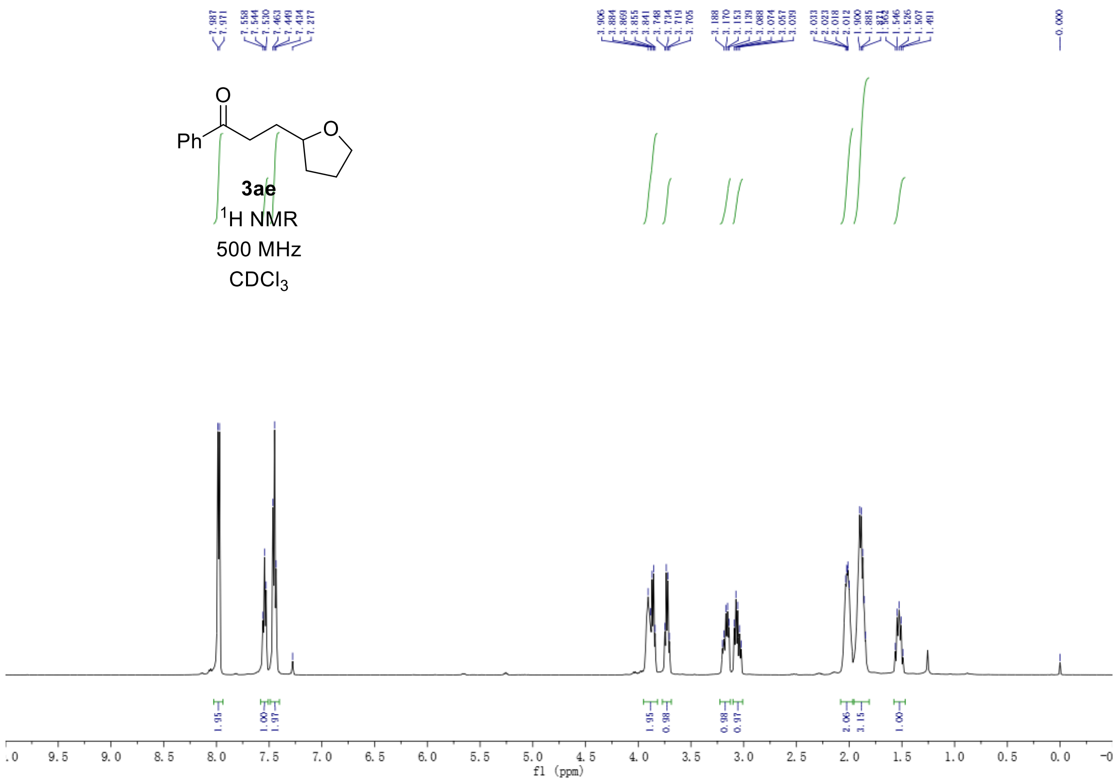

Supplementary Fig. 126 ^{1}H NMR spectrum of compound **3ae**

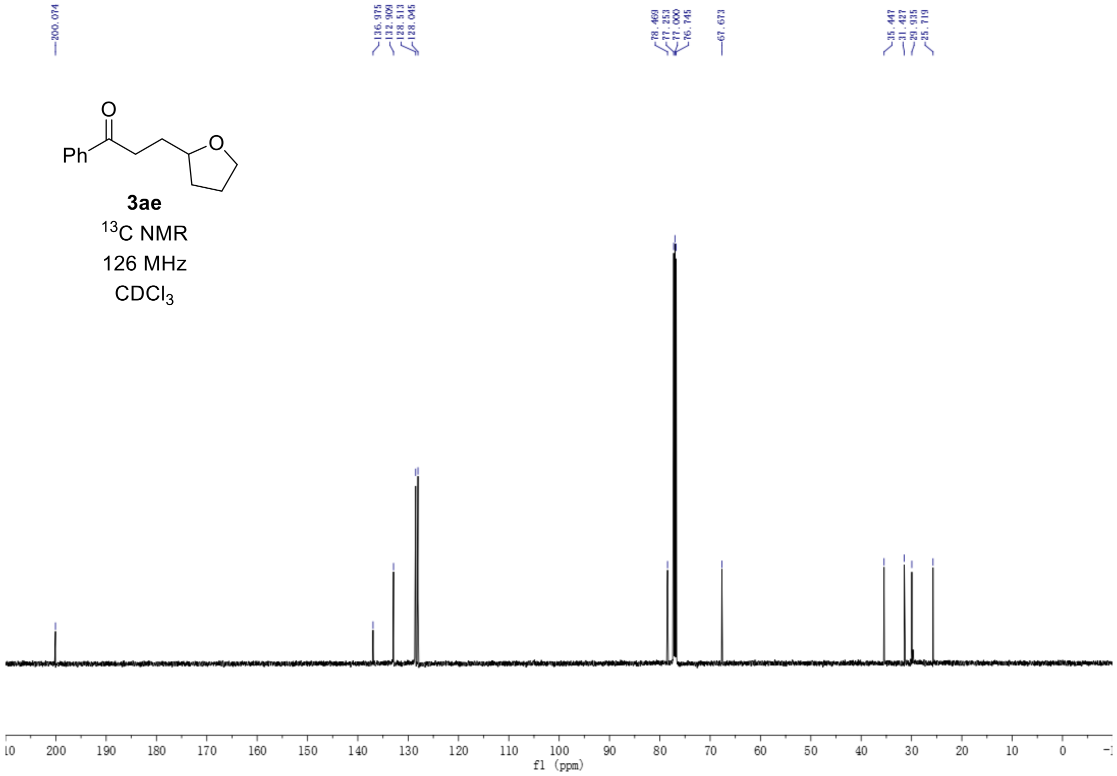

Supplementary Fig. 127 ^{13}C NMR spectrum of compound **3ae**

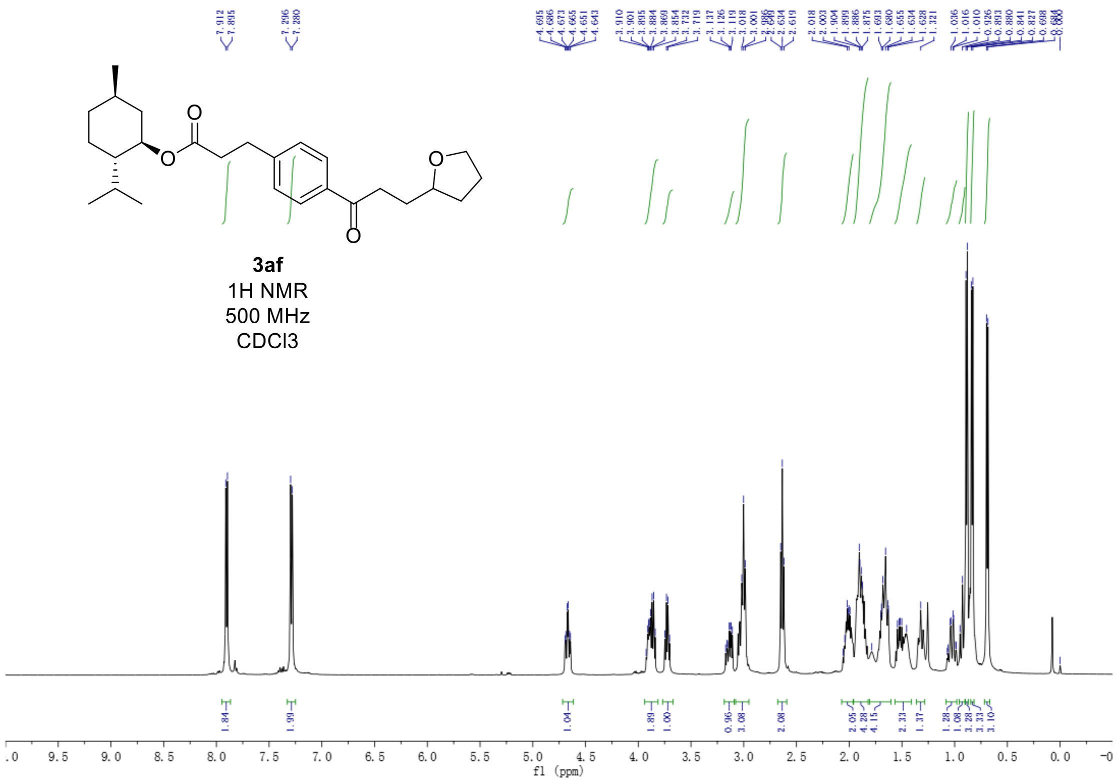

Supplementary Fig. 128 ^{1}H NMR spectrum of compound **3af**

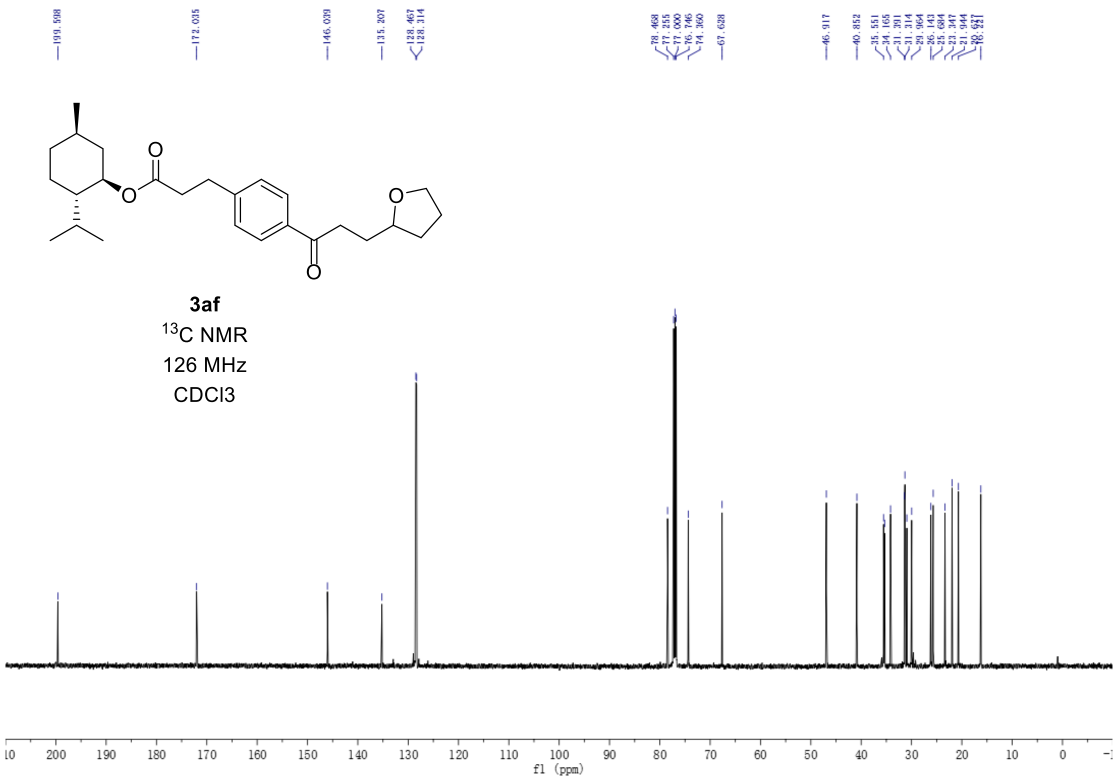

Supplementary Fig. 129 ^{13}C NMR spectrum of compound **3af**

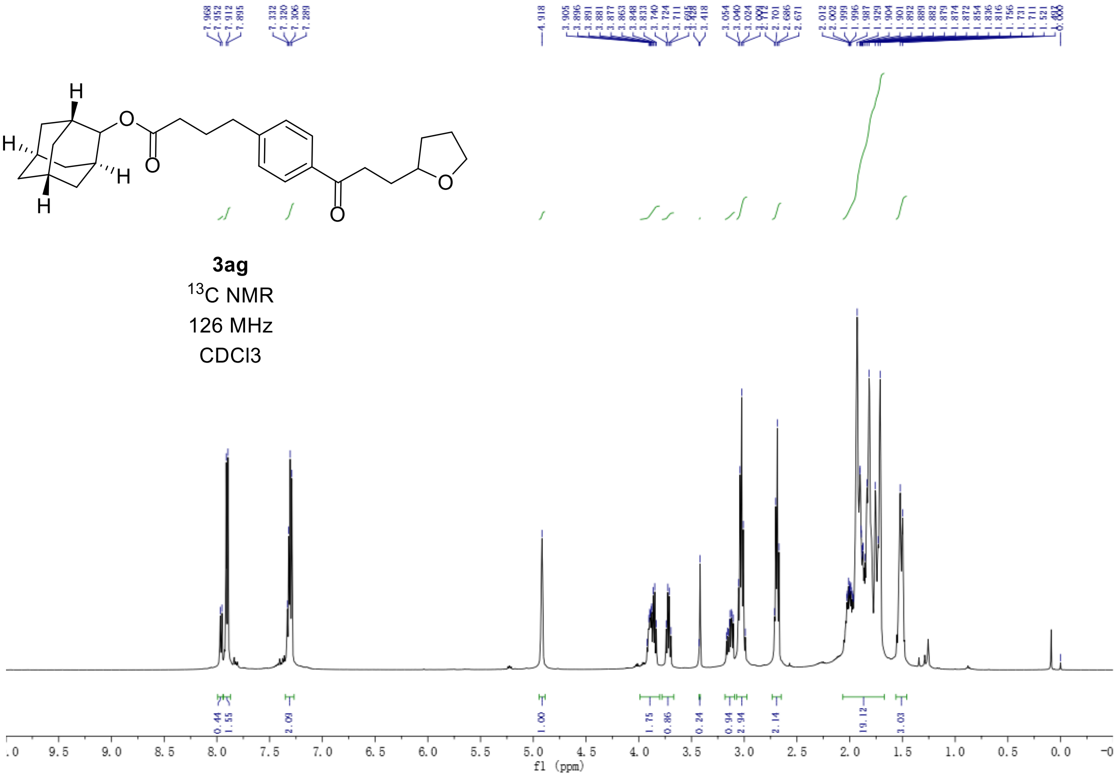

Supplementary Fig. 130 ^{1}H NMR spectrum of compound **3ag**

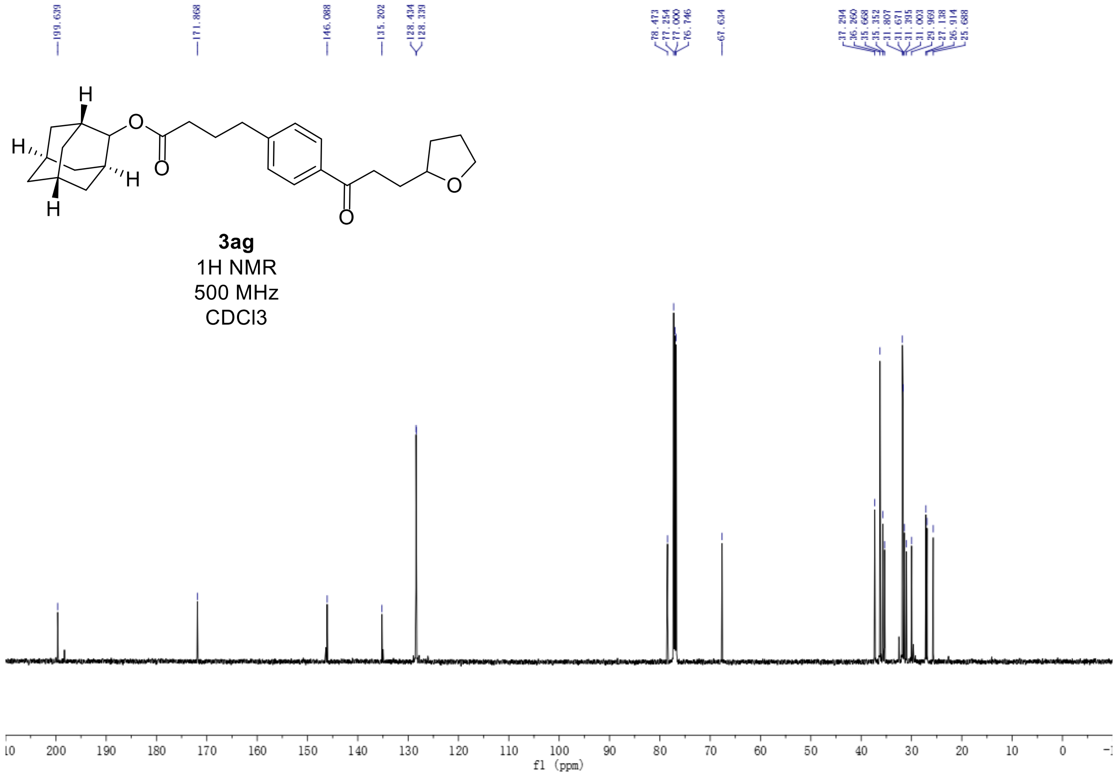

Supplementary Fig. 131 ^{13}C NMR spectrum of compound **3ag**

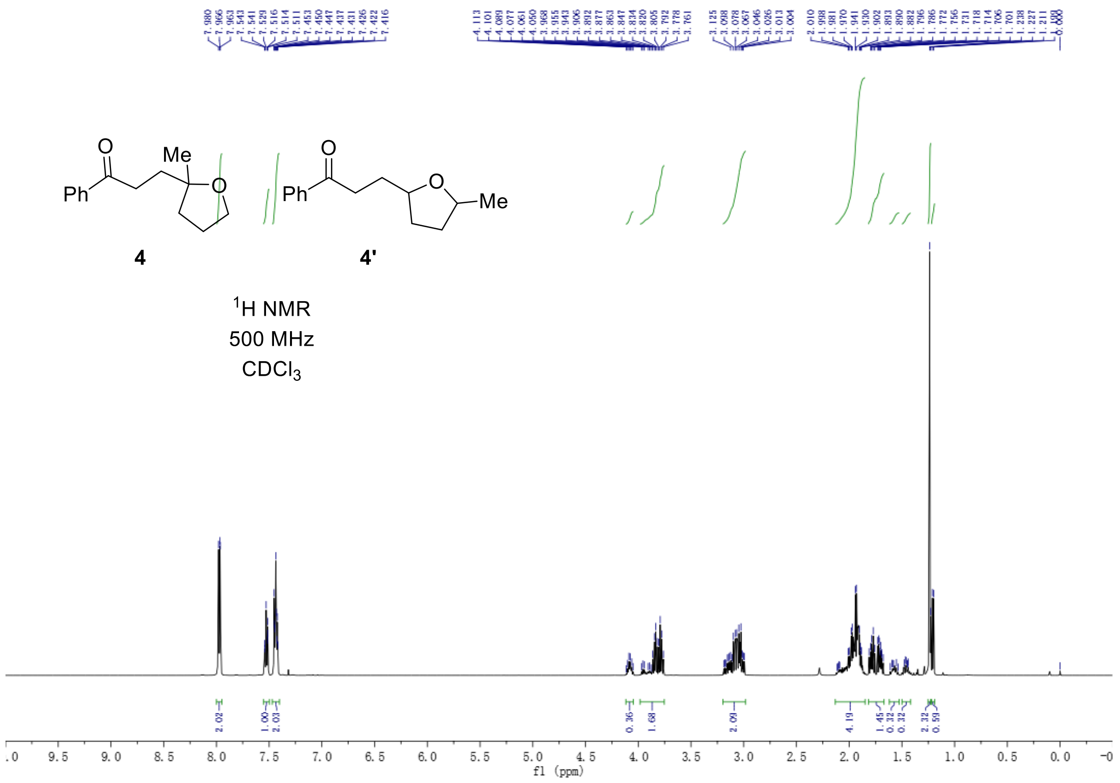

Supplementary Fig. 132 ^{1}H NMR spectrum of mixture of **4** and **4'**

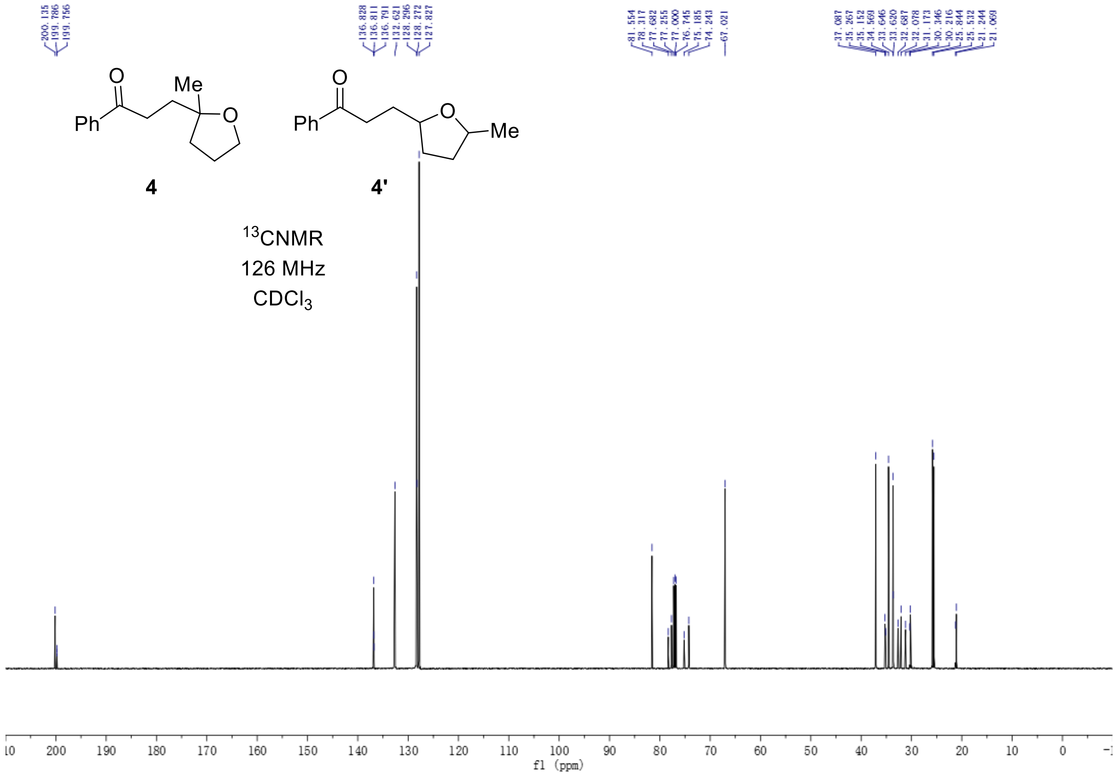

Supplementary Fig. 133 ^{13}C NMR spectrum of mixture of **4** and **4'**

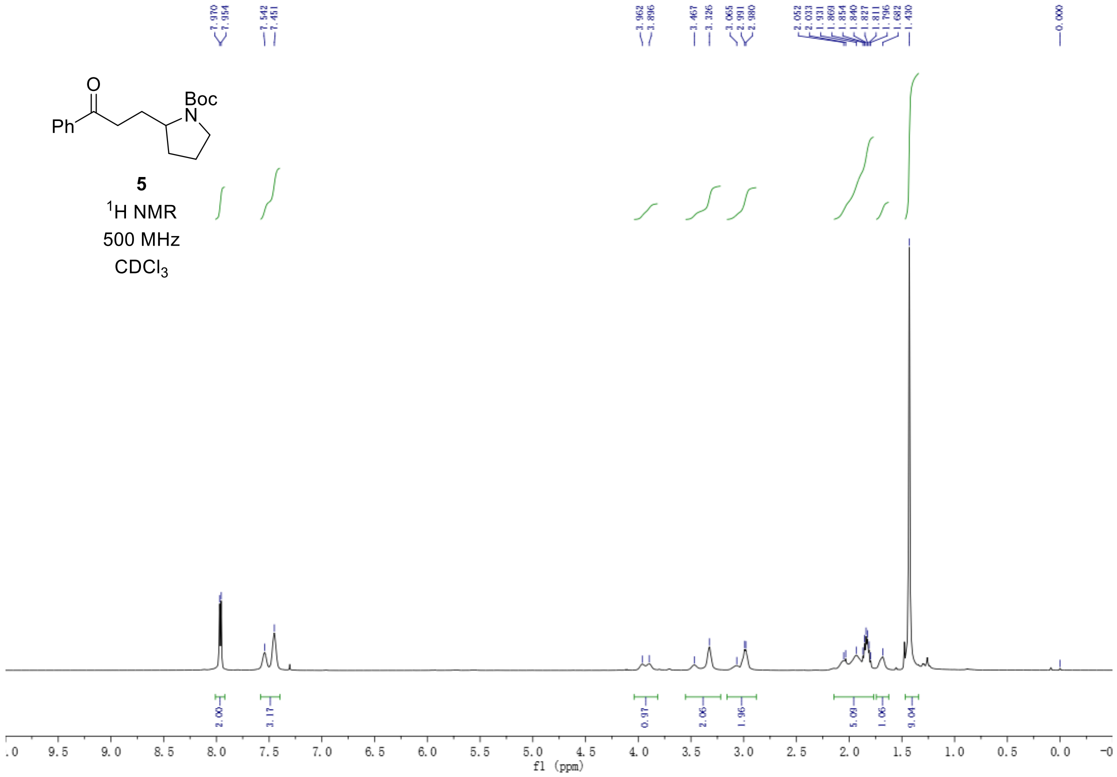

Supplementary Fig. 134 ^{1}H NMR spectrum of compound **5**

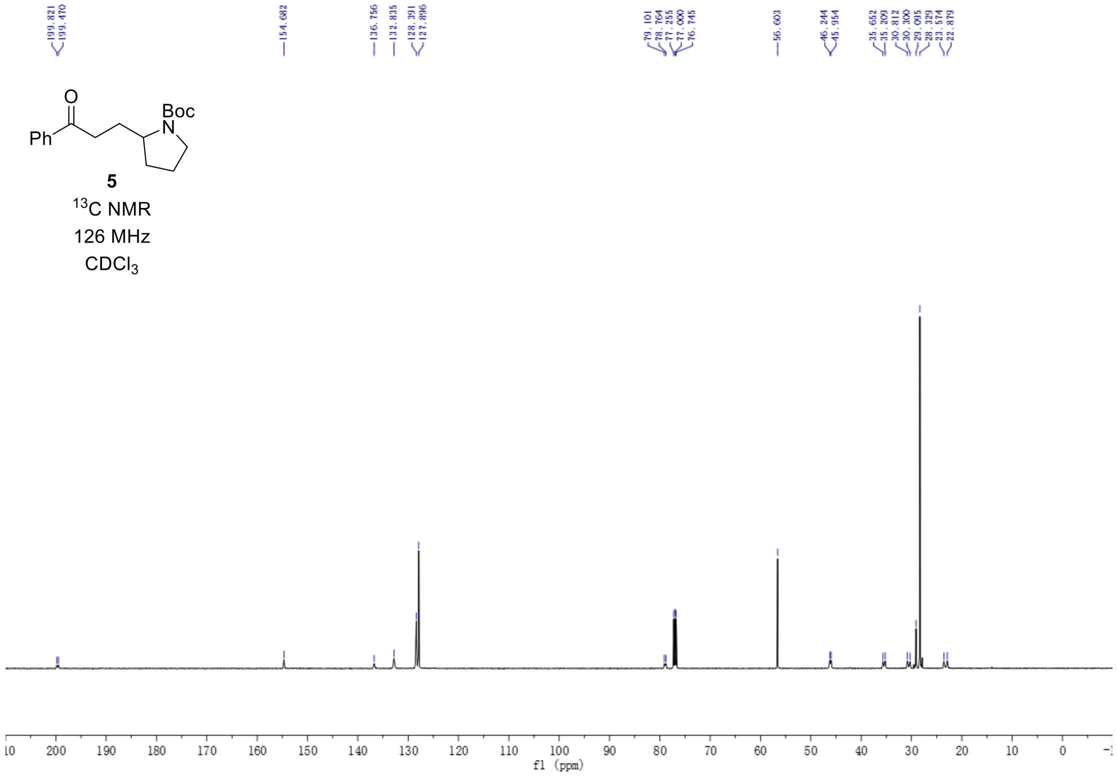

Supplementary Fig. 135 ^{13}C NMR spectrum of compound **5**

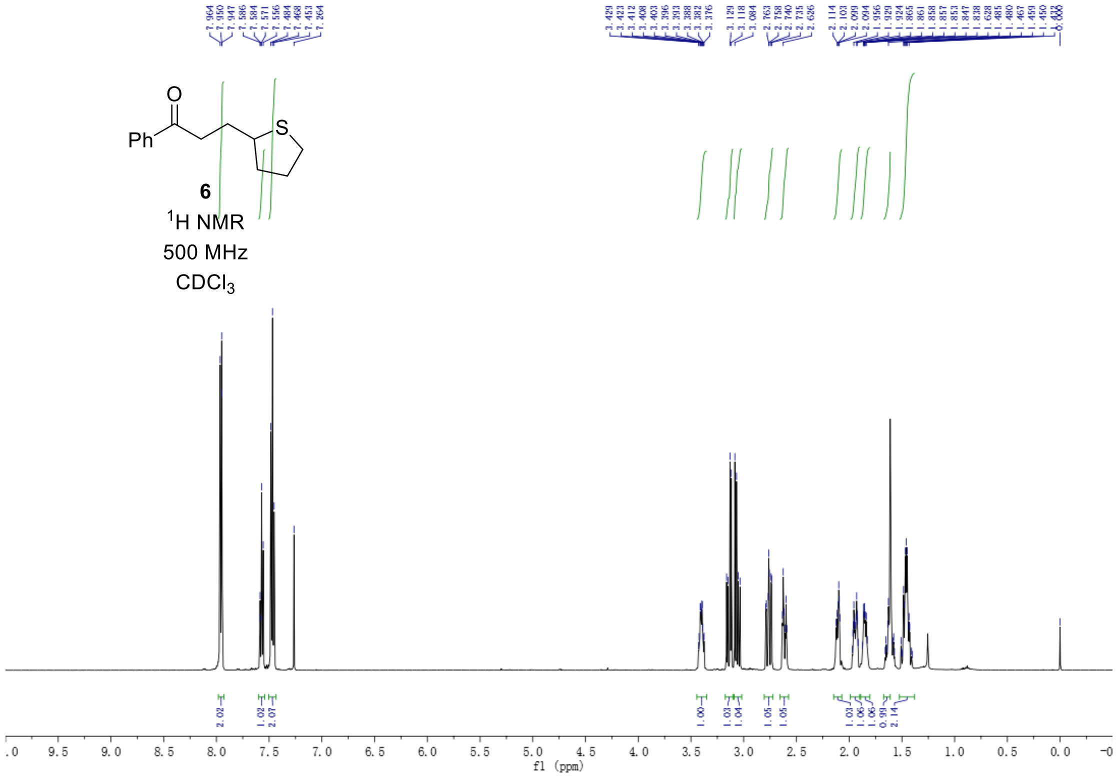

Supplementary Fig. 136 ^{1}H NMR spectrum of compound **6**

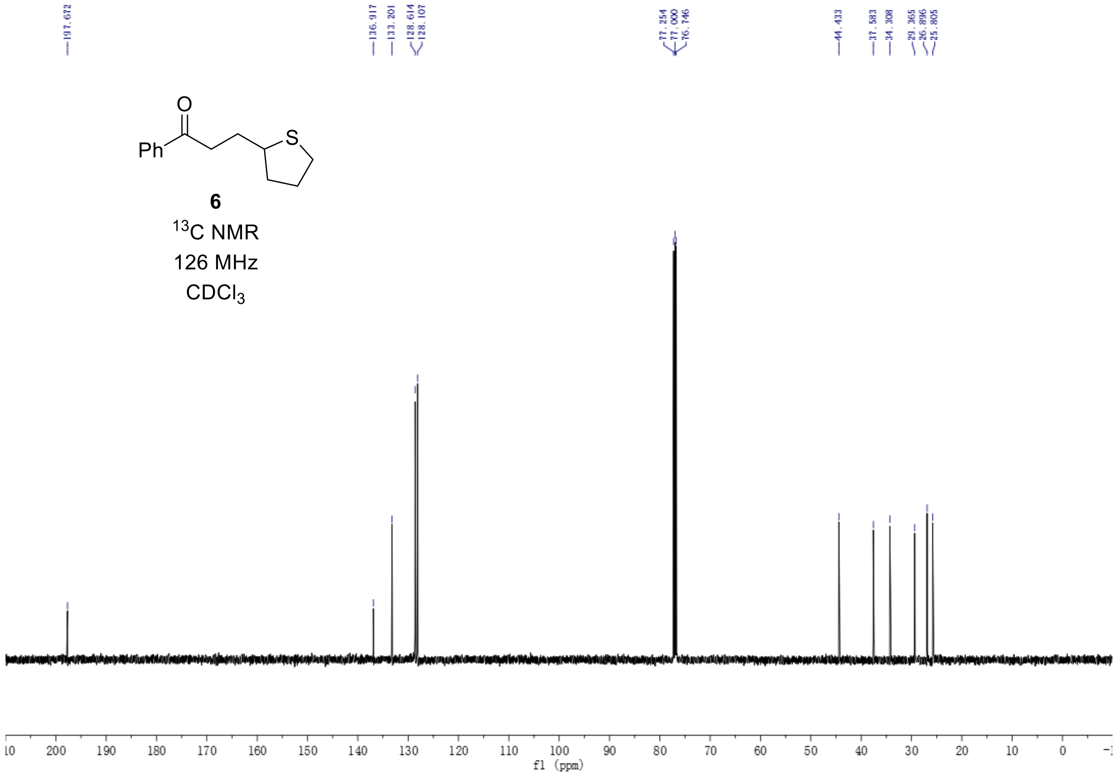

Supplementary Fig. 137 ^{13}C NMR spectrum of compound **6**

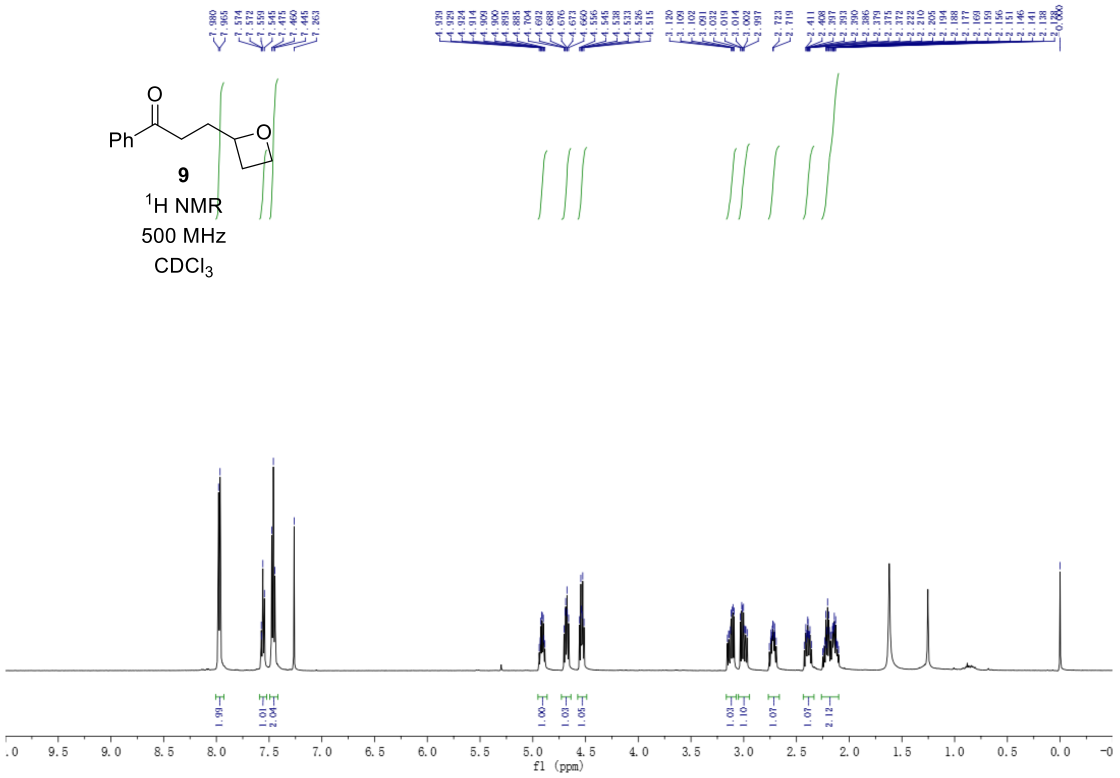

Supplementary Fig. 138 ^{1}H NMR spectrum of compound **9**

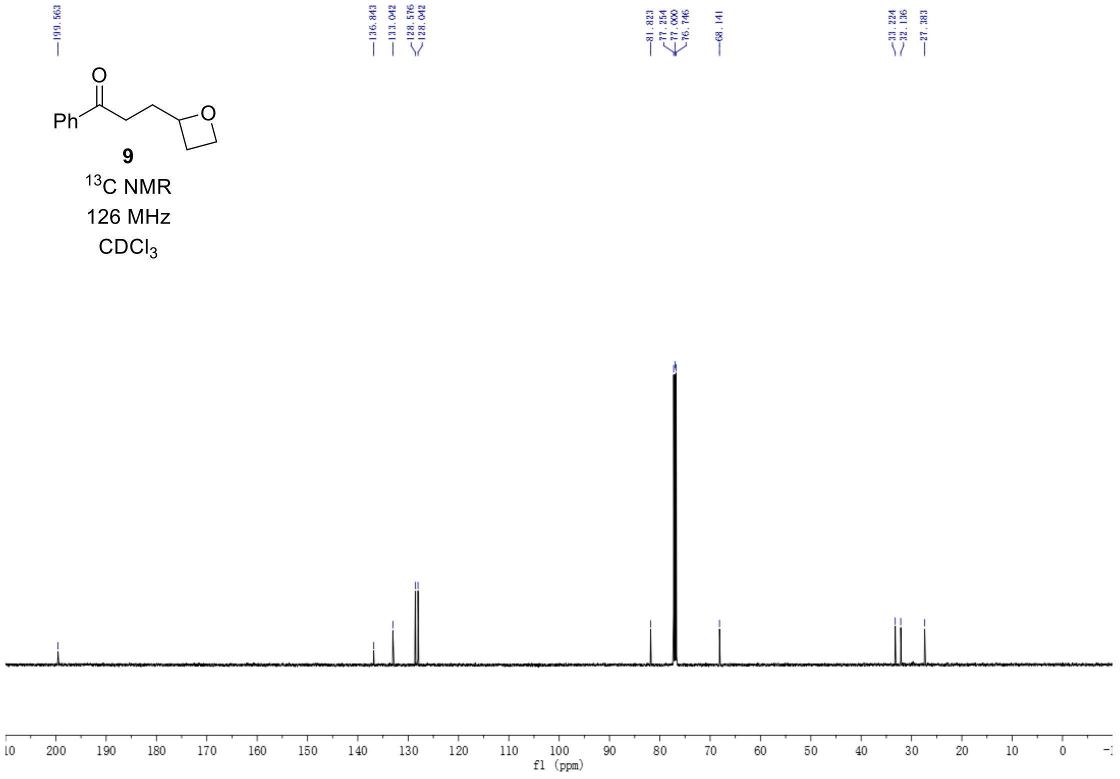

Supplementary Fig. 139 ^{1}H NMR spectrum of compound **9**

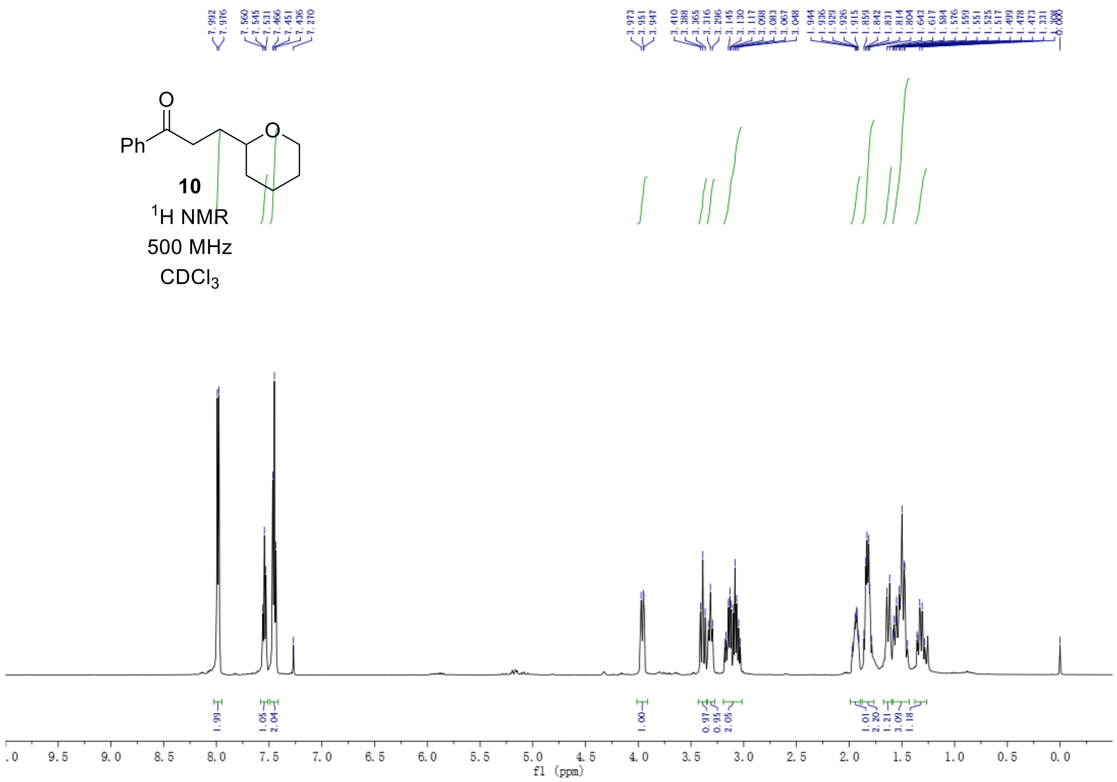

Supplementary Fig. 140 ^{1}H NMR spectrum of compound **10**

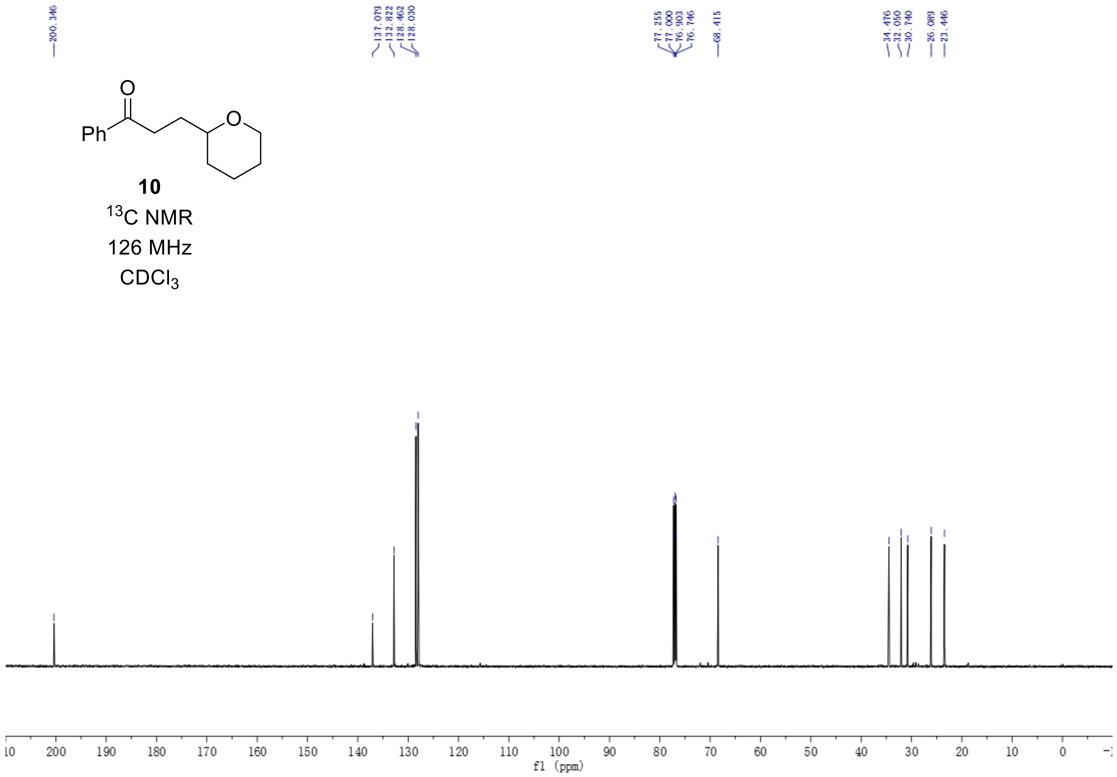

Supplementary Fig. 141 ^{13}C NMR spectrum of compound **10**

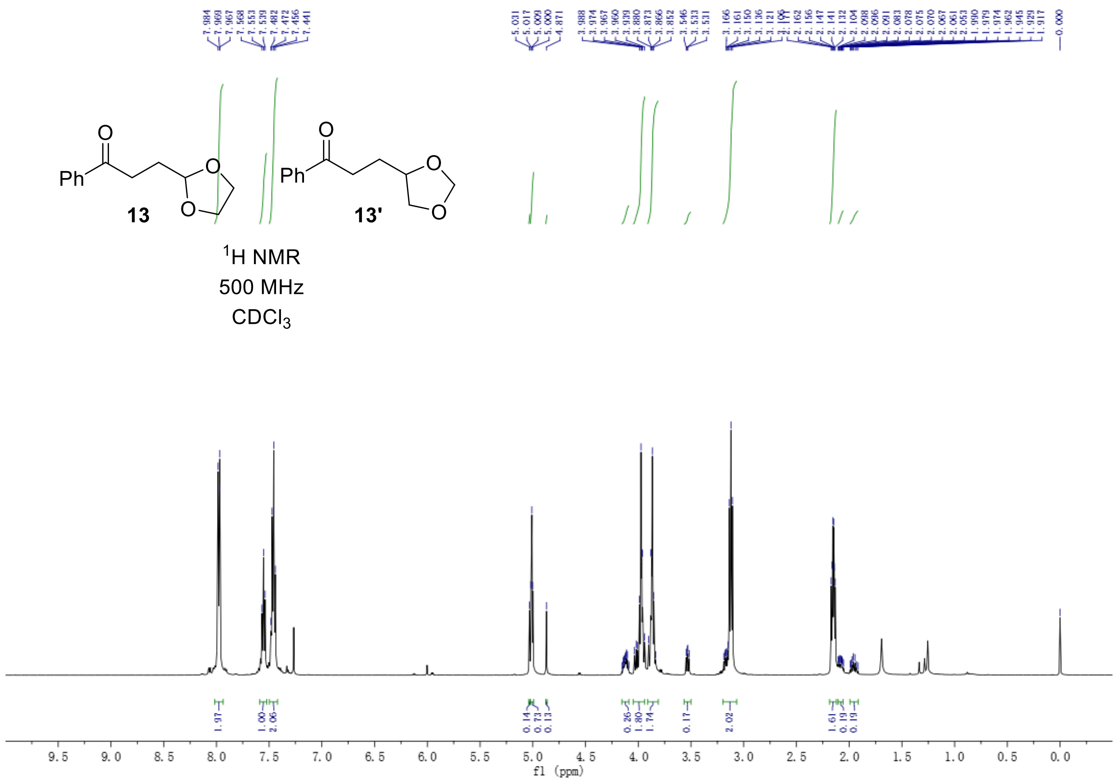

Supplementary Fig. 142 ^{1}H NMR spectrum of mixture of **13** and **13'**

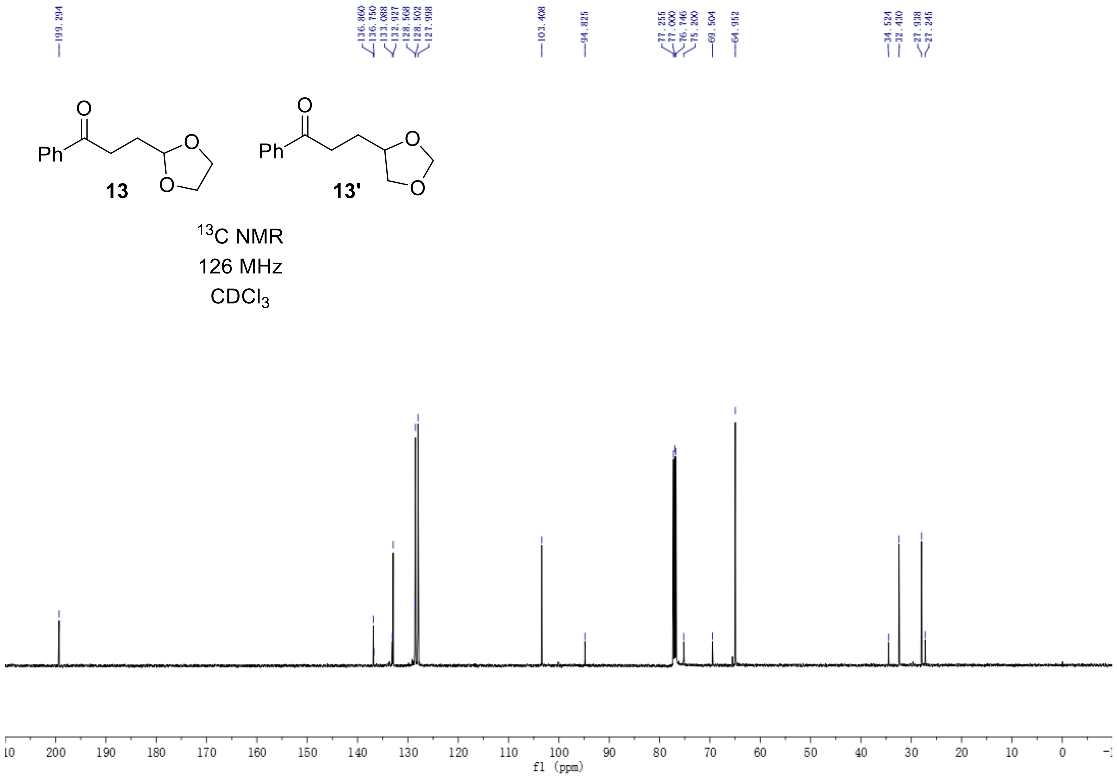

Supplementary Fig. 143 ^{13}C NMR spectrum of mixture of **13** and **13'**

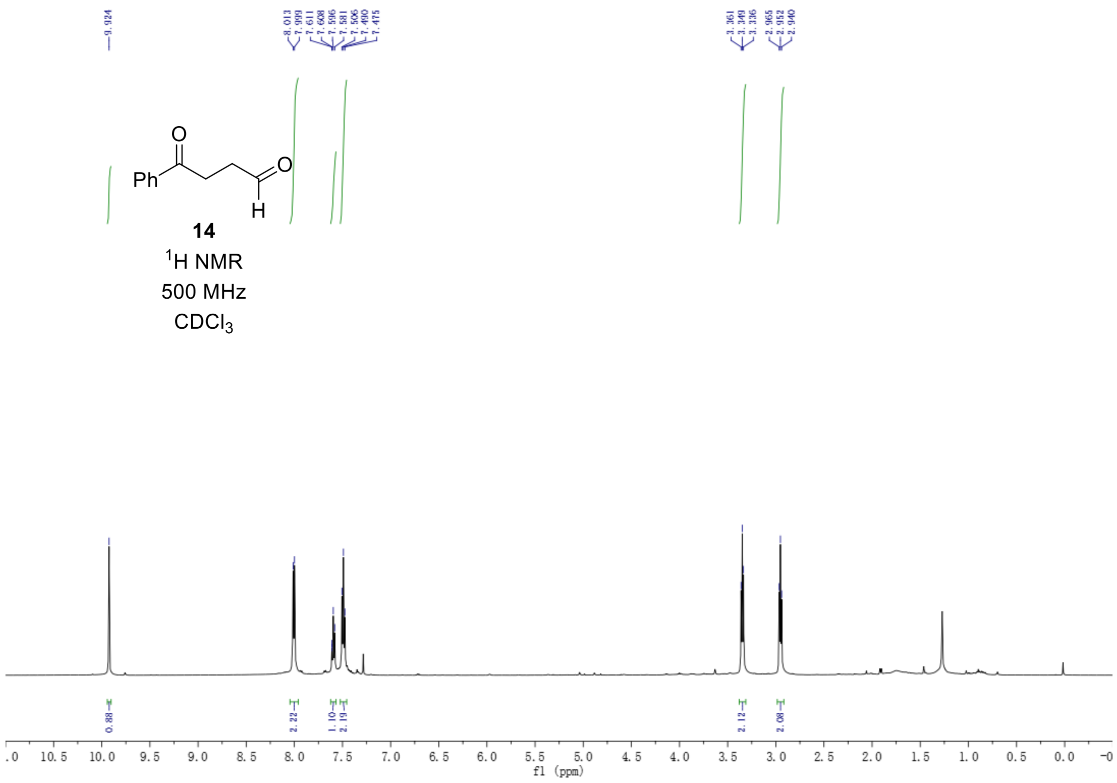

Supplementary Fig. 144 ^{1}H NMR spectrum of compound **14**

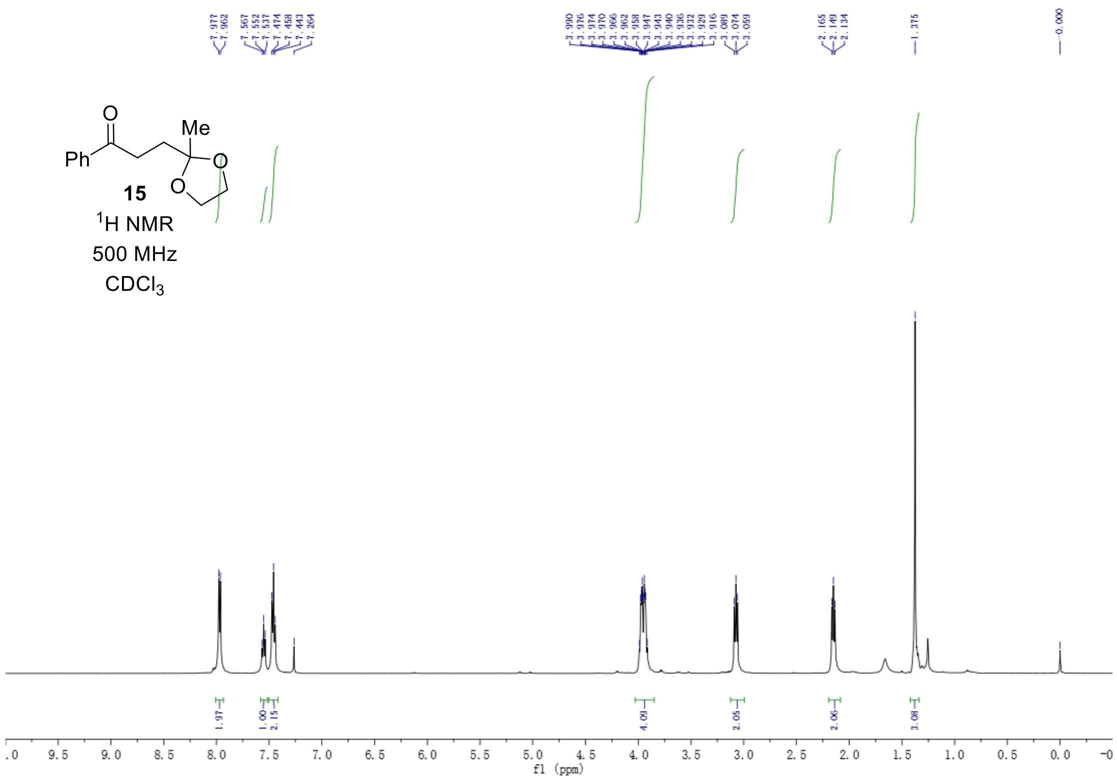

Supplementary Fig. 145 ^{1}H NMR spectrum of compound **15**

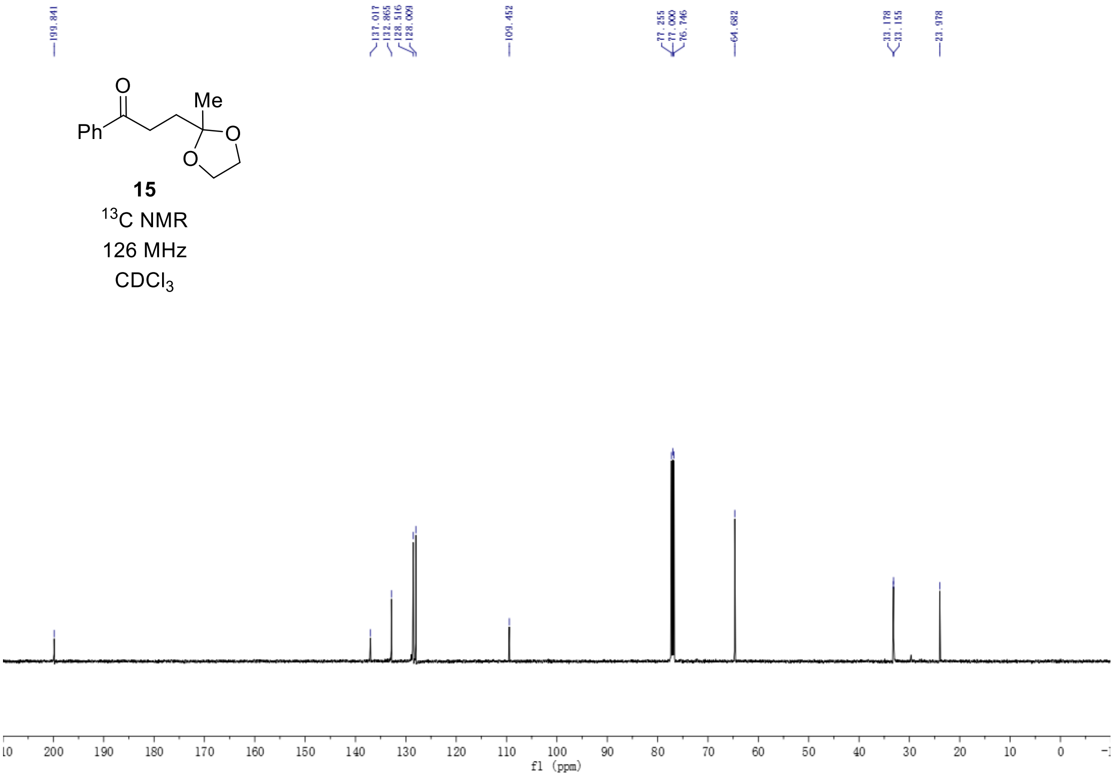

Supplementary Fig. 146 ^{13}C NMR spectrum of compound **15**

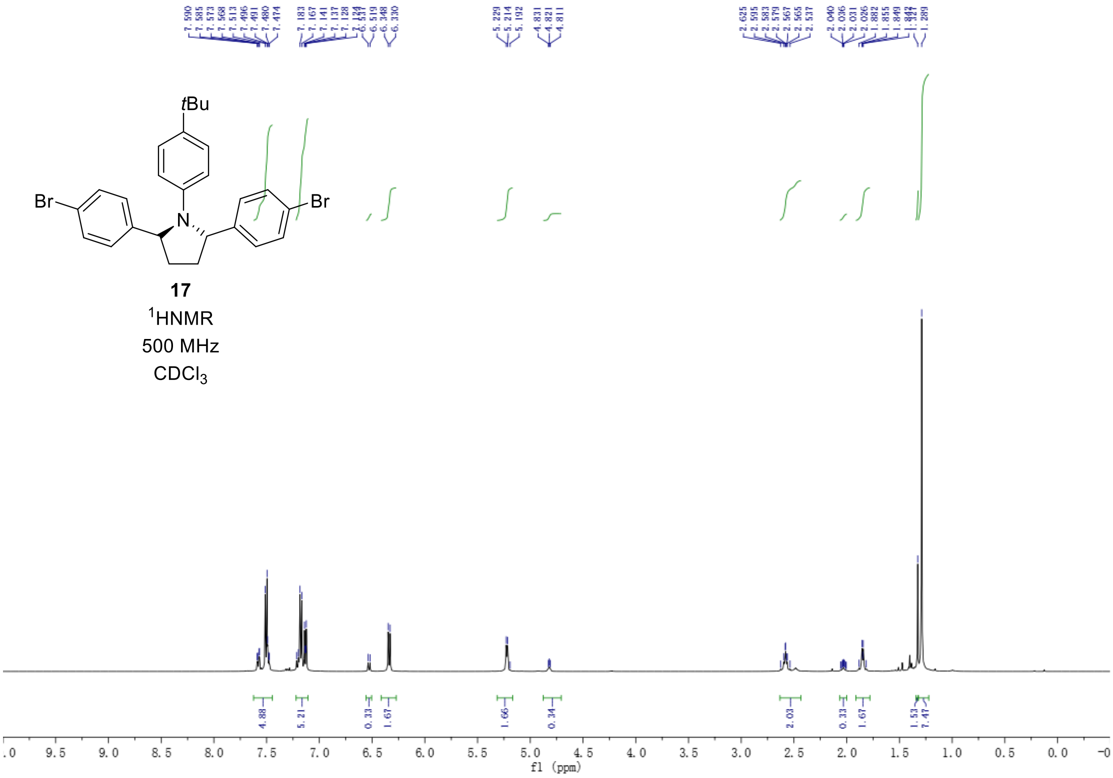

Supplementary Fig. 147 ^{1}H NMR spectrum of compound **17**

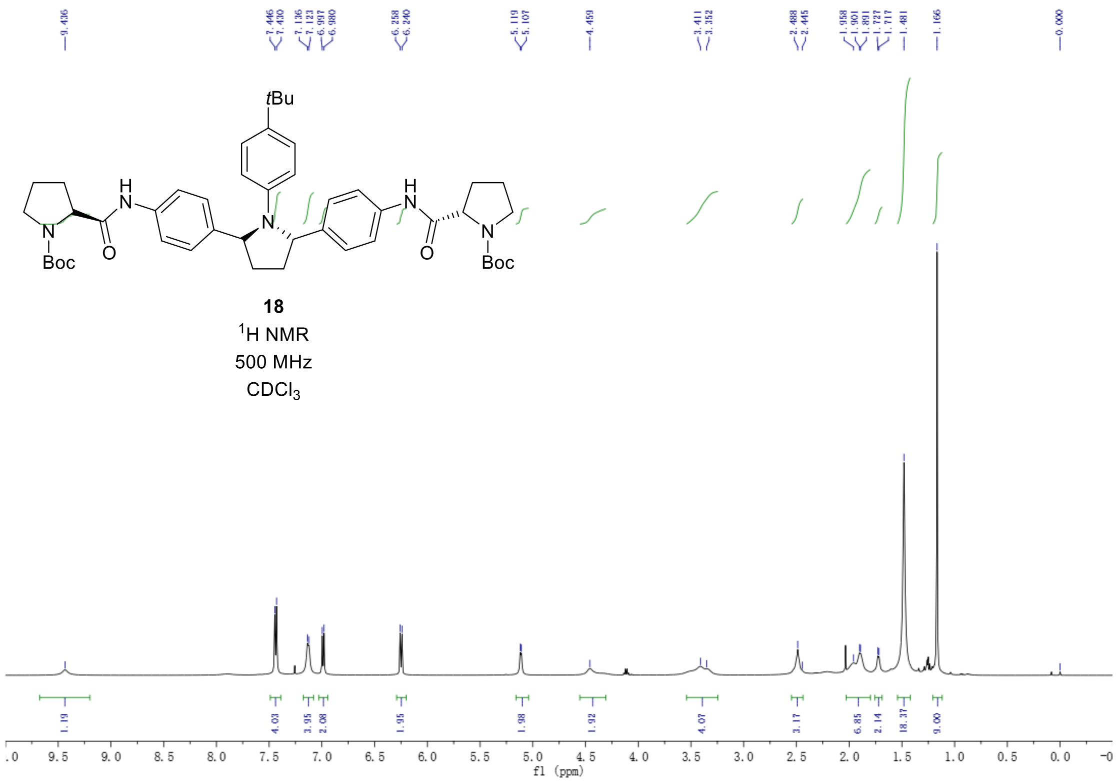

Supplementary Fig. 148 ^{1}H NMR spectrum of compound **18**

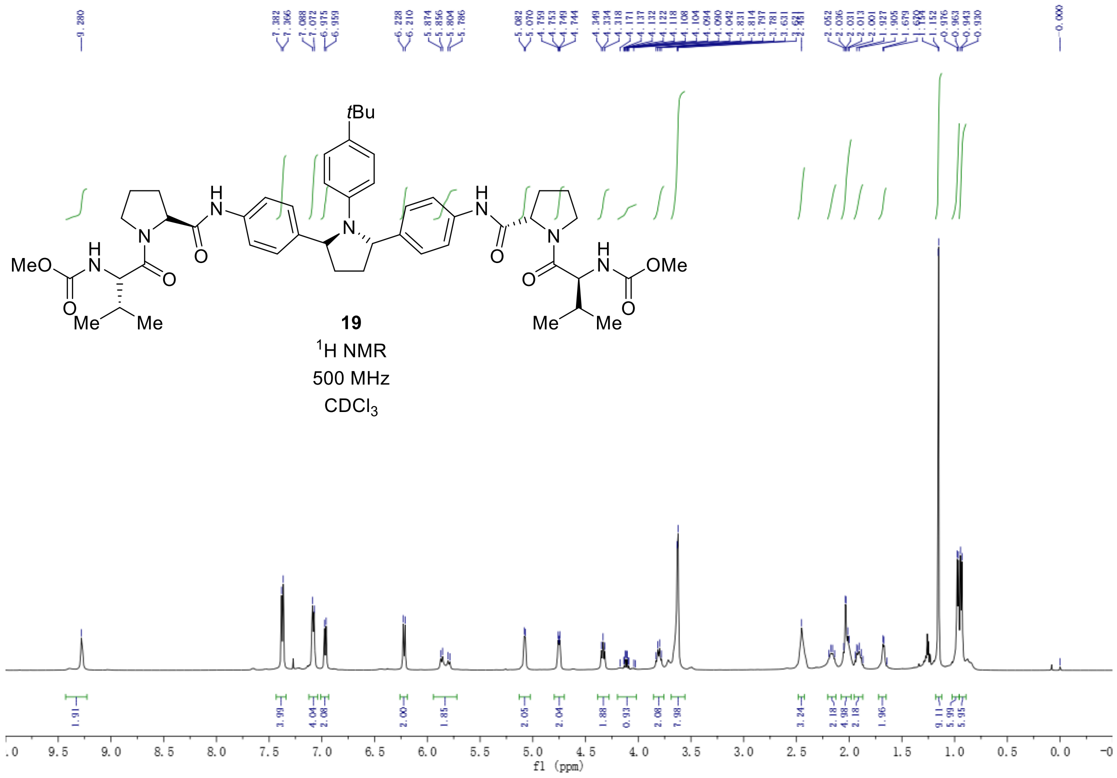

Supplementary Fig. 149 ^{1}H NMR spectrum of compound **19**

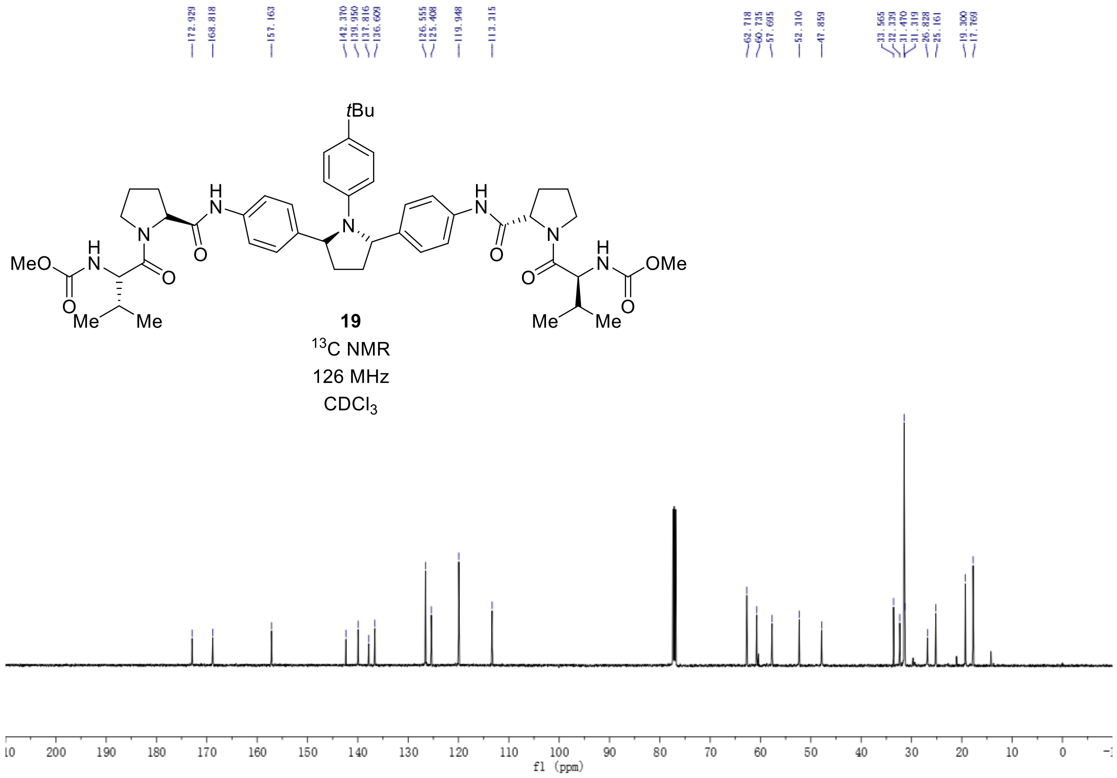

Supplementary Fig. 150 ^{13}C NMR spectrum of compound **19**

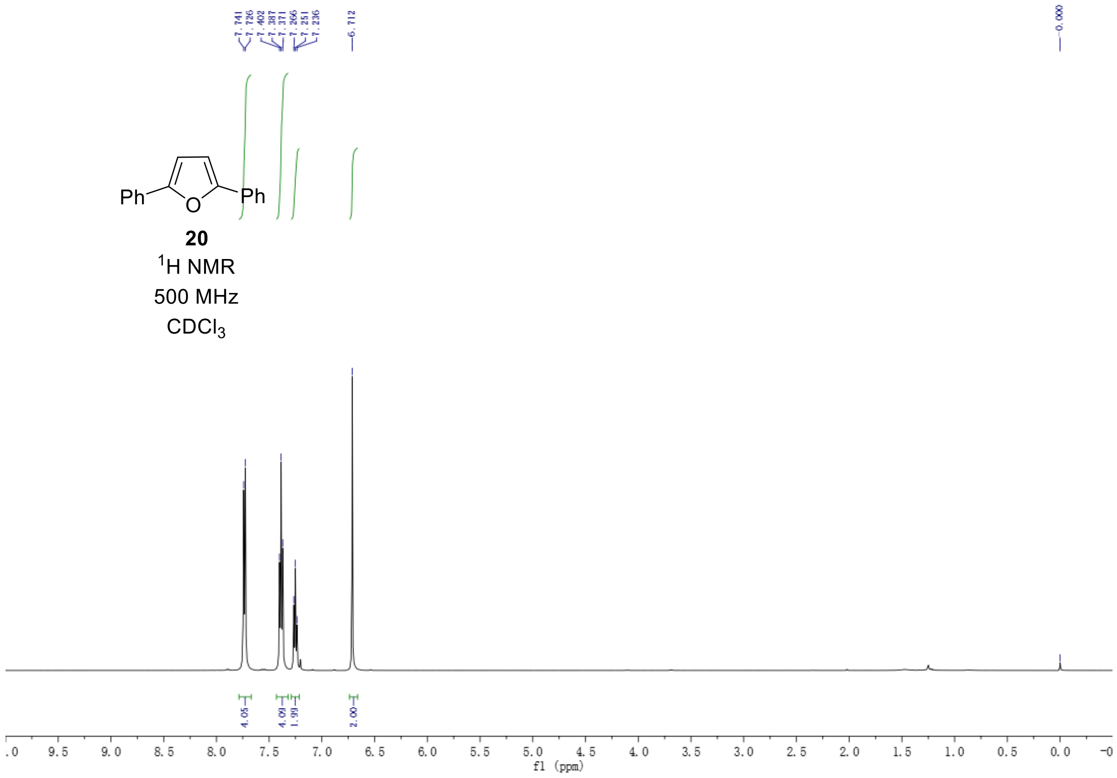

Supplementary Fig. 151 ^{1}H NMR spectrum of compound **20**

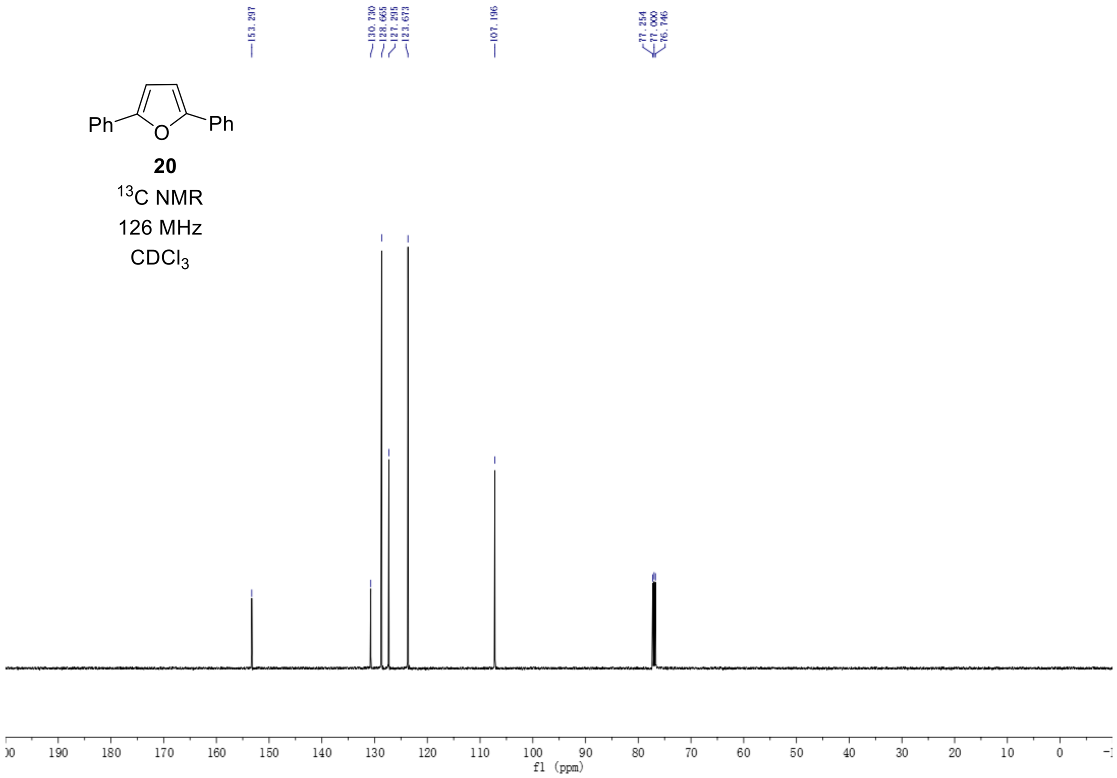

Supplementary Fig. 152 ^{13}C NMR spectrum of compound **20**

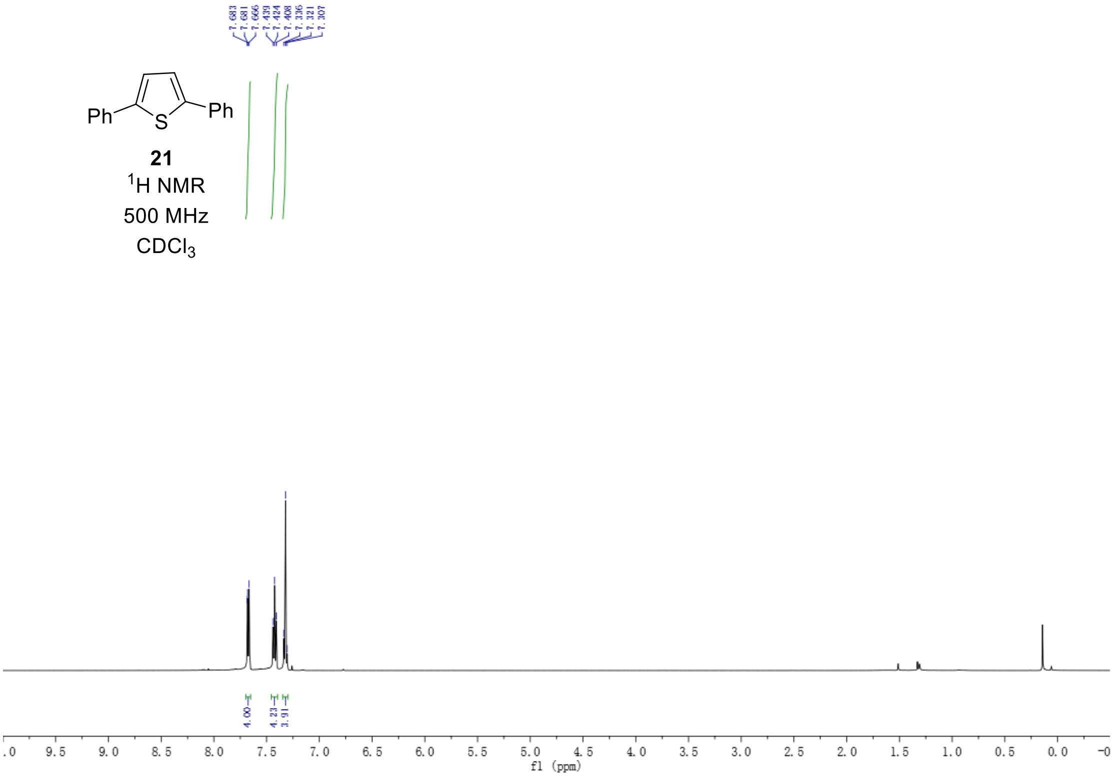

Supplementary Fig. 153 ^{1}H NMR spectrum of compound **21**

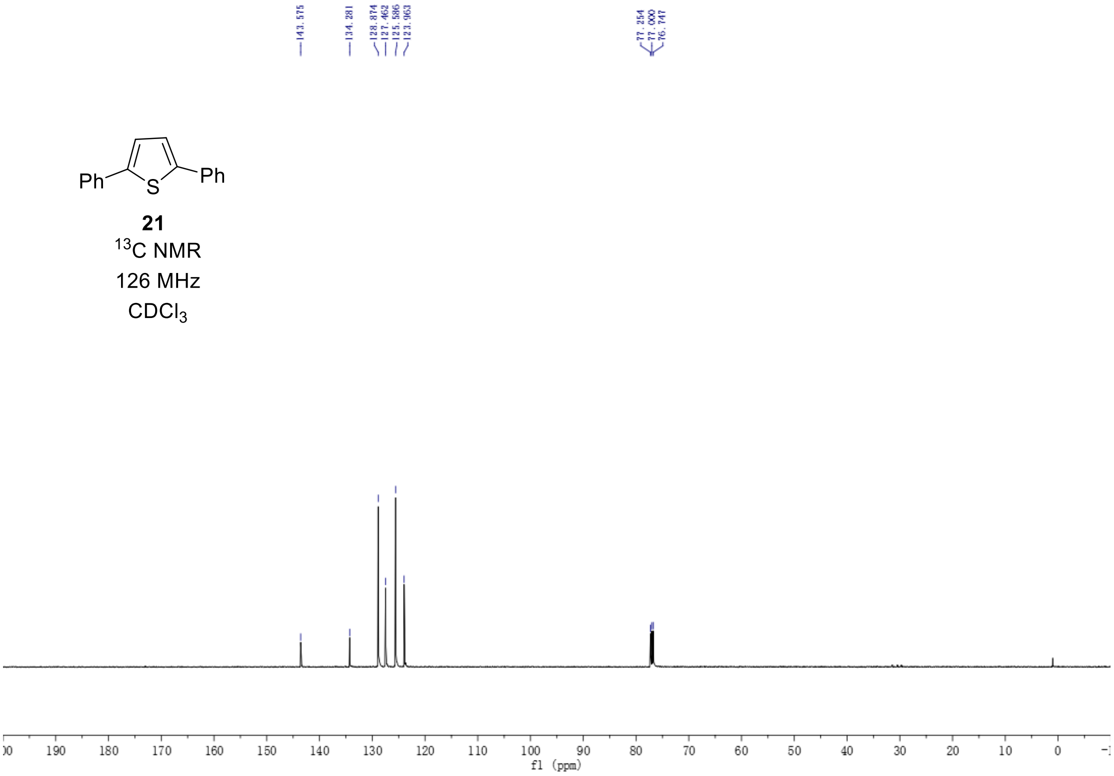

Supplementary Fig. 154 ^{13}C NMR spectrum of compound **21**

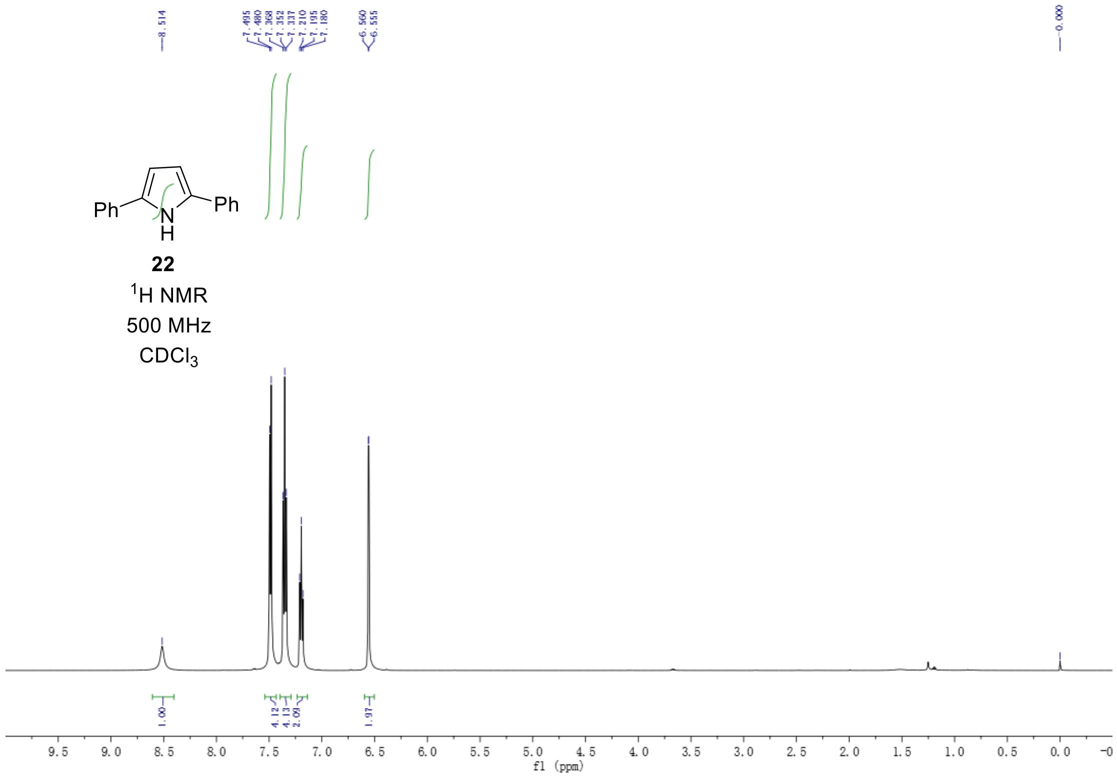

Supplementary Fig. 155 ^{1}H NMR spectrum of compound **22**

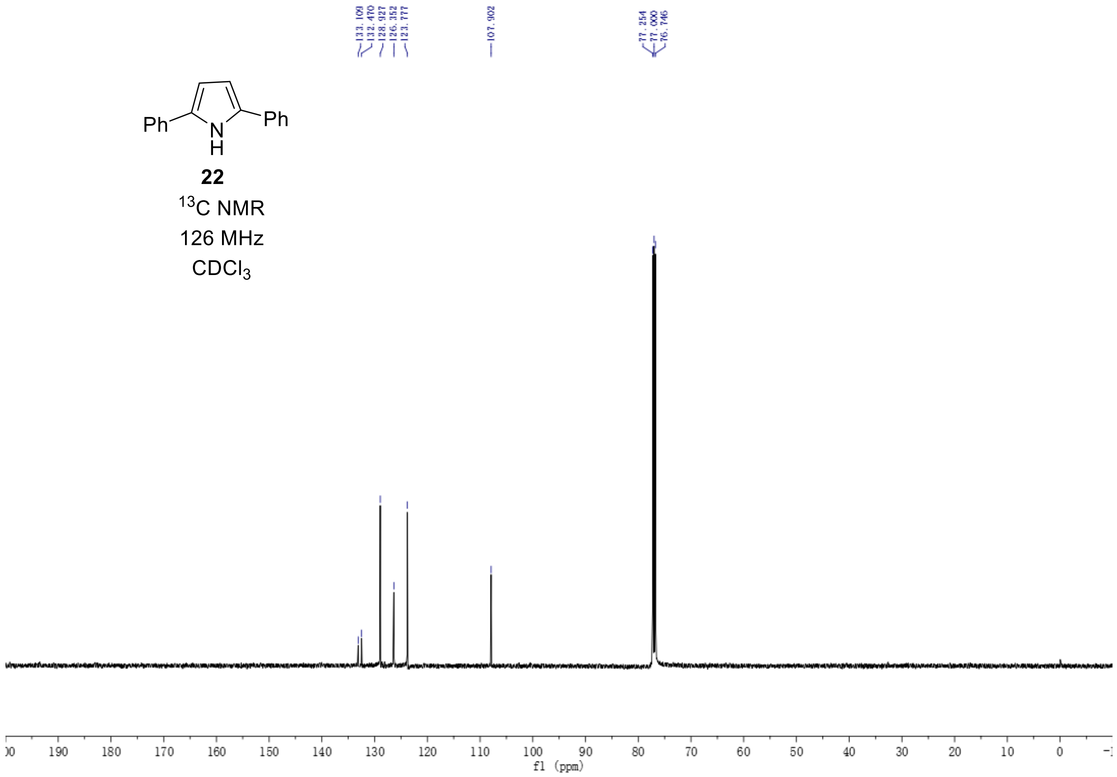

Supplementary Fig. 156 ^{13}C NMR spectrum of compound **22**

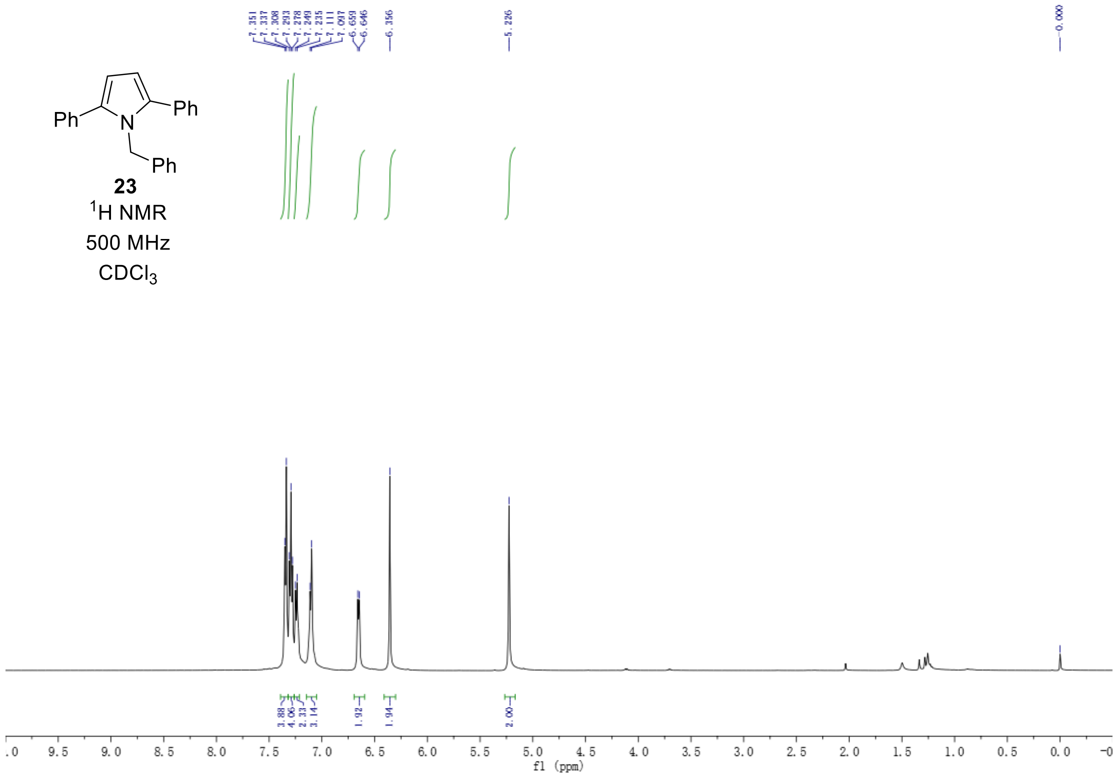

Supplementary Fig. 157 ^{1}H NMR spectrum of compound **23**

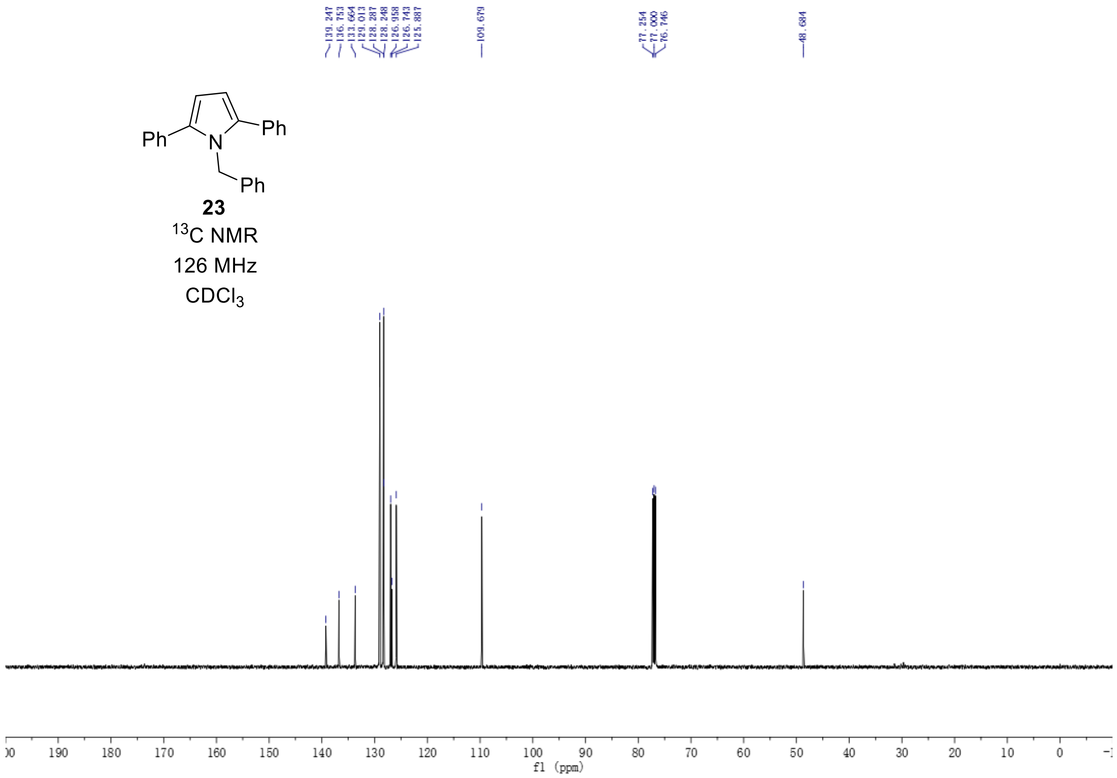

Supplementary Fig. 158 ^{13}C NMR spectrum of compound **23**

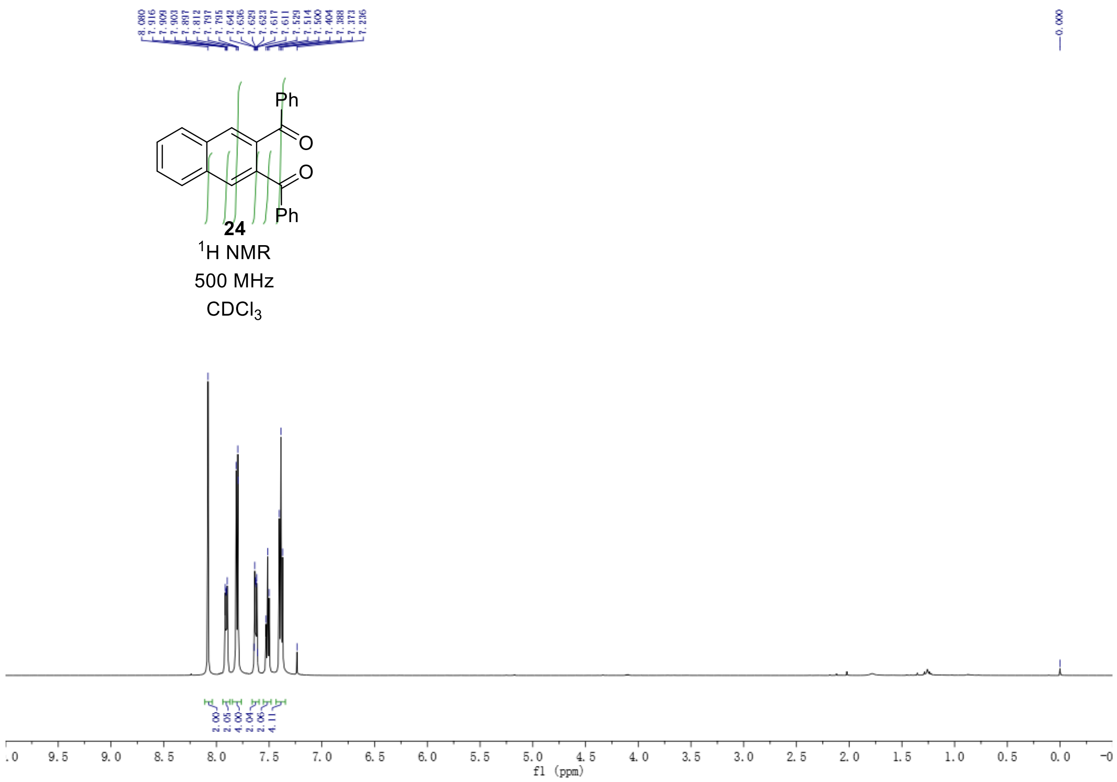

Supplementary Fig. 159 ^{1}H NMR spectrum of compound **24**

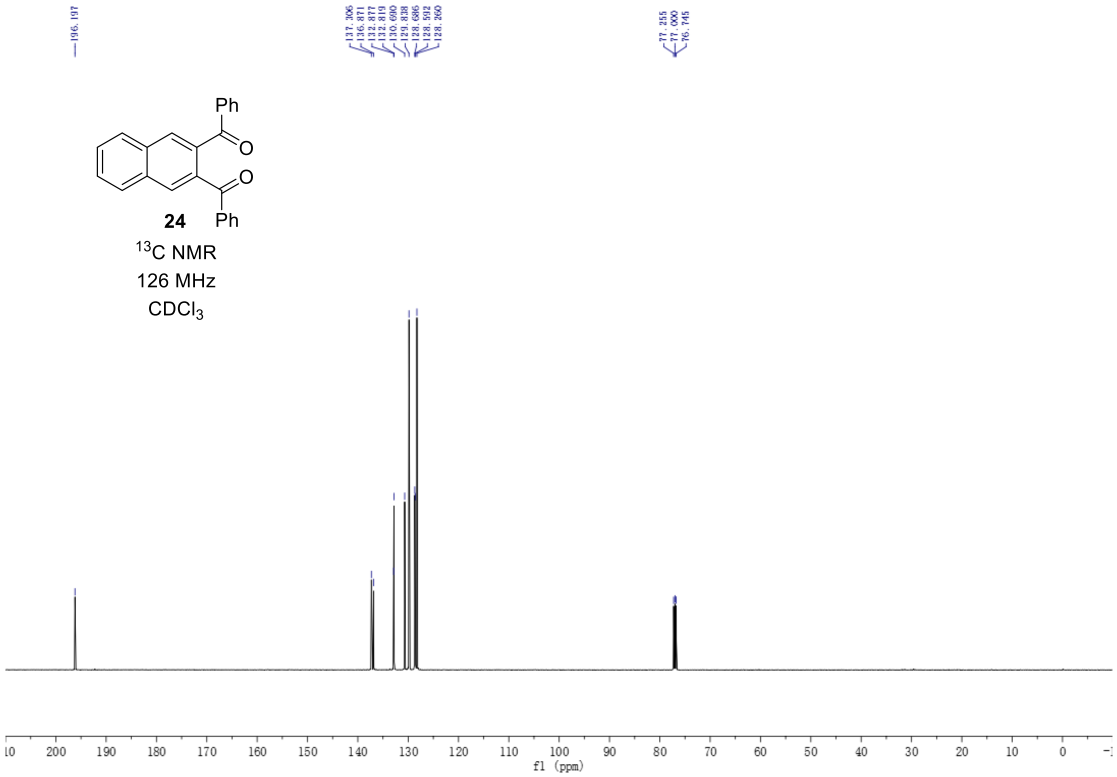

Supplementary Fig. 160 ^{13}C NMR spectrum of compound **24**

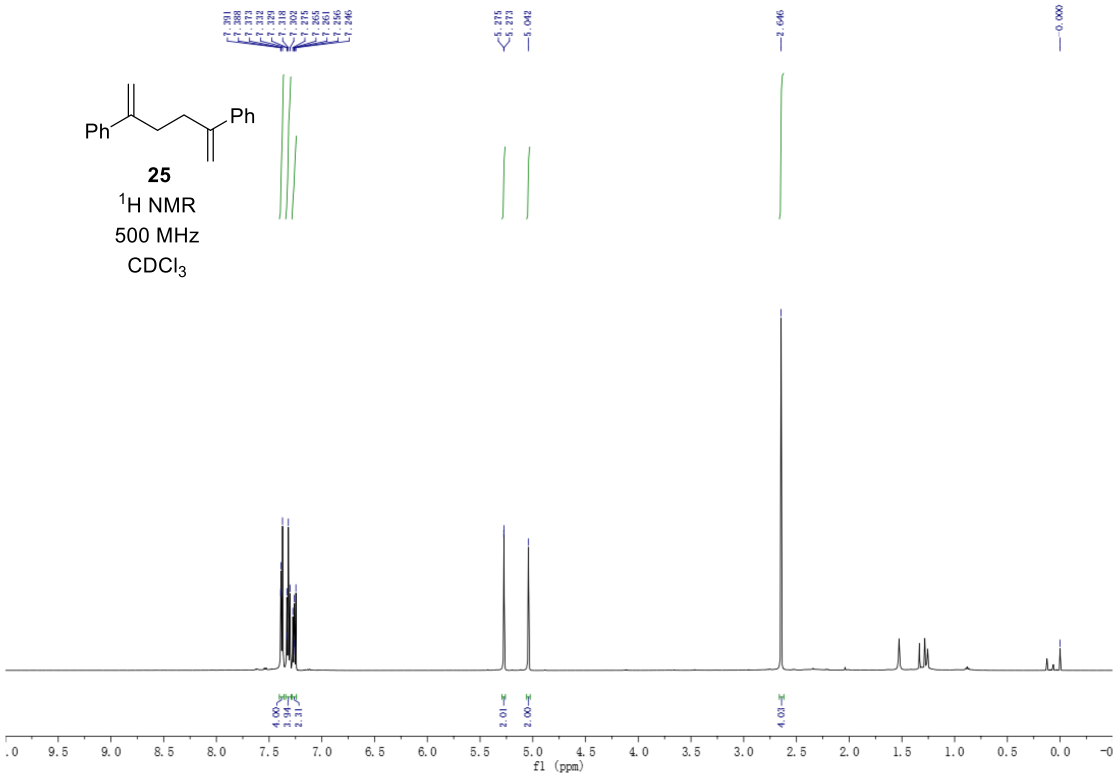

Supplementary Fig. 161 ^{1}H NMR spectrum of compound **25**

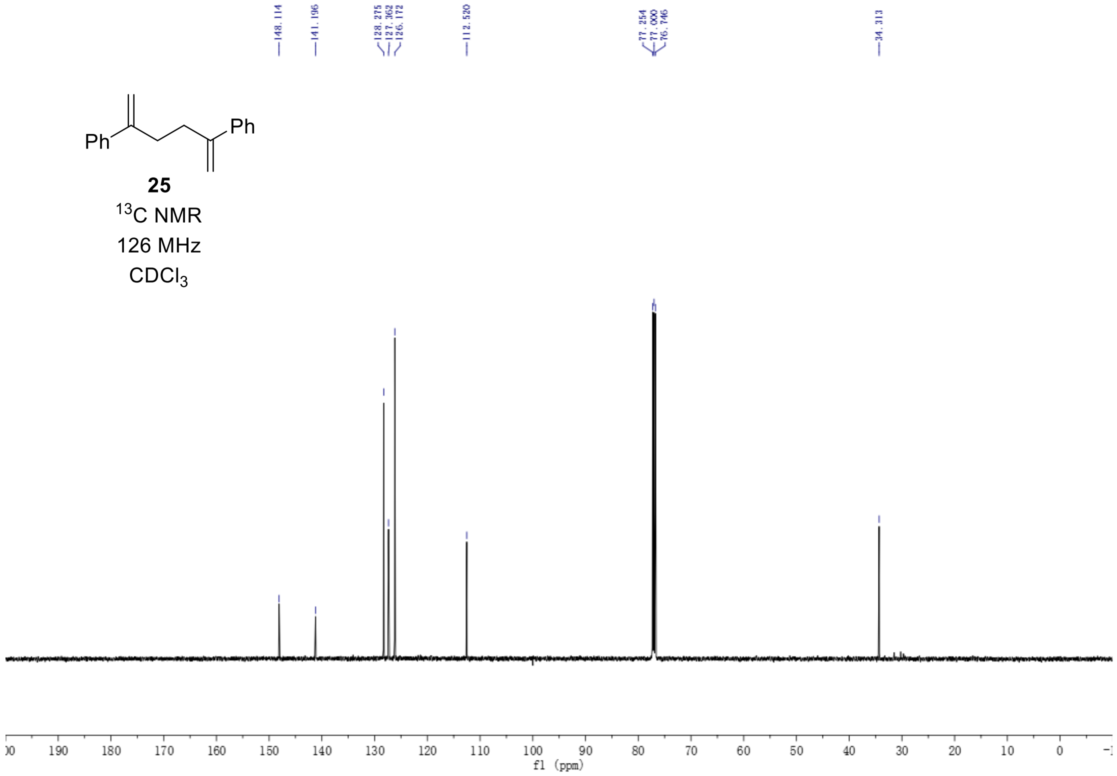

Supplementary Fig. 162 ^{13}C NMR spectrum of compound **25**

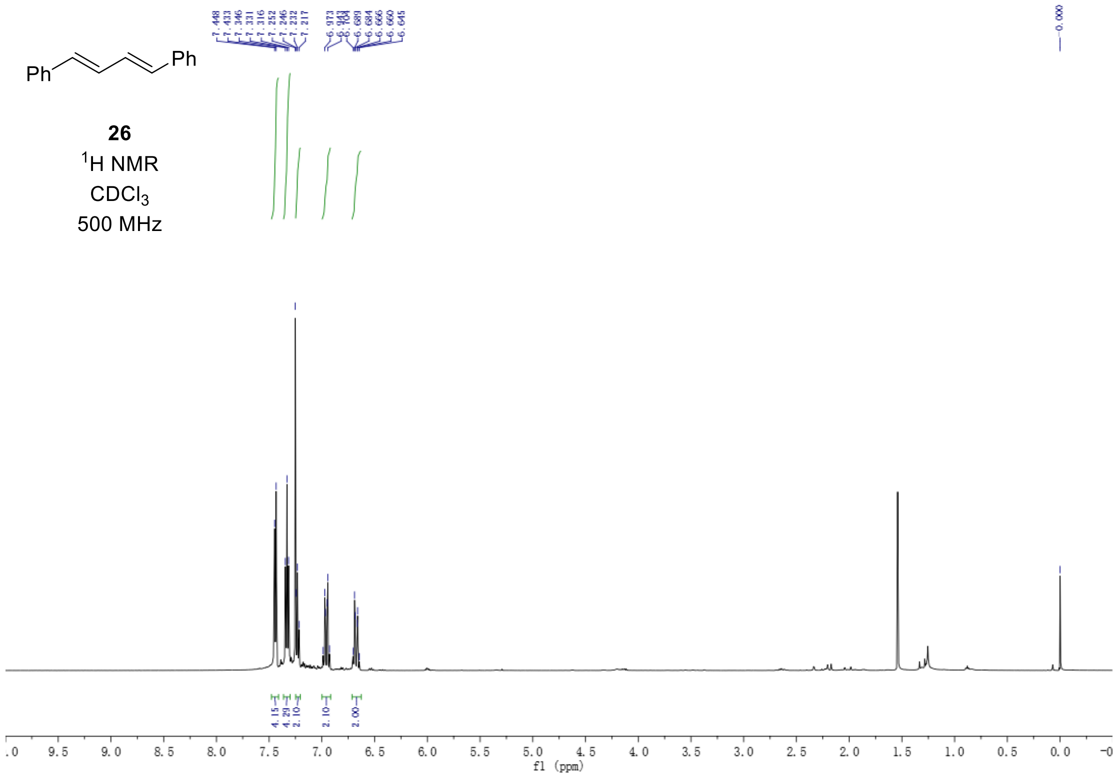

Supplementary Fig. 163 ^{1}H NMR spectrum of compound **26**

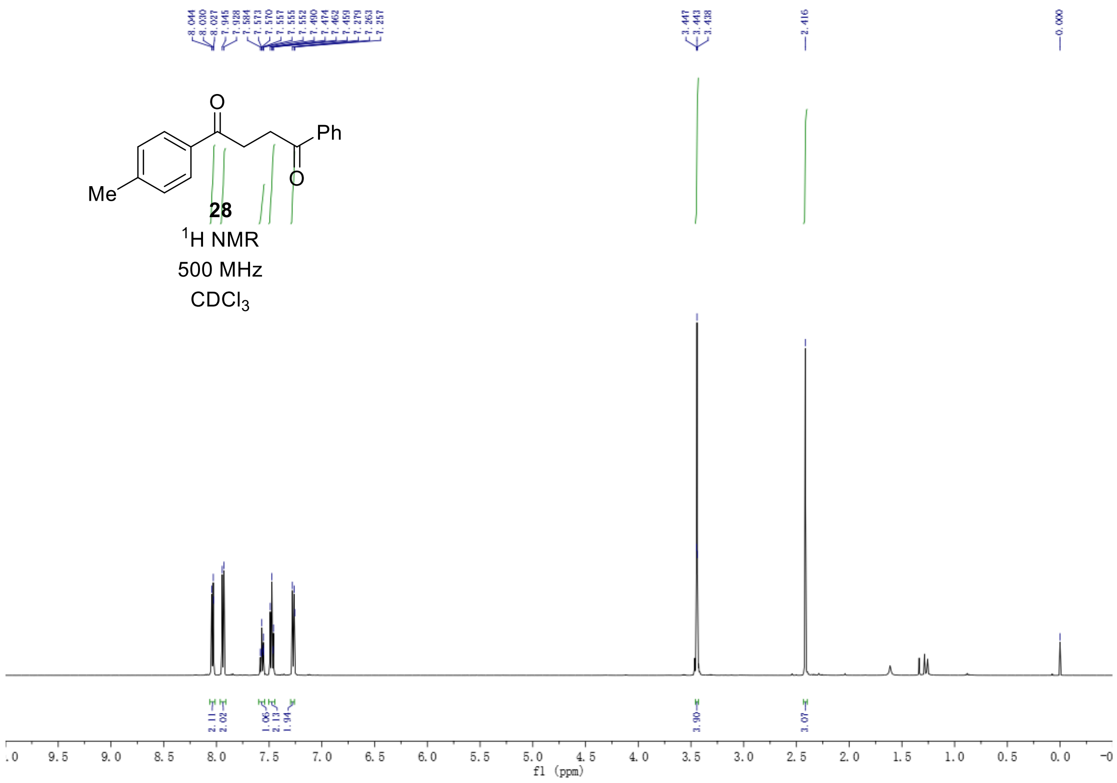

Supplementary Fig. 164 ^{1}H NMR spectrum of compound **28**

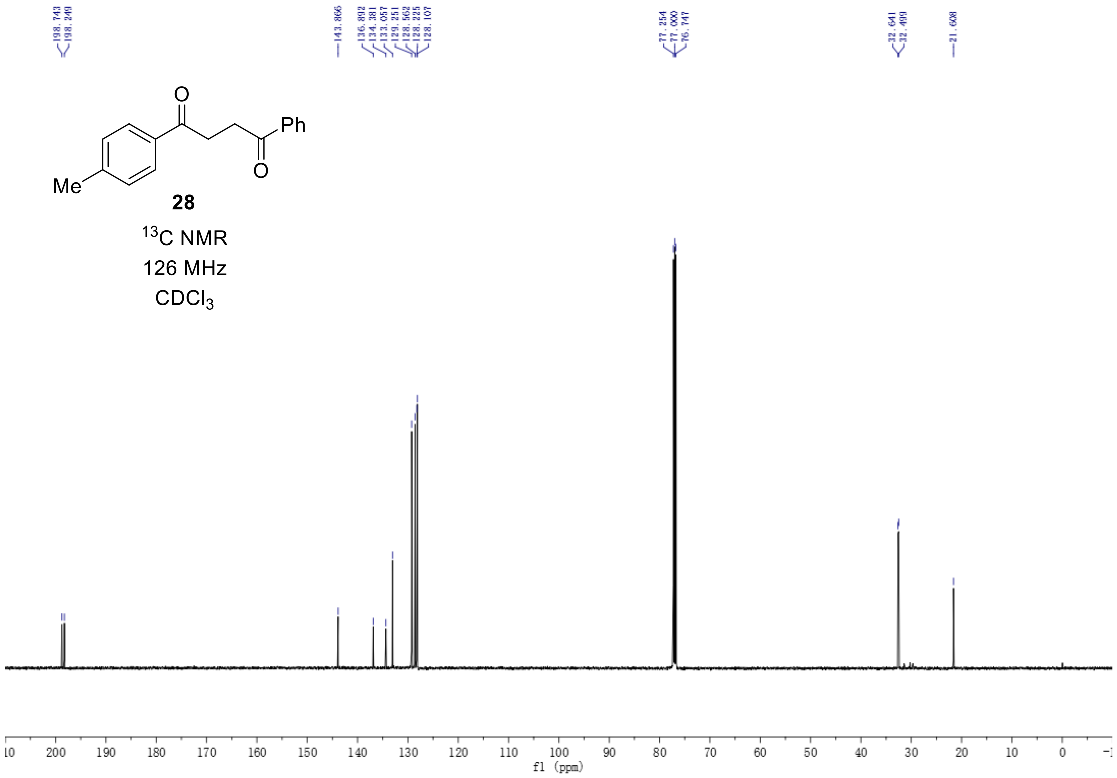

Supplementary Fig. 165 ^{13}C NMR spectrum of compound **28**

Comment: MD, *n*-hexane/*i*-PrOH = 100/0, 1.0 mL/min, 254 nm

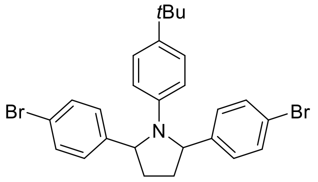

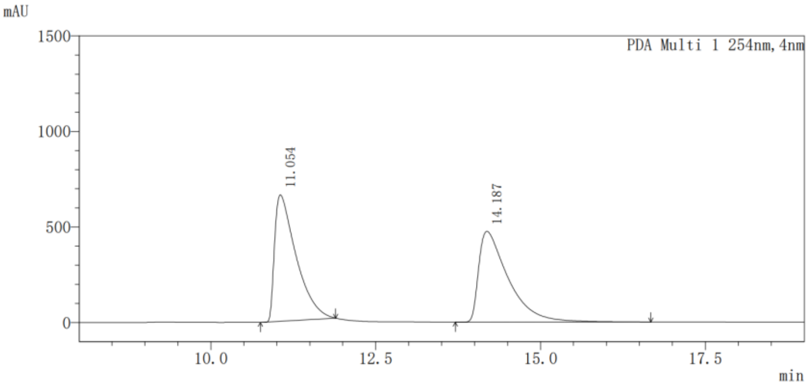

〈峰表〉
PDA Ch1 254nm

峰号	保留时间	面积	高度	面积%	高度%
1	11.054	15055375	662135	50.250	58.205
2	14.187	14905340	475462	49.750	41.795
总计		29960715	1137597	100.000	100.000

Comment: MD, *n*-hexane/*i*-PrOH = 100/0, 1.0 mL/min, 254 nm

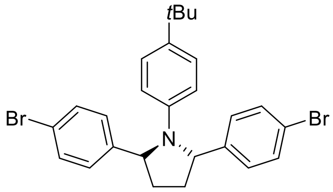

17

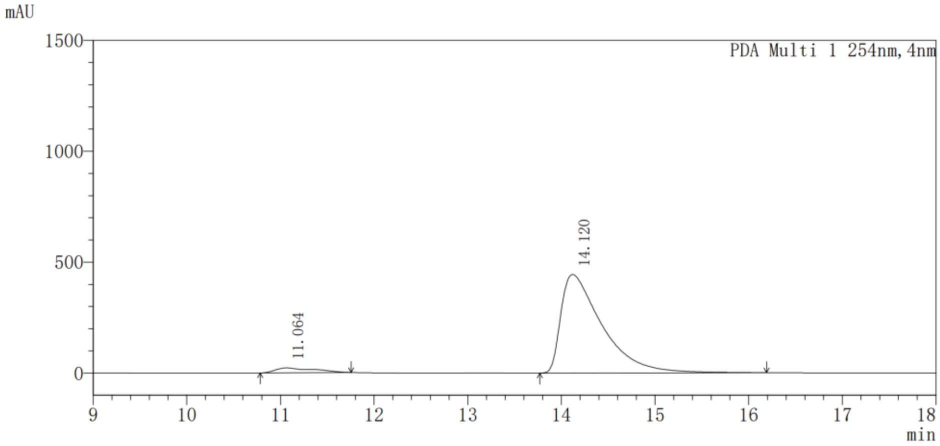

〈峰表〉
PDA Ch1 254nm

峰号	保留时间	面积	高度	面积%	高度%
1	11.064	645153	22411	4.527	4.801
2	14.120	13607322	444379	95.473	95.199
总计		14252475	466789	100.000	100.000

IV. Supplementary References

[1] Hamilton, D. S. & Nicewicz, D. A. Direct catalytic anti-markovnikov hydroetherification of alkenols. *J. Am. Chem. Soc*. **134**, 18577-18580 (2012).

[2] Wang, J., Peng, Y.-B., Tao, N., Zeng, R. & Y. Zhao. Nickel-catalyzed, *para*-selective, radical-based alkylation of aromatic ketones. *Org. Lett.* **22**, 854-857 (2020).

[3] Prakash, R. & Gogoi, S. Copper-catalyzed C-N, C-O coupling reaction of arylglyoxylic acids with isatins. *Adv. Syn. Catal.* **358**, 3046-3049 (2016).

[4] Passera, A. & Mezzetti, A. The manganese(I)-catalyzed asymmetric transfer hydrogenation of ketones: disclosing the macrocylic privilege. *Angew. Chem. Int. Ed*. **59**, 187-191 (2020).

[5] Jayarajan, R., Das, J., Bag, S., Chowdhury, R. & Maiti, D. Diverse *meta*-C-H functionalization of arenes across different linker lengths. *Angew. Chem. Int. Ed*. **57**, 7659-7663 (2018).

[6] Patel, N. R. & Molander, G. A. Phenol derivatives as coupling partners with alkylsilicates in photoredox/nickel dual catalysis. *J. Org. Chem*. **81**, 7271-7275 (2016).

[7] Streit, U., Birbaum, F., Quattropani, A. & Bochet, C. G. Photocycloaddition of arenes and allenes. *J. Org. Chem*. **78**, 6890-6910 (2013).

[8] Xie, S., Li, D., Huang, H., Zhang, F. & Chen, Y. Intermolecular radical addition to ketoacids enabled by boron activation. *J. Am. Chem. Soc*. **141**, 16237-16242 (2019).

[9] Mo, X., Morgan, T. D. R., Ang, H. T. & Hall, D. G. Scope and mechanism of a true organocatalytic beckmann rearrangement with a boronic acid/perfluoropinacol system under ambient conditions. *J. Am. Chem. Soc*. **140**, 5264-5271 (2018).

[10] Yamaguchi, M.; Fujiwara, S. & Manabe, K. Synthesis of 2,2,5-trisubstituted 2*H*-pyrroles and 2,3,5-trisubstituted 1*H*-pyrroles by ligand-controlled site-selective dearomative C2-arylation and direct C3-arylation. *Org. Lett.* **21**, 6972-6977 (2019).

[11] Kemppainen, E. K.; Sahoo, G.; Piisola, A.; Hamza, A.; Kótai, B.; Pápai, I. & Pihko, P. M. Mukaiyama–Michael reactions with *trans*-2,5-diarylpyrrolidine catalysts: enantioselectivity arises from attractive noncovalent interactions, not from steric hindrance. *Chem. Eur. J.* **20**, 5983-5993 (2014).

[12] Das, S.; Lai, D.; Mallick, A. & Roy, S. Photo redox mediated inexpensive one-pot synthesis of 1,4-diphenyl substituted butane-1,4-dione from styrene using polyoxometalate as a catalyst. *Chemistryselect*. **1**, 691-695 (2016).

[13] Liu, Y.; Zhao, H.; Tian, G.; Du, F.; Qi, Y. & Wen, Y. A novel coupling reaction of α-halo ketones promoted by SmI_3/CuI. *RSC Adv*. **6**, 26317-26322 (2017).

[14] Hua, G.; Henry, J. B.; Li, Y.; Mount, A. R.; Slawin, A. M. Z. & Woollins, J. D. Synthesis of novel 2,5-diarylselenophenes from selenation of 1,4-diarylbutane-1,4-diones or methanol/arylacetylenes. *Org. Bio. Chem*. **8**, 1655-1660 (2010).

[15] Shintani, R.; Ito, T.; Nagamoto, M.; Otomo, H. & Hayashi, T. Palladium-catalyzed asymmetric synthesis of 2-pyrrolidinones with a quaternary carbon stereocenter. *Chem. Commun*. **48**, 9936-9938 (2012).

[16] Xuan, J.; Feng, Z.-J.; Chen, J.-R.; Lu, L.-Q. & Xiao, W.-J. Visible-light-induced C-S bond activation: facile access to 1,4-diketones from β-ketosulfones. *Chem. Eur. J*. **20**, 3045-3049 (2014).

[17] Mizar, P. & Wirth, T. Flexible stereoselective functionalizations of ketones through umpolung with hypervalent iodine reagents. *Angew. Chem. Int. Ed*. **53**, 5993-5997 (2014).

[18] Sharma, A.; Kumar, V.; Sinha, A. K. *Adv. Syn. Catal*. **2006**, *348*, 354.

[19] Choi, G.; Zhu, Q.; Miller, D. C.; Gu, C. J. & Knowles, R. R. Catalytic alkylation of remote C–H bonds enabled by proton-coupled electron transfer. *Nature* **539**, 268-271 (2016).

[20] Manfrotto, C.; Mella, M.; Freccero, M.; Fagnoni, M. & Albini, A. Photochemical synthesis of 4-oxobutanal acetals and of 2-hydroxycyclobutanone ketals. *J. Org. Chem*. **64**, 5024-5028 (1999).

[21] Dai, P.-F.; Qu, J.-P. & Kang, Y.-B. Organocatalyzed Aerobic Oxidation of Aldehydes to Acids. *Org. Lett.* **21**, 1393-1396 (2019).

[22] DeGoey, D. A. et al. Discovery of ABT-267, a Pan-Genotypic Inhibitor of HCV NS5A. *J. Med. Chem*. **57**, 2047-2057 (2014).

[23] Xu, H.; Wu, L.; Tian, J.; Wang, J.; Wang, P.; Niu, X. & Yao, X. Copper nanoparticles on ordered mesoporous carbon nitride support: a superior catalyst for homo- and cross-coupling of terminal alkynes under base-free conditions. *Eur. J. Org. Chem*. **2019**, 6690-6696 (2019).

[24] Yamaguchi, M.; Fujiwara, S. & Manabe, K. Synthesis of 2,2,5-trisubstituted 2*H*-pyrroles and 2,3,5-trisubstituted 1*H*-pyrroles by ligand-controlled site-selective dearomative C2-arylation and direct C3-arylation. *Org. Lett*. **21**, 6972-6977 (2019).

[25] Lee, H.; Yi, Y. & Jun, C.-H. Copper(II)-promoted, one-pot conversion of 1-alkynes with anhydrides or primary amines to the respective 2,5-disubstituted furans or pyrroles under microwave irradiation conditions. *Adv. Syn. Catal*. **357**, 3485-3490 (2015).

[26] Clement, J. A. & Mohanakrishnan, A. K. Synthesis and characterization of naphth-annelated thiophene analogs. *Tetrahedron* **66**, 2340-2350 (2010).

[27] Pratsch, G. & Overman, L. E. Synthesis of 2,5-diaryl-1,5-dienes from allylic bromides using visible-light photoredox catalysis. *J. Org. Chem*. **80**, 11388-11397 (2015).

[28] Bhowmik, A. & Fernandes, R. A. Iron(III)/O_2-mediated regioselective oxidative cleavage of 1-arylbutadienes to cinnamaldehydes. *Org. Lett*. **21**, 9203-9207 (2019).

[29] Wang, L.-H. & Zhao, J. Detrifluoroacetylation reaction of trifluoromethyl-β-diketones: facile method for the synthesis of succinimide derivatives and 1,4-diketones. *Eur. J. Org. Chem.* **2018**, 4345-4348 (2018).

[30] Prasanna, R., Guha, S. & Sekar, G. Proton-coupled electron transfer: transition-metal-free selective reduction of chalcones and alkynes using xanthate/formic acid. *Org. Lett.* **21**, 2650-2653 (2019).
